# Supplementary material for: Electrochemical halogen-atom transfer alkylation via α-aminoalkyl radical activation of alkyl iodides
Source: Nat Commun. 2023 Oct 26;14:6825. doi: 10.1038/s41467-023-42566-y (PMC10603137; doi:10.1038/s41467-023-42566-y)
Supplement: Supplementary file 1 — Supplementary Information [file 41467_2023_42566_MOESM1_ESM.pdf]

Supplementary Information

Electrochemical Halogen-Atom Transfer Alkylation via α-Aminoalkyl Radical Activation of Alkyl Iodides

Xiang Sun[1], Ke Zheng[1]*

[1]Key Laboratory of Green Chemistry & Technology, Ministry of Education, College of Chemistry, Sichuan University, Chengdu 610064, P. R. China

Table of Contents

1. General Experimental Details......2
2. Starting Material Synthesis......2
2.1 General procedure for the preparation of secondary and primary alkyl iodides......2
2.2 General procedure for the preparation of primary alkyl iodides......3
2.3 General procedure for the preparation of tertiary alkyl iodides......4
2.4 The preparation of biomolecules alkyl iodides......5
2.5 General procedure for the preparation of complex acrylates......5
3. Reaction Optimization......7
3.1 General procedure for the electrochemical halogen-atom transfer (*e*-XAT) conjugate alkylation reaction optimization......7
3.2 General procedure for the electrochemical halogen-atom transfer (*e*-XAT) conjugate alkylation reactions using alkyl iodides-GP1......10
3.3 General procedure for the electrochemical halogen-atom transfer (*e*-XAT) synthesis aminoacids using alkyl iodides-GP2......10
3.4 Procedure for gram scale synthesis of 3......11
3.5 Procedure for gram scale synthesis of 25......12
4. Mechanistic Studies......12
4.1. Cyclic voltammetry......12
4.2 Aminoalkyl radical *e*-XAT: effect of the amine in the activation of alkyl iodides......14
4.3 Radical clock experiments: involvement of alkyl radicals......15
4.4 Deuteration experiments–H_2O as proton source: involvement of carboanion intermediates......16
4.5 TEMPO trapping experiments: involvement of alkyl radicals......16
4.6 Trapping α-aminoalkyl radicals trapping experiments: involvement of α-aminoalkyl radicals......16
5. Characterization Data for the Electrolysis Products......19
6. NMR Spectra......46
7. References......139

1. General Experimental Details

All required fine chemicals were used directly without purification unless stated otherwise. All air and moisture sensitive reactions were carried out under nitrogen atmosphere using standard Schlenk manifold technique. All solvents were bought from *J&K Scientific* as 99.9% purity under 4 Å molecular sieves. Other commercial reagents were purchased from Bidepharm, Adamas, TCI, Aldrich, and Alfa. Reactions were monitored by thin layer chromatography (TLC) using silica gel 60 F-254 plates. Flash chromatography columns were packed with 300-400 mesh silica gel. NMR-spectra were recorded on bruker ASCENDTM (400 MHz or 600 MHz) and deuterated solvents were purchased from Adamas. ^{1}H and ^{13}C Nuclear Magnetic Resonance (NMR) spectra were acquired at various field strengths as indicated and were referenced to $CHCl_3$ (7.26 and 77.0 ppm for ^{1}H and ^{13}C respectively). ^{19}F chemicals shifts (δ) are quoted in parts per million (ppm) and were calibrated using absolute referencing to the ^{1}H NMR spectrum. ^{1}H NMR coupling constants are reported in Hertz and refer to apparent multiplicities and not true coupling constants. Data are reported as follows: chemical shift, integration, multiplicity (s = singlet, br s = broad singlet, d = doublet, t = triplet, q = quartet, m = multiplet, dd = doublet of doublets, etc.). High resolution mass spectra (HRMS) analysis was recorded on Thermo Scientific Q Exactive hybrid quadrupole-Orbitrap mass spectrometer (ESI Source) and methanol were used to dissolve the sample. Cyclic voltammograms were obtained on a CHI 600E potentiostat.

2. Starting Material Synthesis

2.1 General procedure for the preparation of secondary and primary alkyl iodides[1-8]

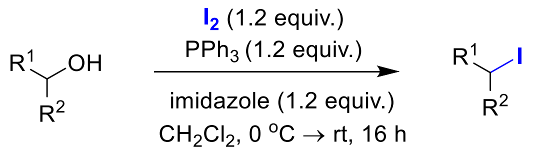

A round-bottom flask equipped with a stirring bar was charged with the corresponding alcohol (1.0 equiv.), Ph_3P (1.2 equiv.), imidazole (1.2 equiv.) and CH_2Cl_2 (0.1 M). The reaction was cooled to 0 °C with an ice-water bath, I_2 (1.2 equiv.) was added portion-wise and then the cooling bath was removed. The reaction was stirred 16 hours at room temperature and then diluted with H_2O (30 mL). The layers were separated and the aqueous layer was extracted with CH_2Cl_2 (3 x 30 mL). The combined organic layers were washed with $Na_2S_2O_3$ (30 mL, saturated), brine (30 mL), dried ($MgSO_4$), filtered and evaporated. Purification by flash column chromatography on silica gel gave the products.

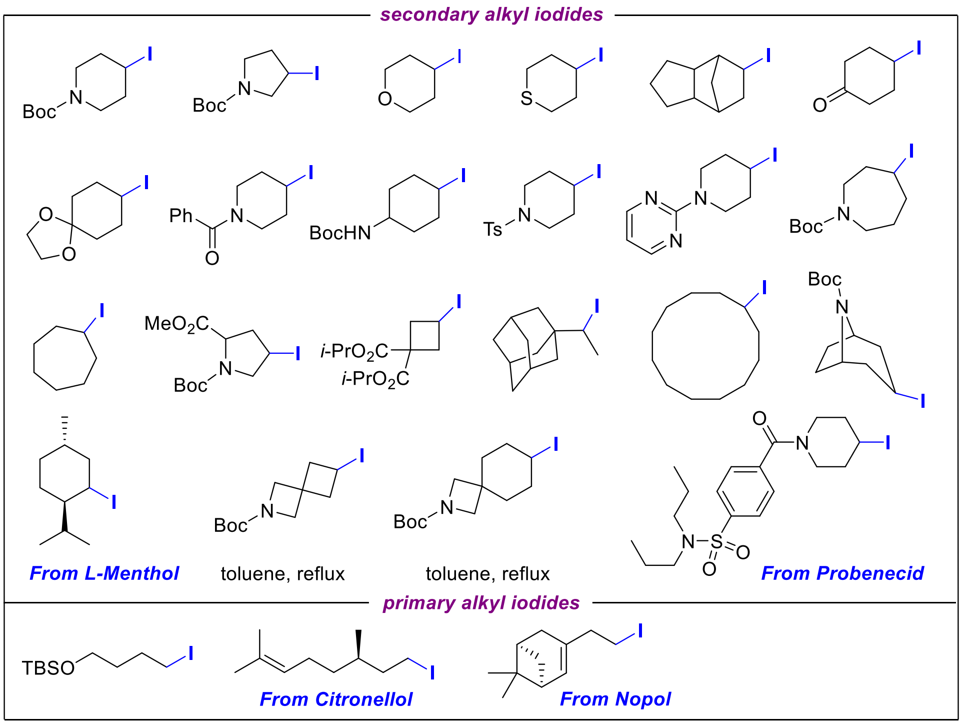

Supplementary Fig. 2.1 | The secondary and primary alkyl iodides were preparated according to the general procedure 2.1.

2.2 General procedure for the preparation of primary alkyl iodides[5]

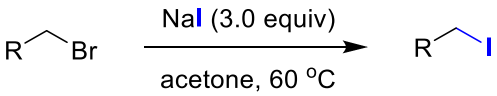

A round-bottom flask equipped with a stirring bar was charged with the corresponding alkyl-X (X = Cl, Br) (1.0 equiv), NaI (3.0 equiv.) and acetone (1 M). The reaction was was stirred at 60 °C for 12 h, then diluted with ethyl acetate, washed with saturated aqueous $Na_2S_2O_3$, and brine, dried over Na_2SO_4, and concentrated on under reduced pressure. Purification by flash column chromatography on silica gel gave the products.

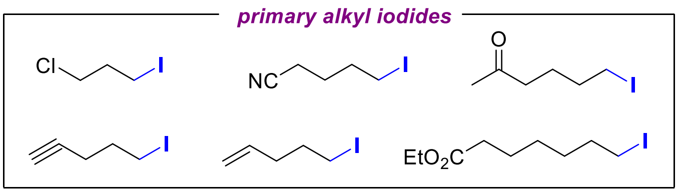

Supplementary Fig. 2.2 | The primary alkyl iodides were preparated according to the general procedure 2.2.

trans-1-Ethoxy-2-iodocyclohexane[2]

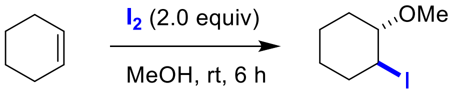

4-Iodo-1-oxaspiro[5.5]undecane[2]

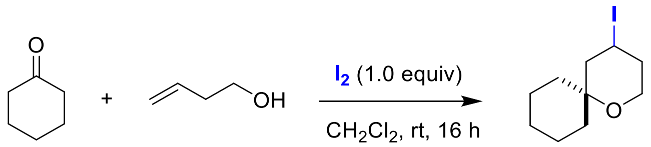

N-(4-iodooctahydropentalen-1-yl)acetamide[3]

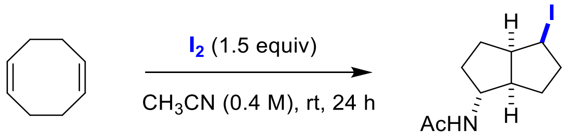

2.3 General procedure for the preparation of tertiary alkyl iodides[5]

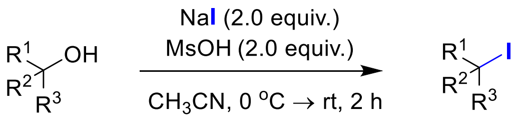

To a mixture of NaI (2.0 equiv) and tertiary alcohol (1.0 equiv) in MeCN (0.2 M) was added methylsulfonic acid (2.0 equiv) dropwise at 0 °C. The reaction mixture was allowed to warm to room temperature and stirred for 2 hours. Next, the reaction mixture was diluted with diethyl ether, washed with water, saturated aqueous $NaHCO_3$, $Na_2S_2O_3$, and brine, dried over Na_2SO_4, and concentrated on under reduced pressure. Purification by flash column chromatography on silica gel gave the products.

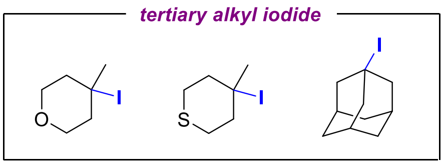

Supplementary Fig. 2.3 | The tertiary alkyl iodides were prepared according to the general procedure 2.3.

3α-iodo-1-tosyloctahydro-1H-indoles[5]

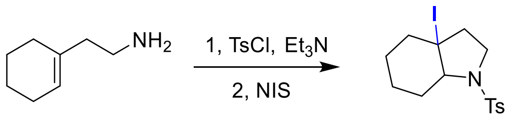

2.4 The preparation of biomolecules alkyl iodides

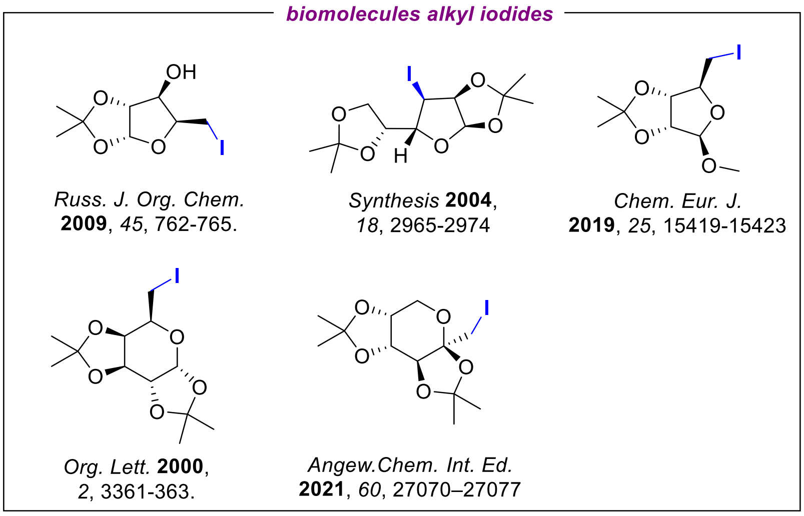

Supplementary Fig. 2.4 | The biomolecules alkyl iodides were preparated according to the reported literature.

2.5 General procedure for the preparation of complex acrylates[9]

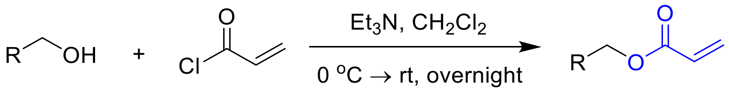

The mixture of alcohols (3.0 mmol) and Et_3N (4.5 mmol) in dry CH_2Cl_2 (10 mL) was cooled to 0 °C in an ice-water bath and acryloyl chloride (3.6 mmol) was added dropwise. The mixture was warmed to room temperature and stirred for overnight. The solvent was removed under reduced pressure and the residue was chromatographed on silica gel to get the desired product.

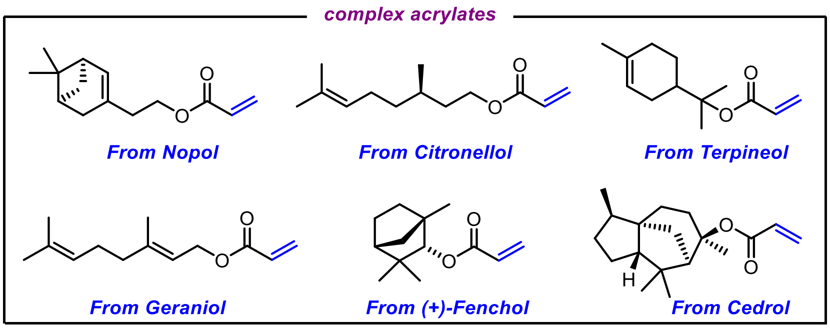

Supplementary Fig. 2.5 | The complex acrylates were preparated according to the general procedure 2.5.

Methyl 2-(di-(tert-butoxycarbonyl) amino) acrylate (dehydrated amino acid)[10-11]

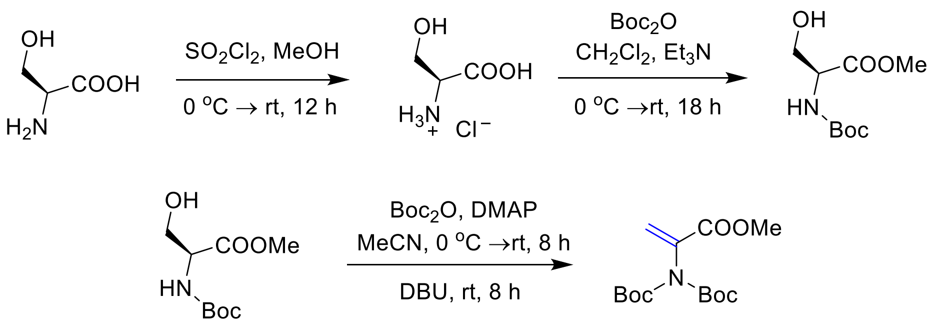

Benzyl (S)-2-(tert-butyl)-4-methylene-5-oxooxazolidine-3-carboxylate (**Karady-Beckwith alkene**) [10-11]

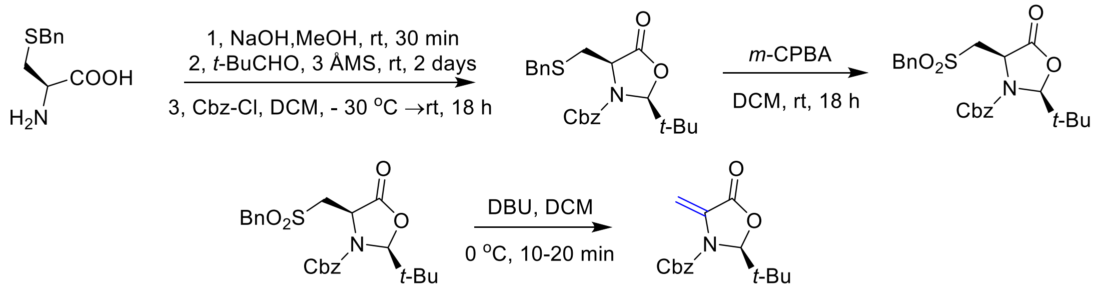

3. Reaction Optimization

3.1 General procedure for the electrochemical halogen-atom transfer (*e*-XAT) conjugate alkylation reaction optimization

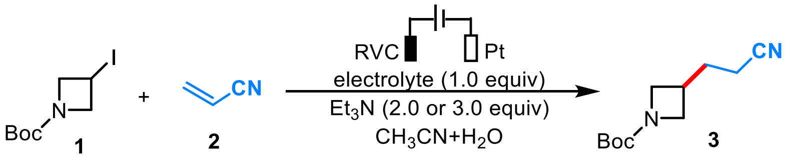

A Schlenk tube (10 mL) equipped with a magnetic stir bar was charged with the *n*-Bu_4NClO_4 (102.6 mg, 0.3 mmol, 0.1 equiv). The tube was equipped with a RVC anode (100 PPI, 1.0 cm x 1.0 cm x 1.2 cm) and a platinum plate (1.0 cm x 1.5 cm) cathode (**Fig. S1**), then sealed, evacuated and refilled with N_2 three times. Degassed solvent (2.0 mL), *tert*-butyl 3-iodoazetidine-1-carboxylate (84.9 mg, 0.3 mmol, 1.0 equiv), acrylonitrile, Et_3N, distilled water and degassed solvent (2.5 mL) were sequentially added. The electrolysis was carried out at 50 ^{o}C (oil bath temperature) using a constant current of 3.0 mA for 28 h. Upon completion, the reaction mixture was diluted with EtOAc (30 mL), 1,3,5-trimethoxybenzene (16.8 mg, 0.3 mmol, 0.1 equiv) was added, washed with H_2O (30 mL) and the aqueous layer was extracted with EtOAc (10 mL). The combined organic layers were dried over Na_2SO_4, filtered and concentrated under reduced pressure. The mixture was analysed by ^{1}H NMR spectroscopy to determine the NMR yield.

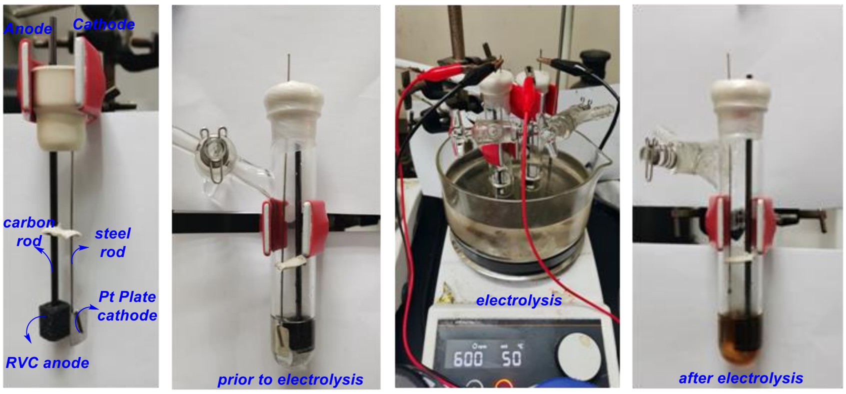

Supplementary Fig. 3.1-1 | Electrolysis setup for milligram scale reaction.

Supplementary Tab 1: Optimization of the reaction conditions.

Electrolyte	Amine	Electrode	Solvent(mL)	Additive(mL)	Current	t/h	Yields[c]
n-Bu_4NBF_4	Et_3N	RVC(+)-Pt(-)	CH_3CN(4.0)	H_2O(0.4)	6.0 mA	23	36%
n-Bu_4NBF_4	Et_3N	RVC(+)-Pt(-)	CH_3CN	H_2O(0.2)	3.0 mA	24	68%
n-Bu_4NBF_4	Et_3N	RVC(+)-Pt(-)	CH_3CN	H_2O(0.4)	3.0 mA	24	28%
n-Bu_4NPF_6	Et_3N	RVC(+)-Pt(-)	CH_3CN	H_2O(0.4)	3.0 mA	24	40%
n-Bu_4NBF_4	Et_3N	RVC(+)-Pt(-)	CH_3CN	H_2O(0.1)	3.0 mA	29	67%
n-Bu_4NClO_4	Et_3N	RVC(+)-Pt(-)	CH_3CN	H_2O(0.1)	3.0 mA	29	56%
n-Bu_4NClO_4	Et_3N	RVC(+)-Pt(-)	CH_3CN	H_2O(0.2)	3.0 mA	29	55%
n-Bu_4NClO_4	Et_3N	RVC(+)-Pt(-)	CH_3CN	H_2O(0.3)	3.0 mA	29	59%

n-Bu_4NClO_4	Et_3N	RVC(+)-Pt(-)	CH_3CN	H_2O(0.4)	3.0 mA	28	33%
n-Bu_4NClO_4	Et_3N	RVC(+)-Pt(-)	CH_3CN	H_2O(0.2)	6.0 mA	29	68%
n-Bu_4NClO_4	Et_3N	RVC(+)-Pt(-)	CH_3CN	H_2O(0.4)	6.0 mA	29	60%
n-Bu_4NClO_4	Et_3N	RVC(+)-Pt(-)	CH_3CN	H_2O(0.5)	6.0 mA	29	27%
n-Bu_4NClO_4	Et_3N	RVC(+)-Pt(-)	CH_3CN	H_2O(0.4)	8.0 mA	29	26%
n-Bu_4NClO_4	Et_3N	RVC(+)-Pt(-)	CH_3CN	H_2O(0.2)	6.0 mA	28	31%[a]
n-Bu_4NClO_4	Et_3N(3.0)	RVC(+)-Pt(-)	CH_3CN	H_2O(0.4)	6.0 mA	28	64%
n-Bu_4NClO_4	Et_3N(3.0)	RVC(+)-Pt(-)	CH_3CN	H_2O(0.4)	3.0 mA	24	79%
n-Bu_4NBF_4	Et_3N(3.0)	RVC(+)-Pt(-)	CH_3CN	H_2O(0.1)	6.0 mA	23	46%
n-Bu_4NBF_4	Et_3N(3.0)	RVC(+)-Pt(-)	CH_3CN	H_2O(0.2)	6.0 mA	23	48%
n-Bu_4NBF_4	Et_3N(3.0)	RVC(+)-Pt(-)	CH_3CN	H_2O(0.45)	3.0 mA	24	70%
n-Bu_4NClO_4	Et_3N(3.0)	RVC(+)-Pt(-)	CH_3CN	H_2O(0.45)	3.0 mA	24	76%
n-Bu_4NClO_4	Et_3N(3.0)	RVC(+)-Pt(-)	CH_3CN	H_2O(0.4)	3.0 mA	28	73%
n-Bu_4NClO_4	Et_3N(3.0)	RVC(+)-Mg(-)	CH_3CN	H_2O(0.4)	3.0 mA	28	15%
n-Bu_4NPF_6	Et_3N(3.0)	RVC(+)-Pt(-)	CH_3CN	H_2O(0.4)	3.0 mA	28	61%
Et_4NPF_6	Et_3N(3.0)	RVC(+)-Pt(-)	CH_3CN	H_2O(0.4)	3.0 mA	28	16%
n-Bu_4NClO_4	Et_3N(3.0)	RVC(+)-Zn(-)	CH_3CN	H_2O(0.45)	3.0 mA	28	41%
n-Bu_4NClO_4	Et_3N(3.0)	RVC(+)-Pt(-)	CH_3CN	H_2O(0.45)	2.0 mA	28	52%
n-Bu_4NClO_4	Et_3N(3.0)	RVC(+)-Pt(-)	CH_3CN	H_2O(0.45)	4.0 mA	28	60%
n-Bu_4NClO_4	Et_3N(3.5)	RVC(+)-Pt(-)	CH_3CN	H_2O(0.45)	3.0 mA	28	71%
n-Bu_4NClO_4	Et_3N(4.0)	RVC(+)-Pt(-)	CH_3CN	H_2O(0.45)	3.0 mA	27	80%
n-Bu_4NClO_4	Et_3N(3.0)	RVC(+)-Pt(-)	CH_3CN	H_2O(0.45)	3.0 mA	27	86%[b]
n-Bu_4NClO_4	Et_3N(3.0)	RVC(+)-Pt(-)	CH_3CN	H_2O(0.45)	3.0 mA	28	89%[b]
n-Bu_4NClO_4	Et_3N(3.0)	RVC(+)-Pt(-)	CH_3CN	—	3.0 mA	28	73%
n-Bu_4NClO_4	—	RVC(+)-Pt(-)	CH_3CN	H_2O(0.45)	3.0 mA	28	—
n-Bu_4NClO_4	Et_3N(3.0)	RVC(+)-Pt(-)	CH_3CN	H_2O(0.45)	—	28	—

Reaction conditions: Undivided cell, RVC anode (100 PPI), Pt plate cathode, **1** (0.3 mmol), **2** (0.6 mmol), CH_3CN (x mL), H_2O (x mL), N_2, X mA, rt, 23-28 h. [a] Reaction was run in the using acrylonitrile (3.0 equiv). [b] Reaction was run at 50 °C (oil bath temperature). [c] Yield determined by ^{1}H-NMR analysis using 1,3,5-trimethoxy benzene as the internal standard.

Supplementary Tab 2: Optimization of the reaction conditions under divided cell[a]

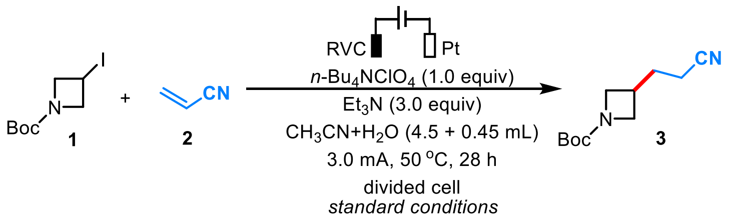

Entry	Anodic chamber	Cathodic chamber	Yield
1	**1 + 2**, Et_3N, n-Bu_4NClO_4, CH_3CN+H_2O	n-Bu_4NClO_4, CH_3CN+H_2O	nr.
2	**1**, Et_3N, n-Bu_4NClO_4, CH_3CN+H_2O	**2**, n-Bu_4NClO_4, CH_3CN+H_2O	nr.
3	n-Bu_4NClO_4, CH_3CN+H_2O	**1 + 2**, Et_3N, n-Bu_4NClO_4, CH_3CN+H_2O	nr.

[a] divided cell, RVC anode (100 PPI), Pt plate cathode, **1** (1.0 equiv, 0.3 mmol), **2** (2.0 equiv, 0.6 mmol), n-Bu_4NClO_4 (1.0 equiv, 0.3 mmol), Et_3N (3.0 equiv, 0.9 mmol), CH_3CN (4.5 mL),

H_2O (0.45 mL), N_2, 3.0 mA, 50 °C.

A H-type divided cell was equipped with a magnetic stir bar in each chamber and conducted the electrolysis in with three methods.

1. The anodic chamber was then charged with **1** (1.0 equiv, 0.3 mmol), **2** (2.0 equiv, 0.6 mmol), Et_3N (3.0 equiv, 0.9 mmol), both chambers were charged with *n*-Bu_4NClO_4 (1.0 equiv, 0.3 mmol), CH_3CN (4.5 mL), H_2O (0.45 mL), a RVC anode (100 PPI) and Pt plate cathode were installed as the anode and cathode. The electrolysis was carried out at 50 °C (oil bath temperature) using a constant current of 3.0 mA for 28 h.

2. The anodic chamber was then charged with **1** (1.0 equiv, 0.3 mmol), Et_3N (3.0 equiv, 0.9 mmol), the cathodic chamber was then charged with **2** (2.0 equiv, 0.6 mmol), both chambers were charged with *n*-Bu_4NClO_4 (1.0 equiv, 0.3 mmol), CH_3CN (4.5 mL), H_2O (0.45 mL), a RVC anode (100 PPI) and Pt plate cathode were installed as the anode and cathode. The electrolysis was carried out at 50 °C (oil bath temperature) using a constant current of 3.0 mA for 28 h.

3. The cathodic chamber was then charged with **1** (1.0 equiv, 0.3 mmol), **2** (2.0 equiv, 0.6 mmol), Et_3N (3.0 equiv, 0.9 mmol), both chambers were charged with *n*-Bu_4NClO_4 (1.0 equiv, 0.3 mmol), CH_3CN (4.5 mL), H_2O (0.45 mL), a RVC anode (100 PPI) and Pt plate cathode were installed as the anode and cathode. The electrolysis was carried out at 50 °C (oil bath temperature) using a constant current of 3.0 mA for 28 h.

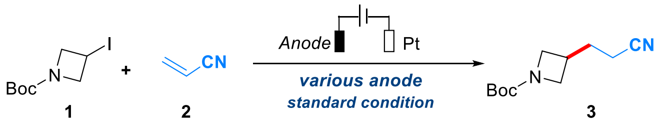

Anode	RVC	Carbon felt (*CF*)	Carbon cloth	Carbon rod	Graphite felt (*GF*)	Pt
with Et_3N	89%	82%	80%	68%	67%	42%
without Et_3N	0	0	0	0	0	0

Reaction conditions: Undivided cell, various anode, Pt plate cathode, **1** (0.3 mmol), **2** (0.6 mmol), CH_3CN (4.5 mL), H_2O (0.45 mL), N_2, 3.0 mA, 50 °C (oil bath temperature), 28 h. Yield determined by 1H-NMR analysis using 1,3,5-trimethoxy benzene as the internal standard.

RVC (100 PPI, 1.0 cm x 1.0 cm x 1.2 cm) **Graphite felt** (1.0 cm x 1.5 cm x 0.2 cm) **Carbon felt** (1.0 cm x 1.5 cm x 0.4 cm) **Carbon rod** (ϕ 0.6 cm, about 1.5 cm immersion depth in solution) **Carbon cloth** (1.0 cm x 1.5 cm) **Pt** (1.0 cm x 1.5 cm)

Supplementary Fig. 3.1-2 | Reaction optimization: effect of the different electrode materials as anodes

In response, we have carried out additional experiments with other electrode materials as anodes, such as Carbon felt (CF), Carbon cloth, Carbon rod, Graphite felt (GF), and Pt under standard electrochemical conditions. The results revealed that RVC anode was the best electrode materials and the desired alkylation product **3** was obtained in 89% yield. The use of Carbon felt (CF) and Carbon cloth as anodes led to a slightly drop in reaction efficiency (82% yield). The yield of the desired product was further decreased with using Carbon rod and Graphite felt (GF) as anodes (68% and 67%, respectively). The Pt anode displayed even lower efficacy (42% yield).

Notably, we conducted control experiments with different anode materials in the absence of Et_3N and the reaction was completely abolished, the results showed that triethylamine (Et_3N) was the key for this transformation and not the anode materials. The efficiency of Et_3N was oxidized at different anode materials could be affected the reaction results. So, we think that the Et_3N underwent one-electron oxidation and deprotonation to generate α-aminoalkyl radical was the key for this transformation and the process was highly possible.

3.2 General procedure for the electrochemical halogen-atom transfer (*e*-XAT) conjugate alkylation reactions using alkyl iodides-GP1

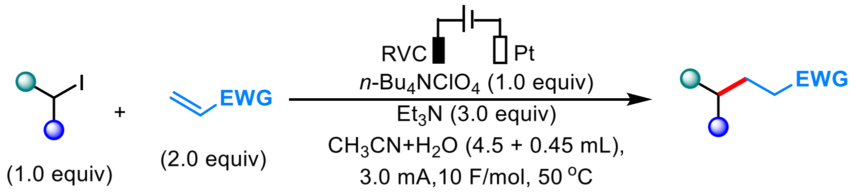

A Schlenk tube (10 mL) equipped with a magnetic stir bar was charged with the *n*-Bu_4NClO_4 (102.6 mg, 0.3 mmol, 1.0 equiv), alkyl iodide, if solid (0.3 mmol, 1.0 equiv.), and the Michael acceptor, if solid (0.6 mmol, 2.0 equiv.). The tube was equipped with a RVC anode (100 PPI, 1.0 cm x 1.0 cm x 1.2 cm) and a platinum plate (1.0 cm x 1.5 cm) cathode (Supplementary **Fig. 3.1-1**), then sealed, evacuated and refilled with N_2 three times. Degassed CH_3CN (2.0 mL) (The alkyl iodide or Michael acceptor if liquid, added at this step), Et_3N (91.1 mg, 0.9 mmol, 3.0 equiv), distilled water (0.45 mL) and degassed CH_3CN (2.5 mL) were sequentially added. The electrolysis was carried out at 50 °C (oil bath temperature) using a constant current of 3.0 mA for 28 h. Upon completion, the reaction mixture was diluted with EtOAc (30 mL), washed with H_2O (30 mL) and the aqueous layer was extracted with EtOAc (10 mL). The combined organic layers were dried over Na_2SO_4, filtered and concentrated under reduced pressure. The mixture was purified by flash column chromatography on silica gel.

3.3 General procedure for the electrochemical halogen-atom transfer (*e*-XAT) synthesis aminoacids using alkyl iodides-GP2

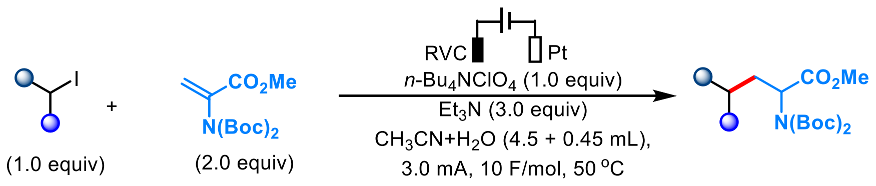

A Schlenk tube (10 mL) equipped with a magnetic stir bar was charged with the *n*-Bu_4NClO_4 (102.6 mg, 0.3 mmol, 1.0 equiv), alkyl iodide, if solid (0.3 mmol, 1.0 equiv.), and methyl 2-(bis(*tert*-butoxycarbonyl)amino)acrylate (180.8 mg, 0.6 mmol, 2.0 equiv.). The tube was equipped with a RVC anode (100 PPI, 1.0 cm x 1.0 cm x 1.2 cm) and a platinum plate (1.0 cm x 1.5 cm) cathode (Supplementary **Fig. 3.1-1**), then sealed, evacuated and refilled with N_2 three times. Degassed CH_3CN (2.0 mL), (alkyl iodide if liquid, added this step), Et_3N (91.1 mg, 0.9 mmol, 3.0 equiv), distilled water

(0.45 mL) and degassed CH_3CN (2.5 mL) were sequentially added. The electrolysis was carried out at 50 °C (oil bath temperature) using a constant current of 3.0 mA for 28 h (10 F/mol, for secondary alkyl iodide) or 48 h **(**18 F/mol, for primary or tertiary alkyl iodide). Upon completion, the reaction mixture was diluted with EtOAc (30 mL), washed with H_2O (30 mL) and the aqueous layer was extracted with EtOAc (10 mL). The combined organic layers were dried over Na_2SO_4, filtered and concentrated under reduced pressure. The mixture was purified by flash column chromatography on silica gel.

3.4 Procedure for gram scale synthesis of 3

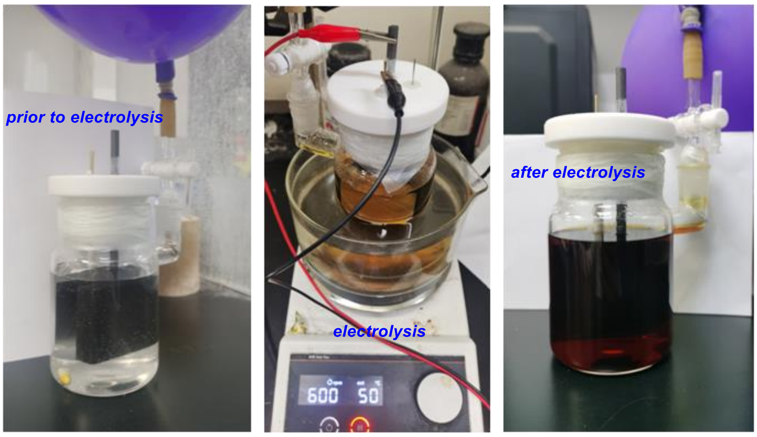

Supplementary Fig. 3.4 | Electrolysis setup for gram scale reaction.

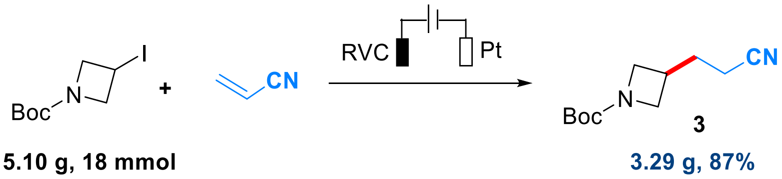

The electrolysis was conducted in a 250 mL beaker-type cell with a RVC (100 PPI, 5.0 cm x 5.0 cm x 1.2 cm) anode, a Pt plate cathode (5.0 cm x 5.0 cm x 0.1 cm) (Supplementary **Fig. 3.4**). The cell was charged with *n*-Bu_4NClO_4 (6.15 g, 18.0 mmol, 1.0 equiv), evacuated and refilled with N_2 three times. Then degassed CH_3CN (270 mL), *tert*-butyl 3-iodoazetidine-1-carboxylate (5.10 g, 18.0 mmol, 1.0 equiv), acrylonitrile (1.91 g, 36.0 mmol, 2.0 equiv), Et_3N (5.46 g, 54.0 mmol, 3.0 equiv), and distilled water (27 mL) sequentially added. The constant current (180 mA) electrolysis was carried out at 50 °C (oil bath temperature) for 28 h. Upon completion, the reaction mixture was diluted with EtOAc (100 mL), washed with H_2O (200 mL) and the aqueous layer was extracted with EtOAc (100 mL). The combined organic layers were dried over Na_2SO_4, filtered and concentrated under reduced pressure. The mixture was purified by flash column chromatography on silica gel to afford **3** as light yellow oil (3.29 g, 87% yield).

3.5 Procedure for gram scale synthesis of 25

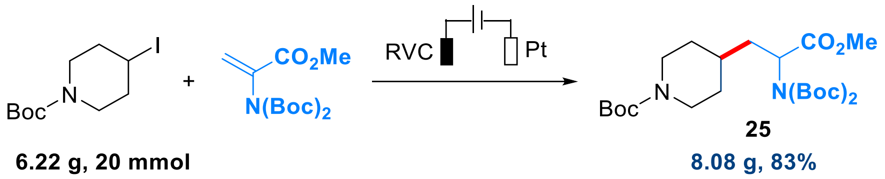

The electrolysis was conducted in a 250 mL beaker-type cell with a RVC (100 PPI, 5.0 cm x 5.0 cm x 1.2 cm) anode, a Pt plate cathode (5.0 cm x 5.0 cm x 0.1 cm) (Supplementary **Fig. 3.4**). The cell was charged with *n*-Bu_4NClO_4 (6.84 g, 20.0 mmol, 1.0 equiv), *tert*-butyl 4-iodopiperidine-1-carboxylate (6.22 g, 20.0 mmol, 1.0 equiv), methyl 2-(bis(*tert*-butoxycarbonyl)amino)acrylate (12.05 g, 40.0 mmol, 2.0 equiv.), evacuated and refilled with N_2 three times. Then degassed CH_3CN (300 mL), Et_3N (6.07 g, 60.0 mmol, 3.0 equiv), and distilled water (30 mL) sequentially added. The constant current (200 mA) electrolysis was carried out at 50 ^oC (oil bath temperature) for 28 h. Upon completion, the reaction mixture was diluted with EtOAc (100 mL), washed with H_2O (200 mL) and the aqueous layer was extracted with EtOAc (100 mL). The combined organic layers were dried over Na_2SO_4, filtered and concentrated under reduced pressure. The mixture was purified by flash column chromatography on silica gel to afford **25** as light yellow oil (8.08 g, 83% yield).

4. Mechanistic Studies

4.1. Cyclic voltammetry

The cyclic voltammograms were recorded in MeCN (5.0 mL) with *n*-Bu_4NClO_4 (0.1 M) as supporting electrolyte using a glassy carbon disk working electrode (diameter, 1 mm), a Pt wire auxiliary electrode and an Ag/AgCl reference electrode. The scan rate was 100 mV/s.

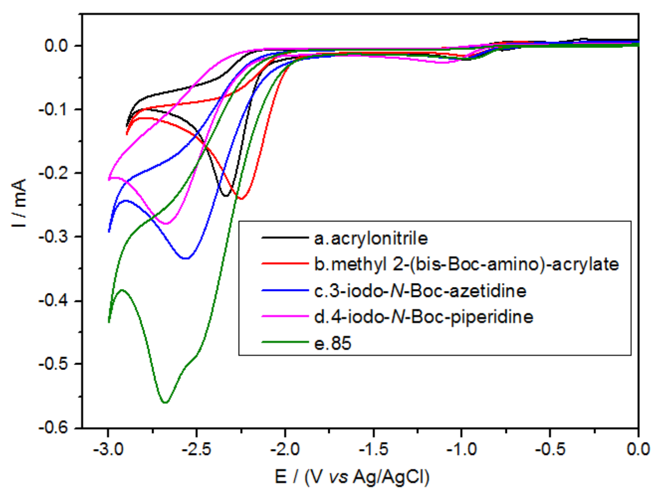

Supplementary Fig. 4.1-1 | Cyclic voltammograms of alkyl iodides and Michael acceptors. a, acrylonitrile (0.1 mmol). **b**, methyl 2-(bis-Boc-amino) acrylate (0.1 mmol). **c**, 3-iodo-N-Boc-azetidine (0.1 mmol). **d**, 4-iodo-N-Boc-piperidine (0.1 mmol). **e**, 85 (0.1 mmol).

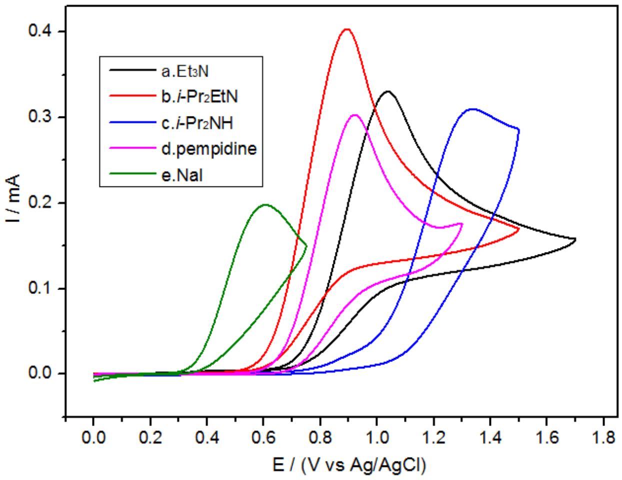

Supplementary Fig. 4.1-2 | Cyclic voltammograms of alkyl amines. a, Et_3N (0.1 mmol). **b**, *i*-Pr_2EtN (0.1 mmol). **c**, *i*-Pr_2NH (0.1 mmol). **d**, pempidine (0.1 mmol). **e**, NaI (0.1 mmol).

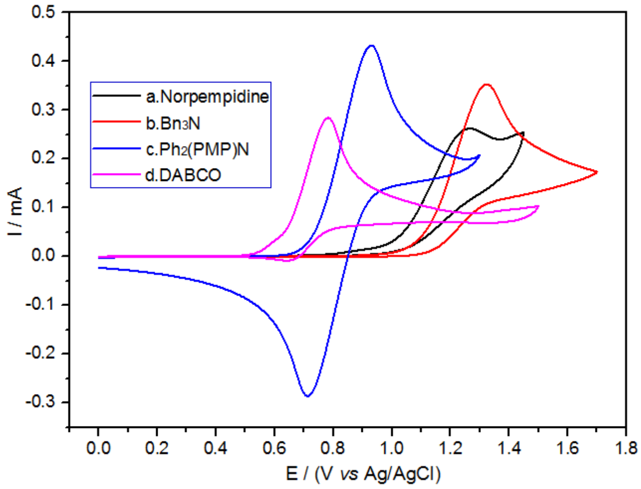

Supplementary Fig. 4.1-3 | Cyclic voltammograms of alkyl amines. a, norpempidine (0.1 mmol). **b**, Bn_3N (0.1 mmol). **c**, $Ph_2(PMP)N$ (0.1 mmol). **d**, DABCO (0.1 mmol).

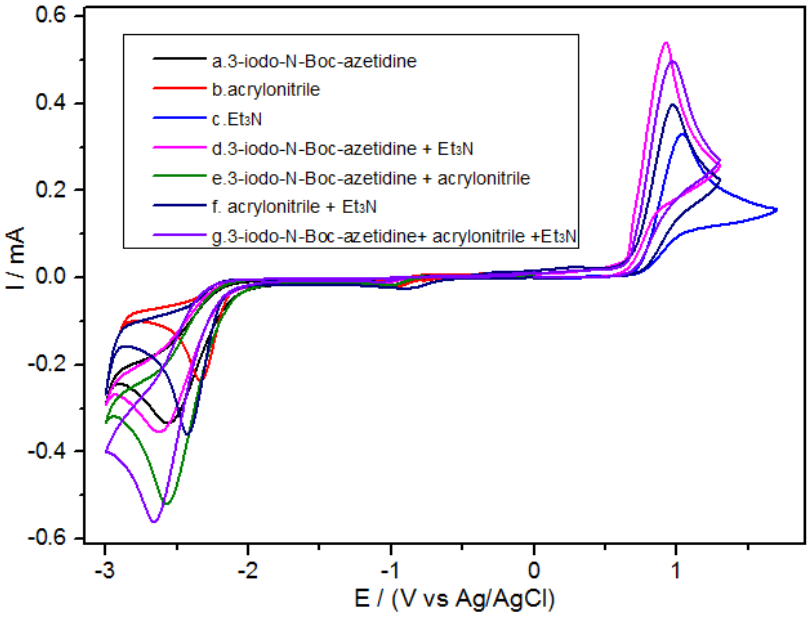

Supplementary Fig. 4.1-4 | Cyclic voltammograms of e-*XAT* Conjugate Alkylation. **a**, 3-iodo-N-Boc-azetidine (0.1 mmol). **b**, acrylonitrile (0.1 mmol). **c**, Et_3N (0.1 mmol). **d**, 3-iodo-N-Boc-azetidine (0.1 mmol) + Et_3N (0.1 mmol). **e**, 3-iodo-N-Boc-azetidine (0.1 mmol) + acrylonitrile (0.1 mmol). **f**, acrylonitrile (0.1 mmol) + Et_3N (0.1 mmol). **g**, 3-iodo-N-Boc-azetidine (0.1 mmol) + acrylonitrile (0.1 mmol) + Et_3N (0.1 mmol).

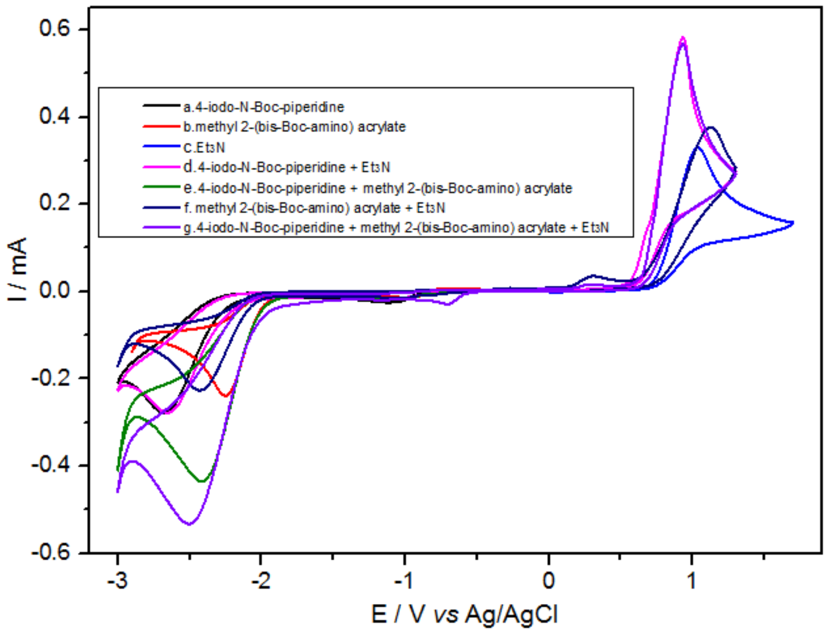

Supplementary Fig. 4.1-5 | Cyclic voltammograms of e-*XAT* Synthesis Aminoacids. **a**, 4-iodo-N-Boc-piperidine (0.1 mmol). **b**, methyl 2-(bis-Boc-amino) acrylate (0.1 mmol). **c**, Et_3N (0.1 mmol). **d**, 4-iodo-N-Boc-piperidine (0.1 mmol) + Et_3N (0.1 mmol). **e**, 4-iodo-N-Boc-piperidine (0.1 mmol) + methyl 2-(bis-Boc-amino) acrylate (0.1 mmol). **f**, methyl 2-(bis-Boc-amino) acrylate (0.1 mmol) + Et_3N (0.1 mmol). **g**, 4-iodo-N-Boc-piperidine (0.1 mmol) + methyl 2-(bis-Boc-amino) acrylate (0.1 mmol) + Et_3N (0.1 mmol).

4.2 Aminoalkyl radical *e*-XAT: effect of the amine in the activation of alkyl iodides

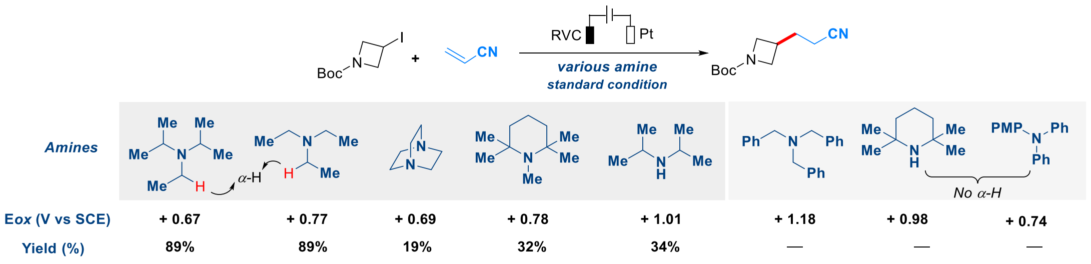

Supplementary Fig. 4.2 | Effect of the amine in the activation of alkyl iodides.

Various amines were tested as XAT-agent precursors under standard electrochemical conditions. The results showed that alkylamines play a fundamental role in this transformation (Supplementary Fig. 4.2). Alkylamines with suitable oxidation potential and secondary α-H (e.g., Et_3N and *i*-Pr_2NEt) gave better outcomes compared to other electron donors. The use of 2,2,6,6-tetramethyl-N-methylpiperidine with primary α-hydrogens and *i*-Pr_2NH with tertiary α-hydrogens resulted in a

noticeable reduction in reaction efficiency. These outcomes might be attributed to the primary or tertiary α-aminoalkyl radicals were more difficult to generate during the initiating stage (electrochemical oxidation stage), while secondary α-aminoalkyl radicals demonstrated better stability (compared to primary α-aminoalkyl radicals) and encountered less steric hindrance (compared to tertiary α-aminoalkyl radicals) in the XAT step. The replacement of Et_3N with alkylamines without *a*-H (those unable to generate α- aminoalkyl radicals), such as Ph_2N(PMP) or 2,2,6,6-tetramethylpiperidine, completely suppressed the reactivity. Similarly, tribenzylamine was unable to initiate the reaction due to its high oxidation potential (E_{ox} = +1.18 V vs SCE).

4.3 Radical clock experiments: involvement of alkyl radicals

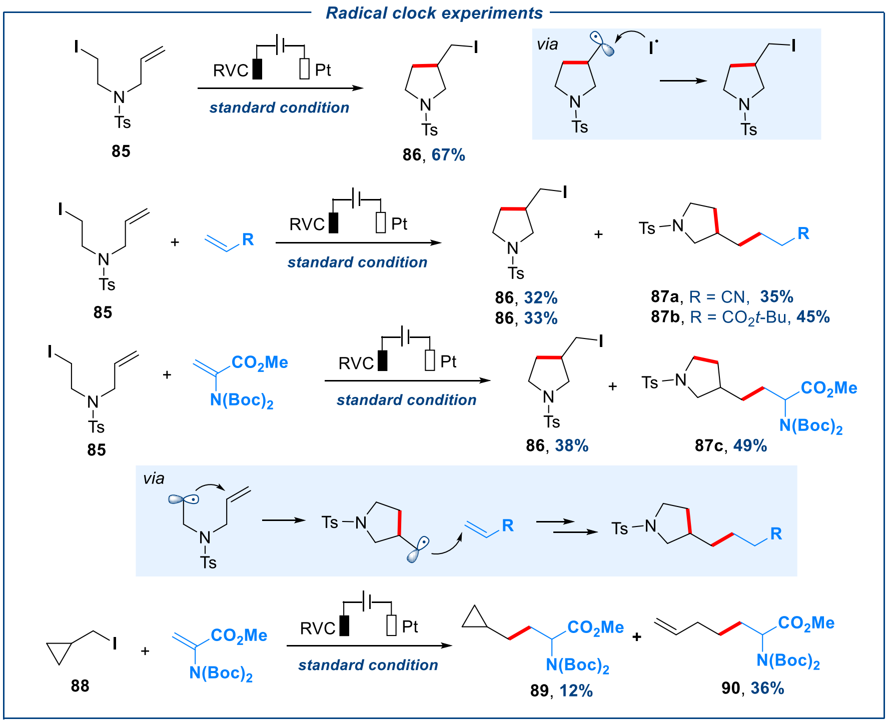

Supplementary Fig. 4.3 | Radical clock experiments.

When the radical probe substrate **85** was subjected to standard conditions, the cyclic product **86** was obtained in moderate yield with no acyclic product detected (Supplementary Fig. 4.3, top). This was likely due to a sequence of halogen-atom transfers, intramolecular alkyl radical additions, and iodide radical trapping. Furthermore, the addition of electron-poor olefins to the reaction resulted in moderate yields of the alkylation products **87a**, **87b** and **87c**, along with **86** (Supplementary Fig. 4.3, middle). These results provided direct evidence that the reaction underwent a radical-polar crossover pathway. Additional evidence was obtained from a free radical clock experiment using cyclopropyl-containing alkyl iodide **88** as a substrate, which produced the ring-opened product **89** in 36% yield with 12% yield of unrearranged product **90** (Supplementary Fig. 4.3, bottom). This indicated that the rate of intermolecular radical trapping by electron-poor olefins was very fast.

4.4 Deuteration experiments–H_2O as proton source: involvement of carboanion intermediates

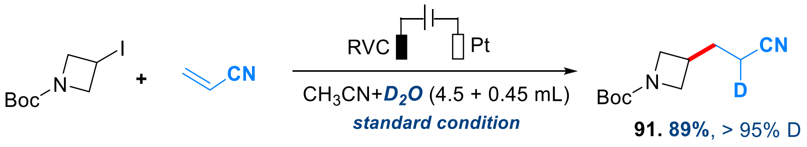

Supplementary Fig. 4.4 | Deuteration experiments–H_2O as proton source.

The use of D_2O instead of H_2O under standard conditions yielded the deuterated product **91** in 89% yield, suggesting that the carbanion intermediates were generated by cathode reduction and that H_2O acted as a H-atom source in this electrochemical process (Supplementary Fig. 4.4).

4.5 TEMPO trapping experiments: involvement of alkyl radicals

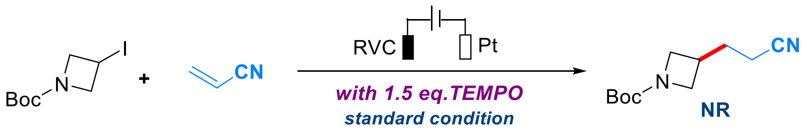

Supplementary Fig. 4.5 | TEMPO trapping experiments.

Moreover, the reaction was completely abolished when TEMPO (1.5 equiv) was added under standard electrochemical conditions (Supplementary Fig. 4.5). We speculated that the oxidation of TEMPO was more easily than the alkylamine. The anodic oxidation of TEMPO to generate $TEMPO^+$ and the oxidation of alkylamine could be completely suppressed. Subsequently, the $TEMPO^+$ could also be reduced at the cathode to form TEMPO. So, the desired alkylation products were not observed.

4.6 Trapping α-aminoalkyl radicals experiments: involvement of α-aminoalkyl radicals

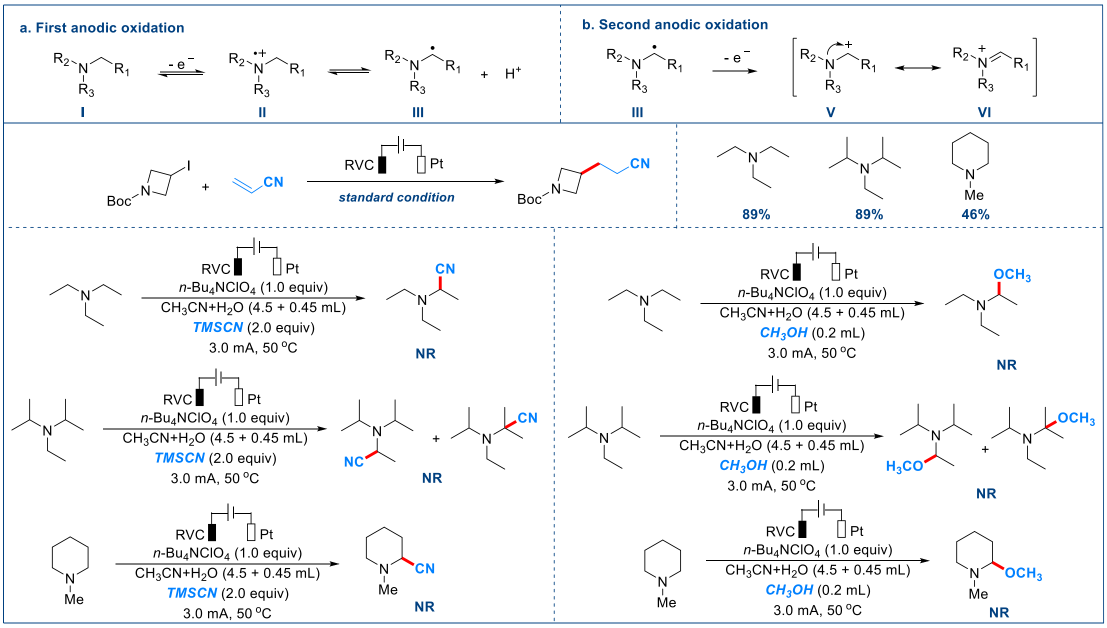

Supplementary Fig. 4.6-1 | Trapping α-aminoalkyl radicals experiments with TMSCN and CH_3OH as nucleophiles.

However, this electrochemical oxidation process could be involved in two steps. Initially, the alkylamine undergoes one-electron anodic oxidation to generate radical cation II, which subsequently undergoes deprotonates to yield α-aminoalkyl radical III (as depicted in Supplementary Fig. **4.6-1a**). Subsequently, this radical III could undergo a secondary oxidation to generate the iminium cation **V** (as illustrated in Supplementary Fig. **4.6-1b**). In previous reports and our perspective, the activation of unactivated alkyl iodides with highly nucleophilic α-aminoalkyl radicals was highly fast, and the XAT process was kinetically feasible and exothermic based on Density functional theory calculations (*Science* **2020**, *367*, 1021−1026).

In order to investigate the possibilities if the iminium cation **V** was produced in our *e*-XAT reaction conditions, we conducted a series of control experiments involving TMSCN and CH_3OH as nucleophiles, in combination with three distinct alkylamines—Et_3N, *i*-Pr_2NEt, and 1-methylpiperidine—in the absence of alkyl iodides. The desired nucleophile addition products were not obtained. Additionally, the formation of iminium cation **V** could not be excluded completely due to 3.0 equiv Et_3N was used in our reaction.

Moreover, we conducted an investigation of different electrode materials as anodes and the reaction was completely abolished in the absence of Et_3N. The results indicated that the triethylamine (Et_3N) was critical for this transformation.

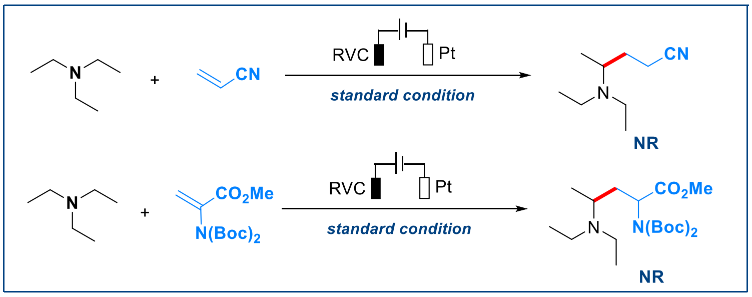

Reaction conditions: Undivided cell, RVC anode (100 PPI), Pt plate cathode, Et_3N (0.9 mmol), alkene (0.6 mmol), CH_3CN (4.5 mL), H_2O (0.45 mL), N_2, 3.0 mA, 50 °C (oil bath temperature), 28 h.

Supplementary Fig. 4.6-2 | Trapping α-aminoalkyl radicals experiments with acrylonitrile and Boc-protected dehydroalanine.

We did not observe trapping products of α-aminoalkyl radicals when acrylonitrile and Boc-protected dehydroalanine were introduced as radical acceptors under standard conditions (Supplementary Fig. **4.6-2**). Our speculation is that the reduction process following the addition of α-aminoalkyl radicals to electron-deficient olefins is highly difficult and could not deliver the desired product through H_2O protonation.

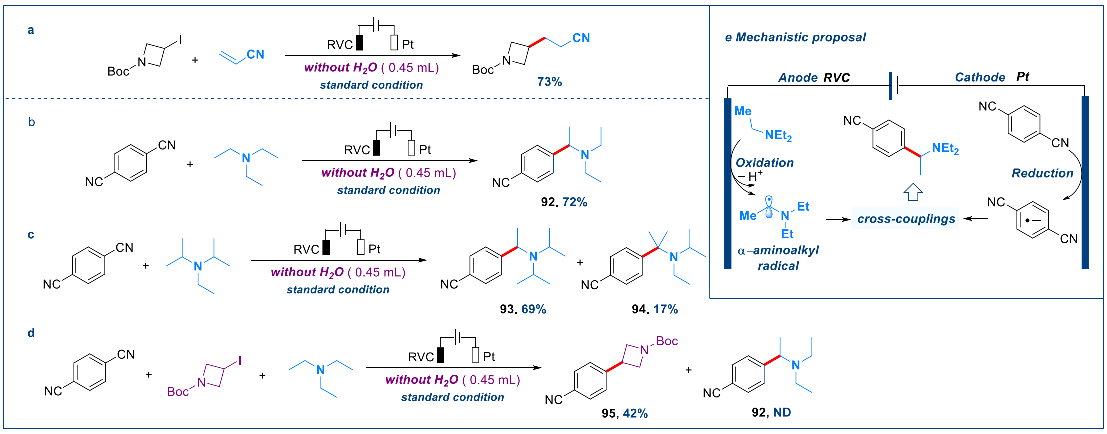

Reaction conditions: Undivided cell, RVC anode (100 PPI), Pt plate cathode, 1,4-dicyanobenzene (0.3 mmol), 3-iodo-N-Boc-azetidine (0.45 mmol), Et_3N or *i*-Pr_2EtN (0.9 mmol), CH_3CN (4.5 mL), N_2, 3.0 mA, 50 ^oC (oil bath temperature), 28 h.

Supplementary Fig. 4.6-3 | Trapping α-aminoalkyl radicals experiments with 1,4-dicyanobenzene.

To our delight, the direct evidence comes from the results: In comparison to the alkylation product obtained with a 73% yield in the absence of H_2O under standard conditions for the model reaction (Supplementary Fig. **4.6-3a**), the radical trapping products **92-94** were achieved with 72-86% yields when employing 1,4-dicyanobenzene as a radical acceptor (Supplementary Fig. **4.6-3b-c**). These results directly demonstrated the successful generation of α-aminoalkyl radicals through anodic oxidation and deprotonation. These radicals are directly captured by the radical anions of 1,4-dicyanobenzene, which are formed *via* cathodic reduction (*Angew. Chem. Int. Ed.* **2019**, 58, 4058–4062), yielding the cross-coupling products **92-94** (Supplementary Fig. **4.6-3e**).

Moreover, the cross-coupling product **95** was achieved with 42% yield when adding alkyl iodide (3-iodo-*N*-Boc-azetidine) to the reaction, arising from the coupling of alkyl radicals with 1,4-dicyanobenzene radical anions. The α-aminoalkyl radical trapping product **92** was not observed (Supplementary Fig. **4.6-3d**). These outcomes revealed that upon the generation of the α-aminoalkyl radical, the XAT process ensued immediately, leading to the formation of alkyl radicals.

5. Characterization Data for the Electrolysis Products

***tert*-butyl 3-(2-cyanoethyl)azetidine-1-carboxylate (3)**

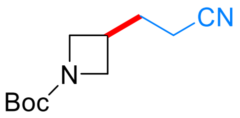

Following **GP1**, acrylonitrile (31.8 mg, 0.6 mmol) gave **3** (54.3 mg, 86%) as an oil. ^{1}H NMR (400 MHz, $CDCl_3$) δ 4.07 (2H, t, J = 8.4 Hz), 3.59 (2H, dd, J = 8.6, 5.4 Hz), 2.70 – 2.60 (1H, m), 2.35 (2H, t, J = 7.2 Hz), 1.97 (2H, q, J = 7.4 Hz), 1.44 (9H, s); ^{13}C NMR (101 MHz, $CDCl_3$) δ 156.2, 118.8, 79.6, 53.8, 29.8, 28.4, 28.0, 15.1; Data in accordance with the literature.[1]

***tert*-butyl 3-(3-methoxy-3-oxopropyl)azetidine-1-carboxylate (4)**

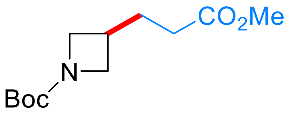

Following **GP1**, methyl acrylate (51.7 mg, 0.6 mmol) gave **4** (62.0 mg, 85%) as an oil. ^{1}H NMR (400 MHz, $CDCl_3$) δ 4.00 (2H, t, J = 8.4 Hz), 3.67 (3H, s), 3.54 (2H, dd, J = 8.6, 5.4 Hz), 2.57–2.46 (1H, m), 2.27 (2H, t, J = 7.6 Hz), 1.91 (2H, q, J = 7.6 Hz), 1.43 (9H, s); ^{13}C NMR (101 MHz, $CDCl_3$) δ 173.3, 156.3, 79.3, 54.1, 51.7, 31.5, 29.4, 28.4, 28.2; Data in accordance with the literature.[1]

***tert*-butyl 3-(3-(*tert*-butoxy)-3-oxopropyl)azetidine-1-carboxylate (5)**

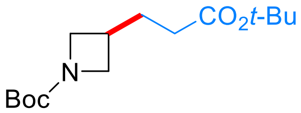

Following **GP1**, *tert*-butyl acrylate (76.9 mg, 0.6 mmol) gave **5** (64.2 mg, 75%) as an oil. ^{1}H NMR (400 MHz, $CDCl_3$) δ 3.99 (2H, t, J = 8.4 Hz), 3.55 (2H, dd, J = 8.4, 5.6 Hz), 2.54 – 2.45 (1H, m), 2.18 (2H, t, J = 7.6 Hz), 1.87 (2H, q, J = 7.4 Hz), 1.44 (18H, d, J = 4.4 Hz); ^{13}C NMR (101 MHz, $CDCl_3$) δ 172.3, 156.4, 80.5, 79.2, 54.1, 33.0, 29.6, 28.4, 28.2, 28.1; HRMS (ESI-FT): Found M+Na$^+$ 308.1833, $C_{15}H_{27}NNaO_4$ requires 308.1832.

3-(1-(*tert*-butoxycarbonyl)azetidin-3-yl)propanoic acid (6)

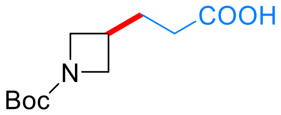

Following **GP1**, acrylic acid (43.2 mg, 0.6 mmol) gave **6** (35.5 mg, 53%) as an oil. ^{1}H NMR (400 MHz, $CDCl_3$) δ 4.03 (2H, t, J = 8.6 Hz), 3.58 (2H, dd, J = 8.4, 5.6 Hz), 2.62 – 2.51 (1H, m), 2.33 (2H, t, J = 7.4 Hz), 1.94 (2H, q, J = 7.4 Hz), 1.45 (9H, s); ^{13}C NMR (101 MHz, $CDCl_3$) δ 178.0, 156.4, 79.6, 53.7, 31.4, 29.2, 28.4, 28.2; Data in accordance with the literature.[1]

***tert*-butyl 3-(2-(phenylsulfonyl)ethyl)azetidine-1-carboxylate (7)**

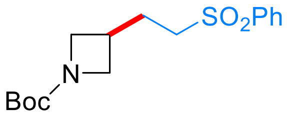

Following **GP1**, (vinylsulfonyl)benzene (100.9 mg, 0.6 mmol) gave **7** (78.1 mg, 80%)

as an oil. ^{1}H NMR (400 MHz, $CDCl_3$) δ7.90 (2H, d, J = 8.0 Hz), 7.68 (2H, t, J = 7.4 Hz), 7.58 (2H, t, J = 7.6 Hz), 4.01 (2H, t, J = 8.4 Hz), 3.49 (2H, dd, J = 8.4, 5.2 Hz), 3.03 (2H, t, J = 8.0 Hz), 2.61 – 2.52 (1H, m), 2.01 (2H, q, J = 7.6 Hz), 1.41 (9H, s); ^{13}C NMR (101 MHz, $CDCl_3$) δ 156.2, 138.9, 133.9, 129.4, 128.0, 79.5, 53.8, 28.4, 27.4, 27.2; HRMS (ESI-FT): Found M+Na$^+$ 348.1243, $C_{16}H_{23}NNaO_4S$ requires 348.1240.

***tert*-butyl 3-(3-oxopentyl)azetidine-1-carboxylate (8)**

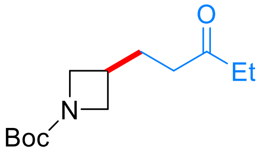

Following **GP1**, pent-1-en-3-one (50.5 mg, 0.6 mmol) gave **8** (50.7 mg, 70%) as an oil. ^{1}H NMR (400 MHz, $CDCl_3$) δ 3.98 (2H, t, J = 8.2 Hz), 3.52 (2H, dd, J = 8.6, 5.4 Hz), 2.51–2.34 (5H, m), 1.85 (2H, q, J = 7.6 Hz), 1.43 (9H, s), 1.05 (3H, t, J = 7.2 Hz); ^{13}C NMR (101 MHz, $CDCl_3$) δ 210.6, 156.3, 79.2, 53.8, 39.3, 36.0, 28.4, 28.3, 28.2; HRMS (ESI-FT): Found M+Na$^+$ 264.1572, $C_{13}H_{23}NNaO_3$ requires 264.1570.

***tert*-butyl 3-(2-(diethoxyphosphoryl)ethyl)azetidine-1-carboxylate (9)**

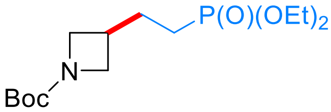

Following **GP1**, diethyl vinylphosphonate (98.5 mg, 0.6 mmol) gave **9** (55.9 mg, 58%) as an oil. ^{1}H NMR (400 MHz, $CDCl_3$) δ 4.14–4.05 (4H, m), 4.00 (2H, t, J = 8.2 Hz), 3.53 (2H, t, J = 7.0 Hz), 2.60–2.50 (1H, m), 1.92–1.84 (2H, m), 1.70–1.59 (2H, m), 1.43 (9H, s), 1.32 (6H, t, J = 7.0 Hz); ^{13}C NMR (101 MHz, $CDCl_3$) δ 156.3, 79.3, 61.6 (d, J = 6.6 Hz), 53.9, 29.2 (d, J = 17.0 Hz), 28.4, 27.1 (d, J = 5.1 Hz), 23.2 (d, J = 142.8 Hz), 16.5 (d, J = 6.1 Hz); ^{31}P NMR (162 MHz, $CDCl_3$) δ 31.1 Data in accordance with the literature.[1]

***tert*-butyl 3-(3-(dimethylamino)-3-oxopropyl)azetidine-1-carboxylate (10)**

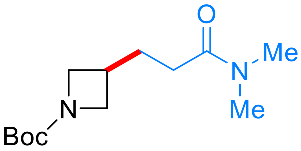

Following **GP1**, *N*,*N*-dimethylacrylamide (59.5 mg, 0.6 mmol) gave **10** (42.3 mg, 55%) as an oil. ^{1}H NMR (400 MHz, $CDCl_3$) δ 4.01 (2H, t, J = 8.2 Hz), 3.56 (2H, dd, J = 8.4, 5.2 Hz), 3.00 (3H, s), 2.95 (3H, s), 2.61 – 2.51 (1H, m), 2.26 (2H, t, J = 7.4 Hz), 1.93 (2H, q, J = 7.6 Hz), 1.44 (9H, s); ^{13}C NMR (101 MHz, $CDCl_3$) δ 172.0, 156.4, 79.2, 54.4, 37.2, 35.4, 30.5, 29.6, 28.4; HRMS (ESI-FT): Found M+Na$^+$ 279.1680, $C_{13}H_{24}N_2NaO_3$ requires 279.1679.

***tert*-butyl 3-(2-(pyridin-2-yl)ethyl)azetidine-1-carboxylate (11)**

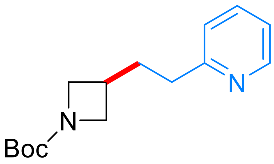

Following **GP1**, 2-vinylpyridine (63.1 mg, 0.6 mmol) gave **11** (51.2 mg, 65%) as an

oil. ^{1}H NMR (400 MHz, $CDCl_3$) δ 8.52–8.50 (1H, m), 7.58 (1H, td, *J* = 7.8, 1.7 Hz), 7.12–7.09 (2H, m), 3.97 (2H, t, *J* = 8.3 Hz), 3.54 (2H, dd, *J* = 8.4, 5.6 Hz), 2.73 (2H, t, *J* = 7.6 Hz), 2.57–2.47(1H, m), 2.02 (2H, q, *J* = 7.7 Hz), 1.42 (9H, s); ^{13}C NMR (101 MHz, $CDCl_3$) δ 161.1, 156.3, 149.3, 136.4, 122.7, 121.2, 79.1, 54.1, 35.7, 34.3, 28.5, 28.4; Data in accordance with the literature.[1]

***tert*-butyl 3-(3-oxo-3-(2,2,2-trifluoroethoxy)propyl)azetidine-1-carboxylate (12)**

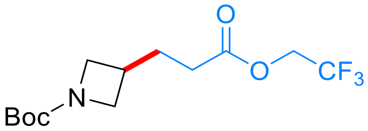

Following **GP1**, 2,2,2-trifluoroethyl acrylate (92.5 mg, 0.6 mmol) gave **12** (62.6 mg, 67%) as an oil. ^{1}H NMR (400 MHz, $CDCl_3$) δ 4.47 (2H, q, *J* = 8.4 Hz), 4.02 (2H, t, *J* = 8.4 Hz), 3.56 (2H, dd, *J* = 8.6, 5.4 Hz), 2.58–2.48 (1H, m), 2.39 (2H, t, *J* = 7.6 Hz), 1.96 (2H, q, *J* = 7.6 Hz), 1.44 (9H, s); ^{13}C NMR (101 MHz, $CDCl_3$) δ 171.3, 122.9 (q, *J* = 278.1 Hz), 79.4, 60.3 (q, *J* = 36.7 Hz), 54.0, 31.1, 29.1, 28.4, 28.1; ^{19}F NMR: (376 MHz, $CDCl_3$) δ -73.8; HRMS (ESI-FT): Found M+Na^{+} 334.1237, $C_{13}H_{20}F_3NNaO_4$ requires 334.1237.

***tert*-butyl 3-(3-(2-chloroethoxy)-3-oxopropyl)azetidine-1-carboxylate (13)**

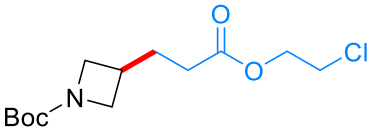

Following **GP1**, 2-chloroethyl acrylate (80.7 mg, 0.6 mmol) gave **13** (84.0 mg, 96%) as an oil. ^{1}H NMR (400 MHz, $CDCl_3$) δ 4.34 (2H, t, *J* = 5.6 Hz), 4.01 (2H, t, *J* = 8.4 Hz), 3.69 (2H, t, *J* = 5.6 Hz), 3.55 (2H, dd, *J* = 8.4, 5.6 Hz), 2.58–2.48 (1H, m), 2.33 (2H, t, *J* = 7.6 Hz), 1.93 (2H, q, *J* = 7.6 Hz), 1.43 (9H, s); ^{13}C NMR (101 MHz, $CDCl_3$) δ 172.5, 156.3, 79.3, 64.1, 54.0, 41.6, 31.5, 29.3, 28.4, 28.2; HRMS (ESI-FT): Found M+Na^{+} 314.1133, $C_{13}H_{22}ClNNaO_4$ requires 314.1130.

***tert*-butyl 3-(3-(2-bromoethoxy)-3-oxopropyl)azetidine-1-carboxylate (14)**

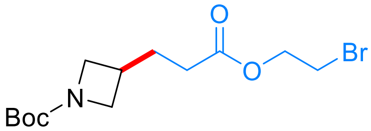

Following **GP1**, 2-bromoethyl acrylate (107.4 mg, 0.6 mmol) gave **14** (76.7 mg, 76%) as an oil. ^{1}H NMR (400 MHz, $CDCl_3$) δ 4.33 (2H, t, *J* = 6.0 Hz), 3.94 (2H, t, *J* = 8.4 Hz), 3.48 (2H, dd, *J* = 8.8, 5.6 Hz), 3.45 (2H, t, *J* = 6.0 Hz), 2.52–2.41 (1H, m), 2.26 (2H, t, *J* = 7.6 Hz), 1.87 (2H, q, *J* = 7.6 Hz), 1.36 (9H, s); ^{13}C NMR (101 MHz, $CDCl_3$) δ 172.5, 156.4, 79.4, 63.9, 54.2, 31.5 29.4, 28.7, 28.4, 28.2; HRMS (ESI-FT): Found M+Na^{+} 358.0628, $C_{13}H_{22}BrNNaO_4$ requires 358.0624.

***tert*-butyl 3-(3-(oxiran-2-ylmethoxy)-3-oxopropyl)azetidine-1-carboxylate (15)**

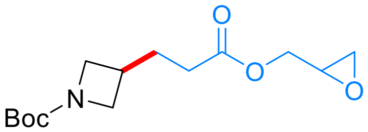

Following **GP1**, oxiran-2-ylmethyl acrylate (76.9 mg, 0.6 mmol) gave **15** (68.5 mg, 80%) as an oil. ^{1}H NMR (400 MHz, $CDCl_3$) δ 4.37 (1H, dd, *J* = 12.2, 3.0 Hz), 3.94 (2H, t, *J* = 8.4 Hz), 3.83 (1H, dd, *J* = 12.2, 6.6 Hz), 3.48 (2H, dd, *J* = 8.6, 5.4 Hz), 3.16–3.12 (1H, m), 2.79 (1H, t, *J* = 4.6 Hz), 2.58 (1H, dd, *J* = 4.8, 2.8 Hz), 2.51–2.40 (1H, m), 2.26 (2H, t, *J* = 7.6 Hz), 1.86 (2H, q, *J* = 7.6 Hz), 1.36 (9H, s); ^{13}C NMR (101 MHz, $CDCl_3$) δ 172.6, 156.4, 79.4, 65.1, 54.5, 49.3, 44.7, 31.5, 29.4, 28.4, 28.2; HRMS (ESI-FT): Found M+Na$^+$ 308.1471, $C_{14}H_{23}NNaO_5$ requires 308.1468.

***tert*-butyl 3-(3-methoxy-2-methyl-3-oxopropyl)azetidine-1-carboxylate (16)**

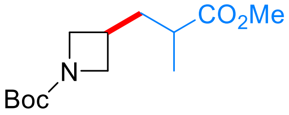

Following **GP1**, methyl methacrylate (60.1 mg, 0.6 mmol) gave **16** (73.3 mg, 95%) as an oil. ^{1}H NMR (400 MHz, $CDCl_3$) δ 4.00 (1H, t, *J* = 8.4 Hz), 3.97 (1H, t, *J* = 8.4 Hz), 3.67 (3H, s), 3.53 (1H, t, *J* = 6.0 Hz), 3.51 (1H, t, *J* = 6.0 Hz), 2.59–2.48 (1H, m), 2.45–2.36 (1H, m), 1.96 (1H, dt, *J* =13.6, 7.6 Hz), 1.70 (1H, ddd, *J* =14.0, 8.4, 6.2 Hz), 1.43 (9H, s), 1.15 (3H, d, *J* = 7.2 Hz); ^{13}C NMR (101 MHz, $CDCl_3$) δ 176.4, 156.3, 79.2, 54.5, 51.7, 38.4, 37.7, 28.4, 27.1, 17.1; Data in accordance with the literature.[1]

***tert*-butyl 3-(4-methoxy-4-oxobutan-2-yl)azetidine-1-carboxylate (17)**

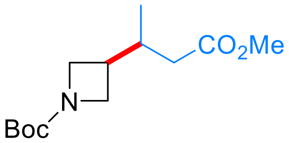

Following **GP1**, methyl (*E*)-but-2-enoate (60.1 mg, 0.6 mmol) gave **17** (40.1 mg, 52%) as an oil. ^{1}H NMR (400 MHz, $CDCl_3$) δ 3.94 (1H, t, *J* = 8.4 Hz), 3.93 (1H, t, *J* = 8.4 Hz), 3.67 (3H, s), 3.63–3.59 (2H,m), 2.39–2.31 (1H, m), 2.29–2.25 (1H, m), 2.16–2.11 (1H, m), 2.09–2.04 (1H, m), 1.43 (9H, s), 0.92 (3H, d, *J* = 6.4 Hz); ^{13}C NMR (101 MHz, $CDCl_3$) δ 172.8, 156.3, 79.3, 52.5, 51.6, 38.7, 34.4, 34.3, 28.4, 16.6; Data in accordance with the literature.[1]

Dimethyl 2-(1-(*tert*-butoxycarbonyl)azetidin-3-yl)succinate (18)

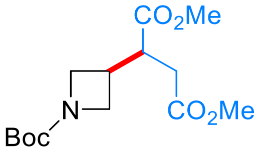

Following **GP1**, dimethyl fumarate (86.5 mg, 0.6 mmol) gave **18** (36.2 mg, 40%) as an oil. ^{1}H NMR (400 MHz, $CDCl_3$) δ 3.93 (2H, q, *J* = 8.9 Hz), 3.72 (1H, dd, *J* = 8.8, 6.0 Hz), 3.64 (3H, s), 3.62 (3H, s), 3.58 (1H, dd, *J* = 8.4, 6.0 Hz), 2.96 (1H, td, *J* = 9.8, 4.0 Hz), 2.73–2.65 (1H, m), 2.59 (1H, dd, *J* = 16.8, 9.2 Hz), 2.37 (1H, dd, *J* = 16.6, 4.2 Hz), 1.36 (9H, s); ^{13}C NMR (101 MHz, $CDCl_3$) δ 173.3, 171.7, 156.2, 79.6, 53.0, 52.2, 52.0, 44.5, 33.5, 30.3, 28.4; HRMS (ESI-FT): Found M+Na$^+$ 324.1420, $C_{14}H_{23}NNaO_6$ requires 324.1418.

***tert*-butyl 3-((2-oxotetrahydrofuran-3-yl)methyl)azetidine-1-carboxylate (19)**

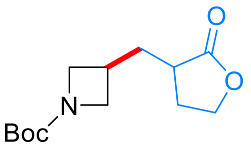

Following **GP1**, 3-methylenedihydrofuran-2(3H)-one (58.9 mg, 0.6 mmol) gave **19** (74.3 mg, 97%) as an oil. ^{1}H NMR (400 MHz, $CDCl_3$) δ 4.34 (1H, t, *J* = 8.8 Hz), 4.21 – 4.14 (1H, m), 4.05 – 4.00 (2H, m), 3.59 – 3.56 (2H, m), 2.74 – 2.63 (1H, m), 2.49 – 2.33 (2H, m), 2.19 – 2.12 (1H, m), 1.97 – 1.67 (1H, m), 1.79 – 1.71 (1H, m), 1.42 (9H, s); ^{13}C NMR (101 MHz, $CDCl_3$) δ 178.6, 156.3, 79.4, 66.5, 54.1, 37.6, 34.8, 28.9, 28.4, 27.1; HRMS (ESI-FT): Found M+Na^{+} 278.1364, $C_{13}H_{21}NNaO_4$ requires 278.1363.

Benzyl (2*R*)-4-((1-(*tert*-butoxycarbonyl)azetidin-3-yl)methyl)-2-(*tert*-butyl)-5-oxo oxazolidine-3-carboxylate (20)

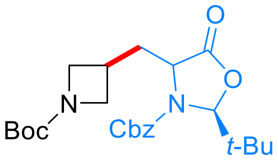

Following **GP1**, benzyl (S)-2-(*tert*-butyl)-4-methylene-5-oxooxazolidine- 3-carboxylate (173.6 mg, 0.6 mmol) gave **20** (101.9 mg, 76%) as an oil. ^{1}H NMR (400 MHz, $CDCl_3$) δ 7.34–7.27 (5H, m), 5.48 (1H, s), 5.14 (1H, d, *J* = 12.0 Hz), 5.06 (1H, d, *J* = 12.0 Hz), 4.12 (1H, t, *J* = 6.0 Hz), 3.92 (2H, t, *J* = 8.0 Hz), 3.53–3.47 (2H, m), 2.76 (1H, br), 2.17–2.10 (1H, m), 2.06–1.97 (1H, m), 1.36 (9H, s), 0.89 (9H, s); ^{13}C NMR (101 MHz, $CDCl_3$) δ 172.2, 156.3, 156.0, 135.0, 128.9, 128.8, 128.7, 96.6, 79.3, 68.7, 55.8, 54.0, 37.5, 36.9, 28.4, 26.9, 26.2, 24.9; HRMS (ESI-FT): Found M+Na^{+} 469.2317, $C_{24}H_{34}N_2NaO_6$ requires 469.2309.

***tert*-butyl 3-(3-oxocyclopentyl)azetidine-1-carboxylate (21)**

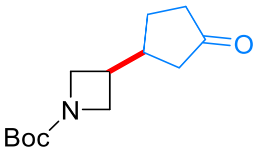

Following **GP1**, cyclopent-2-en-1-one (49.3 mg, 0.6 mmol) gave **21** (30.2 mg, 42%) as an oil. ^{1}H NMR (400 MHz, $CDCl_3$) δ 4.03 (2H, q, *J* = 8.8 Hz), 3.68 (1H, dd, *J* = 8.6, 5.0 Hz), 3.61 (1H, dd, *J* = 8.6, 4.6 Hz), 2.54–2.41 (2H, m), 2.38–2.13 (3H, m), 1.86–1.27 (12H, m); ^{13}C NMR (101 MHz, $CDCl_3$) δ 218.3, 156.3, 79.5, 52.4, 42.2, 40.7, 38.1, 33.1, 31.0, 28.4, 26.5; Data in accordance with the literature.[1]

***tert*-butyl 3-(3-oxocyclohexyl)azetidine-1-carboxylate (22)**

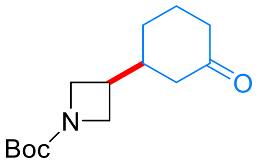

Following **GP1**, cyclohex-2-en-1-one (57.7 mg, 0.6 mmol) gave **22** (35.7 mg, 47%) as an oil. ^{1}H NMR (400 MHz, $CDCl_3$) δ 4.06–3.94 (2H, m), 3.68–3.60 (2H, m), 2.43–2.34 (2.63H, m), 2.32–2.24 (1H, m), 2.12–1.86 (3.63H, m), 1.76–1.60 (1.45H, m), 1.44 (9H, s), 1.35–1.25 (1.31H, m); ^{13}C NMR (101 MHz, $CDCl_3$) δ 210.3, 156.3,

79.4, 52.4, 44.9, 42.8, 41.2, 34.0, 31.0, 28.4, 28.1, 24.9; HRMS (ESI-FT): Found M+Na$^+$ 276.1573, $C_{14}H_{23}NNaO_3$ requires 276.1570.

***tert*-Butyl 3-(2-(Bis(*tert*-butoxycarbonyl)amino)-3-methoxy-3-oxopropyl)azetidine-1-carboxylate (23)**

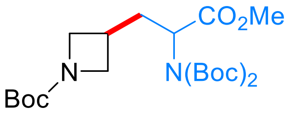

Following **GP2**, *tert*-butyl 3-iodoazetidine-1-carboxylate (84.9 mg, 0.3 mmol) gave **23** (119.7 mg, 87%) as an oil. ^{1}H NMR (400 MHz, $CDCl_3$) δ 4.80 (1H, dd, *J* = 9.6, 4.8 Hz), 3.96 (2H, q, *J* = 8.4 Hz), 3.69 (3H, s), 3.59–3.53 (2H, m), 2.56–2.51 (1H, m), 2.39–2.32 (1H, m), 2.22–2.14 (1H, m), 1.48 (18H, s), 1.40 (9H, s); ^{13}C NMR (101 MHz, $CDCl_3$) δ 170.8, 156.2, 151.9, 83.4, 79.2, 56.4, 54.3, 52.3, 34.4, 28.4, 28.0, 26.3; Data in accordance with the literature.[1]

***tert*-Butyl 3-(2-(Bis(*tert*-butoxycarbonyl)amino)-3-methoxy-3-oxopropyl)pyrrolidine-1-carboxylate (24)**

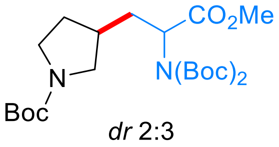

Following **GP2**, *tert*-butyl 3-iodopyrrolidine-1-carboxylate (89.1 mg, 0.3 mmol) gave **24** (120.5 mg, 85%) as an oil. ^{1}H NMR (400 MHz, $CDCl_3$, diastereomers) δ 4.85–4.78 (1H, m), 3.65 (3H, s), 3.53–3.32 (2H, m), 3.24–3.15 (1H, m), 2.91–2.77 (1H, m), 2.14–1.81 (4.44H, m), 1.41 (27.55H, d, *J* = 21.6 Hz); ^{13}C NMR (101 MHz, $CDCl_3$, diastereomers) δ 171.08, 154.52, 154.47, 152.12, 151.96, 151.90, 83.36, 79.09, 78.97, 57.21, 57.12, 56.81, 52.28, 51.54, 51.39, 51.21, 51.02, 45.62, 45.33, 45.18, 36.36, 36.12, 35.37, 35.21, 33.33, 33.24, 33.00, 31.86, 31.27, 31.10, 28.52, 27.97; HRMS (ESI-FT): Found M+Na$^+$ 495.2686, $C_{23}H_{40}N_2NaO_8$ requires 495.2677.

***tert*-Butyl 4-(2-(bis(*tert*-butoxycarbonyl)amino)-3-methoxy-3-oxopropyl) piperidine-1-carboxylate (25)**

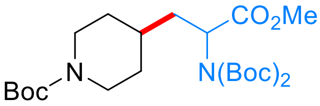

Following **GP2**, *tert*-butyl 4-iodopiperidine-1-carboxylate (93.4 mg, 0.3 mmol) gave **25** (122.6 mg, 84%) as an oil. ^{1}H NMR (400 MHz, $CDCl_3$) δ 4.96 (1H, dd, *J* = 9.4, 5.0 Hz), 4.07 (2H, br), 3.71 (3H, s), 2.66 (2H, dd, *J* = 26.6, 13.0 Hz), 2.06–1.99 (1H, m), 1.86–1.77 (2H, m), 1.65–1.58 (1H, m), 1.50 (18H, s), 1.45 (9H, s), 1.33–1.02 (3H, m); ^{13}C NMR (101 MHz, $CDCl_3$) δ 171.5, 154.8, 152.1, 83.2, 79.3, 55.6, 52.2, 43.9, 36.8, 33.0, 32.5, 31.6, 28.4, 28.0; Data in accordance with the literature.[1]

***tert*-Butyl 6-(2-(bis(*tert*-butoxycarbonyl)amino)-3-methoxy-3-oxopropyl) 2-azaspiro[3.3]heptane-2-carboxylate (26)**

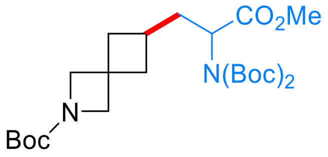

Following **GP2**, *tert*-butyl 6-iodo-2-azaspiro[3.3]heptane-2-carboxylate (97.0 mg, 0.3 mmol) gave **26** (142.1 mg, 95%) as an oil. ^{1}H NMR (400 MHz, $CDCl_3$) δ 4.79 (1H, dd, *J* = 9.6, 4.4 Hz), 3.89 (2H, s), 3.78 (2H, s), 3.69 (3H, s), 2.28–2.22 (2H, m), 2.21–2.09 (2H, m), 2.01–1.93 (1H, m), 1.81–1.77 (2H, m), 1.49 (18H, s), 1.41 (9H, s); ^{13}C NMR (101 MHz, $CDCl_3$) δ 171.2, 156.2, 152.0, 83.1, 79.2, 61.7, 56.7, 52.1, 39.0, 38.9, 36.5, 34.3, 28.4, 28.0, 27.1; HRMS (ESI-FT): Found M+Na$^+$ 521.2845, $C_{25}H_{42}N_2NaO_8$ requires 521.2833.

***tert*-Butyl 2-(2-(bis(*tert*-butoxycarbonyl)amino)-3-methoxy-3-oxopropyl) 7-azaspiro[3.5]nonane-7-carboxylate (27)**

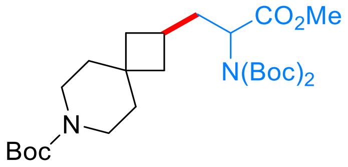

Following **GP2**, *tert*-butyl 2-iodo-7-azaspiro[3.5]nonane-7-carboxylate (105.4 mg, 0.3 mmol) gave **27** (113.8 mg, 72%) as an oil. ^{1}H NMR (400 MHz, $CDCl_3$) δ 4.82 (1H, dd, *J* = 10.0, 4.8 Hz), 3.71 (3H, s), 3.32 (2H, t, *J* = 5.4 Hz), 3.25 (2H, t, *J* = 5.4 Hz), 2.32–2.24 (1H, m), 2.21–2.14 (1H, m), 2.08–2.00 (1H, m), 1.95 (2H, t, *J* = 10.2 Hz), 1.55–1.50 (20H, m), 1.44–1.39 (12H, m), 0.94–0.83 (1H, m); ^{13}C NMR (101 MHz, $CDCl_3$) δ 171.4, 155.0, 152.0, 83.0, 79.2, 56.8, 52.1, 40.4, 39.6, 37.8, 37.6, 36.2, 34.1, 28.5, 28.0, 26.1; HRMS (ESI-FT): Found M+Na$^+$ 549.3158, $C_{27}H_{46}N_2NaO_8$ requires 549.3146.

***tert*-Butyl 4-(2-(Bis(*tert*-butoxycarbonyl)amino)-3-methoxy-3-oxopropyl)azepane -1-carboxylate (28)**

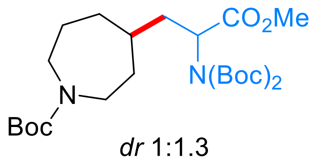

dr 1:1.3

Following **GP2**, *tert*-butyl 4-iodoazepane-1-carboxylate (97.6 mg, 0.3 mmol) gave **28** (139.7 mg, 93%) as an oil. ^{1}H NMR (400 MHz, $CDCl_3$, diastereomers) δ 4.98–4.90 (1H, m), 3.71 (3H, s), 3.67–3.38 (2H, m), 3.36–3.04 (2H, m), 2.10–1.98 (1H, m), 1.90–1.69 (4H, m), 1.50 (18H, s), 1.45–1.38 (10.51H, m), 1.34–0.85 (2.53H, m); ^{13}C NMR (101 MHz, $CDCl_3$, diastereomers) δ 171.65, 171.60, 155.66, 155.53, 152.18, 152.09, 152.05, 83.14, 83.11, 79.04, 78.98, 56.24, 56.19, 56.10, 56.06, 52.21, 46.92, 46.39, 46.33, 45.57, 45.30, 45.11, 45.03, 44.68, 37.56, 37.50, 37.30, 37.18, 36.26, 35.84, 35.64, 35.49, 34.15, 34.13, 33.84, 32.22, 31.67, 28.50, 27.98, 27.02, 26.98, 26.84, 26.78; HRMS (ESI-FT): Found M+Na$^+$ 523.3002, $C_{25}H_{44}N_2NaO_8$ requires 523.2990.

tert-butyl 3-(2-(bis(*tert*-butoxycarbonyl)amino)-3-methoxy-3-oxopropyl)-8-azabicyclo[3.2.1]octane-8-carboxylate (29)

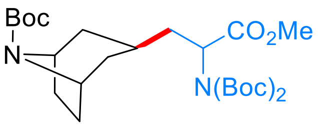

Following **GP2**, *tert*-butyl 3-*exo*-Iodo-8-azabicyclo[3.2.1]octane-8-carboxylate (101.2 mg, 0.3 mmol) gave **29** (104.6 mg, 68%) as an oil. ^{1}H NMR (400 MHz, $CDCl_3$) δ 4.88 (1H, dd, *J* = 9.4, 4.6 Hz), 4.15 (2H, dd, *J* = 36.8 Hz), 3.68 (3H, s), 2.02–1.73 (5H, m), 1.71–1.55 (3H, m), 1.48–1.40 (29H, m), 1.29–1.22 (1H, m); ^{13}C NMR (101 MHz, $CDCl_3$) δ 171.4, 153.3, 152.1, 83.1, 79.0, 55.6, 53.7, 53.0, 52.2, 38.3, 37.6, 37.0, 36.8, 28.5, 28.0, 27.8, 25.4; HRMS (ESI-FT): Found $M+Na^+$ 535.3004, $C_{26}H_{44}N_2NaO_8$ requires 535.2990.

1-(*tert*-butyl)-2-methyl 4-(2-(Bis(*tert*-butoxycarbonyl)amino)-3-methoxy-3-oxopropyl)pyrrolidine-1,2-dicarboxylate (30)

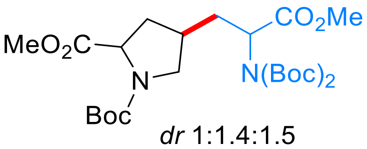

dr 1:1.4:1.5

Following **GP2**, 1-(*tert*-butyl) 2-methyl (2*S*,4*R*)-4-iodopyrrolidine-1,2-dicarboxylate (106.6 mg, 0.3 mmol) gave **30** (136.9 mg, 86%) as an oil. ^{1}H NMR (400 MHz, $CDCl_3$, diastereomers) δ 4.91–4.82 (1H, m), 4.38–4.15 (1H, m), 3.84–3.69 (7H, m), 3.10–2.94 (1H, m), 2.55–2.15 (2.51H, m), 2.14–1.84 (2.54H, m), 1.58–1.40 (27H, m); ^{13}C NMR (101 MHz, $CDCl_3$, diastereomers) δ 173.54, 173.39, 173.30, 173.06, 170.99, 170.91, 170.84, 154.23, 154.14, 154.09, 154.06, 153.54, 153.48, 153.37, 153.33, 152.10, 152.06, 152.01, 151.91, 151.82, 83.45, 83.37, 79.97, 79.95, 79.92, 79.88, 79.86, 79.82, 59.40, 59.32, 59.01, 58.92, 58.86, 58.76, 58.72, 58.51, 57.19, 57.06, 56.96, 56.93, 56.84, 56.72, 56.64, 52.29, 52.14, 52.10, 52.07, 52.02, 51.92, 51.87, 51.79, 51.73, 51.63, 51.53, 36.96, 36.88, 36.60, 36.25, 36.20, 36.17, 35.99, 35.82, 35.75, 35.66, 35.22, 35.10, 34.64, 34.55, 33.88, 33.54, 33.34, 33.31, 33.19, 33.02, 32.86, 32.79, 32.51, 28.38, 28.22, 28.20, 27.93, 27.92, 26.86; HRMS (ESI-FT): Found $M+Na^+$ 553.2744, $C_{25}H_{42}N_2NaO_{10}$ requires 553.2732.

2-(4-(2-(bis(*tert*-butoxycarbonyl)amino)-3-methoxy-3-oxopropyl)piperidin-1-yl)pyrimidine (31)

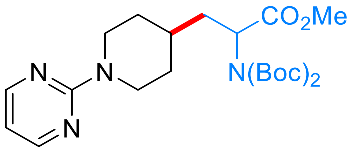

Following **GP2**, 2-(4-iodopiperidin-1-yl)pyrimidine (86.7 mg, 0.3 mmol) gave **31** (76.7 mg, 55%) as an oil. ^{1}H NMR (400 MHz, $CDCl_3$) δ 8.29 (2H, d, *J* = 4.8 Hz), 6.43 (1H, t, *J* = 4.8 Hz), 5.00 (1H, dd, *J* = 9.4, 5.0 Hz), 4.74 (2H, t, *J* = 13.2 Hz), 3.72 (3H, s), 2.90–2.79 (2H, m), 2.09–2.02 (1H, m), 1.92–1.85 (2H, m), 1.73 (1H, d, *J* = 13.2 Hz), 1.60–1.57 (1H, m), 1.51 (18H, s), 1.31–1.15 (2H, m); ^{13}C NMR (101 MHz, $CDCl_3$) δ 171.5, 161.6, 157.7, 152.1, 109.3, 83.2, 55.8, 52.2, 44.0, 43.9, 36.9, 33.3,

32.5, 31.6, 28.0; HRMS (ESI-FT): Found M+Na$^+$ 487.2536, $C_{23}H_{36}N_4NaO_6$ requires 487.2527.

Methyl 2-(Bis(*tert*-butoxycarbonyl)amino)-3-(cyclopentane-4-yl)propanoate (32)

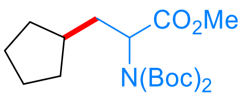

Following **GP2**, iodocyclopentane (58.8 mg, 0.3 mmol) gave **32** (84.7 mg, 76%) as an oil. ^{1}H NMR (400 MHz, $CDCl_3$) δ 4.84 (1H, dd, *J* = 9.6, 4.8 Hz), 3.64 (3H, s), 2.02–1.95 (1H, m), 1.91–1.84 (1H, m), 1.79–1.65 (3H, m), 1.58–1.51 (2H, m), 1.48–1.43 (20H, m), 1.11–1.00 (2H, m); ^{13}C NMR (101 MHz, $CDCl_3$) δ 171.7, 152.1, 82.9, 57.7, 52.1, 36.9, 36.0, 32.9, 32.4, 28.0, 25.2, 25.0; HRMS (ESI-FT): Found M+Na$^+$ 394.2206, $C_{19}H_{33}NNaO_6$ requires 394.2200.

Methyl 2-(Bis(*tert*-butoxycarbonyl)amino)-3-(cyclohexane-4-yl)propanoate (33)

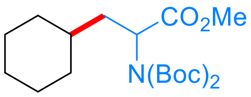

Following **GP2**, iodocyclohexane (63.0 mg, 0.3 mmol) gave **33** (112.2 mg, 97%) as an oil. ^{1}H NMR (400 MHz, $CDCl_3$) δ 4.95 (1H, dd, *J* = 9.6, 4.8 Hz), 3.69 (3H, s), 1.98–1.91 (1H, m), 1.83–1.74 (2H, m), 1.65 (4H, dd, *J* = 21.2, 10.8 Hz), 1.48 (18H, s), 1.26–1.11 (4H, m), 1.01–0.81 (2H, m); ^{13}C NMR (101 MHz, $CDCl_3$) δ 171.8, 152.0, 82.9, 55.9, 52.1, 37.6, 34.4, 33.9, 32.6, 27.9, 26.5, 26.3, 26.1; HRMS (ESI-FT): Found M+Na$^+$ 408.2362, $C_{20}H_{35}NNaO_6$ requires 408.2357.

Methyl 2-(bis(*tert*-butoxycarbonyl)amino)-3-((2*S*,5*R*)-2-isopropyl-5-methyl cyclohexyl)propanoate (34)

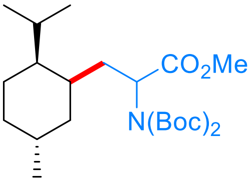

Following **GP2**, (*1R,2R,4S*)-2-iodo-1-isopropyl-4-methylcyclohexane (79.9 mg, 0.3 mmol) gave **34** (90.1 mg, 68%) as an oil. ^{1}H NMR (400 MHz, $CDCl_3$, diastereomers) δ 5.00 (0.23H, dd, *J* = 11.4, 3.4 Hz), 4.93 (0.80H, dd, *J* = 11.8, 5.8 Hz), 3.72 (3H, t, *J* = 3.0 Hz), 2.65–2.59 (0.46H, m), 2.46–2.39 (0.23H, m), 2.25–2.19 (0.25H, m), 2.07–1.79 (1.64H, m), 1.72–1.58 (2.53H, m), 1.56–1.43 (19.47H, m), 1.34–1.23 (1.44H, m), 0.99–0.80 (11.51H, m), 0.71–0.59 (1.52H, m); ^{13}C NMR (101 MHz, $CDCl_3$, diastereomers) δ 172.10, 172.05, 171.71, 152.19, 151.98, 151.88, 82.95, 82.92, 82.88, 57.67, 56.29, 56.04, 52.15, 52.04, 48.80, 47.26, 46.70, 41.63, 41.31, 38.88, 37.19, 35.78, 35.48, 35.23, 35.20, 34.29, 33.13, 32.76, 32.71, 32.57, 29.37, 28.01, 27.98, 27.96, 26.72, 26.69,26.35, 26.07, 24.96, 24.35, 22.74, 22.70, 21.70, 21.65. 21.58, 20.91, 15.24, 15.20; HRMS (ESI-FT): Found M+Na$^+$ 464.2993, $C_{24}H_{43}NNaO_6$ requires 464.2983.

Methyl 2-(Bis(*tert*-butoxycarbonyl)amino)-3-(cycloheptane-4-yl)propanoate (35)

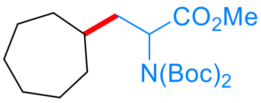

Following **GP2**, iodocycloheptane (67.2 mg, 0.3 mmol) gave **35** (99.5 mg, 83%) as an oil. ^{1}H NMR (400 MHz, $CDCl_3$) δ 4.87 (1H, dd, *J* = 9.6, 4.8 Hz), 3.63 (3H, s), 1.96–1.89 (1H, m), 1.76–1.64 (2H, m), 1.61–1.27 (28H, m), 1.21–1.05 (2H, m); ^{13}C NMR (101 MHz, $CDCl_3$) δ 171.9, 152.1, 82.9, 56.4, 52.1, 38.0, 35.8, 35.5, 33.3, 28.5, 28.2, 28.0, 26.4, 26.2; HRMS (ESI-FT): Found M+Na$^+$ 422.2516, $C_{21}H_{37}NNaO_6$ requires 422.2513.

Methyl 2-(Bis(*tert*-butoxycarbonyl)amino)-3-(cyclododecane-4-yl)propanoate (36)

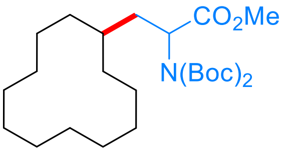

Following **GP2**, iodocyclododecane (88.3 mg, 0.3 mmol) gave **36** (70.5 mg, 50%) as an oil. ^{1}H NMR (400 MHz, $CDCl_3$) δ 4.95 (1H, dd, *J* = 9.6, 4.8 Hz), 3.72 (3H, s), 2.00–1.93 (1H, m), 1.88–1.81 (1H, m), 1.51 (18H, s), 1.45–1.25 (22H, m), 1.20–1.15 (1H, m); ^{13}C NMR (101 MHz, $CDCl_3$) δ 171.98, 152.22, 82.90, 56.21, 52.13, 34.92, 30.57, 29.04, 28.87, 28.00, 24.59, 24.55, 23.96, 23.95, 23.80, 22.82, 22.73, 21.89, 21.36; HRMS (ESI-FT): Found M+Na$^+$ 492.3303, $C_{26}H_{47}NNaO_6$ requires 492.3296.

Methyl 2-(Bis(*tert*-butoxycarbonyl)amino)-3-(tetrahydro-2H-pyran-4-yl) propanoate (37)

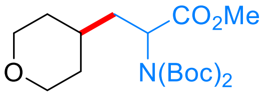

Following **GP2**, 4-iodotetrahydro-2H-pyran (63.6 mg, 0.3 mmol) gave **37** (95.3 mg, 82%) as an oil. ^{1}H NMR (400 MHz, $CDCl_3$) δ 4.96 (1H, dd, *J* = 9.4, 5.0 Hz), 3.95 (2H, dd, *J* = 11.2, 3.6 Hz), 3.72 (3H, s), 3.34–3.30 (2H, m), 2.08–2.01 (1H, m), 1.88–1.81 (1H, m), 1.73 (1H, d, *J* = 13.2 Hz), 1.57–1.47 (20H, m), 1.41–1.25 (2H, m); ^{13}C NMR (101 MHz, $CDCl_3$) δ 171.5, 152.1, 83.2, 67.9, 67.8, 55.4, 52.2, 37.2, 33.4, 32.6, 32.0, 28.0; HRMS (ESI-FT): Found M+Na$^+$ 410.2156, $C_{19}H_{33}NNaO_7$ requires 410.2149.

Methyl 2-(Bis(*tert*-butoxycarbonyl)amino)-3-(tetrahydro-2H-thiopyran-4-yl) propanoate (38)

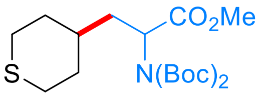

Following **GP2**, 4-iodotetrahydro-2H-thiopyran (68.4 mg, 0.3 mmol) gave **38** (116.2 mg, 96%) as an oil. ^{1}H NMR (400 MHz, $CDCl_3$) δ 4.89 (1H, dd, *J* = 9.4, 5.0 Hz), 3.64 (3H, s), 2.63–2.50 (4H, m), 2.07 (1H, d, *J* = 9.6 Hz), 2.00–1.93 (1H, m), 1.87 (1H, d, *J* = 13.2 Hz), 1.77–1.68 (1H, m), 1.43 (18H, s), 1.38–1.26 (3H, m); ^{13}C NMR (101 MHz, $CDCl_3$) δ 171.5, 152.1, 83.2, 55.3, 52.2, 37.6, 34.7, 34.1, 33.5, 28.7, 28.5, 28.0; HRMS (ESI-FT): Found M+Na$^+$ 426.1926, $C_{19}H_{33}NNaO_6S$ requires 426.1921.

Methyl 2-(Bis(*tert*-butoxycarbonyl)amino)-3-(oxetan-3-yl)propanoate (39)

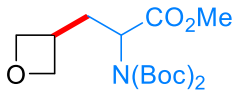

Following **GP2**, 3-iodooxetane (55.2 mg, 0.3 mmol) gave **39** (97.0 mg, 90%) as an oil. ^{1}H NMR (400 MHz, $CDCl_3$) δ 4.80 (1H, dd, J = 9.8, 5.0 Hz), 4.78–4.73 (2H, m), 4.44 (1H, t, J = 6.6 Hz), 4.41 (1H, t, J = 6.6 Hz), 3.72 (3H, s), 3.14–3.03 (1H, m), 2.52–2.45 (1H, m), 2.35–2.27 (1H, m), 1.51 (18H, s); ^{13}C NMR (101 MHz, $CDCl_3$) δ 170.8, 152.0, 83.4, 77.2, 56.5, 52.3, 33.7, 32.7, 28.0; Data in accordance with the literature.[1]

Methyl 2-(Bis(*tert*-butoxycarbonyl)amino)-3-[(diisopropyl cyclobutane-1,1-dicarboxylate)-3-yl]propanoate (40)

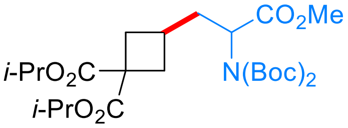

Following **GP2**, diisopropyl 3-iodocyclobutane-1,1-dicarboxylate (106.25 mg, 0.3 mmol) gave **40** (85.80 mg, 54%) as an oil. ^{1}H NMR (400 MHz, $CDCl_3$) δ 5.10–4.97 (2H, m), 4.82 (1H, dd, J = 9.8, 5.0 Hz), 3.70 (3H, s), 2.64–2.56 (2H, m), 2.45–2.33 (1H, m), 2.22–2.16 (3H, m), 2.10–2.02 (1H, m), 1.50 (18H, s), 1.21 (12H, dd, J = 6.0, 3.2 Hz); ^{13}C NMR (101 MHz, $CDCl_3$) δ 171.3, 171.1, 171.0, 151.9, 83.2, 68.6, 56.5, 52.2, 49.7, 36.4, 34.5, 34.4, 28.0, 26.8, 21.5; HRMS (ESI-FT): Found M+Na$^+$ 552.2790, $C_{26}H_{43}NNaO_{10}$ requires 552.2779.

Methyl 2-(Bis(*tert*-butoxycarbonyl)amino)-3-[(1,4-dioxaspiro[4.5]decane)-8-yl] propanoate (41)

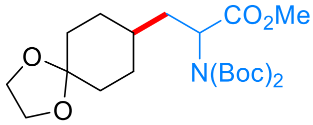

Following **GP2**, 8-iodo-1,4-dioxaspiro[4.5]decane (80.4 mg, 0.3 mmol) gave **41** (122.4 mg, 92%) as an oil. ^{1}H NMR (400 MHz, $CDCl_3$) δ 4.95 (1H, dd, J = 9.4, 5.0 Hz), 3.94 (4H, s), 3.71 (3H, s), 2.06–1.99 (1H, m), 1.87–1.80 (2H, m), 1.78–1.73 (2H, m), 1.56–1.45 (20H, m), 1.43–1.18 (4H, m); ^{13}C NMR (101 MHz, $CDCl_3$) δ 171.6, 152.1, 108.9, 83.1, 64.2, 56.2, 52.2, 36.5, 34.6, 34.3, 33.3, 30.8, 29.5, 28.0; HRMS (ESI-FT): Found M+Na$^+$ 466.2422, $C_{22}H_{37}NNaO_8$ requires 466.2411.

Methyl 2-(Bis(*tert*-butoxycarbonyl)amino)-3-(methoxycyclohexane-2-yl) propanoate (42)

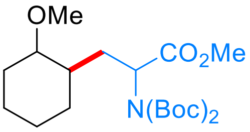

dr 2:1

Following **GP2**, 1-iodo-2-methoxycyclohexane (72.0 mg, 0.3 mmol) gave **42** (86.0 mg, 69%) as an oil. ^{1}H NMR (400 MHz, $CDCl_3$) δ 5.01 (1H, dd, J = 5.6, 3.6 Hz), 3.63 (3H, s), 3.23 (3H, s), 2.75 (1H, td, J = 6.2, 2.6 Hz), 2.43–2.39 (1H, m), 1.99 (1H, dd, J

= 7.6 Hz), 1.68–1.57 (3H, m), 1.55–1.50 (1H, m), 1.46–1.41 (19H, m), 1.16–1.03 (3H, m), 1.00–0.93 (1H, m); ^{13}C NMR (101 MHz, $CDCl_3$) δ 171.8, 152.0, 83.8, 82.8, 57.5, 55.8, 52.1, 39.9, 33.9, 31.4, 30.0, 28.0, 25.3, 24.2; HRMS (ESI-FT): Found M+Na$^+$ 438.2468, $C_{21}H_{37}NNaO_7$ requires 438.2462.

Methyl 2-(Bis(*tert*-butoxycarbonyl)amino)-3-(cyclohexanone-4-yl)propanoate (43)

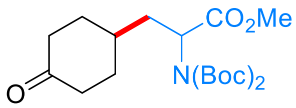

Following **GP2**, 4-iodocyclohexan-1-one (67.2 mg, 0.3 mmol) gave **43** (80.3 mg, 67%) as an oil. ^{1}H NMR (400 MHz, $CDCl_3$) δ 4.91 (1H, dd, J = 9.2, 5.2 Hz), 3.66 (3H, s), 2.34–2.17 (4H, m), 2.15–2.01 (2H, m), 1.98–1.91 (1H, m), 1.87–1.80 (1H, m), 1.76–1.68 (1H, m), 1.44 (18H, s), 1.42–1.28 (2H, m); ^{13}C NMR (101 MHz, $CDCl_3$) δ 211.7, 171.4, 152.2, 83.4, 56.1, 52.3, 40.8, 40.5, 35.9, 33.1, 33.0, 32.0, 28.0; HRMS (ESI-FT): Found M+Na$^+$ 422.2153, $C_{20}H_{33}NNaO_7$ requires 422.2149.

Methyl 2-(Bis(*tert*-butoxycarbonyl)amino)-3-(1-benzoylpiperidin-4-yl) propanoate (44)

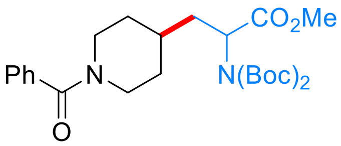

Following **GP2**, (4-iodopiperidin-1-yl)(phenyl)methanone (94.6 mg, 0.3 mmol) gave **44** (139.8 mg, 95%) as an oil. ^{1}H NMR (400 MHz, $CDCl_3$) δ 7.40–7.35 (5H, m), 4.95 (1H, dd, J = 8.2, 5.0 Hz), 4.69 (1H, br), 3.71 (4H, s), 2.93 (1H, s), 2.75 (1H, s), 2.11–2.03 (1H, m), 1.93–1.58 (4H, m), 1.49 (18H, s), 1.33–1.15 (2H, m); ^{13}C NMR (101 MHz, $CDCl_3$) δ 171.4, 170.3, 152.1, 136.3, 129.4, 126.4, 126.8, 83.3, 55.6, 52.3, 47.9, 42.3, 36.7, 33.3, 32.4, 31.4, 28.0; HRMS (ESI-FT): Found M+Na$^+$ 513.2581, $C_{26}H_{38}N_2NaO_7$ requires 513.2571.

Methyl2-(bis(*tert*-butoxycarbonyl)amino)-3-(4-((*tert*-butoxycarbonyl)amino)cyclohexyl)propanoate (45)

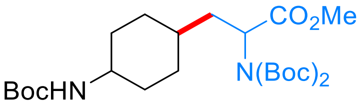

dr 3:2

Following **GP2**, *tert*-butyl (4-iodocyclohexyl)carbamate (97.6 mg, 0.3 mmol) gave **45** (112.6 mg, 75%) as an oil. ^{1}H NMR (400 MHz, $CDCl_3$, diastereomers) δ 4.93 (1H, dd, J = 9.2, 4.8 Hz), 4.63 (0.68 H, br), 4.38 (0.29 H, br), 3.71 (3H, d, J = 1.2 Hz), 2.11–2.04 (0.76H, m), 1.99–1.88 (1H, m), 1.83–1.76 (1.33H, m), 1.67–1.54 (4.51H, m), 1.49 (18H, dd, J = 1.2 Hz), 1.44 (9H, dd, J = 0.8 Hz), 1.28–1.15 (2.24H, m), 1.12–0.94 (1.49H, m), 0.88–0.79 (0.85H, m); ^{13}C NMR (101 MHz, $CDCl_3$, diastereomers) δ 171.62, 171.58, 155.19, 155.13, 152.11, 152.00, 83.08, 79.00, 56.02, 55.99, 52.17, 52.15, 46.44, 37.08, 35.71, 33.74, 33.21, 32.50, 32.37, 31.20, 29.74, 29.50, 28.43, 28.41, 27.96, 27.05, 26.89; HRMS (ESI-FT): Found M+Na$^+$ 523.2998, $C_{25}H_{44}N_2NaO_8$ requires 523.2990.

Methyl 2-(Bis(*tert*-butoxycarbonyl)amino)-3-(octahydro-1H-4,7-methanoindene -4-yl)propanoate (46)

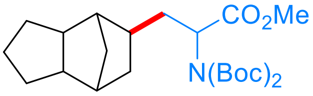

dr 1:1.3

Following **GP2**, 5-iodooctahydro-1H-4,7-methanoindene (78.6 mg, 0.3 mmol) gave **46** (120.8 mg, 92%) as an oil. ^{1}H NMR (400 MHz, $CDCl_3$, diastereomers) δ 4.87 (0.46H, dd, J = 6.4, 3.2 Hz), 4.80 (0.52H, dd, J = 6.2, 3.4 Hz), 3.63 (3H, s), 1.98–1.93 (0.51H, m), 1.89–1.81 (2H, m), 1.77–1.62 (5.58H, m), 1.56–1.52 (1H, m), 1.43 (18H, s), 1.32–1.23 (2H, m), 1.18–1.05 (2H, m), 1.00–0.92 (2H, m), 0.87–0.78 (2H, m); ^{13}C NMR (101 MHz, $CDCl_3$, diastereomers) δ 171.80, 171.14, 152.05, 151.92, 82.93, 82.91, 57.22, 56.90, 52.13, 52.10, 48.70, 48.58, 47.44, 45.94, 44.78, 40.99, 40.96, 38.40, 38.12, 37.14, 36.99, 36.40, 32.29, 32.27, 32.00, 31.96, 29.32, 29.18, 27.98, 27.45; HRMS (ESI-FT): Found M+Na$^+$ 460.2675, $C_{24}H_{39}NNaO_6$ requires 460.2670.

Methyl 2-(Bis(*tert*-butoxycarbonyl)amino)-3-(4-acetamidooctahydropentalen -1-yl)propanoate (47)

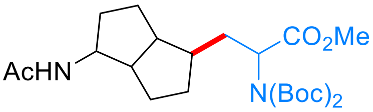

dr 1:2.6:4.1

Following **GP2**, N-(4-iodooctahydropentalen-1-yl)acetamide (88.0 mg, 0.3 mmol) gave **47** (112.5 mg, 80%) as an oil. ^{1}H NMR (400 MHz, $CDCl_3$, diastereomers) δ 6.24–6.09 (0.14H, m), 5.95–5.90 (0.57H, m), 5.86–5.81 (0.37H, m), 4.92–4.85 (1H, m), 4.10–4.04 (0.40H, m), 3.92–3.83 (0.64H, m), 3.71 (3H, m), 2.80–2.59 (0.59H, m), 2.42–2.33 (0.40H, m), 2.24–2.08 (2H, m), 2.00–1.78 (7H, m), 1.74–1.57 (2H, m), 1.50 (18H, m), 1.45–1.28 (2.78H, m), 1.21–1.05 (1.33H, m); ^{13}C NMR (101 MHz, $CDCl_3$, diastereomers) δ 171.72, 171.60, 171.55, 171.49, 169.91, 169.73, 169.72, 152.14, 152.10, 152.02, 152.00, 83.06, 83.05, 60.36, 58.76, 57.64, 57.64, 57.40, 57.37, 57.34, 57.10, 57.01, 53.98, 53.17, 53.11, 52.17, 52.14, 52.12, 50.50, 50.19, 48.89, 48.63, 48.58, 48.28, 48.07, 48.03, 47.34, 46.50, 45.83, 44.77, 44.56, 44.52, 44.26, 44.13, 39.22, 36.49, 35.88, 35.67, 35.32, 34.85, 34.62, 33.53, 33.25, 33.17, 33.00, 32.95, 32.85, 32.56, 31.76, 31.69, 31.01, 30.94, 30.83, 30.81, 30.26, 29.77, 29.58, 29.37, 28.63, 27.96, 27.94, 27.31, 25.68, 23.39, 23.32, 23.25, 23.18, 21.02, 14.16; HRMS (ESI-FT): Found M+Na$^+$ 491.2738, $C_{24}H_{40}N_2NaO_7$ requires 491.2728.

Methyl 2-(Bis(*tert*-butoxycarbonyl)amino)-4-((*1r,3R,5S*)-adamantan-1-yl) pentanoate (48)

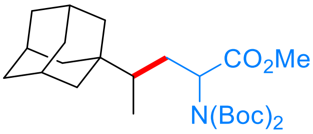

dr 1:3.5

Following **GP2**, 1-(1-iodoethyl)adamantane (87.1 mg, 0.3 mmol) gave **48** (109.0 mg, 78%) as an oil. ^{1}H NMR (400 MHz, $CDCl_3$, diastereomers) δ 4.85 (0.22H, dd, J =

11.6, 3.6 Hz), 4.73 (0.78H, dd, J = 7.8, 5.0 Hz), 3.64 (3H, d, J = 6.4 Hz), 2.52 (0.78H, dd, J = 13.2, 8.4 Hz), 2.19 (0.23H, t, J = 12.8 Hz), 1.89 (3H, s), 1.69–1.35 (30H, m), 1.18–1.10 (1H, m), 1.05–0.98 (0.82H, m), 0.86–0.76 (3.31H, m); ^{13}C NMR (101 MHz, $CDCl_3$, diastereomers) δ 172.14, 171.64, 152.24, 151.95, 82.98, 82.89, 58.07, 56.92, 52.15, 51.98, 40.61, 39.33, 39.15, 37.34, 34.98, 34.45, 32.56, 30.15, 28.67, 28.01, 27.98, 13.48, 12.73; HRMS (ESI-FT): Found M+Na$^+$ 488.2988, $C_{26}H_{43}NNaO_6$ requires 488.2983.

Methyl 2-(Bis(*tert*-butoxycarbonyl)amino)-3-(1-oxaspiro[5.5]undecane-4-yl) propanoate (49)

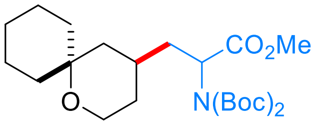

dr 1:1

Following **GP2**, 4-iodo-1-oxaspiro[5.5]undecane (84.1 mg, 0.3 mmol) gave **49** (132.6 mg, 97%) as an oil. ^{1}H NMR (400 MHz, $CDCl_3$, diastereomers) δ 4.99–4.92 (1H, m), 3.71–3.67 (4H, m), 3.57 (1H, dd, J = 27.6, 12.6 Hz), 1.97–1.60 (7H, m), 1.50–1.13 (27H, m), 1.04–0.87 (1H, m); ^{13}C NMR (101 MHz, $CDCl_3$, diastereomers) δ 171.61, 171.58, 152.18, 152.13, 83.09, 72.25, 72.10, 60.39, 60.33, 55.51, 55.18, 52.20, 43.01, 41.92, 40.37, 40.34, 37.48, 37.45, 33.43, 32.56, 29.74, 27.98, 27.32, 27.16, 26.20, 21.74, 21.69, 21.42, 21.39; HRMS (ESI-FT): Found M+Na$^+$ 478.2783, $C_{24}H_{41}NNaO_7$ requires 478.2775.

3-(1-tosylpiperidin-4-yl)propanenitrile (50)

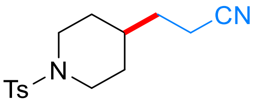

Following **GP1**, 4-iodo-1-oxaspiro[5.5]undecane (109.6 mg, 0.3 mmol), acrylonitrile (31.8 mg, 0.6 mmol) gave **50** (54.39 mg, 62%) as an oil. ^{1}H NMR (400 MHz, $CDCl_3$) δ 7.56 (2H, d, J = 8.0 Hz), 7.26 (2H, d, J = 8.0 Hz), 3.72 (2H, d, J = 12.0 Hz), 2.37 (3H, s), 2.28 (2H, t, J = 7.2 Hz), 2.15 (2H, t, J = 11.4 Hz), 1.68 (2H, d, J = 10.0 Hz), 1.51 (2H, q, J = 6.8 Hz), 1.31–1.18 (3H, m); ^{13}C NMR (101 MHz, $CDCl_3$) δ 143.7, 132.8, 129.7, 127.7, 119.3, 46.2, 34.0, 31.1, 30.8, 21.5, 14.6; HRMS (ESI-FT): Found M+Na$^+$ 315.1137, $C_{15}H_{20}N_2NaO_2S$ requires 315.1138.

Methyl 2-(bis(*tert*-butoxycarbonyl)amino)-6-methylheptanoate (51)

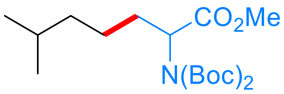

Following **GP2**, 1-iodo-3-methylbutane (59.4 mg, 0.3 mmol) gave **51** (80.7 mg, 72%) as an oil. ^{1}H NMR (400 MHz, $CDCl_3$) δ 4.86 (1H, dd, J = 9.6, 5.2 Hz), 3.71 (3H, s), 2.12–2.03 (1H, m), 1.92–1.82 (1H, m), 1.57–1.50 (19H, m), 1.37–1.30 (2H, m), 1.27–1.15 (2H, m), 0.86 (6H, dd, J = 6.8, 2.8 Hz); ^{13}C NMR (101 MHz, $CDCl_3$) δ 171.5, 152.1, 82.9, 58.1, 52.1, 38.5, 30.1, 28.0, 27.8, 24.0, 22.6, 22.4; Data in accordance with the literature.[1]

Methyl 2-(bis(*tert*-butoxycarbonyl)amino)-undecanoate (52)

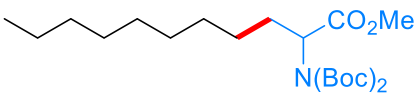

Following **GP2**, 1-iodooctane (72.0 mg, 0.3 mmol) gave **52** (120.9 mg, 97%) as an oil. ^{1}H NMR (400 MHz, $CDCl_3$) δ 4.78 (1H, dd, J = 9.6, 5.2 Hz), 3.64 (3H, s), 2.06–1.97 (1H, m), 1.85–1.76 (1H, m), 1.43 (18H, s), 1.26–1.18 (14H, m), 0.80 (3H, t, J = 6.8 Hz); ^{13}C NMR (101 MHz, $CDCl_3$) δ 171.6, 152.1, 82.9, 58.1, 52.1, 31.9, 29.8, 29.5, 29.4, 29.3, 29.2, 28.0, 26.2, 22.7, 14.1; HRMS (ESI-FT): Found M+Na$^+$ 438.2831, $C_{22}H_{41}NNaO_6$ requires 438.2826.

Methyl 2-(bis(*tert*-butoxycarbonyl)amino)-6-chlorohexanoate (53)

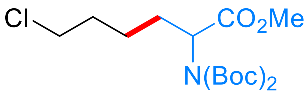

Following **GP2**, 1-chloro-3-iodopropane (61.3 mg, 0.3 mmol) gave **53** (78.6 mg, 69%) as an oil. ^{1}H NMR (400 MHz, $CDCl_3$) δ 4.86 (1H, dd, J = 9.6, 5.2 Hz), 3.72 (3H, s), 3.54 (2H, t, J = 6.6 Hz), 2.18–2.09 (1H, m), 1.97–1.89 (1H, m), 1.87–1.74 (2H, m), 1.53–1.49 (20H, m); ^{13}C NMR (101 MHz, $CDCl_3$) δ 171.2, 152.1, 83.1, 57.8, 52.2, 44.7, 32.1, 29.1, 28.0, 23.5; Data in accordance with the literature.[1]

Methyl 2-(bis(*tert*-butoxycarbonyl)amino)oct-7-ynoate (54)

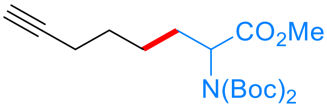

Following **GP2**, 5-iodopent-1-yne (58.2 mg, 0.3 mmol) gave **54** (47.7 mg, 43%) as an oil. ^{1}H NMR (400 MHz, $CDCl_3$) δ 4.87 (1H, dd, J = 9.6, 5.2 Hz), 3.72 (3H, s), 2.20 (2H, td, J = 6.8, 2.4 Hz), 2.17–2.08 (1H, m), 1.96–1.86 (2H, m), 1.62–1.45 (22H, m); ^{13}C NMR (101 MHz, $CDCl_3$) δ 171.3, 152.1, 84.2, 83.1, 68.4, 58.0, 52.1, 29.4, 28.1, 28.0, 25.3, 18.2; Data in accordance with the literature.[1]

Methyl 2-(bis(*tert*-butoxycarbonyl)amino)-oct-7-enoate (55)

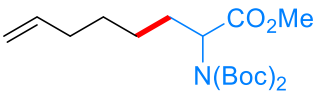

Following **GP2**, 5-iodopent-1-ene (58.8 mg, 0.3 mmol) gave **55** (42.4 mg, 38%) as an oil. ^{1}H NMR (400 MHz, $CDCl_3$) δ 5.77–5.67 (1H, m), 4.94–4.85 (2H, m), 4.78 (1H, dd, J = 9.6, 5.2 Hz), 3.64 (3H, s), 2.08–1.96 (3H, m), 1.87–1.77 (1H, m), 1.43 (18H, s), 1.39–1.25 (4H, m); ^{13}C NMR (101 MHz, $CDCl_3$) δ 171.5, 152.1, 138.7, 114.5, 83.0, 58.1, 52.1, 33.6, 29.7, 28.5, 28.0, 25.7; HRMS (ESI-FT): Found M+Na$^+$ 394.2205, $C_{19}H_{33}NNaO_6$ requires 394.2200.

Methyl 2-(bis(*tert*-butoxycarbonyl)amino)-6-hydroxyhexanoate (56)

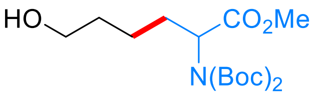

Following **GP2**, 3-iodopropan-1-ol (55.8 mg, 0.3 mmol) gave **56** (96.5 mg, 89%) as an oil. ^{1}H NMR (400 MHz, $CDCl_3$) δ 4.86 (1H, dd, J = 9.4, 5.4 Hz), 3.71 (3H, s), 3.64 (2H, t, J = 5.8 Hz), 2.18–2.09 (1H, m), 1.95–1.85 (1H, m), 1.67–1.55 (3H, m), 1.50

(18H, s), 1.44 (2H, q, J = 7.6 Hz); ^{13}C NMR (101 MHz, $CDCl_3$) δ 171.4, 152.2, 83.1, 62.5, 58.0, 52.2, 32.2, 29.6, 28.0, 22.4; HRMS (ESI-FT): Found M+Na$^+$ 384.1993, $C_{17}H_{31}NNaO_7$ requires 384.1993.

Methyl 2-(bis(*tert*-butoxycarbonyl)amino)-7-((*tert*-butyldimethylsilyl)oxy)heptanoate (57)

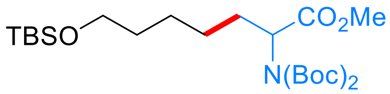

Following **GP2**, *tert*-butyl(4-iodobutoxy)dimethylsilane (94.3 mg, 0.3 mmol) gave **57** (82.3 mg, 56%) as an oil. ^{1}H NMR (400 MHz, $CDCl_3$) δ 4.86 (1H, dd, J = 9.6, 4.8 Hz), 3.71 (3H, s), 3.60 (2H, t, J = 6.6 Hz), 2.15–2.05 (1H, m), 1.93–1.84 (1H, m), 1.56–1.50 (20H, m), 1.41–1.32 (4H, m), 0.89 (9H, t, J = 2.8 Hz), 0.04 (6H, t, J = 3.0 Hz); ^{13}C NMR (101 MHz, $CDCl_3$) δ 171.4, 152.1, 82.9, 63.1, 58.1, 52.1, 32.7, 29.9, 28.0, 26.1, 26.0, 25.5, 18.3, 5.3; HRMS (ESI-FT): Found M+Na$^+$ 512.3024, $C_{24}H_{47}NNaO_7Si$ requires 512.3014.

Methyl 2-(bis(*tert*-butoxycarbonyl)amino)-7-cyanoheptanoate (58)

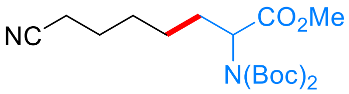

Following **GP2**, 5-iodopentanenitrile (62.7 mg, 0.3 mmol) gave **58** (65.7 mg, 57%) as an oil. ^{1}H NMR (400 MHz, $CDCl_3$) δ 4.77 (1H, dd, J = 9.6, 5.2 Hz), 3.64 (3H, s), 2.27 (2H, t, J = 7.2 Hz), 2.10–2.01 (1H, m), 1.86–1.76 (1H, m), 1.60 (2H, q, J = 7.2 Hz), 1.43 (18H, s), 1.40–1.27 (4H, m); ^{13}C NMR (101 MHz, $CDCl_3$) δ 171.3, 152.2, 119.7, 83.2, 57.8, 52.2, 29.6, 28.3, 28.0, 25.5, 25.2, 17.0; HRMS (ESI-FT): Found M+Na$^+$ 407.2155, $C_{19}H_{32}N_2NaO_6$ requires 407.2153.

Methyl 2-(Bis(*tert*-butoxycarbonyl)amino)-4-(trimethylsilyl)butanoate (59)

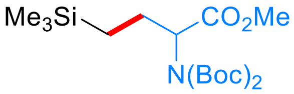

Following **GP2**, (iodomethyl)trimethylsilane (64.2 mg, 0.3 mmol) gave **59** (84.2 mg, 72%) as an oil. ^{1}H NMR (400 MHz, $CDCl_3$) δ 4.81 (1H, dd, J = 9.6, 5.2 Hz), 3.71 (3H, s), 2.13–2.04 (1H, m), 1.93–1.82 (1H, m), 1.50 (18H, s), 0.53–0.44 (2H, m), 0.00 (9H, s); ^{13}C NMR (101 MHz, $CDCl_3$) δ 171.5, 152.2, 82.9, 60.5, 52.0, 28.0, 24.2, 12.8, 1.9; Data in accordance with the literature.[1]

Methyl 2-(bis(*tert*-butoxycarbonyl)amino)-8-oxononanoate (60)

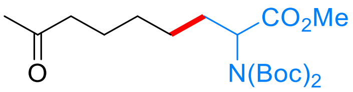

Following **GP2**, 6-iodohexan-2-one (67.8 mg, 0.3 mmol) gave **60** (98.8 mg, 82%) as an oil. ^{1}H NMR (400 MHz, $CDCl_3$) δ 4.77 (1H, dd, J = 6.2, 3.2 Hz), 3.64 (3H, s), 2.35 (2H, t, J = 5.0 Hz), 2.06 (3H, s), 2.04–2.01 (1H, m), 1.82–1.77 (1H, m), 1.52–1.48 (2H, m), 1.42 (18H, s), 1.32–1.22 (4H, m); ^{13}C NMR (101 MHz, $CDCl_3$) δ 209.1, 171.4, 152.1, 83.1, 58.0, 52.1, 43.6, 29.9, 29.7, 28.8, 28.0, 26.0, 23.6; HRMS (ESI-FT): Found M+Na$^+$ 424.2308, $C_{20}H_{35}NNaO_7$ requires 424.2306.

Methyl 2-(bis(*tert*-butoxycarbonyl)amino)-10-ethyl decanedioate (61)

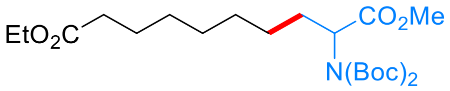

Following **GP2**, ethyl 7-iodoheptanoate (85.2 mg, 0.3 mmol) gave **61** (97.9 mg, 71%) as an oil. ^{1}H NMR (400 MHz, $CDCl_3$) δ 4.84 (1H, q, J = 3.2 Hz), 4.11 (2H, q, J = 4.8 Hz), 3.70 (3H, s), 2.27 (2H, t, J = 5.0 Hz), 2.09–2.06 (1H, m), 1.89–1.83 (1H, m), 1.63–1.57 (2H, m), 1.49 (18H, s), 1.34–1.24 (11H, m); ^{13}C NMR (101 MHz, $CDCl_3$) δ 173.8, 171.5, 152.1, 83.0, 60.2, 58.1, 52.1, 34.3, 29.8, 29.1, 29.0, 28.0, 26.1, 24.9, 14.3; HRMS (ESI-FT): Found M+Na^{+} 482.2731, $C_{23}H_{41}N_2NaO_8$ requires 482.2724.

Methyl 2-(bis(*tert*-butoxycarbonyl)amino)-6,6,7,7,8,8,9,9,10,10,11,11,12,12,13,13,13-heptadecafluorotridecanoate (62)

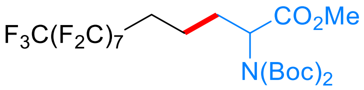

Following **GP2**, 1,1,1,2,2,3,3,4,4,5,5,6,6,7,7,8,8-heptadecafluoro-10-iododecane (172.2 mg, 0.3 mmol) gave **62** (89.9 mg, 40%) as an oil. ^{1}H NMR (400 MHz, $CDCl_3$) δ 4.82 (1H, dd, J = 9.6, 5.2 Hz), 3.65 (3H, s), 2.20–2.05 (2H, m), 2.03–1.90 (2H, m), 1.65–1.57 (2H, m), 1.43 (18H, s); ^{13}C NMR (101 MHz, $CDCl_3$) δ 170.9, 152.1, 121.2, 118.3, 115.6, 112.8, 110.5, 108.4, 83.4, 57.5, 52.3, 30.5 (t, J = 22.22 Hz), 29.3, 27.9, 17.1; ^{19}F NMR: (376 MHz, $CDCl_3$) δ -80.88 (3F, t, J = 9.9 Hz), -114.42 (2F, q, J = 5.64 Hz), -121.81 (2F, s), -122.01 (4F, s), -122.81 (2F, s), -123.56 (2F, s), -126.21 (2F, s); HRMS (ESI-FT): Found M+Na^{+} 772.1552, $C_{24}H_{28}F_{17}NNaO_6$ requires 772.1537.

Dimethyl 2,8-bis(bis(*tert*-butoxycarbonyl)amino)nonanedioate (63)

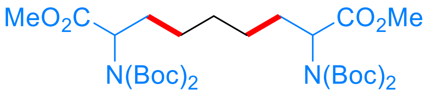

Following **GP2**, 6-iodohexan-2-one (88.8 mg, 0.3 mmol) gave **63** (139.7 mg, 72%) as an oil. ^{1}H NMR (400 MHz, $CDCl_3$) δ 4.77 (2H, dd, J = 9.4, 5.0 Hz), 3.63 (6H, s), 2.06–1.98 (2H, m), 1.84–1.77 (2H, m), 1.42 (36H, s), 1.31–1.25 (6H, m); ^{13}C NMR (101 MHz, $CDCl_3$) δ 171.4, 152.1, 83.0, 58.1, 52.1, 29.9, 29.1, 28.0, 26.2; HRMS (ESI-FT): Found M+Na^{+} 669.3578, $C_{31}H_{54}N_2NaO_{12}$ requires 669.3569.

***tert*-butyl 4-(4-(bis(*tert*-butoxycarbonyl)amino)-5-methoxy-5-oxopentyl) piperidine-1-carboxylate (64)**

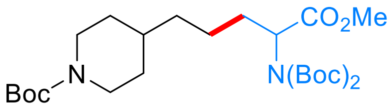

Following **GP2**, tert-butyl 4-(2-iodoethyl)piperidine-1-carboxylate (101.8 mg, 0.3 mmol) gave **64** (92.6 mg, 60%) as an oil. ^{1}H NMR (400 MHz, $CDCl_3$) δ 4.85 (1H, dd, J = 9.6, 5.2 Hz), 4.06 (2H, br), 3.71 (3H, s), 2.65 (2H, t, J = 11.6 Hz), 2.13–2.04 (1H, m), 1.91–1.82 (1H, m), 1.62 (2H, d, J = 13.6 Hz), 1.50 (18H, s), 1.45 (9H, s), 1.39–1.33 (3H, m), 1.31–1.22 (2H, m), 1.10–1.00 (2H, m); ^{13}C NMR (101 MHz, $CDCl_3$) δ 171.4, 154.9, 152.2, 83.0, 79.1, 58.0, 52.1, 44.0, 36.0, 35.7, 32.2, 32.0, 29.9, 28.5, 28.0, 23.2; HRMS (ESI-FT): Found M+Na^{+} 537.3153, $C_{26}H_{46}N_2NaO_8$ requires

537.3146.

Methyl 2-(Bis(*tert*-butoxycarbonyl)amino)-3-(4-methyltetrahydro-2H-pyran-4-yl) propanoate (65)

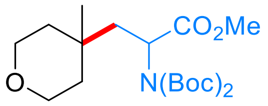

Following **GP2**, 4-iodo-4-methyltetrahydro-2H-pyran (67.8 mg, 0.3 mmol) gave **65** (56.6 mg, 47%) as an oil. ^{1}H NMR (400 MHz, $CDCl_3$) δ 4.99 (1H, dd, *J* = 8.0, 3.2 Hz), 3.77–3.62 (7H, m), 2.35 (1H, dd, *J* = 15.2, 3.2 Hz), 1.83 (1H, q, *J* = 7.6 Hz), 1.57–1.47 (20H, m), 1.41–1.35 (2H, m), 1.05 (3H, s); ^{13}C NMR (101 MHz, $CDCl_3$) δ 172.1, 152.2, 83.2, 63.9, 63.8, 54.7, 52.5, 42.4, 37.7, 37.6, 30.5, 28.0, 23.3; HRMS (ESI-FT): Found M+Na$^+$ 424.2307, $C_{20}H_{35}NNaO_7$ requires 424.2306.

Methyl 2-(Bis(*tert*-butoxycarbonyl)amino)-3-(4-methyltetrahydro-2H-thiopyran-4-yl)propanoate (66)

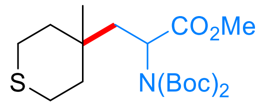

Following **GP2**, 4-iodo-4-methyltetrahydro-2H-pyran (72.6 mg, 0.3 mmol) gave **66** (70.2 mg, 56%) as an oil. ^{1}H NMR (400 MHz, $CDCl_3$) δ 4.95 (1H, dd, *J* = 7.2, 3.2 Hz), 3.72 (3H, s), 2.70–2.53 (4H, m), 2.31 (1H, dd, *J* = 15.2, 3.2 Hz), 1.78–1.63 (5H, m), 1.51 (18H, s), 0.94 (3H, s); ^{13}C NMR (101 MHz, $CDCl_3$) δ 172.0, 152.1, 83.2, 54.4, 52.5, 41.7, 38.3, 38.2, 31.8, 28.0, 24.2, 23.7; HRMS (ESI-FT): Found M+Na$^+$ 440.2084, $C_{20}H_{35}NNaO_6S$ requires 440.2077.

Methyl 2-(Bis(*tert*-butoxycarbonyl)amino)-3-(1-tosyloctahydro-3aH-indol-3a-yl) propanoate (67)

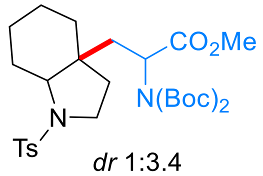

dr 1:3.4

Following **GP2**, 3α-iodo-1-[(4-methylphenyl)sulfonyl]octahydro-1H-indole (121.6 mg, 0.3 mmol) gave **67** (125.4 mg, 72%) as an oil. ^{1}H NMR (400 MHz, $CDCl_3$, diastereomers) δ 7.62 (2H, d, *J* = 8.4 Hz), 7.23 (2H, d, *J* = 8.0 Hz), 4.78 (0.23H, dd, *J* = 9.2, 3.2 Hz), 4.65 (0.78H, dd, *J* = 6.8, 3.6 Hz), 3.59 (3H, d, *J* = 15.2 Hz), 3.45–3.36 (1H, m), 3.33–3.25 (1H, m), 2.89 (1H, t, *J* = 5.0 Hz), 2.35 (3H, d, *J* = 6.8 Hz), 2.12–1.86 (1.71H, m), 1.76–1.47 (5.28H, m), 1.45–1.35 (18H, m), 1.33–1.18 (4H, m), 1.08–0.75 (1H, m); ^{13}C NMR (101 MHz, $CDCl_3$, diastereomers) δ 171.70, 171.50, 152.09, 152.07, 143.19, 143.13, 134.64, 129.65, 129.57, 127.45, 127.33, 83.36, 83.33, 65.37, 65.20, 55.23, 54.92, 52.46, 46.85, 46.13, 43.53, 42.87, 37.06, 35.76, 33.63, 32.26, 29.76, 28.63, 27.97, 27.93, 27.74, 26.90, 21.54, 21.50, 21.31, 21.20, 21.07, 20.77; HRMS (ESI-FT): Found M+Na$^+$ 603.2718, $C_{29}H_{44}N_2NaO_8S$ requires 603.2711.

Methyl 3-(Adamantan-1-yl)-2-(bis(*tert*-butoxycarbonyl)amino)propanoate (68)

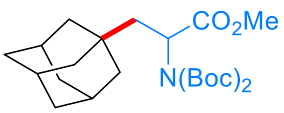

Following **GP2**, 1-iodoadamantane (78.6 mg, 0.3 mmol) gave **68** (111.6 mg, 85%) as an oil. ^{1}H NMR (400 MHz, $CDCl_3$) δ 4.99 (1H, dd, *J* = 7.2, 3.6 Hz), 3.71 (3H, s), 2.13 (1H, dd, *J* = 15.2, 3.6 Hz), 1.96 (3H, s), 1.71 (3H, d, *J* = 12.0 Hz), 1.63 (3H, d, *J* = 10.8Hz), 1.58–1.49 (25H, m); ^{13}C NMR (101 MHz, $CDCl_3$) δ 172.4, 152.0, 82.9, 54.1, 52.3, 44.7, 42.1, 37.0, 32.2, 28.6, 28.0; Data in accordance with the literature.[1]

3-(1-tosyloctahydro-3aH-indol-3a-yl)propanenitrile (69)

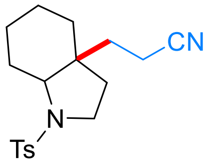

Following **GP2**, 3α-iodo-1-[(4-methylphenyl)sulfonyl]octahydro-1H-indole (121.6 mg, 0.3 mmol) gave **69** (91.8 mg, 92%) as an oil. ^{1}H NMR (400 MHz, $CDCl_3$) δ 7.64 (2H, d, *J* = 5.6 Hz), 7.27 (2H, d, *J* = 5.2 Hz), 3.45–3.41 (1H, m), 3.22 (1H, dd, *J* = 11.6, 5.6 Hz), 2.98 (1H, t, *J* = 4.2 Hz), 2.37 (3H, s), 2.08–1.98 (2H, m), 1.80–1.68 (3H, m), 1.59–1.44 (2H, m), 1.41–1.22 (5H, m), 1.19–1.06 (2H, m); ^{13}C NMR (101 MHz, $CDCl_3$) δ 143.8, 134.9, 129.8, 127.1, 119.6, 63.9, 45.4, 32.8, 31.5, 29.7, 29.1, 22.0, 21.6, 21.1, 12.1; HRMS (ESI-FT): Found M+Na$^+$ 355.1454, $C_{18}H_{24}N_2NaO_2S$ requires 355.1451.

***tert*-butyl 3-(1-tosyloctahydro-3aH-indol-3a-yl)propanoate (70)**

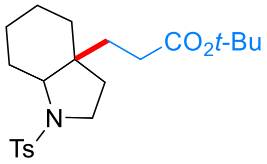

Following **GP2**, 3α-iodo-1-[(4-methylphenyl)sulfonyl]octahydro-1H-indole (121.6 mg, 0.3 mmol) gave **70** (103.9 mg, 85%) as an oil. ^{1}H NMR (400 MHz, $CDCl_3$) δ 7.62 (2H, d, *J* = 4.0 Hz), 7.23 (2H, d, *J* = 4.8 Hz), 3.43–3.38 (1H, m), 3.25–3.21 (1H, m), 2.93 (1H, t, *J* = 3.0 Hz), 2.35 (3H, s), 2.05–1.97 (1H, m), 1.91–1.86 (2H, m), 1.73–1.68 (1H, m), 1.61–1.56 (1H, m), 1.51–1.38 (3H, m), 1.33–1.05 (15H, m); ^{13}C NMR (101 MHz, $CDCl_3$) δ 172.8, 143.2, 134.8, 129.6, 127.3, 80.3, 64.6, 45.9, 43.2, 32.4, 31.6, 30.4, 29.7, 28.4, 28.0, 21.6, 21.3; HRMS (ESI-FT): Found M+Na$^+$ 430.2026, $C_{22}H_{33}NNaO_4S$ requires 430.2023.

***tert*-butyl 3-(3-(2-((*1S*,*5R*)-6,6-dimethylbicyclo[3.1.1]hept-2-en-3-yl)ethoxy)-3-oxopropyl)azetidine-1-carboxylate (71)**

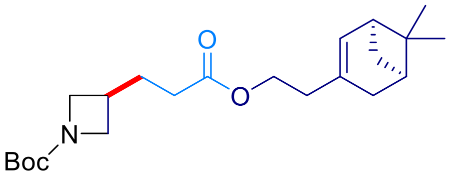

Following **GP1**, 2-((*1R*,*5S*)-6,6-dimethylbicyclo[3.1.1]hept-2-en-3-yl)ethyl acrylate

(132.2 mg, 0.6 mmol) gave **71** (71.4 mg, 63%) as an oil. ^{1}H NMR (400 MHz, $CDCl_3$) δ 5.23–5.21 (1H, m), 4.04–3.98 (2H, m), 3.92 (2H, t, *J* = 8.4 Hz), 3.47 (2H, dd, *J* = 8.6, 5.4 Hz), 2.50–2.40 (1H, m), 2.32–2.27 (1H, m), 2.23–2.13 (6H, m), 2.03–1.95 (2H, m), 1.83 (2H, q, *J* = 7.6 Hz), 1.36 (9H, s), 1.20 (3H, s), 1.06 (1H, d, *J* = 8.4 Hz), 0.75 (3H, s); ^{13}C NMR (101 MHz, $CDCl_3$) δ 172.9, 156.3, 144.0, 118.8, 79.3, 62.8, 54.2, 45.6, 40.7, 38.0, 35.9, 31.7, 31.6, 31.3, 29.5, 28.4, 28.2, 26.3, 21.1; HRMS (ESI-FT): Found M+Na$^+$ 400.2462, $C_{22}H_{35}NNaO_4$ requires 400.2458.

***tert*-butyl (*R*)-3-(3-((3,7-dimethyloct-6-en-1-yl)oxy)-3-oxopropyl)azetidine-1-carboxylate (72)**

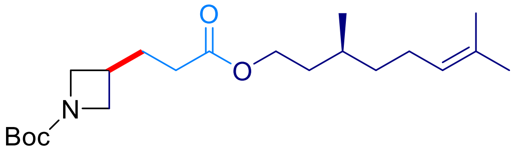

Following **GP1**, (*R*)-3,7-dimethyloct-6-en-1-yl acrylate (126.2 mg, 0.6 mmol) gave **72** (56.2 mg, 51%) as an oil. ^{1}H NMR (400 MHz, $CDCl_3$) δ 5.01 (1H, t, *J* = 7.2 Hz), 4.09–3.98 (2H, m), 3.93 (2H, t, *J* = 8.4 Hz), 3.47 (2H, dd, *J* = 8.8, 5.6 Hz), 2.50–2.39 (1H, m), 2.19 (2H, t, *J* = 7.4 Hz), 1.91–1.81 (3H, m), 1.65–1.57 (4.29H, m), 1.53 (3H, s), 1.49–1.39 (1.77H, m), 1.36 (9H, s), 1.34–1.07 (3H, m), 0.84 (3H, d, *J* = 6.4 Hz); ^{13}C NMR (101 MHz, $CDCl_3$) δ 173.0, 156.4, 131.4, 124.5, 79.3, 63.1, 54.3, 37.0, 35.4, 31.8, 29.5, 29.4, 28.4, 28.3, 25.7, 25.4, 19.4, 17.7; HRMS (ESI-FT): Found M+Na$^+$ 390.2617, $C_{21}H_{37}NNaO_4$ requires 390.2615.

***tert*-butyl 3-(3-((2-(4-methylcyclohex-3-en-1-yl)propan-2-yl)oxy)-3-oxopropyl) azetidine-1-carboxylate (73)**

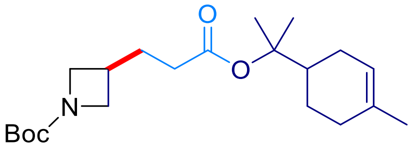

Following **GP1**, 2-(4-methylcyclohex-3-en-1-yl)propan-2-yl acrylate (125.0 mg, 0.6 mmol) gave **73** (34.0 mg, 31%) as an oil. ^{1}H NMR (400 MHz, $CDCl_3$) δ 5.30 (1H, s), 3.92 (2H, t, *J* = 8.4 Hz), 3.47 (2H, dd, *J* = 8.4, 5.6 Hz), 2.48–2.38 (1H, m), 2.11 (2H, t, *J* = 7.6 Hz), 1.99–1.86 (4H, m), 1.82–1.67 (4H, m), 1.59 (4H, t, *J* = 12.4 Hz), 1.36 (15H, t, *J* = 5.6 Hz), 1.27–1.17 (2H, m); ^{13}C NMR (101 MHz, $CDCl_3$) δ 172.2, 156.4, 134.0, 120.2, 85.2, 79.3, 54.2, 42.7, 33.0, 30.9, 29.6, 28.4, 28.3, 26.4, 23.9, 23.4, 23.3, 23.2; HRMS (ESI-FT): Found M+Na$^+$ 388.2462, $C_{21}H_{35}NNaO_4$ requires 388.2458.

***tert*-butyl (*E*)-3-(3-((3,7-dimethylocta-2,6-dien-1-yl)oxy)-3-oxopropyl)azetidine-1-carboxylate (74)**

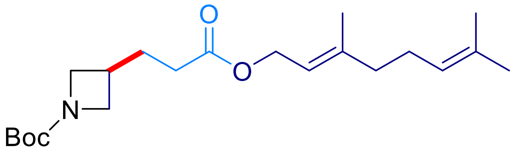

Following **GP1**, (*E*)-3,7-dimethylocta-2,6-dien-1-yl acrylate (125.0 mg, 0.6 mmol) gave **74** (53.7 mg, 49%) as an oil. ^{1}H NMR (400 MHz, $CDCl_3$) δ 5.26 (1H, t, *J* = 4.8 Hz), 5.01 (1H, t, *J* = 4.0 Hz), 4.52 (2H, d, *J* = 4.8 Hz), 3.92 (2H, t, *J* = 5.6 Hz), 3.47 (2H, q, *J* = 4.0 Hz), 2.48–2.41 (1H, m), 2.20 (2H, t, *J* = 5.0 Hz), 2.05–1.96 (4H, m),

1.85 (2H, t, J = 5.2 Hz), 1.62 (6H, t, J = 8.4 Hz), 1.53 (3H, s), 1.36 (9H, s); ^{13}C NMR (101 MHz, $CDCl_3$) δ 173.0, 156.4, 142.5, 131.9, 123.7, 118.1, 79.3, 61.5, 54.6, 39.5, 31.8, 29.5, 28.4, 28.3, 26.3, 25.7, 17.7, 16.5; HRMS (ESI-FT): Found M+Na$^+$ 388.2460, $C_{21}H_{35}NNaO_4$ requires 388.2458.

***tert*-butyl 3-(3-oxo-3-(((*3R,3aS,6R,7R,8aS*)-3,6,8,8-tetramethyloctahydro-1H-3a,7-methanoazulen-6-yl)oxy)propyl)azetidine-1-carboxylate (75)**

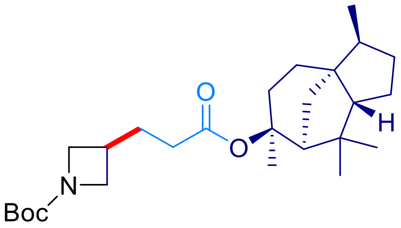

Following **GP1**, (*3R,3aS,6R,7R,8aS*)-3,6,8,8-tetramethyloctahydro-1H-3a,7-methano azulen-6-yl acrylate (165.9 mg, 0.6 mmol) gave **75** (84.6 mg, 65%) as an oil. ^{1}H NMR (400 MHz, $CDCl_3$) δ 3.92 (2H, t, J = 8.4 Hz), 3.47 (2H, dd, J = 8.4, 5.6 Hz), 2.49–2.38 (1H, m), 2.31 (1H, d, J = 5.2 Hz), 2.09 (2H, dd, J = 7.6, 2.4 Hz), 1.97 (1H, dd, J = 13.6, 5.6 Hz), 1.89–1.71 (6H, m), 1.62–1.55 (2H, m), 1.49–1.43 (4H, m), 1.36 (9H, s), 1.33–1.19 (4H, m), 1.07 (3H, s), 0.90 (3H, s), 0.77 (3H, d, J = 7.2 Hz); ^{13}C NMR (101 MHz, $CDCl_3$) δ 172.0, 156.4, 86.7, 79.2, 57.0, 56.6, 54.7, 54.0, 43.4, 41.3, 41.0, 36.9, 33.2, 33.0, 31.2, 29.4, 28.5, 28.4, 28.3, 27.1, 25.9, 25.3, 15.5; HRMS (ESI-FT): Found M+Na$^+$ 456.3091, $C_{26}H_{43}NNaO_4$ requires 456.3084.

***tert*-butyl 3-(3-oxo-3-(((*1R,2R,4S*)-1,3,3-trimethylbicyclo[2.2.1]heptan-2-yl)oxy) propyl)azetidine-1-carboxylate (76)**

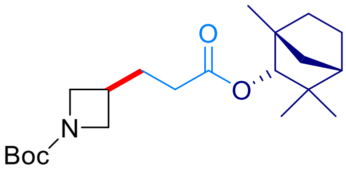

Following **GP1**, (*1R,2R,4S*)-1,3,3-trimethylbicyclo[2.2.1]heptan-2-yl acrylate (125.0 mg, 0.6 mmol) gave **76** (92.1 mg, 84%) as an oil. ^{1}H NMR (400 MHz, $CDCl_3$) δ 4.29 (1H, d, J = 2.0 Hz), 3.93 (2H, td, J = 8.4, 2.0 Hz), 3.48 (2H, q, J = 5.6 Hz), 2.52–2.41 (1.12H, m), 2.23 (1.79H, dd, J = 8.2, 1.6 Hz), 1.86 (2.28H, q, J = 7.6 Hz), 1.65–1.58 (3.15H, m), 1.52–1.49 (1.45H, m), 1.41–1.34 (10.61H, m), 1.13–1.10 (1.15H, m), 1.05–1.02 (3.25H, m), 0.98–0.96 (3.17H, m), 0.71–0.68 (3.15H, m); ^{13}C NMR (101 MHz, $CDCl_3$) δ 173.3, 156.3, 86.3, 79.3, 54.1, 48.3, 48.2, 41.3, 39.4, 31.8, 29.7, 29.6, 28.4, 28.2, 26.6, 25.8, 20.1, 19.4; HRMS (ESI-FT): Found M+Na$^+$ 388.2462, $C_{21}H_{35}NNaO_4$ requires 388.2458.

Methyl 2-(Bis(*tert*-butoxycarbonyl)amino)-5-((*1S,5R*)-6,6-dimethylbicyclo[3.1.1] hept-3-ene-3-yl)pentanoate (77)

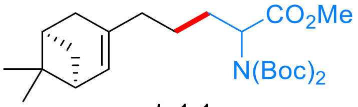

dr 1:1

Following **GP2**, (*1R,5S*)-3-(2-iodoethyl)-6,6-dimethylbicyclo[3.1.1]hept-2-ene (82.9

mg, 0.3 mmol) gave **77** (55.6 mg, 41%) as an oil. ^{1}H NMR (400 MHz, $CDCl_3$, diastereomers) δ 5.12–5.10 (1H, m), 4.79 (1H, q, *J* = 4.8 Hz), 3.64 (3H, s), 2.29–2.24 (1H, m), 2.13 (2H, dd, *J* = 31.2, 17.6 Hz), 2.04–1.95 (2H, m), 1.93–1.75 (4H, m), 1.43 (18H, s), 1.38–1.27 (2H, m), 1.19 (3H, s), 1.06 (1H, dd, *J* = 8.4, 1.6 Hz), 0.74 (3H, d, *J* = 2.0 Hz); ^{13}C NMR (101 MHz, $CDCl_3$, diastereomers) δ 171.49, 152.12, 152.11, 147.91, 147.78, 116.17, 116.04, 82.99, 58.11, 58.05, 52.11, 45.77, 45.63, 40.84, 37.93, 37.91, 36.50, 36.44, 31.66, 31.24, 29.88, 29.68, 28.00, 26.33, 24.01, 23.78, 21.24, 21.18; HRMS (ESI-FT): Found M+Na$^+$ 474.2835, $C_{25}H_{41}NNaO_6$ requires 474.2826.

Methyl 2-(bis(*tert*-butoxycarbonyl)amino) (*S*)-6,10-dimethylundec-9-enoate (78)

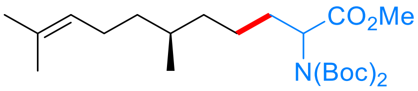

dr 1:1.9

Following **GP2**, (*R*)-8-iodo-2,6-dimethyloct-2-ene (79.9 mg, 0.3 mmol) gave **78** (64.9 mg, 49%) as an oil. ^{1}H NMR (400 MHz, $CDCl_3$, diastereomers) δ 5.10 (0.35H, dd, *J* = 7.0 Hz), 4.96 (0.65H, dd, *J* = 8.0, 2.8 Hz), 4.87 (0.35H, dd, *J* = 9.4, 5.0 Hz), 3.72 (3H, d, *J* = 4.0 Hz), 2.24 (0.66H, dd, *J* = 15.6, 2.8 Hz), 2.13–1.86 (1.46H, m), 1.83–1.69 (3H, m), 1.67–1.61 (1.54H, m), 1.52 (18H, d, *J* = 3.2 Hz), 1.44–1.16 (3.74H, m), 1.14–0.90 (3.23H, m), 0.89–0.85 (7H, m); ^{13}C NMR (101 MHz, $CDCl_3$, diastereomers) δ 172.39, 171.53, 152.20, 152.13, 131.01, 131.00, 124.93, 82.92, 82.89, 55.26, 52.32, 52.08, 45.70, 39.62, 39.55, 35.82, 35.77, 34.86, 32.85, 28.04, 27.98, 27.11, 26.99, 25.71, 25.52, 24.82, 24.62, 22.64, 17.62 ; HRMS (ESI-FT): Found M+Na$^+$ 464.2990, $C_{24}H_{43}NNaO_6$ requires 464.2983.

Methyl 2-(bis(*tert*-butoxycarbonyl)amino)-4-((*3aR,4R,6R,6aR*)-6-methoxy-2,2-dimethyltetrahydrofuro[3,4-d][1,3]dioxol-4-yl)butanoate (79)

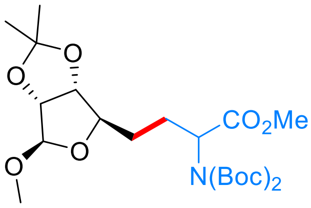

dr 1:1

From D-ribofuranoside

Following **GP2**, (*3aS,4S,6R,6aR*)-4-(iodomethyl)-6-methoxy-2,2-dimethyltetrahydro furo[3,4-d][1,3]dioxole (94.2 mg, 0.3 mmol) gave **79** (142.5 mg, 97%) as an oil. ^{1}H NMR (400 MHz, $CDCl_3$, diastereomers) δ 4.87 (1H, d, *J* = 4.4 Hz), 4.82–4.76 (1H, m), 4.52 (1H, d, *J* = 6.0 Hz), 4.47 (0.49H, d, *J* = 6.0 Hz), 4.42 (0.51H, d, *J* = 6.0 Hz), 4.07 (1H, dd, *J* = 16.2, 8.6 Hz), 3.64 (3H, s), 3.25 (3H, d, *J* = 11.2 Hz), 3.30–2.11 (1H, m), 2.01–1.81 (1H, m), 1.68–1.40 (23H, m), 1.24 (3H, d, *J* = 0.8 Hz); ^{13}C NMR (101 MHz, $CDCl_3$, diastereomers) δ 171.06, 170.98, 151.99, 112.25, 112.21, 109.46, 109.42, 86.85, 86.73, 85.48, 84.12, 84.07, 83.19, 57.95, 57.85, 54.92, 54.87, 52.17, 31.96, 31.93, 27.94, 27.93, 27.13, 27.00, 26.45, 24.96, 24.95; HRMS (ESI-FT): Found M+Na$^+$ 512.2477, $C_{23}H_{39}NNaO_{10}$ requires 512.2466.

Methyl 2-(Bis(*tert*-butoxycarbonyl)amino)-4-((*3aR,6S,6aR*)-6-hydroxy-2,2dimethyltetrahydrofuro[2,3-d][1,3]dioxol-5-yl)butanoate (80)

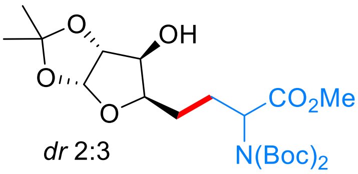

From D-xylofuranose

Following **GP2**, (*3aR,5R,6R,6aR*)-5-(iodomethyl)-2,2-dimethyltetrahydrofuro[2,3-d][1,3]dioxol-6-ol (90.0 mg, 0.3 mmol) gave **80** (118.4 mg, 83%) as an oil. ^{1}H NMR (400 MHz, $CDCl_3$, diastereomers) δ 5.88 (1H, d, *J* = 3.2 Hz), 4.89 (0.43H, dd, *J* = 9.4, 5.4 Hz), 4.83 (0.61H, t, *J* = 6.8 Hz), 4.52 (0.60H, d, *J* = 2.4 Hz), 4.50 (0.40H, d, *J* = 3.6 Hz), 4.13–4.07 (2H, m), 3.71 (3H, s), 2.89 (0.57H, br), 2.35–2.12 (1.46H, m), 2.02–1.49 (24H, m), 1.30 (3H, s); ^{13}C NMR (101 MHz, $CDCl_3$, diastereomers) δ 171.70, 171.14, 152.38, 151.95, 111.33, 111.25, 104.42, 104.34, 85.21, 85.11, 83.41, 83.32, 80.04, 79.92, 74.99, 74.89, 58.11, 57.70, 52.38, 52.23, 27.95, 26.63, 26.60, 26.50, 26.31, 26.13, 26.12, 24.63, 24.23; Data in accordance with the literature.[1]

Methyl 2-(bis(*tert*-butoxycarbonyl)amino)-3-((*3aR,5S,6S,6aR*)-5-((*R*)-2,2-dimethyl-1,3-dioxolan-4-yl)-2,2-dimethyltetrahydrofuro[2,3-d][1,3]dioxol-6-yl)propanoate (81)

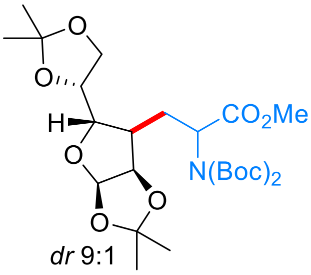

From D-glucofuranose

Following **GP2**, (*3aR,5R,6S,6aS*)-5-((*R*)-2,2-dimethyl-1,3-dioxolan-4-yl)-6-iodo-2,2-dimethyltetrahydrofuro[2,3-d][1,3]dioxole (111.1 mg, 0.3 mmol) gave **81** (98.2 mg, 60%) as an oil. ^{1}H NMR (400 MHz, $CDCl_3$, diastereomers) δ 5.67 (1H, d, *J* = 3.6 Hz), 5.00 (0.10H, d, *J* = 11.4, 4.2 Hz), 4.94 (0.91H, t, *J* = 6.2 Hz), 4.62 (0.09H, t, *J* = 4.2 Hz), 4.48 (0.91H, d, *J* = 3.6 Hz), 4.10–3.99 (2.90H, m), 3.87–3.81 (1.10H, m), 3.66 (3H, d, *J* = 4.0 Hz), 2.60–2.50 (1.88H, m), 2.40–2.22 (0.21H, m), 1.44–1.33 (25H, m), 1.26 (3H, s), 1.23 (3H, s); ^{13}C NMR (101 MHz, $CDCl_3$, diastereomers) δ 170.77, 151.87, 111.28, 109.32, 104.69, 84.05, 83.42, 80.99, 73.33, 68.45, 57.03, 52.29, 44.36, 27.97, 26.76, 26.75, 26.54, 26.11, 25.26; HRMS (ESI-FT): Found $M+Na^+$ 568.2740, $C_{26}H_{43}NNaO_{11}$ requires 568.2728.

Methyl 2-(bis(*tert*-butoxycarbonyl)amino)-4-((*3aR,5R,5aS,8aS,8bR*)-2,2,7,7-*tetra*methyltetrahydro-5H-bis([1,3]dioxolo)[4,5-b:4',5'-d]pyran-5-yl)butanoate (82)

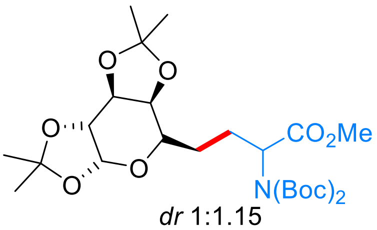

From D-galactopyranose

Following **GP2**, (*3aR,5S,5aR,8aS,8bR*)-5-(iodomethyl)-2,2,7,7-tetramethyltetrahydro-5H-bis([1,3]dioxolo)[4,5-b:4',5'-d]pyran (111.1 mg, 0.3 mmol) gave **82** (134.2 mg, 82%) as an oil. ^{1}H NMR (400 MHz, $CDCl_3$, diastereomers) δ 5.45 (1H, d, *J* = 4.8 Hz), 4.83 (0.53H, dd, *J* = 10.2, 5.0 Hz), 4.80 (0.46H, dd, *J* = 8.4, 4.8 Hz), 4.51 (1H, t, *J* = 7.8, 1.8 Hz), 4.22 (1H, dd, *J* = 5.0, 2.2 Hz), 4.06 (1H, t, *J* = 6.4 Hz), 3.69–3.63 (4H, m), 2.22–2.08 (1H, m), 1.99–1.86 (1H, m), 1.68–1.52 (2H, m), 1.44 (21H, d, *J* = 10.4 Hz), 1.36 (3H, d, *J* = 4.4 Hz), 1.25 (6H, t, *J* = 3.6 Hz); ^{13}C NMR (101 MHz, $CDCl_3$, diastereomers) δ 171.34, 171.24, 152.10, 151.99, 109.00, 108.95, 108.32, 108.30, 96.49, 96.45, 83.03, 83.00, 72.63, 72.19, 70.91, 70.79, 70.47, 67.50, 67.01, 58.25, 57.77, 52.16, 52.13, 27.95, 26.88, 26.83, 26.02, 25.97, 25.94, 24.93, 24.44, 24.40; HRMS (ESI-FT): Found $M+Na^+$ 568.2739, $C_{26}H_{43}NNaO_{11}$ requires 568.2728.

Methyl 2-(bis(*tert*-butoxycarbonyl)amino)-4-((*3aS,5aR,8aR,8bS*)-2,2,7,7-*tetra* methyltetrahydro-3aH-bis([1,3]dioxolo)[4,5-b:4',5'-d]pyran-3a-yl)butanoate(83)

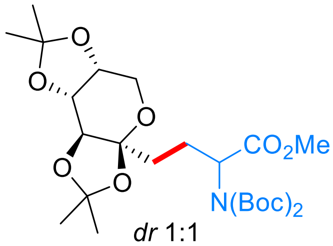

From D-fructopyranose

Following **GP2**, (3aR,5aR,8aR,8bS)-3a-(iodomethyl)-2,2,7,7-tetramethyltetrahydro-5H-bis([1,3]dioxolo)[4,5-b:4',5'-d]pyran (111.1 mg, 0.3 mmol) gave **83** (142.4 mg, 87%) as an oil. ^{1}H NMR (400 MHz, $CDCl_3$, diastereomers) δ 4.80 (1H, dd, *J* = 9.6, 5.6 Hz), 4.48 (1H, dd, *J* = 7.8, 1.8 Hz), 4.13 (1H, d, *J* = 8.0 Hz), 4.04 (0.52H, d, *J* = 2.4 Hz), 3.99 (0.50H, d, *J* = 2.4 Hz), 3.76 (1H, d, *J* = 12.8 Hz), 3.66–3.61 (4H, m), 2.42–2.22 (1H, m), 2.19–2.01 (1H, m), 1.90–1.79 (1H, m), 1.74–1.65 (1H, m), 1.43 (24H, d, *J* = 8.4 Hz), 1.26 (6H, dd, *J* = 6.0, 3.2 Hz); ^{13}C NMR (101 MHz, $CDCl_3$, diastereomers) δ 171.12, 171.10, 151.93, 151.87, 108.94, 108.87, 107.46, 107.35, 103.82, 103.78, 82.97, 73.71, 70.76, 70.68, 70.57, 70.54, 60.97, 60.90, 58.14, 58.07, 52.04, 37.47, 37.30, 27.93, 26.37, 26.36, 25.83, 25.82, 25.05, 24.19, 24.04, 23.85, 23.78; HRMS (ESI-FT): Found $M+Na^+$ 568.2740, $C_{26}H_{43}NNaO_{11}$ requires 568.2728.

4-(4-(2-cyanoethyl)piperidine-1-carbonyl)-N,N-dipropylbenzenesulfonamide (84)

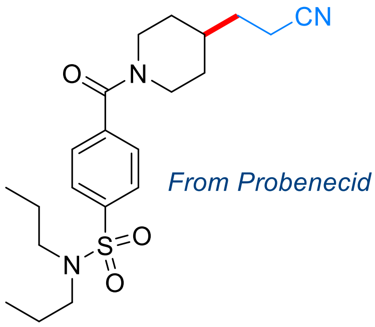

Following **GP2**, 4-(4-iodopiperidine-1-carbonyl)-N,N-dipropylbenzenesulfonamide (143.5 mg, 0.3 mmol) gave **84** (87.6 mg, 72%) as an oil. ^{1}H NMR (400 MHz, $CDCl_3$) δ 7.83 (2H, d, *J* = 8.4 Hz), 7.49 (2H, d, *J* = 8.0 Hz), 4.72 (1H, d, *J* = 9.2 Hz), 3.61 (1H, d, *J* = 10.0 Hz), 3.06 (5H, t, *J* = 7.6 Hz), 2.78 (1H, s), 2.40 (2H, t, *J* = 7.0 Hz), 1.86–1.50 (9H, m), 1.28–1.04 (2H, m), 0.86 (6H, d, *J* = 7.4 Hz); ^{13}C NMR (101 MHz, $CDCl_3$) δ 168.6, 141.2, 139.9, 127.4, 127.3, 119.4, 50.1, 47.6, 42.1, 34.9, 32.1, 31.4, 31.1, 22.1, 14.5, 11.2; HRMS (ESI-FT): Found M+Na$^+$ 428.1987, $C_{21}H_{31}N_3NaO_3S$ requires 428.1978.

(*R*)-3-(iodomethyl)-1-tosylpyrrolidine (86)

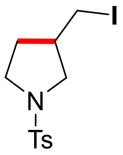

Following **GP**, N-allyl-N-(2-iodoethyl)-4-methylbenzenesulfonamide (109.6 mg, 0.3 mmol) gave **86** (73.4 mg, 67%) as an oil. ^{1}H NMR (400 MHz, $CDCl_3$) δ 7.65 (2H, d, *J* = 8.4 Hz), 7.27 (2H, d, *J* = 8.0 Hz), 3.39 (1H, dd, *J* = 10.2, 7.4 Hz), 3.35–3.29 (1H, m), 3.20–3.14 (1H, m), 3.00–2.87 (3H, m), 2.37 (3H, s), 2.35–2.28 (1H, m), 1.99–1.91 (1H, m), 1.58–1.37 (1H, m); ^{13}C NMR (101 MHz, $CDCl_3$) δ 143.7, 133.4, 129.8, 127.6, 53.7, 47.5, 41.3, 32.1, 21.6, 7.2; HRMS (ESI-FT): Found M+Na$^+$ 387.9842, $C_{12}H_{16}INNaO_2S$ requires 387.9839.

4-(1-tosylpyrrolidin-3-yl)butanenitrile (87a)

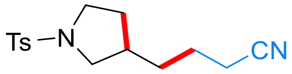

Following **GP1**, N-allyl-N-(2-iodoethyl)-4-methylbenzenesulfonamide (109.6 mg, 0.3 mmol), acrylonitrile (31.8 mg, 0.6 mmol) gave **86** (35.1 mg, 32%) and **87a** (30.7 mg, 35%) as an oil. ^{1}H NMR (400 MHz, $CDCl_3$) δ 7.64 (2H, d, *J* = 8.0 Hz), 7.27 (2H, d, *J* = 8.0 Hz), 3.37 (1H, dd, *J* = 9.6, 7.2 Hz), 3.31–3.25 (1H, m), 3.17–3.13 (1H, m), 2.72 (1H, dd, *J* = 9.6, 8.0 Hz), 2.37 (3H, s), 2.24 (2H, t, *J* = 6.8 Hz), 2.01–1.85 (2H, m), 1.55–1.47 (2H, m), 1.37–1.28 (3H, m); ^{13}C NMR (101 MHz, $CDCl_3$) δ 143.5, 133.6, 129.7, 127.5, 119.2, 52.9, 47.4, 38.1, 32.1, 31.2, 23.9, 21.6, 17.3; HRMS (ESI-FT): Found M+Na$^+$ 315.1137, $C_{15}H_{20}N_2NaO_2S$ requires 315.1138.

***tert*-butyl 4-(1-tosylpyrrolidin-3-yl)butanoate (87b)**

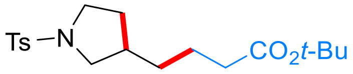

Following **GP1**, N-allyl-N-(2-iodoethyl)-4-methylbenzenesulfonamide (109.6 mg, 0.3

mmol), *tert*-butyl acrylate (76.9 mg, 0.6 mmol) gave **86** (36.2 mg, 33%) and **87b** (49.6 mg, 45%) as an oil. ^{1}H NMR (400 MHz, $CDCl_3$) δ 7.64 (2H, d, *J* = 8.0 Hz), 7.25 (2H, d, *J* = 8.0 Hz), 3.36 (1H, dd, *J* = 9.6, 7.6 Hz), 3.29–3.24 (1H, m), 3.15–3.08 (1H, m), 2.70 (1H, dd, *J* = 9.6, 8.0 Hz), 2.37 (3H, s), 2.08 (2H, t, *J* = 7.4 Hz), 1.96–1.82 (2H, m), 1.49–1.27 (12H, m), 1.20–1.15 (2H, m); ^{13}C NMR (101 MHz, $CDCl_3$) δ 172.7, 143.3, 133.8, 129.6, 127.5, 80.3, 53.2, 47.6, 38.6, 35.3, 32.5, 31.4, 28.1, 23.5, 21.5; HRMS (ESI-FT): Found M+Na^{+} 390.1680, $C_{19}H_{29}NNaO_4S$ requires 390.1710.

Methyl 2-(Bis(*tert*-butoxycarbonyl)amino)-4-(tosylpyrrolidine-4-yl)butanoate (87c)

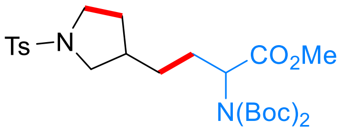

Following **GP2**, N-allyl-N-(2-iodoethyl)-4-methylbenzenesulfonamide (109.6 mg, 0.3 mmol) gave **86** (41.6 mg, 38%) and **87c** (79.5 mg, 49%) as an oil. ^{1}H NMR (400 MHz, $CDCl_3$) δ 7.63 (2H, dd, *J* = 7.8, 3.0 Hz), 7.25 (2H, d, *J* = 7.6 Hz), 4.71–4.67 (1H, m), 3.63 (3H, s), 3.37 (1H, t, *J* = 8.6 Hz), 3.30–3.24 (1H, m), 3.10 (1H, t, *J* = 16.8, 8.8 Hz), 2.72–2.63 (1H, m), 2.36 (3H, s), 2.02–1.84 (3H, m), 1.79–1.70 (1H, m), 1.41 (18H, d, *J* = 2.4 Hz), 1.29–1.18 (3H, m); ^{13}C NMR (101 MHz, $CDCl_3$) δ 171.08, 152.11, 143.37, 133.73, 129.66, 127.51, 83.29, 83.26, 57.80, 57.78, 53.21, 53.15, 52.22, 47.60, 47.58, 38.50, 38.44, 31.44, 31.27, 29.82, 29.67, 28.52, 28.30, 27.96, 21.52; HRMS (ESI-FT): Found M+Na^{+} 563.2410, $C_{26}H_{40}N_2NaO_8S$ requires 563.2398.

Methyl 2-(bis(*tert*-butoxycarbonyl)amino)-4-(cyclopropaneyl)butanoate (89) + Methyl 2-(bis(*tert*-butoxycarbonyl)amino)-hept-6-enoate (90)

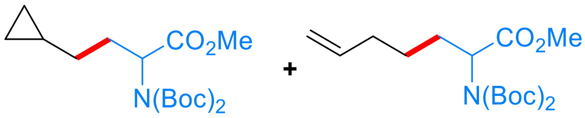

Following **GP2**, (iodomethyl)cyclopropane (54.6 mg, 0.3 mmol) gave **89** (12.9 mg, 12%) and **90** (38.6 mg, 36%) as an oil. ^{1}H NMR (400 MHz, $CDCl_3$) δ 5.77–5.67 (1H, m), 4.97–4.87 (2H, m), 4.80 (1H, q, *J* = 4.8 Hz), 4.75 (0.29H, q, *J* = 4.8 Hz), 3.64 (3H, s), 3.63 (1H, s), 2.31–2.23 (0.29H, m), 2.06–1.92 (4.29H, m), 1.89–1.75 (1.55H, m), 1.61–1.55 (1.12H, m), 1.43–1.33 (26H, m); ^{13}C NMR (101 MHz, $CDCl_3$) δ 171.6, 171.4, 152.1, 152.0, 138.3, 114.9, 83.0, 82.9, 57.9, 56.8, 52.1, 36.8, 33.3, 33.1, 29.7, 29.3, 28.3, 28.2, 28.0, 25.4, 18.5; HRMS (ESI-FT): Found M+Na^{+} 380.2046, $C_{18}H_{31}NNaO_6$ requires 380.2044.

***tert*-butyl 3-(2-cyanoethyl-2-d)azetidine-1-carboxylate (91)**

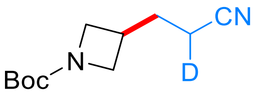

Following **GP1**, acrylonitrile (31.8 mg, 0.6 mmol), D_2O (0.45 mL) gave **91** (56.4 mg, 89%) as an oil. ^{1}H NMR (400 MHz, $CDCl_3$) δ 4.00 (2H, t, *J* = 8.4 Hz), 3.52 (2H, dd, *J* = 8.8, 5.6 Hz), 2.63–2.53 (1H, m), 2.30–2.24 (1H, m), 1.90 (2H, t, *J* = 7.4 Hz), 1.37

(9H, s); ^{13}C NMR (101 MHz, $CDCl_3$) δ 156.2, 118.9, 79.6, 54.0, 29.7, 28.4, 27.9, 15.1, 14.9, 14.7; Data in accordance with the literature.[1]

(*R*)-4-(1-(diethylamino)ethyl)benzonitrile (92)

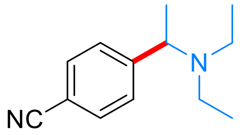

Following **GP1**, using terephthalonitrile (38.4 mg, 0.3 mmol) and Et_3N (91.1 mg, 0.9 mmol), without using alkyl iodides and H_2O (0.45 mL) gave **92** (43.7 mg, 72%) as oil. ^1H NMR (400 MHz, $CDCl_3$) δ 7.62 (2H, d, J = 8.0 Hz), 7.52 (2H, d, J = 8.4 Hz), 3.85 (1H, dd, J = 13.6, 6.8 Hz), 2.59–2.49 (4H, m), 1.34 (3H, d, J = 6.8 Hz), 1.01 (6H, t, J = 7.2 Hz); ^{13}C NMR (101 MHz, $CDCl_3$) δ 151.6, 132.0, 128.2, 119.2, 110.3, 62.4, 59.1, 42.9, 17.7, 12.1; Data in accordance with the literature.[12]

(*R*)-4-(1-(diisopropylamino)ethyl)benzonitrile (93) + 4-(2-(ethyl(isopropyl)amino) propan-2-yl)benzonitrile (94)

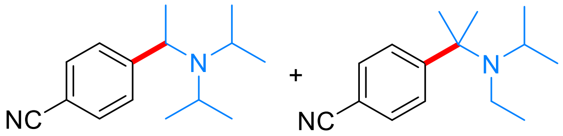

Following **GP1**, using 1,4-dicyanobenzene (38.4 mg, 0.3 mmol) and *i*-Pr_2EtN (116.3 mg, 0.9 mmol), without using alkyl iodides and H_2O (0.45 mL) gave **93** (47.7 mg, 69%) and **94** (11.8 mg, 17%) as oil. ^1H NMR (400 MHz, $CDCl_3$) δ 7.70 (0.33H, d, J = 8.4 Hz), 7.60 (4.29H, d, J = 10.4 Hz), 4.16 (1H, dd, J = 13.6, 6.8 Hz), 3.13–3.03 (2H, m), 2.77–2.63 (0.50H, m), 1.49 (3H, d, J = 6.8 Hz), 1.11 (6.54H, dd, J = 12.8, 6.4 Hz), 0.99 (6.98H, dd, J = 16.8, 6.8 Hz); ^{13}C NMR (101 MHz, $CDCl_3$) δ 157.4, 153.5, 131.8, 131.6, 128.3, 127.1, 119.3, 109.60, 109.56, 62.4, 52.1, 48.4, 45.4, 35.7, 27.1, 23.4, 22.5, 22.4, 20.2, 19.2; Data in accordance with the literature.[12]

***tert*-butyl 3-(4-cyanophenyl)azetidine-1-carboxylate (95)**

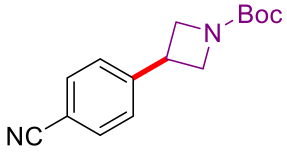

Following **GP1**, using 1,4-dicyanobenzene (38.4 mg, 0.3 mmol) and *tert*-butyl 3-iodoazetidine-1-carboxylate (127.4 mg, 0.45 mmol) gave **4** (32.6 mg, 42%) as an oil. ^1H NMR (400 MHz, $CDCl_3$) δ 7.66 (2H, d, J = 8.0 Hz), 7.45 (2H, d, J = 8.0 Hz), 4.38 (2H, t, J = 8.6 Hz), 3.95 (2H, dd, J = 8.6, 5.8 Hz), 3.82–3.75 (1H, m), 1.47 (9H, s); ^{13}C NMR (101 MHz, $CDCl_3$) δ 156.3, 147.7, 132.6, 127.7, 118.7, 110.9, 79.9, 56.0, 33.5, 28.4; Data in accordance with the literature.[13]

6. NMR Spectra

***tert*-butyl 3-(2-cyanoethyl)azetidine-1-carboxylate (3)-** ^{1}H NMR (400 MHz, $CDCl_3$)

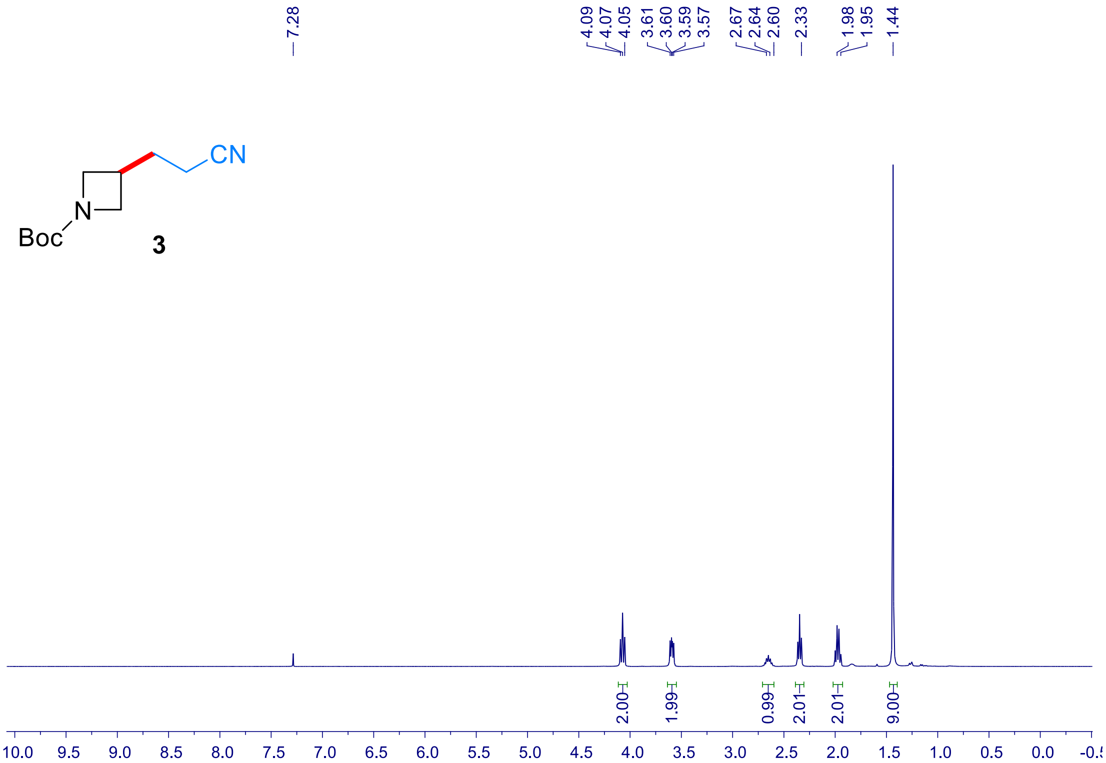

(3)- ^{13}C NMR (101 MHz, $CDCl_3$)

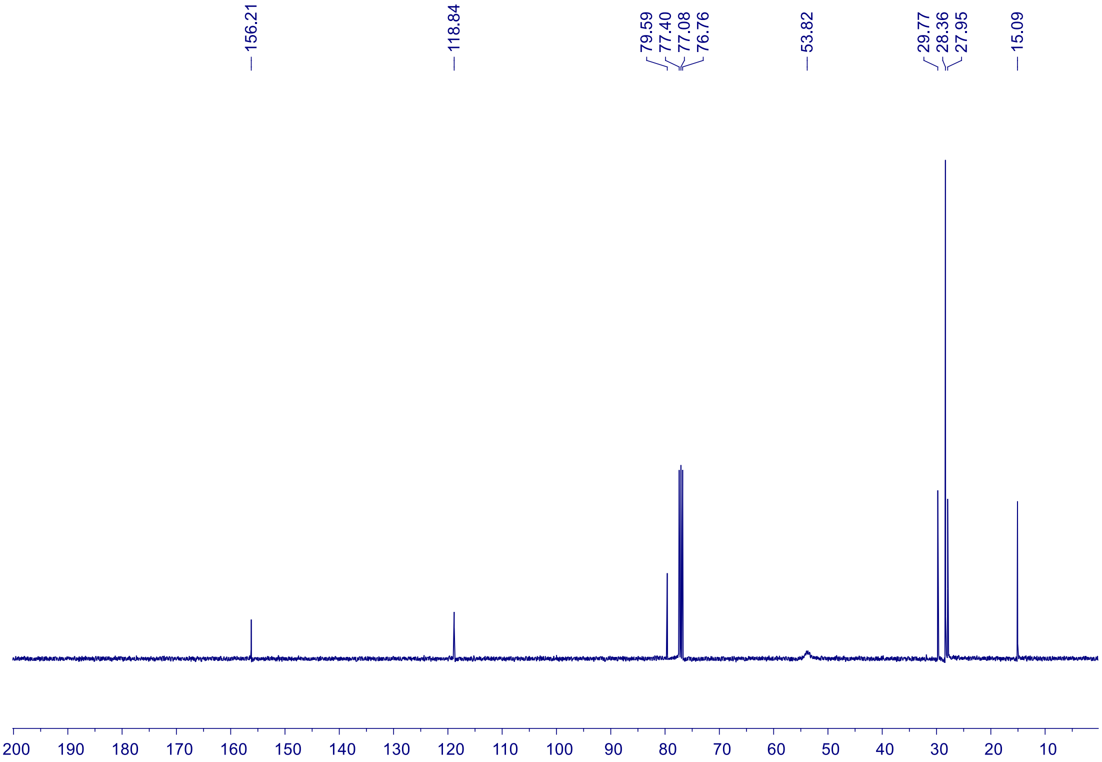

***tert*-butyl 3-(3-methoxy-3-oxopropyl)azetidine-1-carboxylate (4)**-^{1}H NMR (400 MHz, $CDCl_3$)

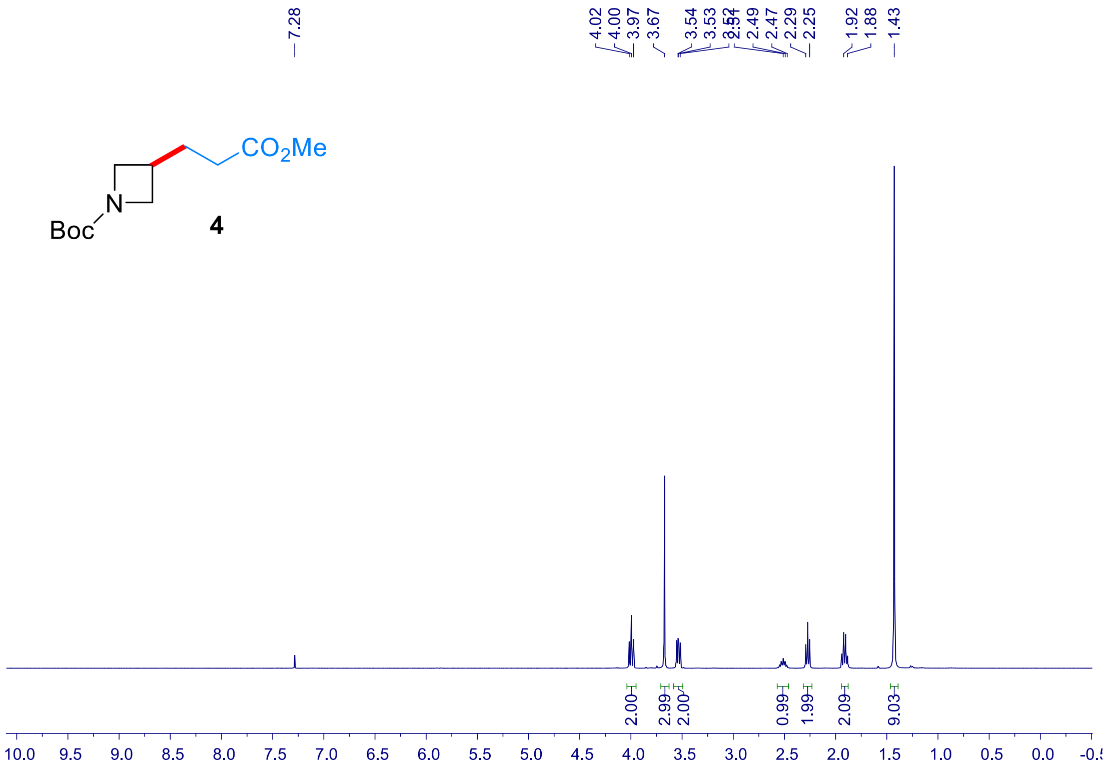

(4)- ^{13}C NMR (101 MHz, $CDCl_3$)

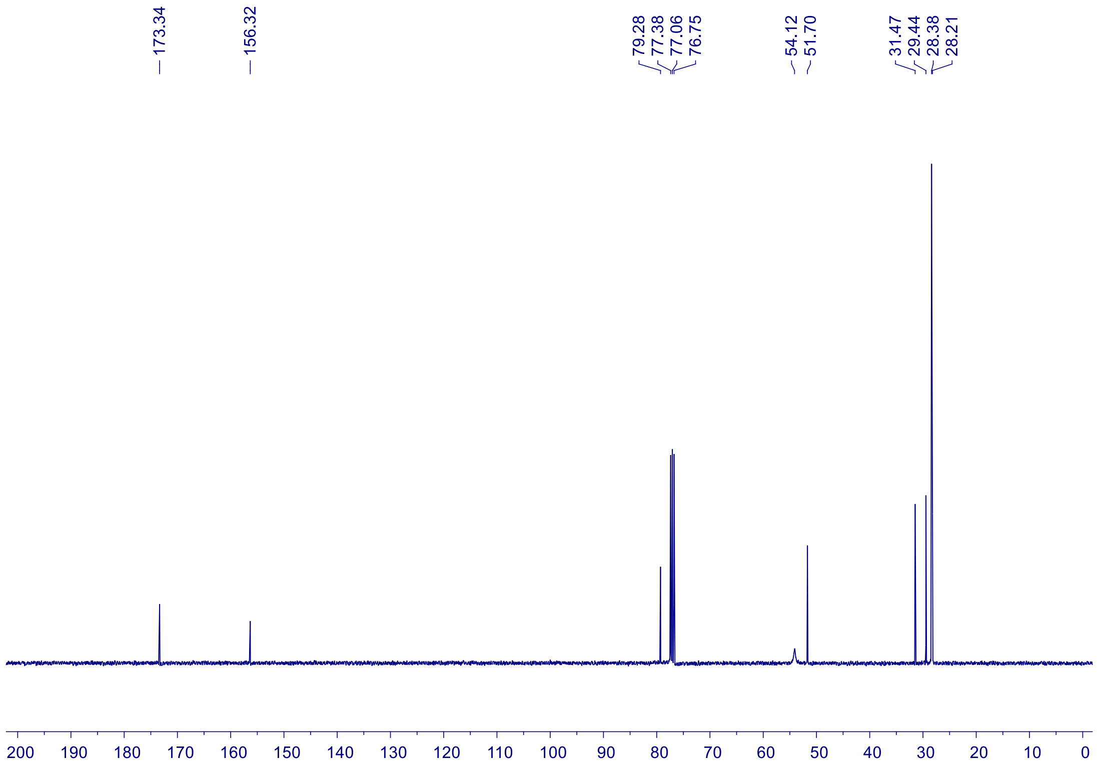

***tert*-butyl 3-(3-(*tert*-butoxy)-3-oxopropyl)azetidine-1-carboxylate (5)**-^{1}H NMR (400 MHz, $CDCl_3$)

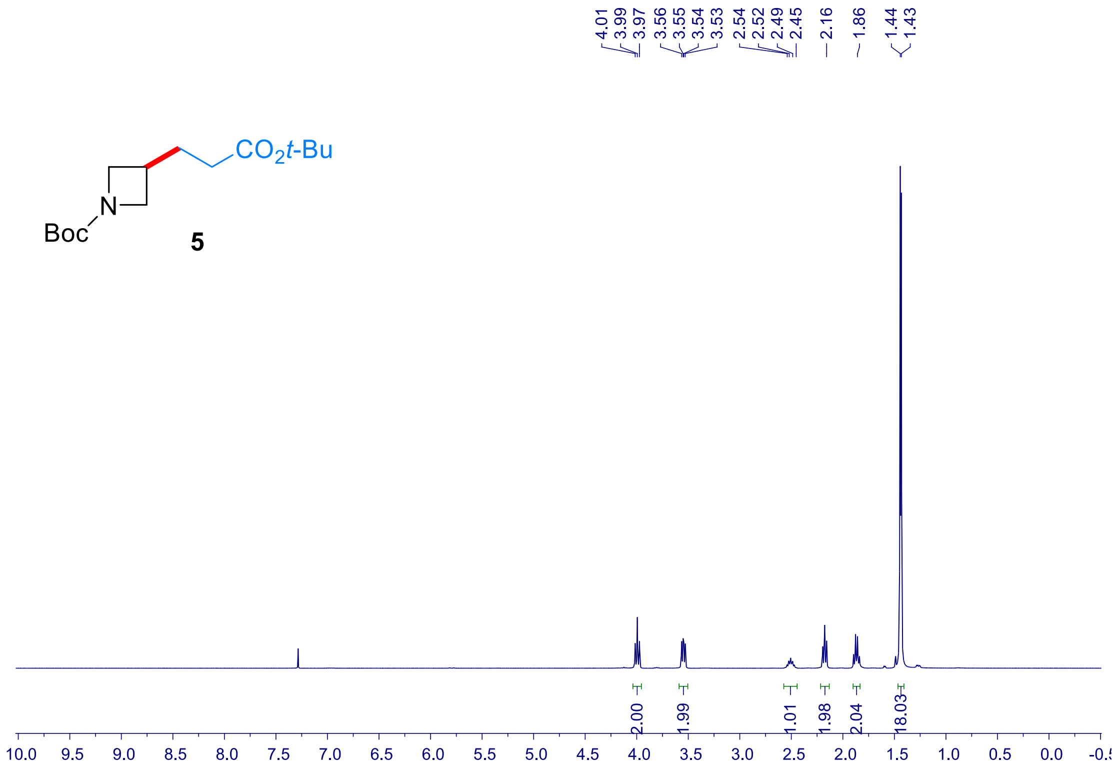

(5)- ^{13}C NMR (101 MHz, $CDCl_3$)

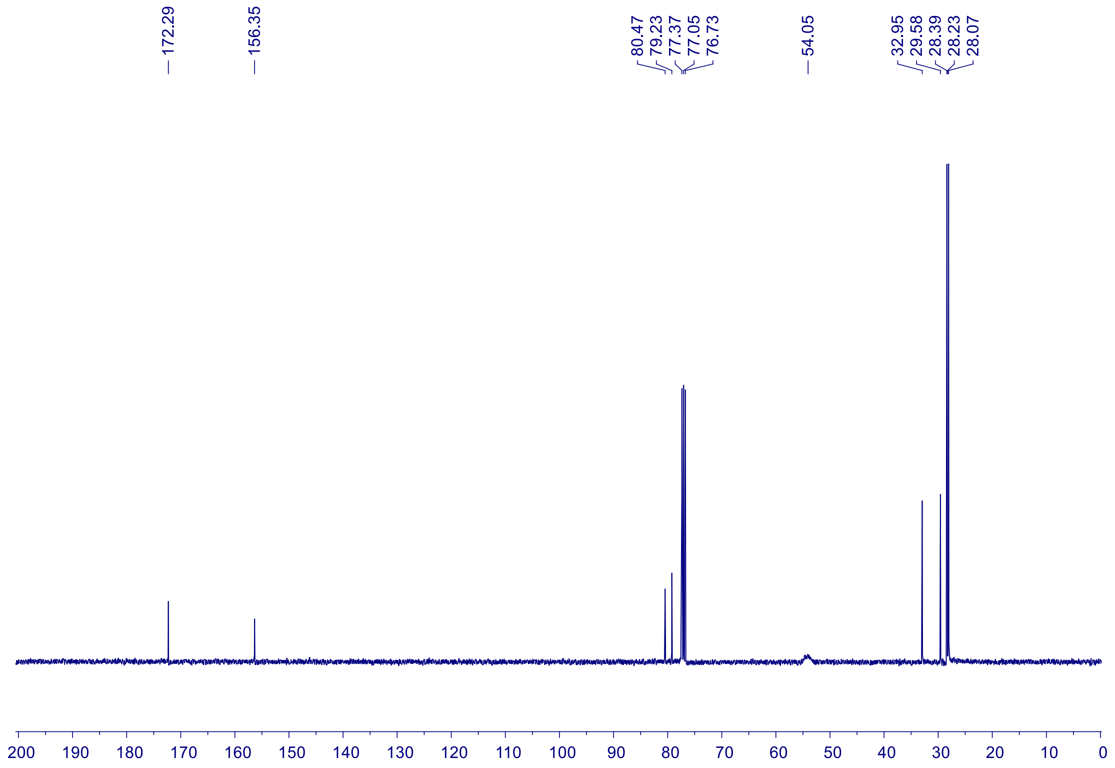

3-(1-(*tert*-butoxycarbonyl)azetidin-3-yl)propanoic acid (6)-^{1}H NMR (400 MHz, $CDCl_3$)

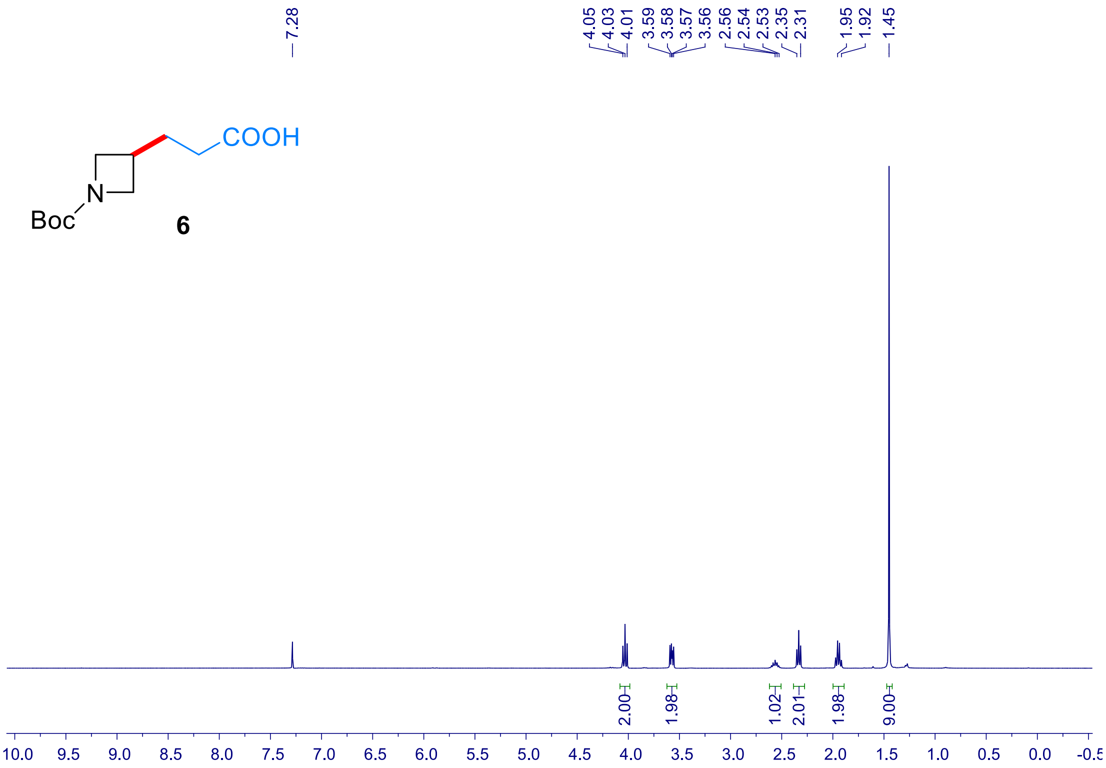

(6)- ^{13}C NMR (101 MHz, $CDCl_3$)

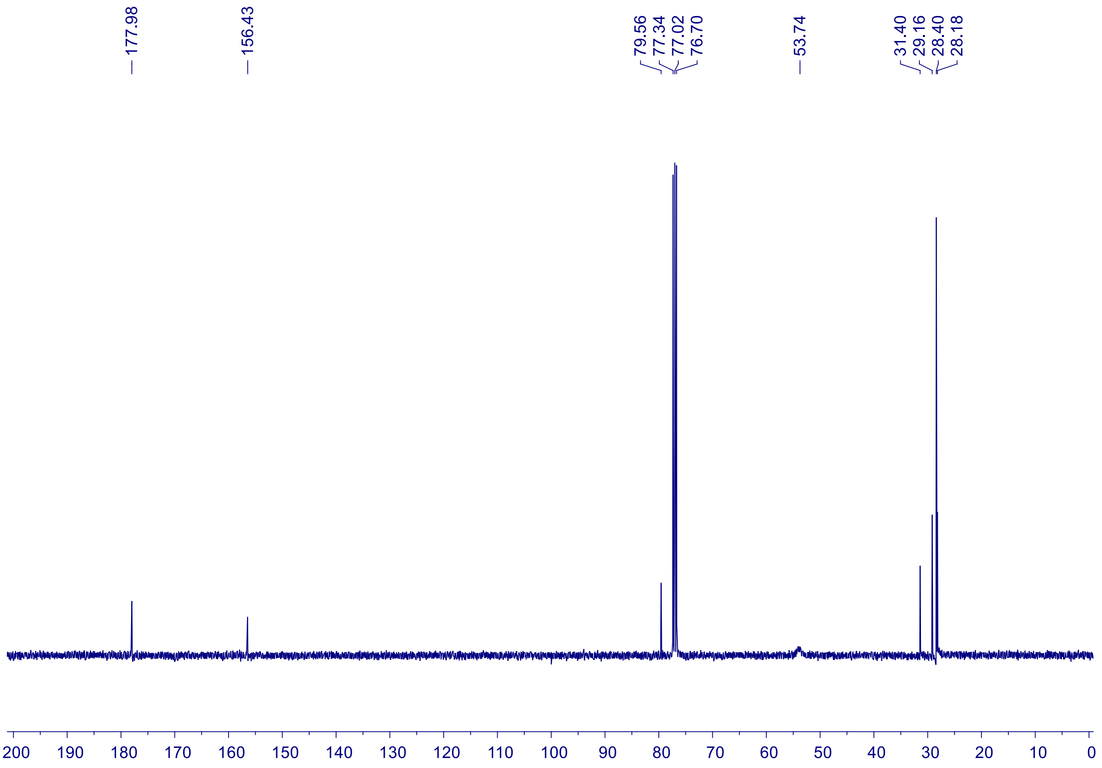

***tert*-butyl 3-(2-(phenylsulfonyl)ethyl)azetidine-1-carboxylate (7)**-^{1}H NMR (400 MHz, $CDCl_3$)

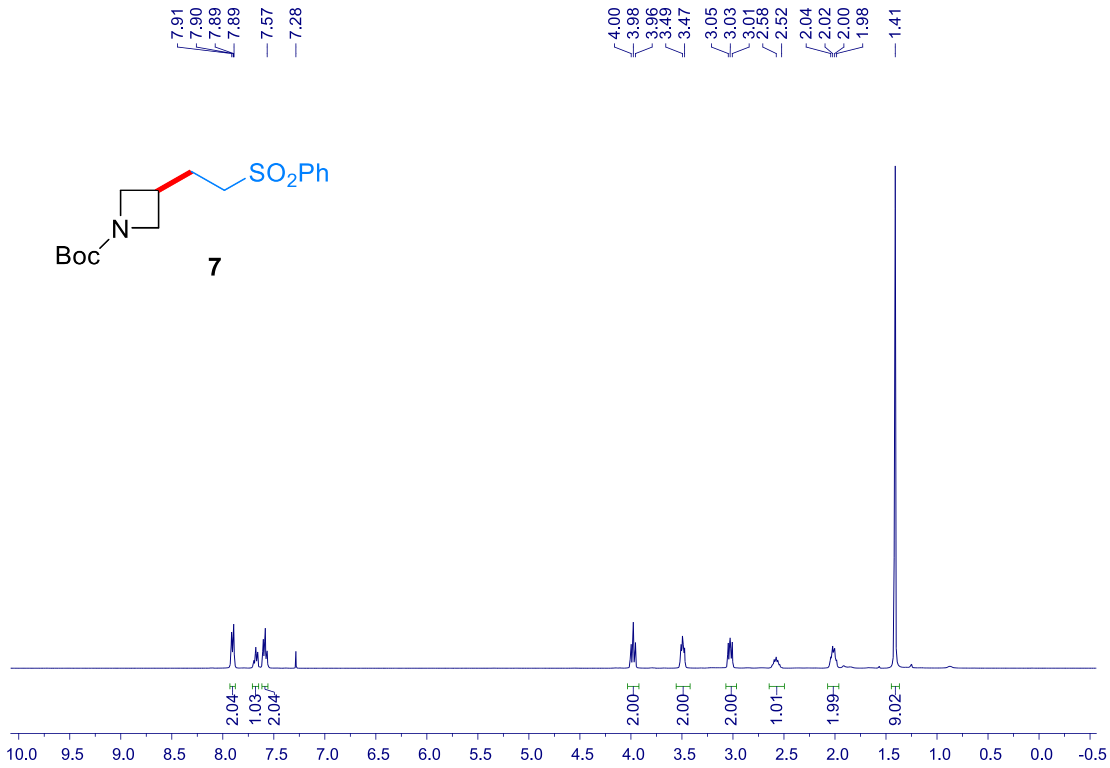

(7)-^{13}C NMR (101 MHz, $CDCl_3$)

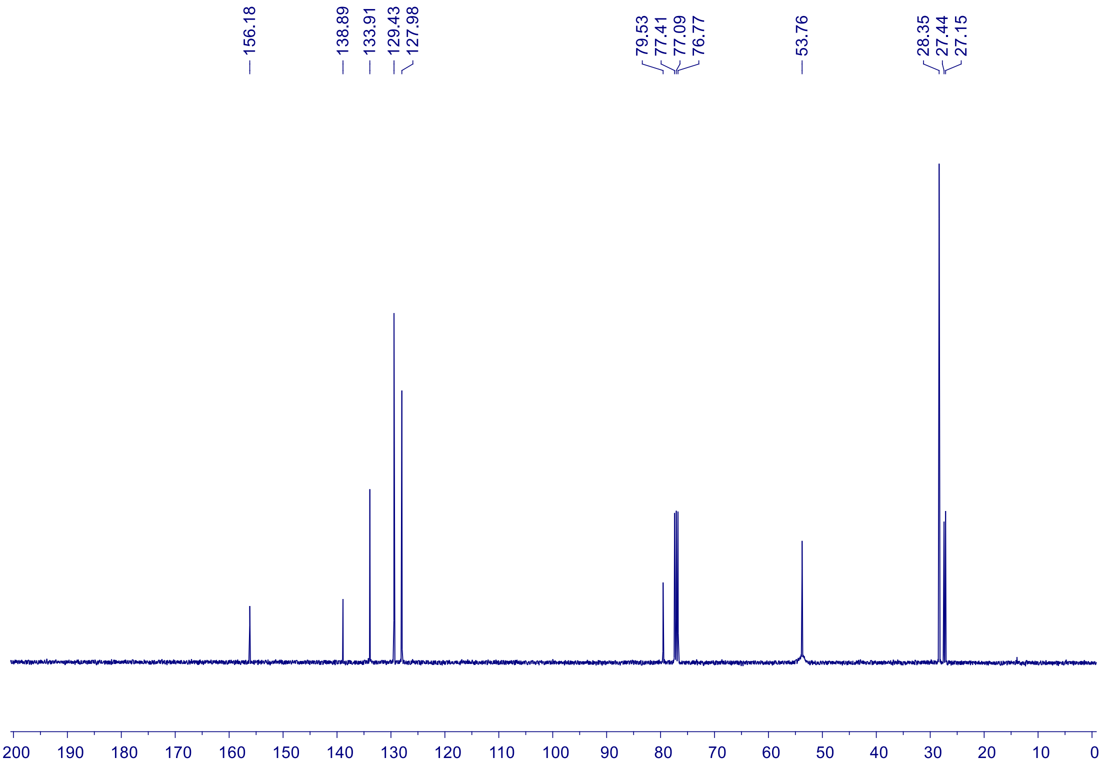

***tert*-butyl 3-(3-oxopentyl)azetidine-1-carboxylate (8)**-^{1}H NMR (400 MHz, $CDCl_3$)

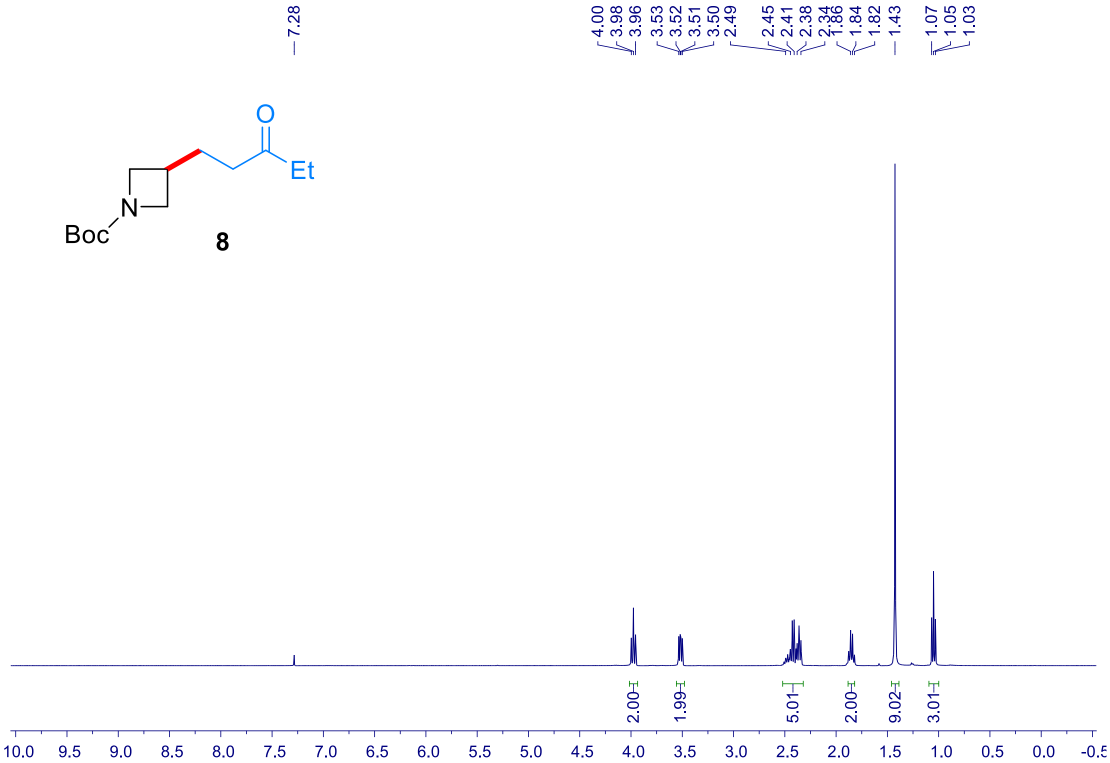

(8)-^{13}C NMR (101 MHz, $CDCl_3$)

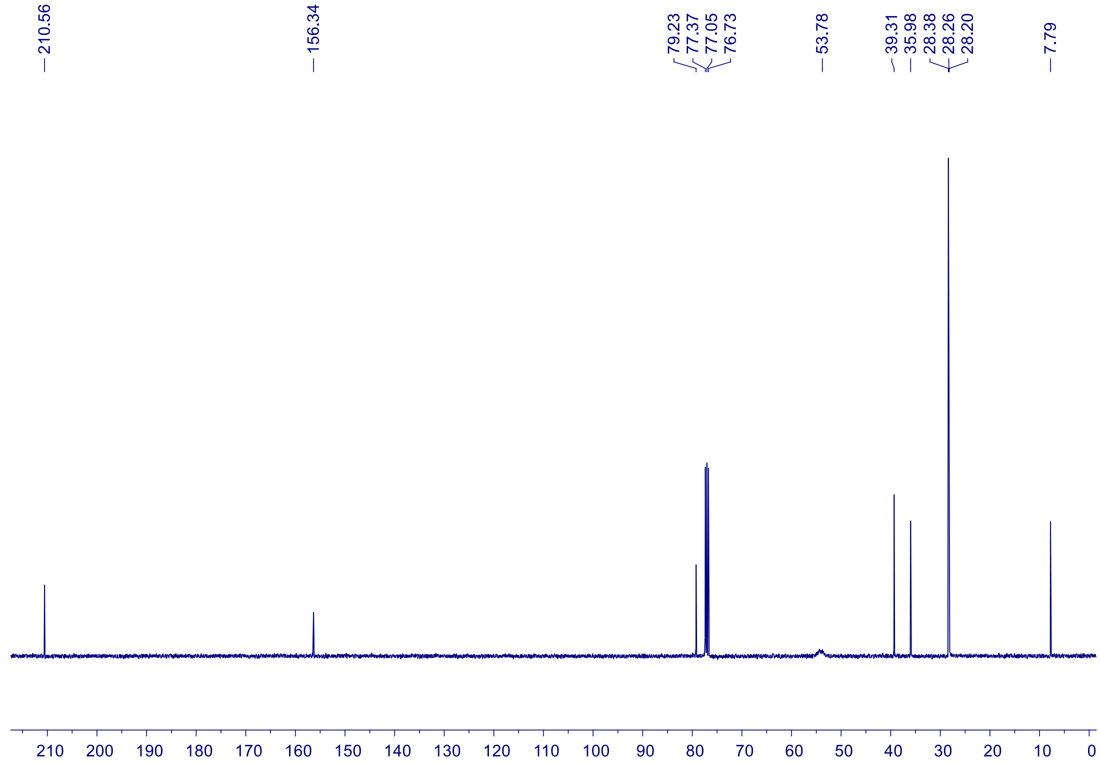

***tert*-butyl 3-(2-(diethoxyphosphoryl)ethyl)azetidine-1-carboxylate (9)**-^{1}H NMR (400 MHz, $CDCl_3$)

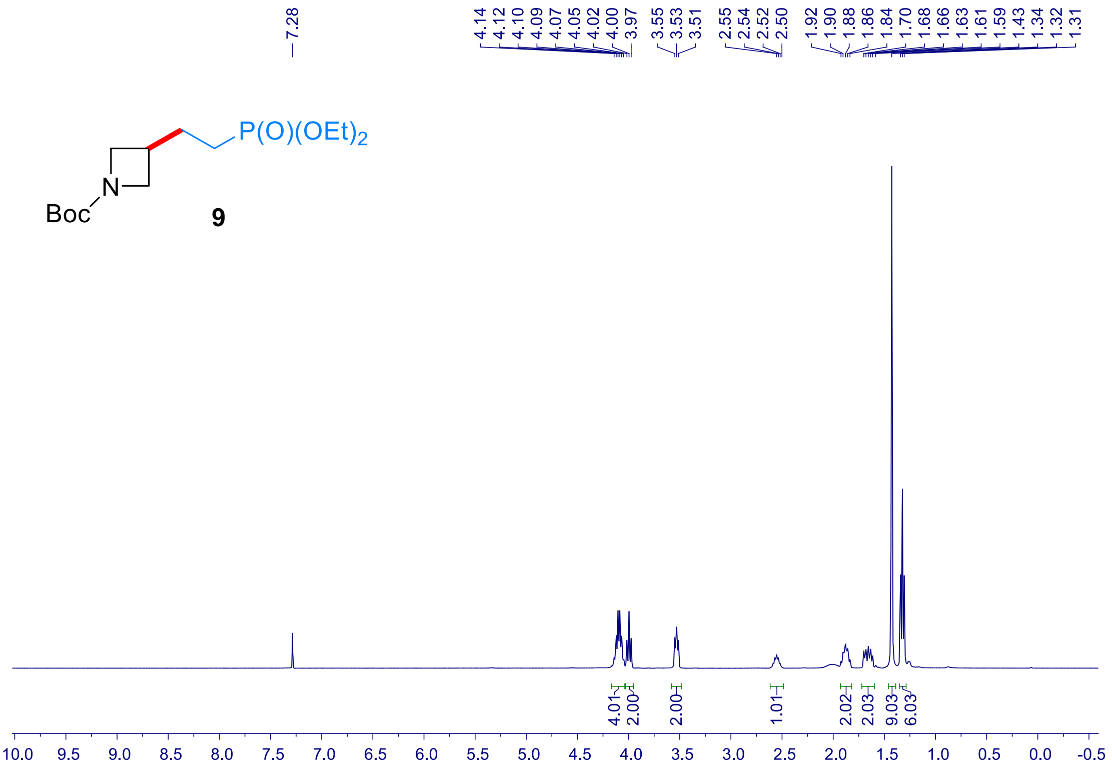

(9)-^{13}C NMR (101 MHz, $CDCl_3$)

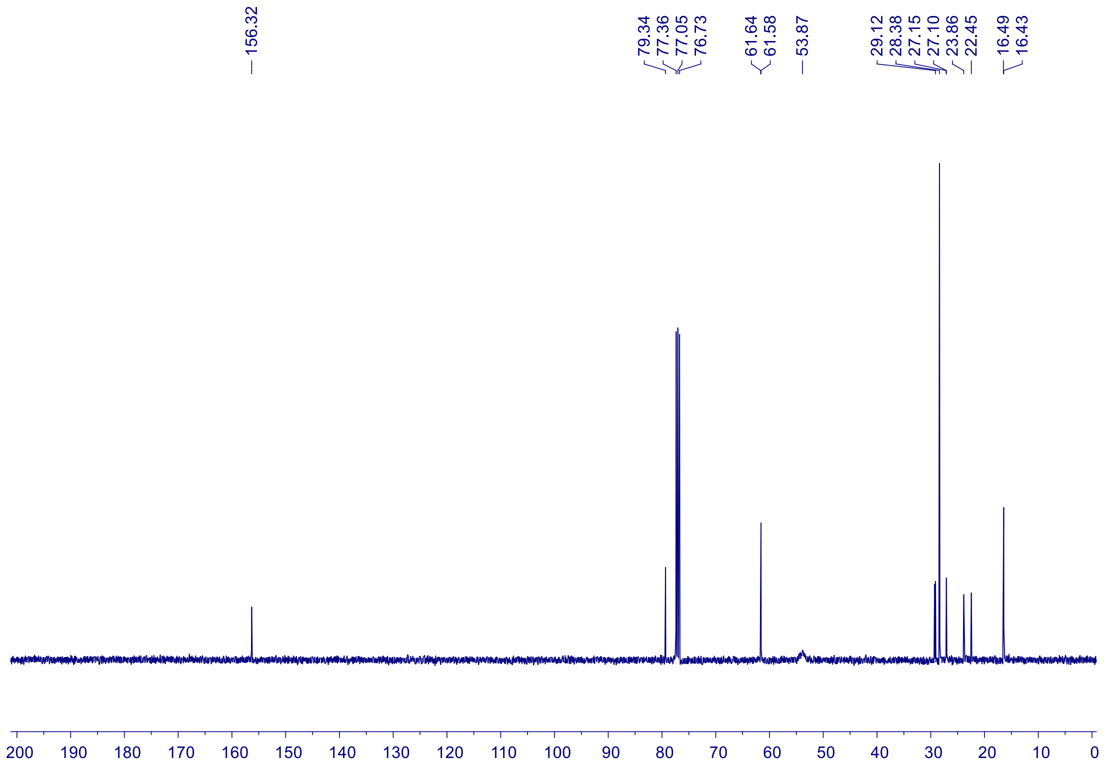

(9)-^{31}P NMR (162 MHz, $CDCl_3$)

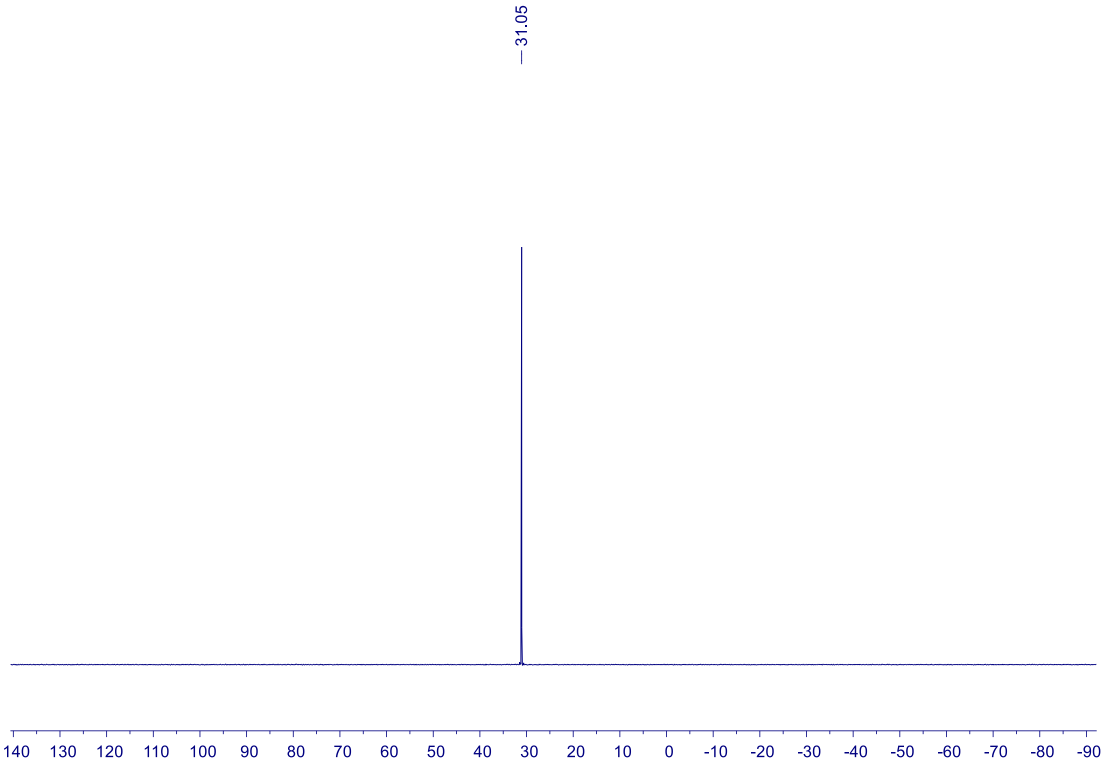

***tert*-butyl 3-(3-(dimethylamino)-3-oxopropyl)azetidine-1-carboxylate (10)-**^{1}H NMR (400 MHz, $CDCl_3$)

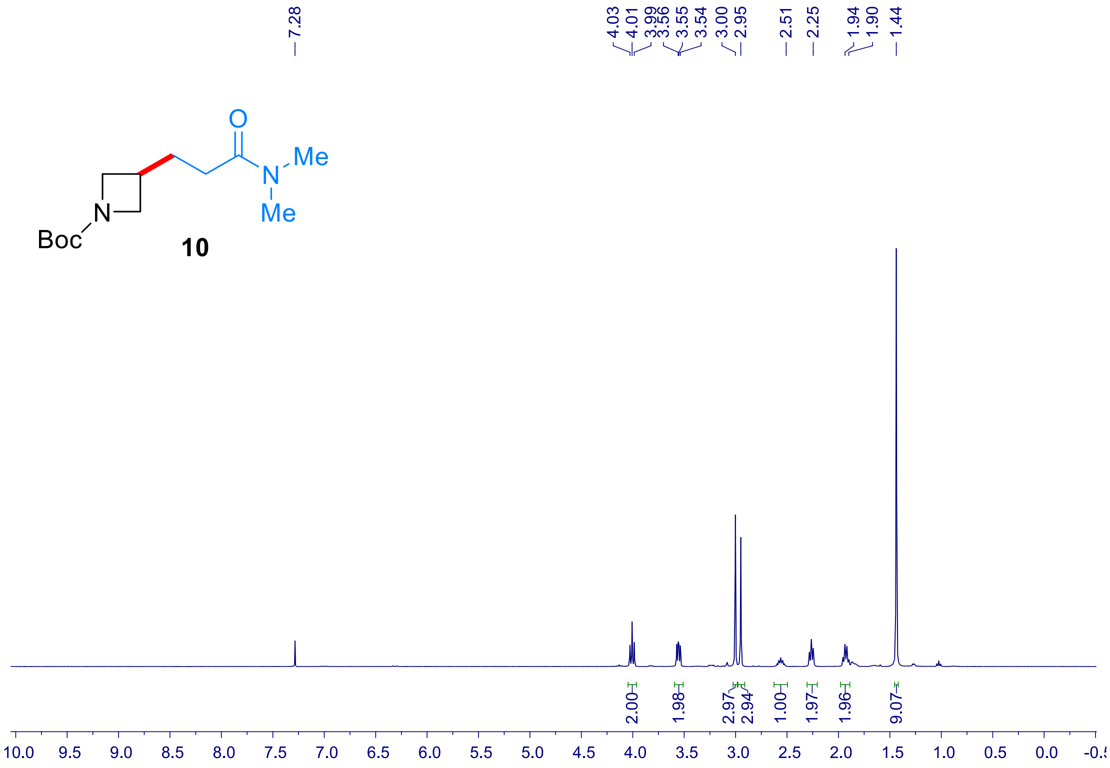

(10)-^{13}C NMR (101 MHz, $CDCl_3$)

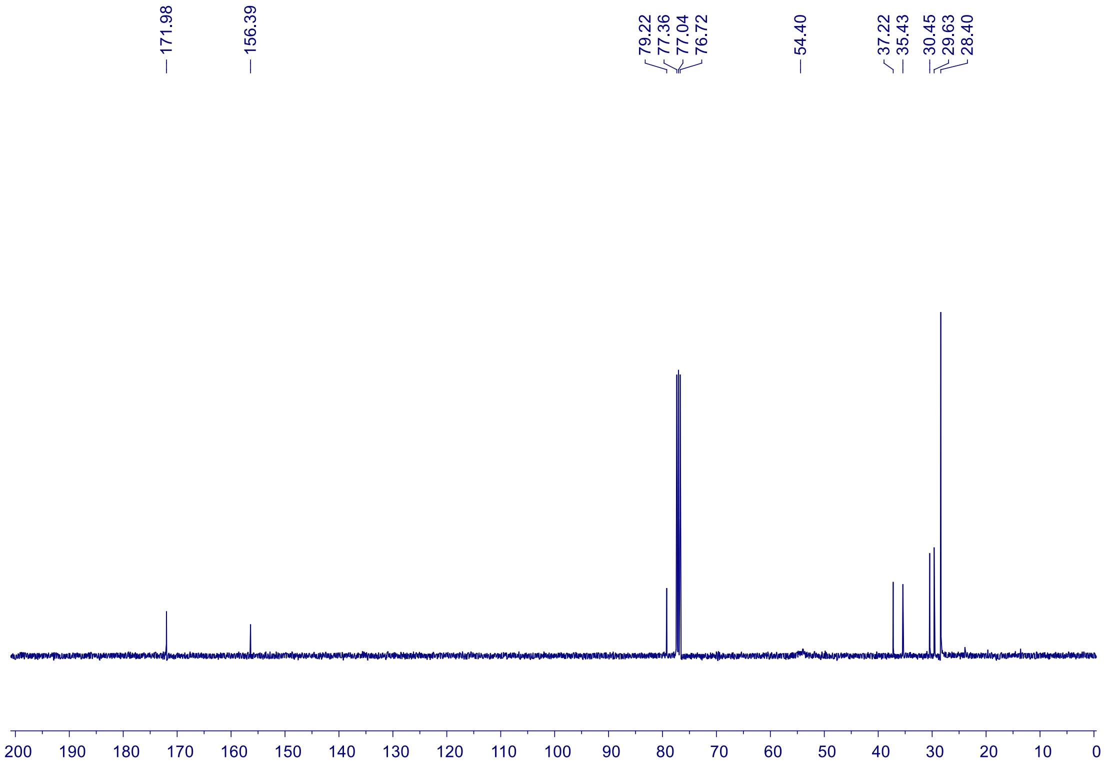

***tert*-butyl 3-(2-(pyridin-2-yl)ethyl)azetidine-1-carboxylate (11)**-^{1}H NMR (400 MHz, $CDCl_3$)

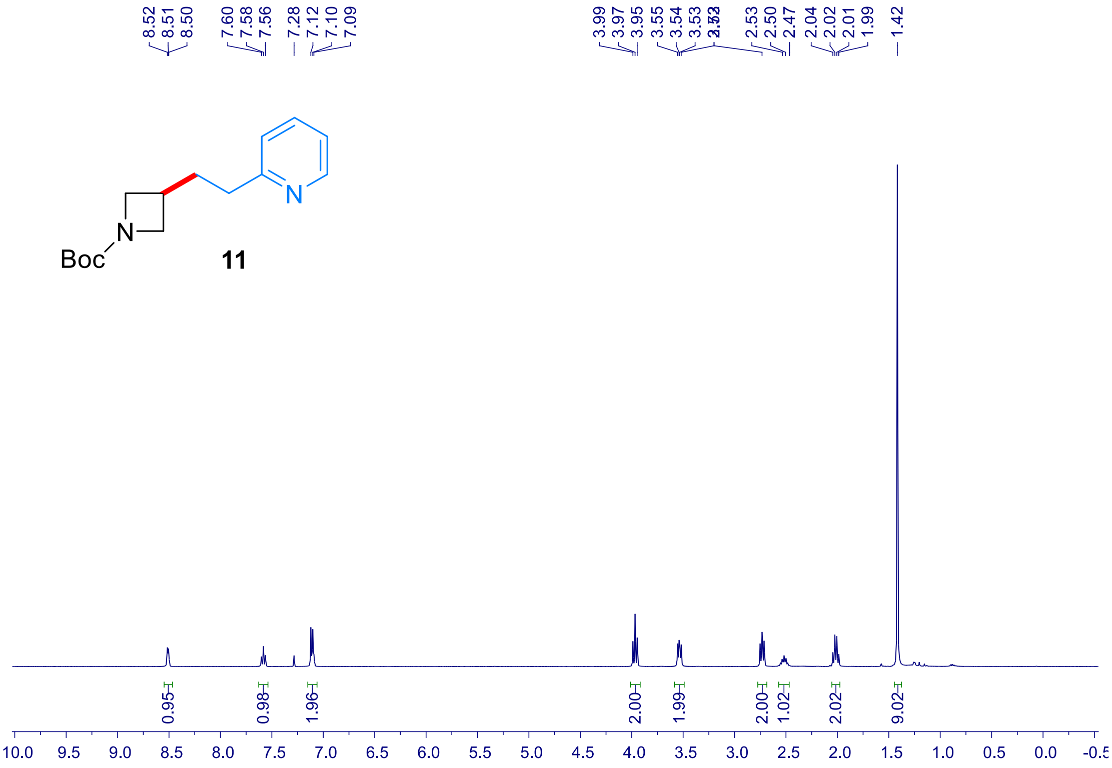

(11)-^{13}C NMR (101 MHz, $CDCl_3$)

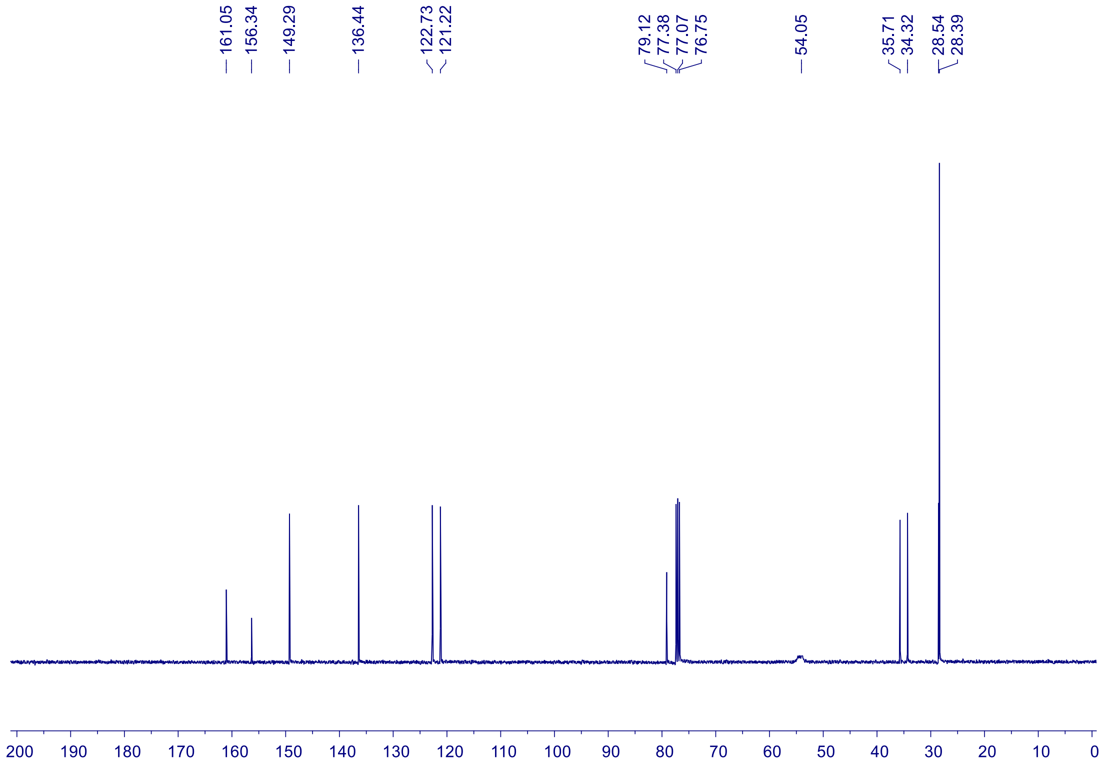

***tert*-butyl 3-(3-oxo-3-(2,2,2-trifluoroethoxy)propyl)azetidine-1-carboxylate (12)-**
^{1}H NMR (400 MHz, $CDCl_3$)

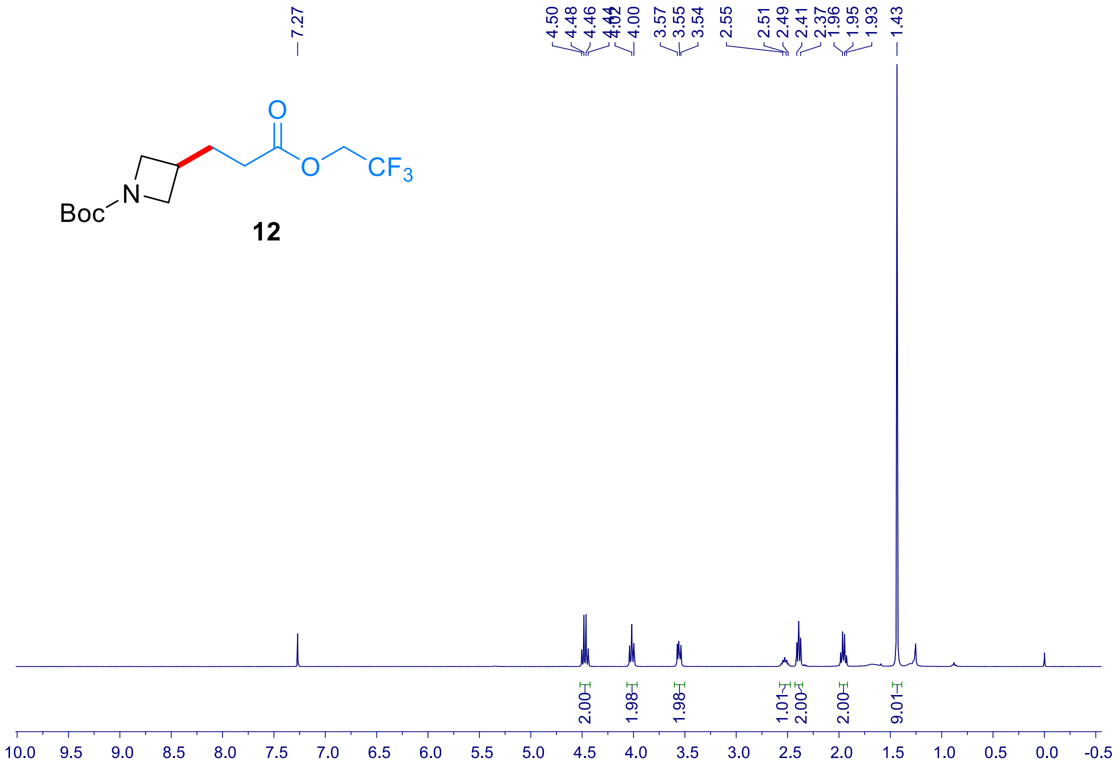

(12)-^{13}C NMR (101 MHz, $CDCl_3$)

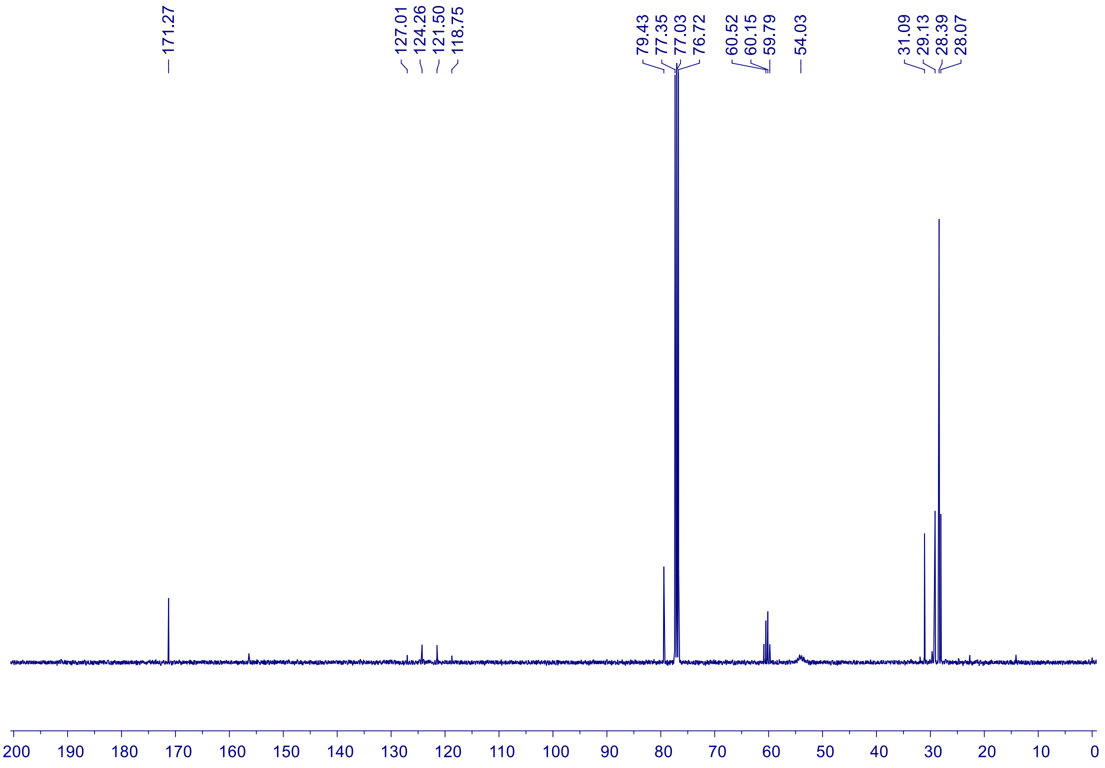

(12)-^{19}F NMR: (376 MHz, $CDCl_3$)

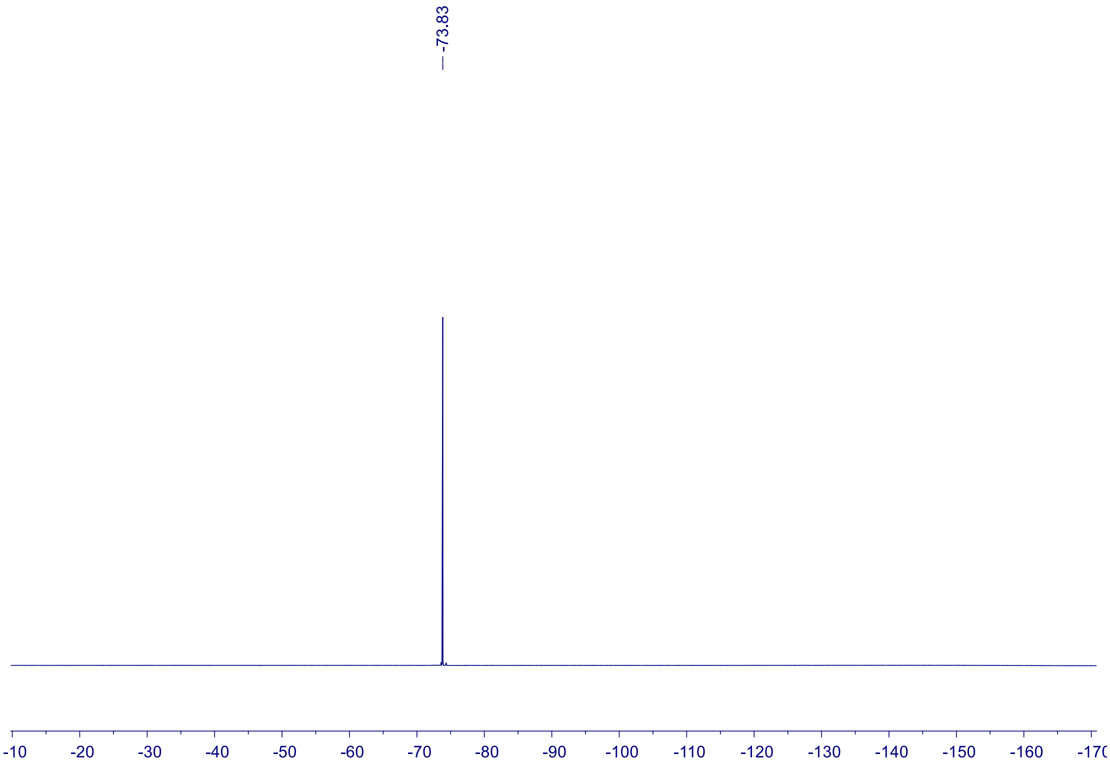

***tert*-butyl 3-(3-(2-chloroethoxy)-3-oxopropyl)azetidine-1-carboxylate (13)**-^{1}H NMR (400 MHz, $CDCl_3$)

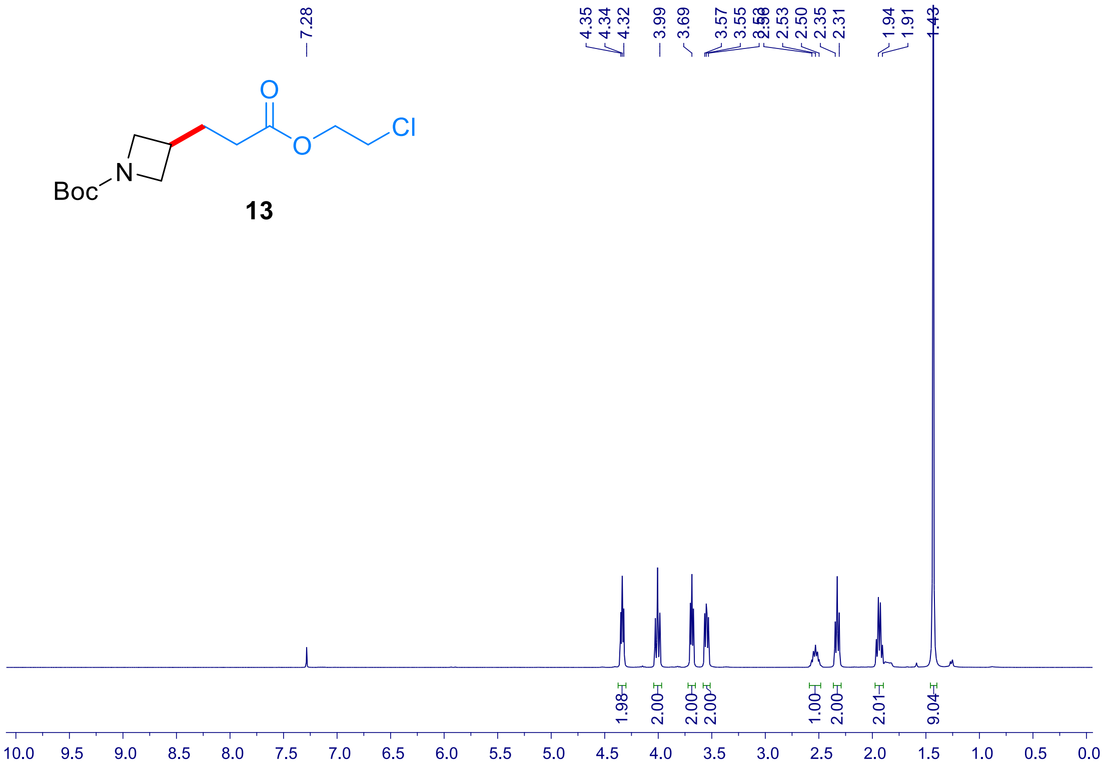

(13)-^{13}C NMR (101 MHz, $CDCl_3$)

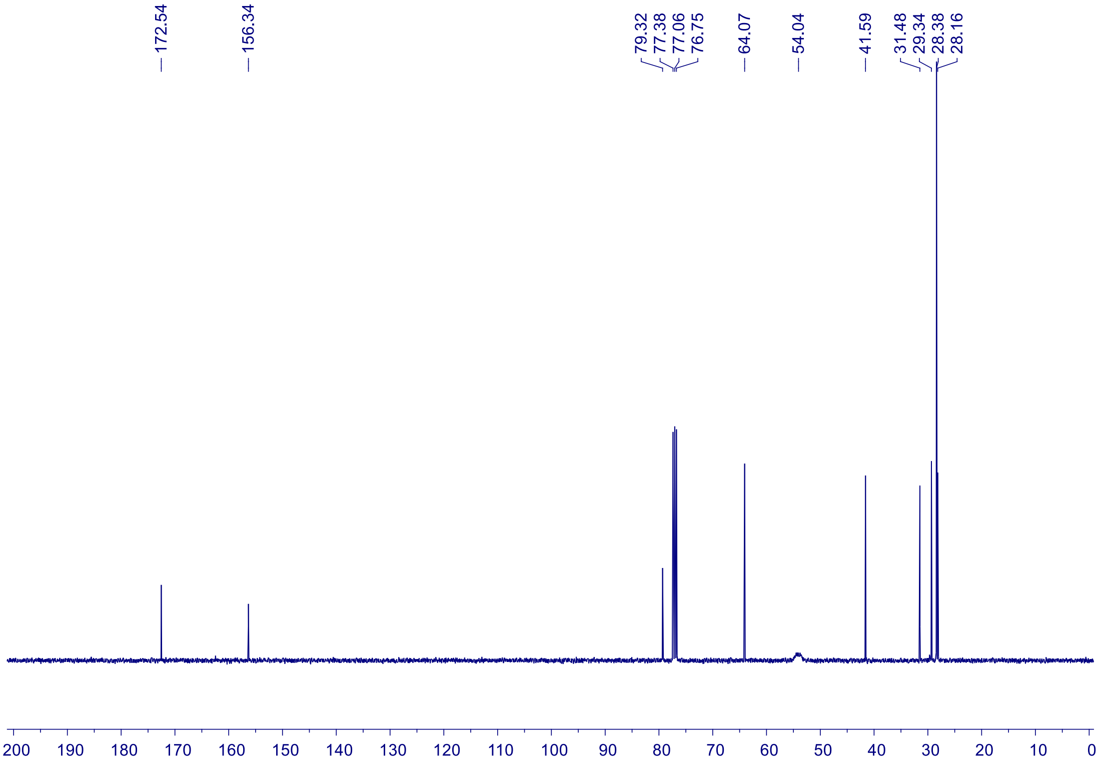

***tert*-butyl 3-(3-(2-bromoethoxy)-3-oxopropyl)azetidine-1-carboxylate (14)-**^{1}H NMR (400 MHz, $CDCl_3$)

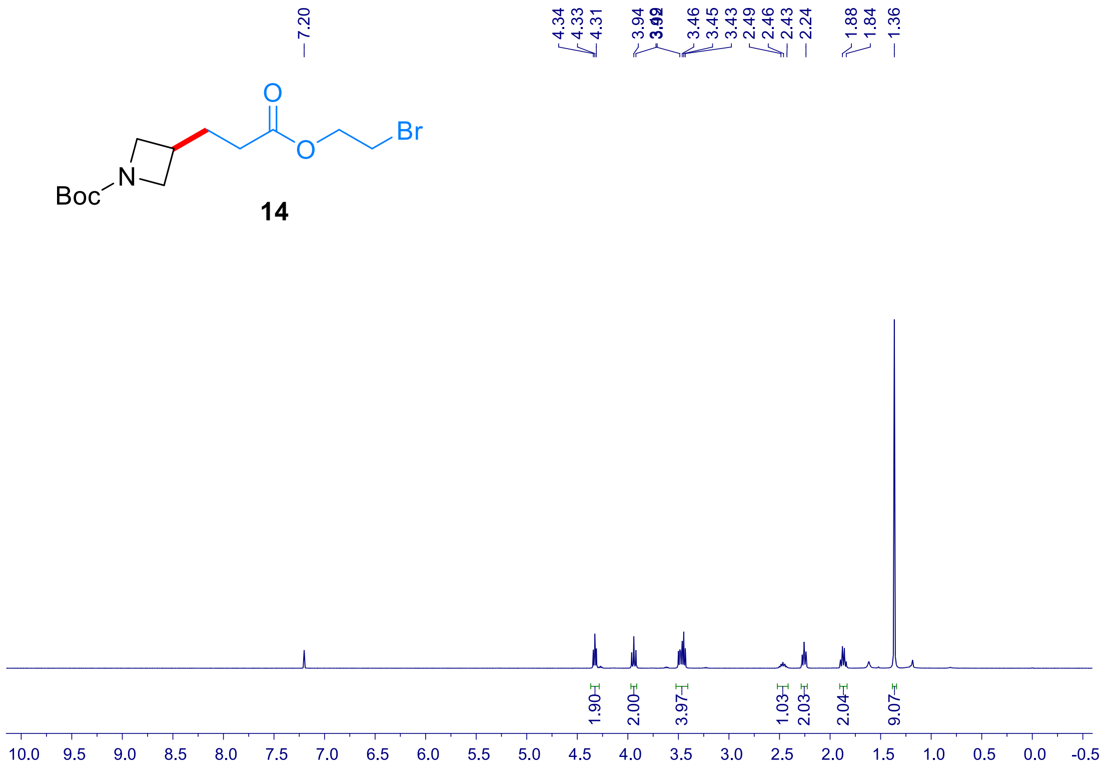

(14)-^{13}C NMR (101 MHz, $CDCl_3$)

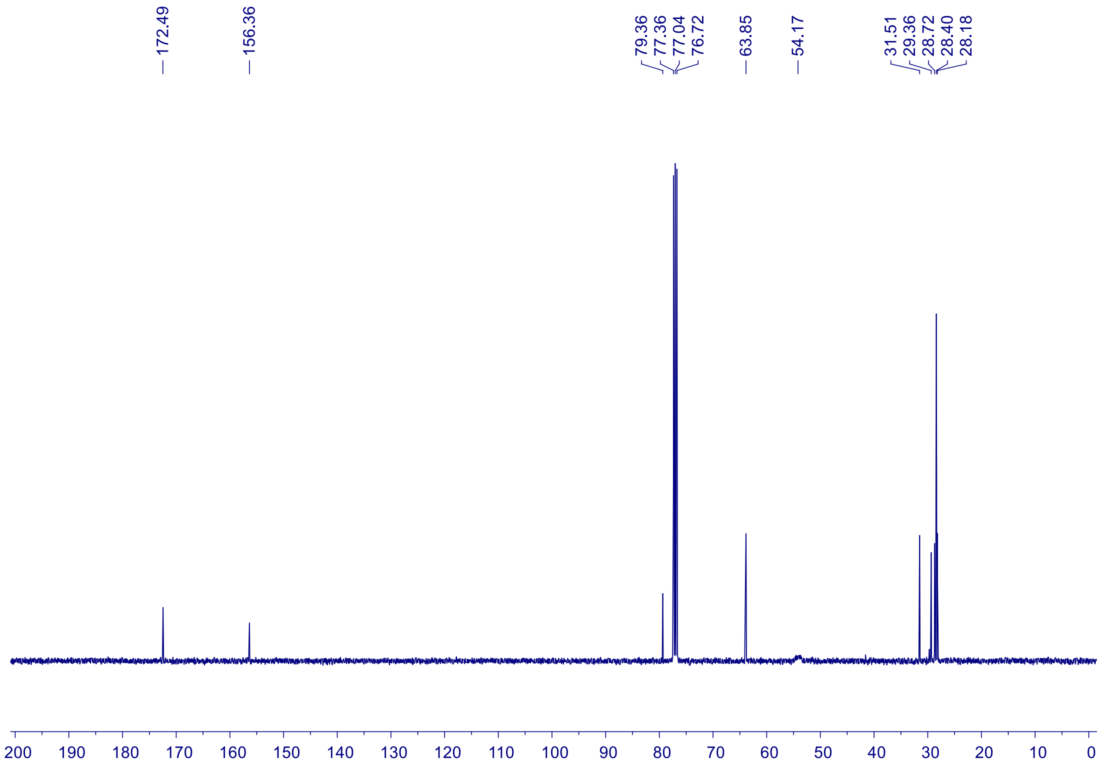

***tert*-butyl 3-(3-(oxiran-2-ylmethoxy)-3-oxopropyl)azetidine-1-carboxylate (15)-**^{1}H NMR (400 MHz, $CDCl_3$)

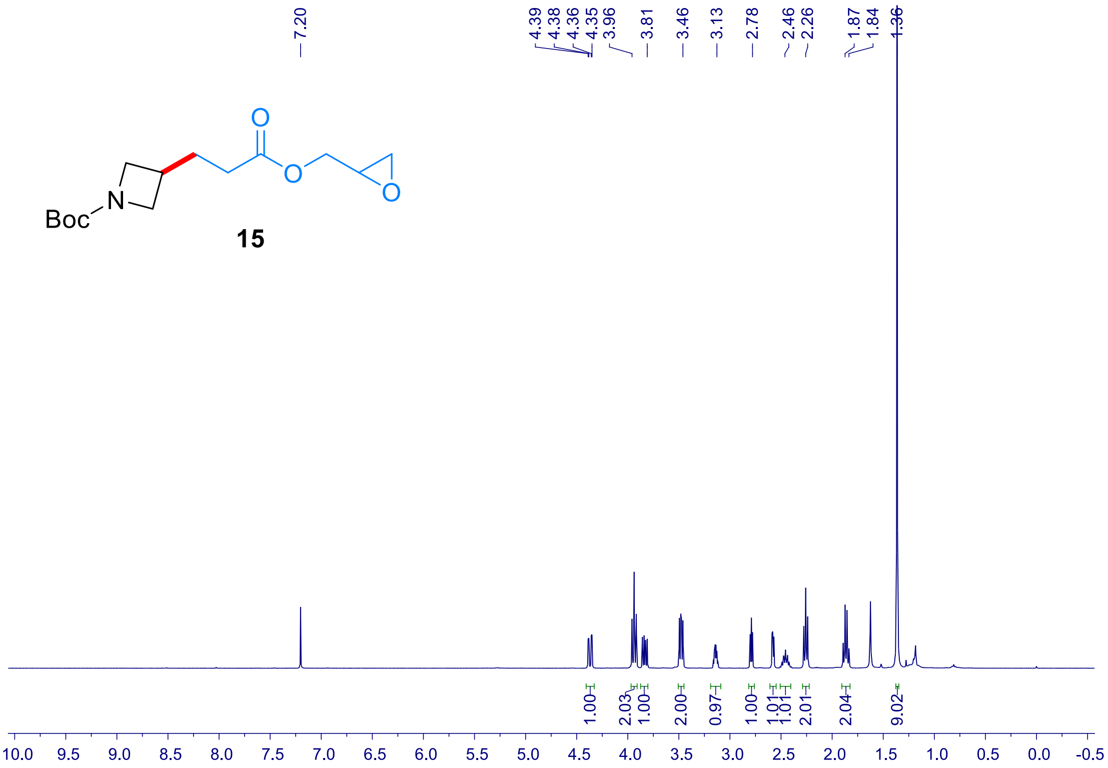

(15)-^{13}C NMR (101 MHz, $CDCl_3$)

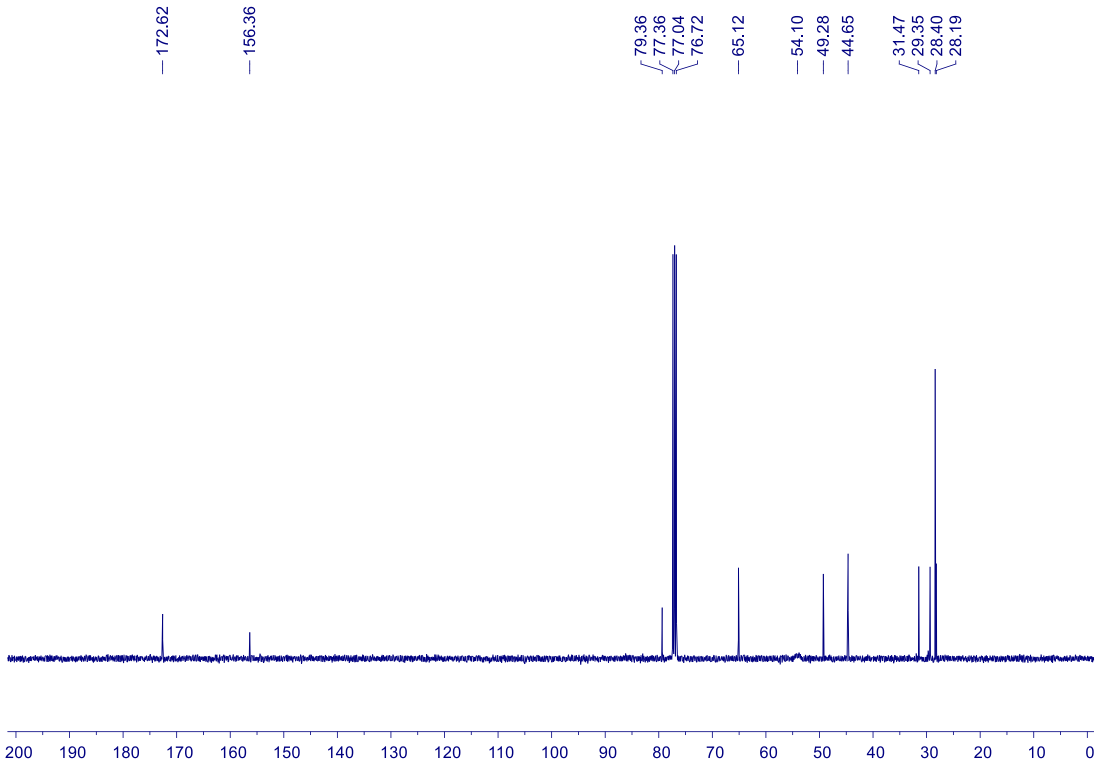

tert-butyl 3-(3-methoxy-2-methyl-3-oxopropyl)azetidine-1-carboxylate (16)-^{1}H NMR (400 MHz, $CDCl_3$)

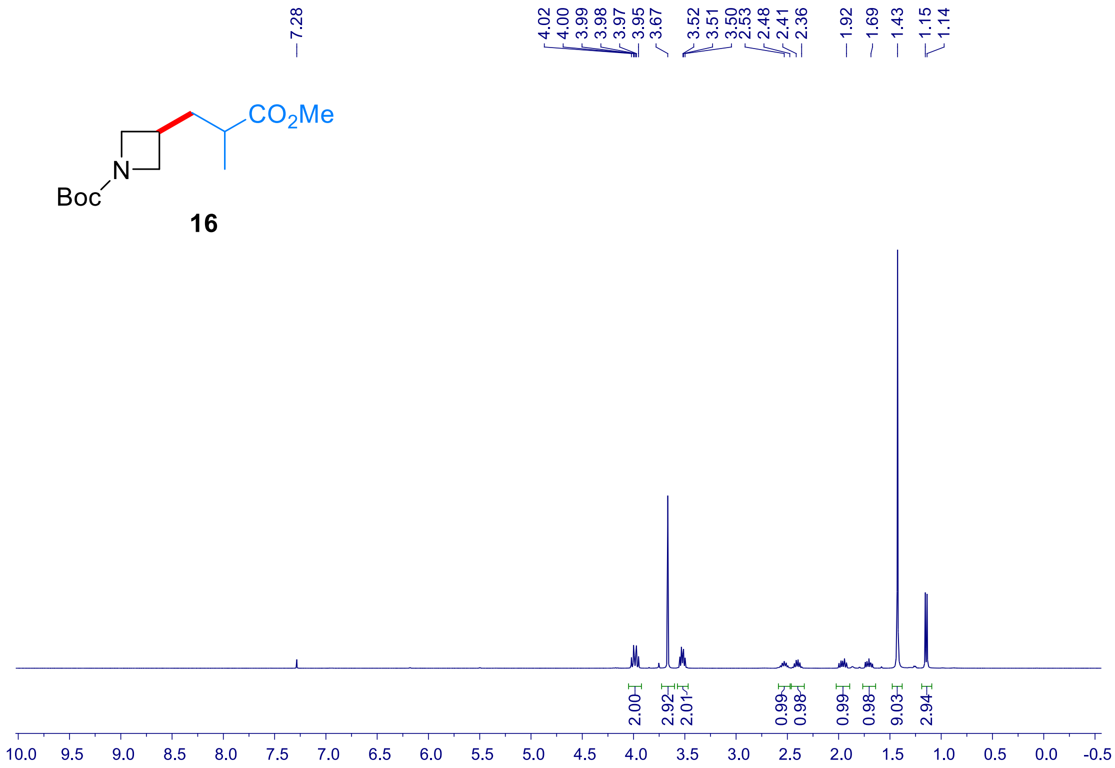

(16)-^{13}C NMR (101 MHz, $CDCl_3$)

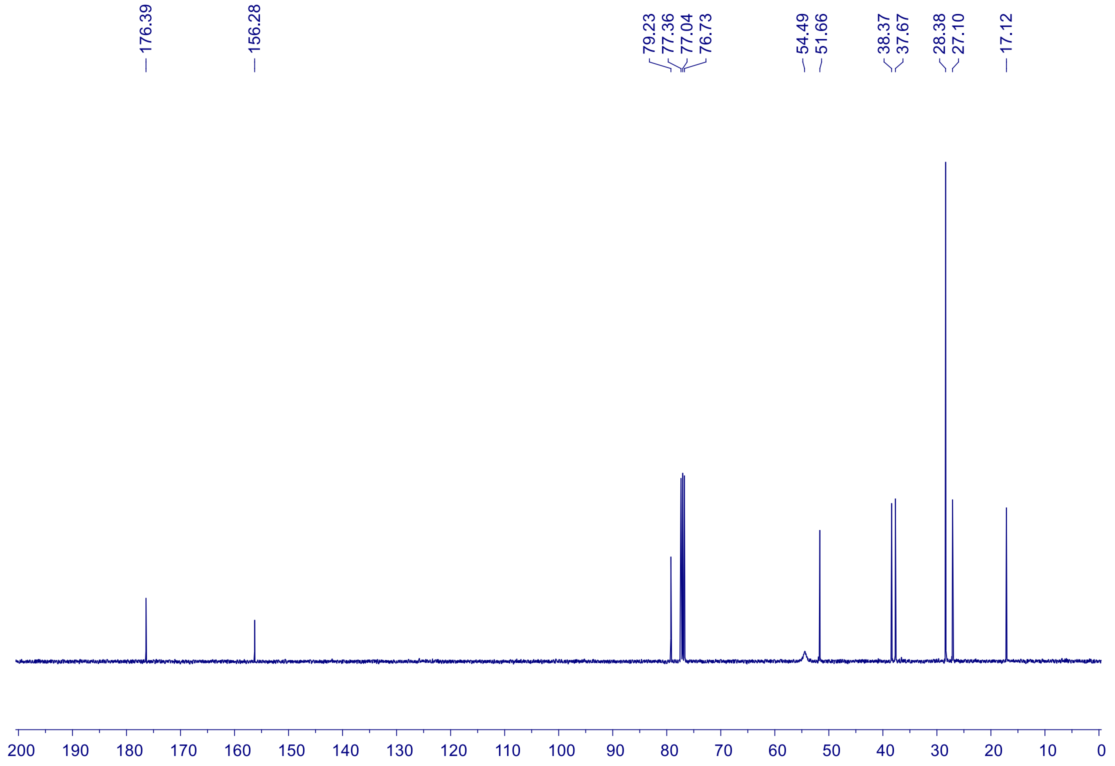

***tert*-butyl 3-(4-methoxy-4-oxobutan-2-yl)azetidine-1-carboxylate (17)**-^{1}H NMR (400 MHz, $CDCl_3$)

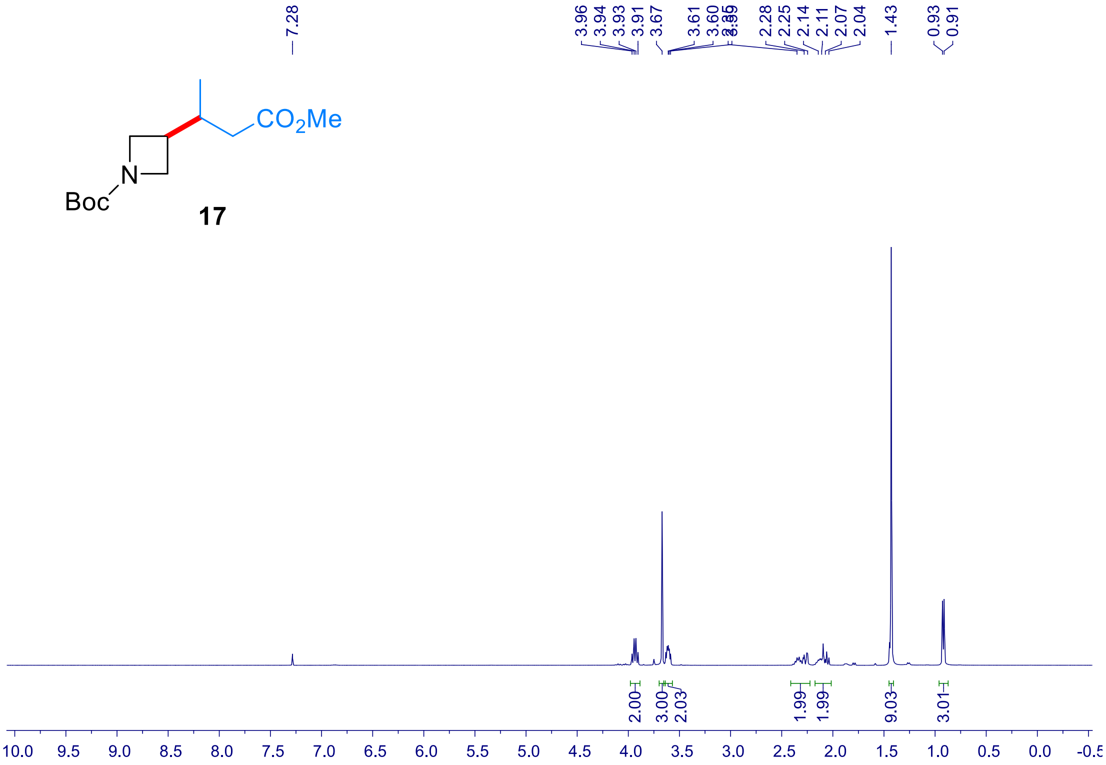

(17)-^{13}C NMR (101 MHz, $CDCl_3$)

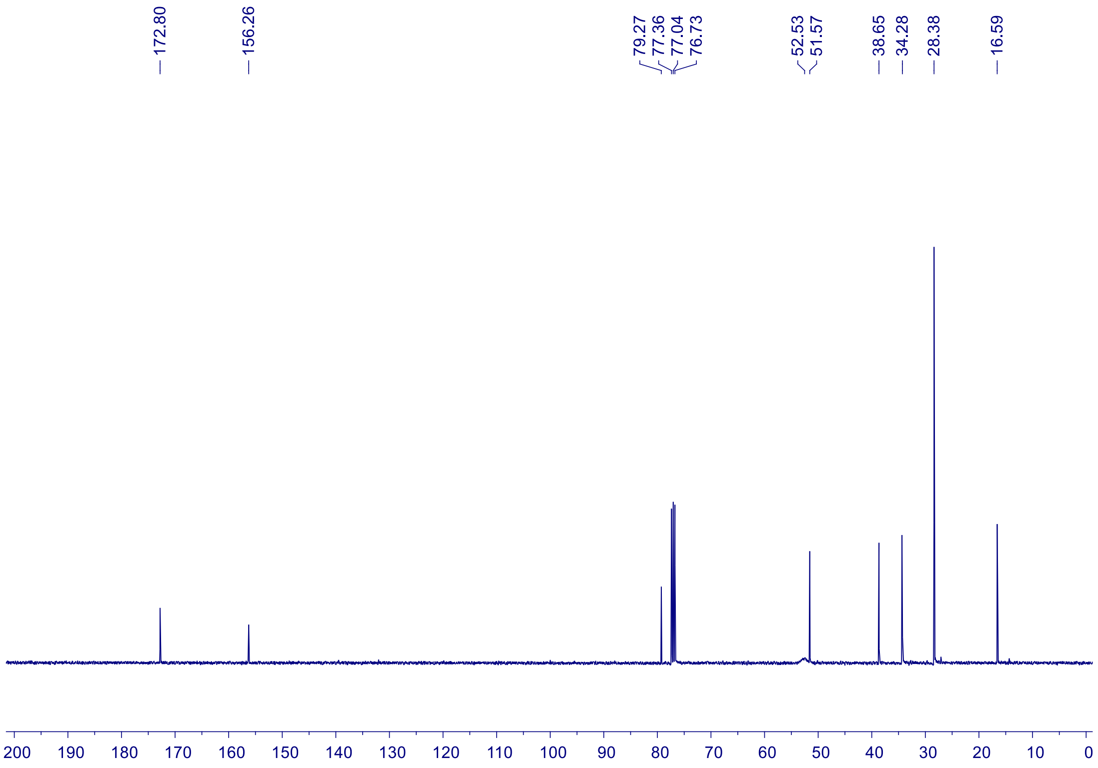

Dimethyl 2-(1-(*tert*-butoxycarbonyl)azetidin-3-yl)succinate (18)-^{1}H NMR (400 MHz, $CDCl_3$)

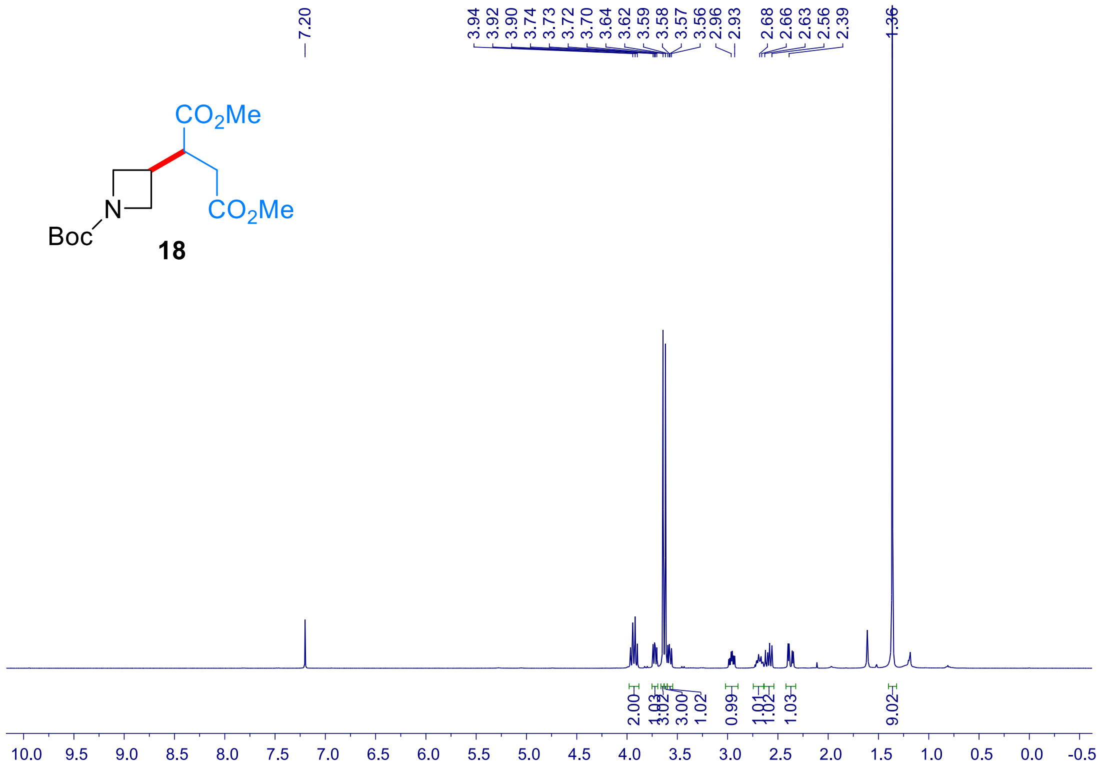

(18)-^{13}C NMR (101 MHz, $CDCl_3$)

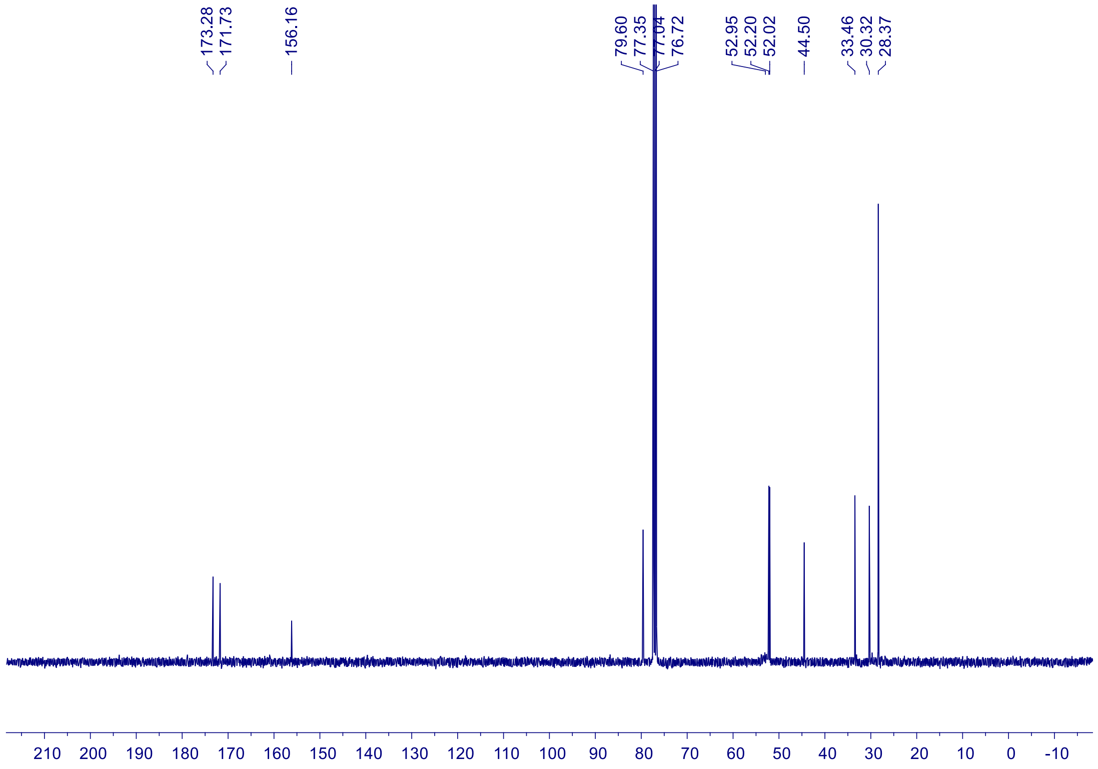

***tert*-butyl 3-((2-oxotetrahydrofuran-3-yl)methyl)azetidine-1-carboxylate (19)**-^{1}H NMR (400 MHz, $CDCl_3$)

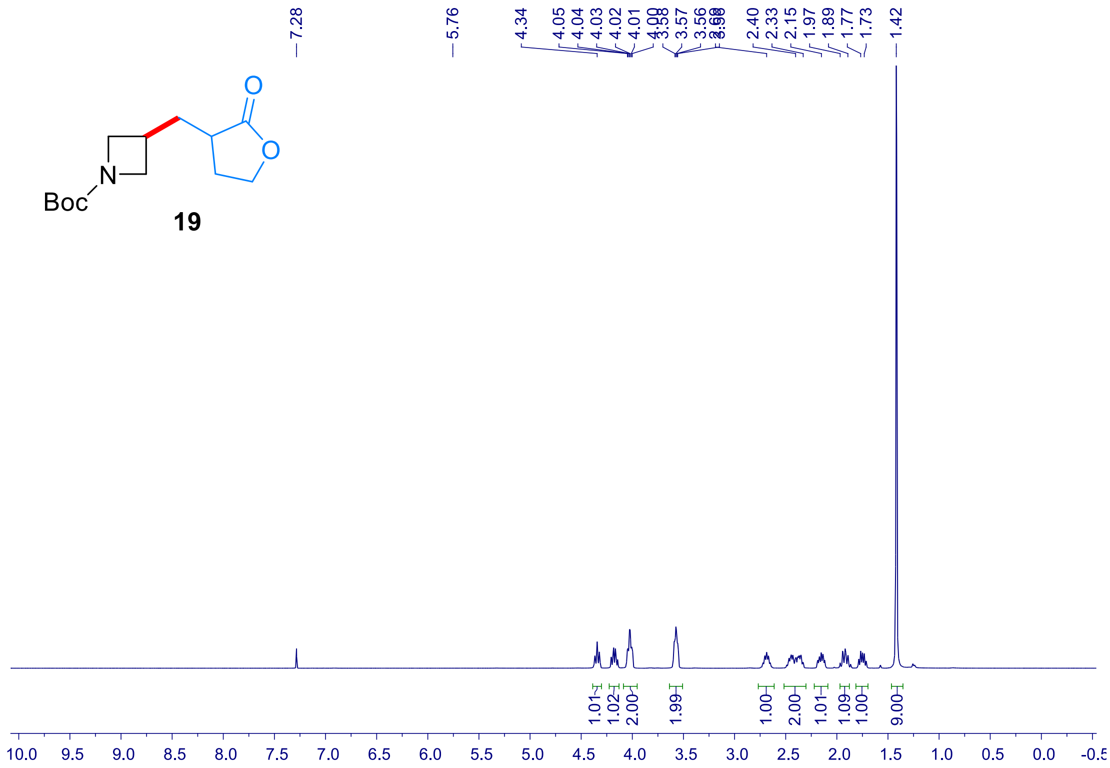

(19)-^{13}C NMR (101 MHz, $CDCl_3$)

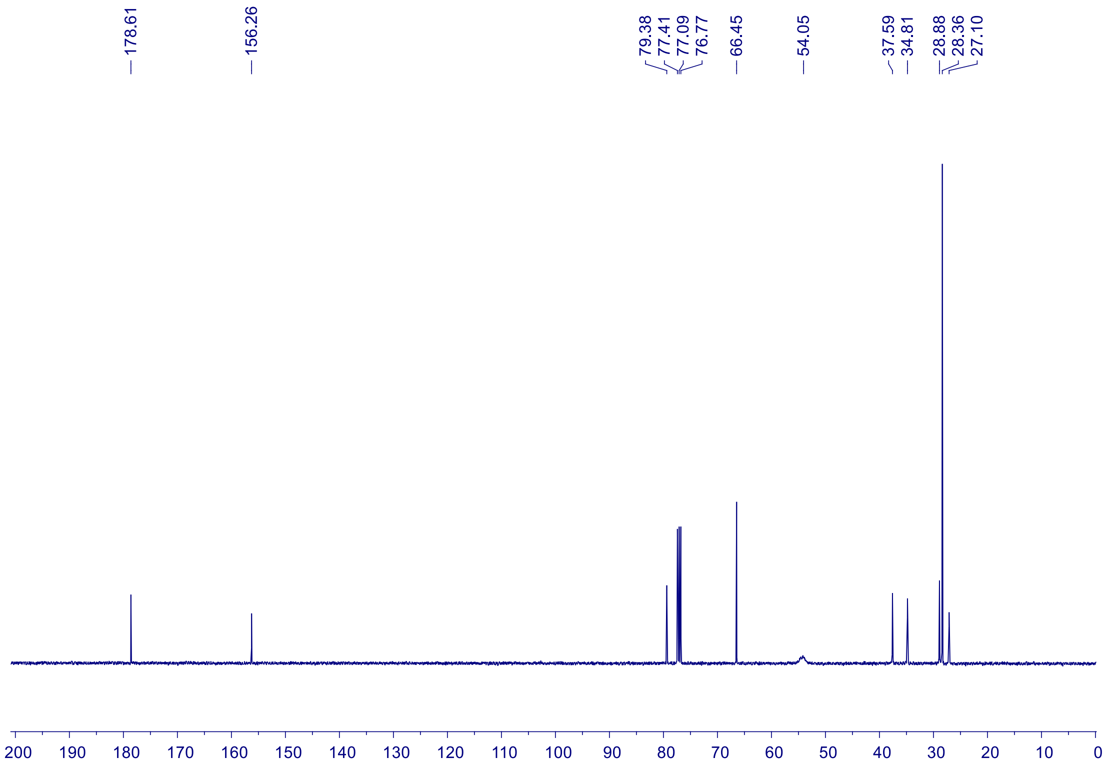

Benzyl (2*R*)-4-((1-(*tert*-butoxycarbonyl)azetidin-3-yl)methyl)-2-(*tert*-butyl)-5-oxo oxazolidine-3-carboxylate (20)-^{1}H NMR (400 MHz, $CDCl_3$)

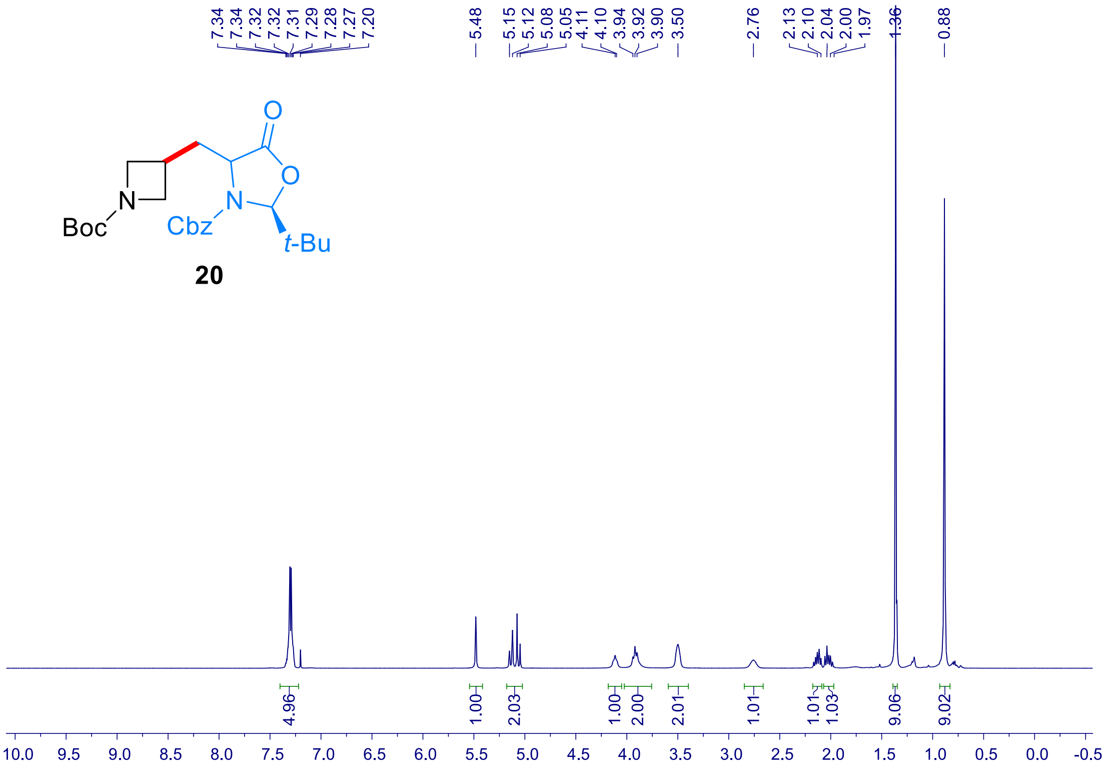

(20)-^{13}C NMR (101 MHz, $CDCl_3$)

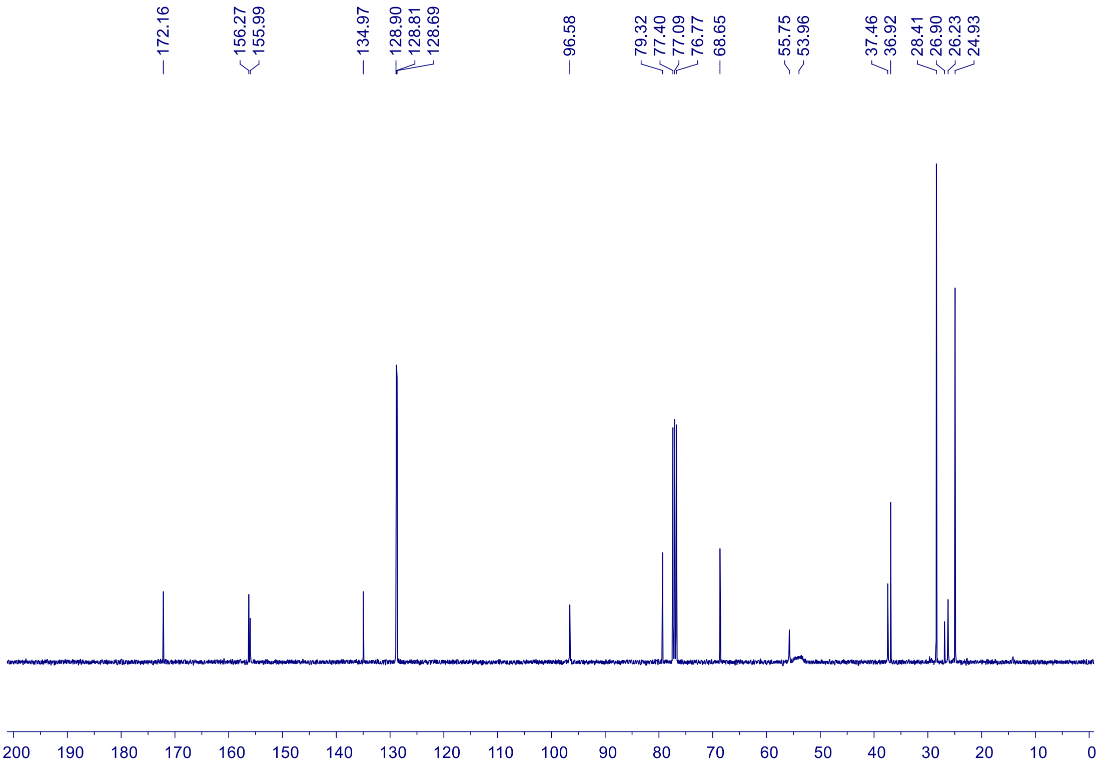

***tert*-butyl 3-(3-oxocyclopentyl)azetidine-1-carboxylate (21)**-^{1}H NMR (400 MHz, $CDCl_3$)

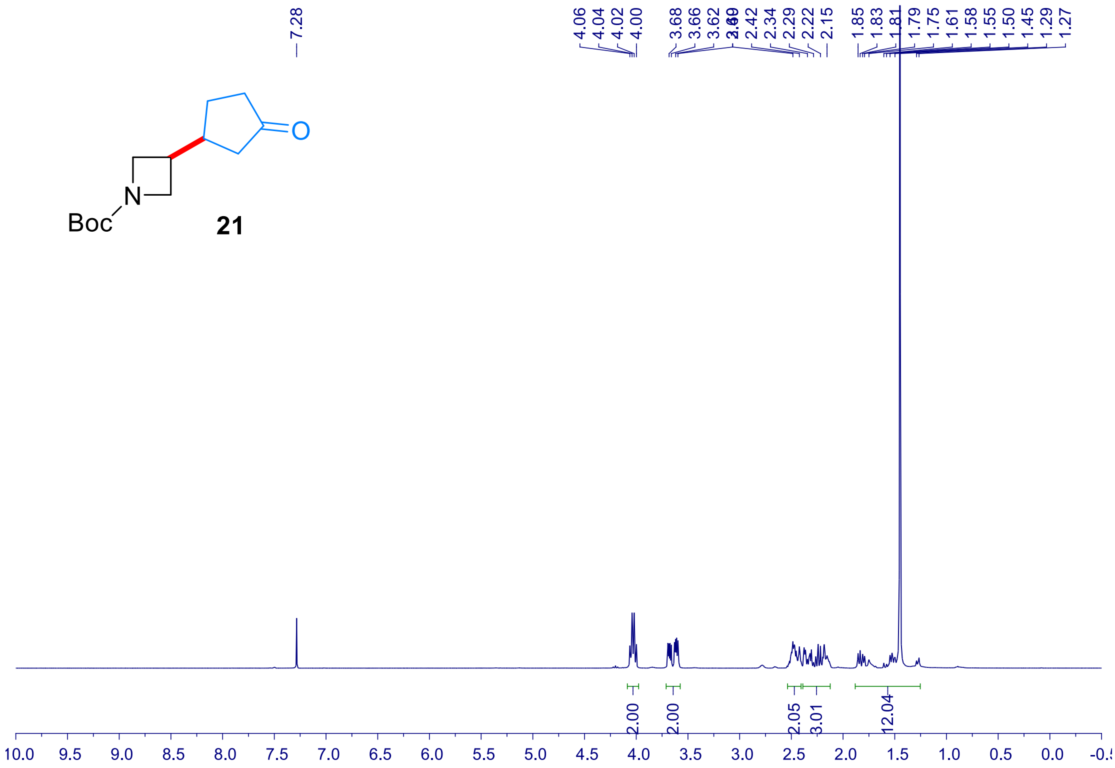

21

(21)-^{13}C NMR (101 MHz, $CDCl_3$)

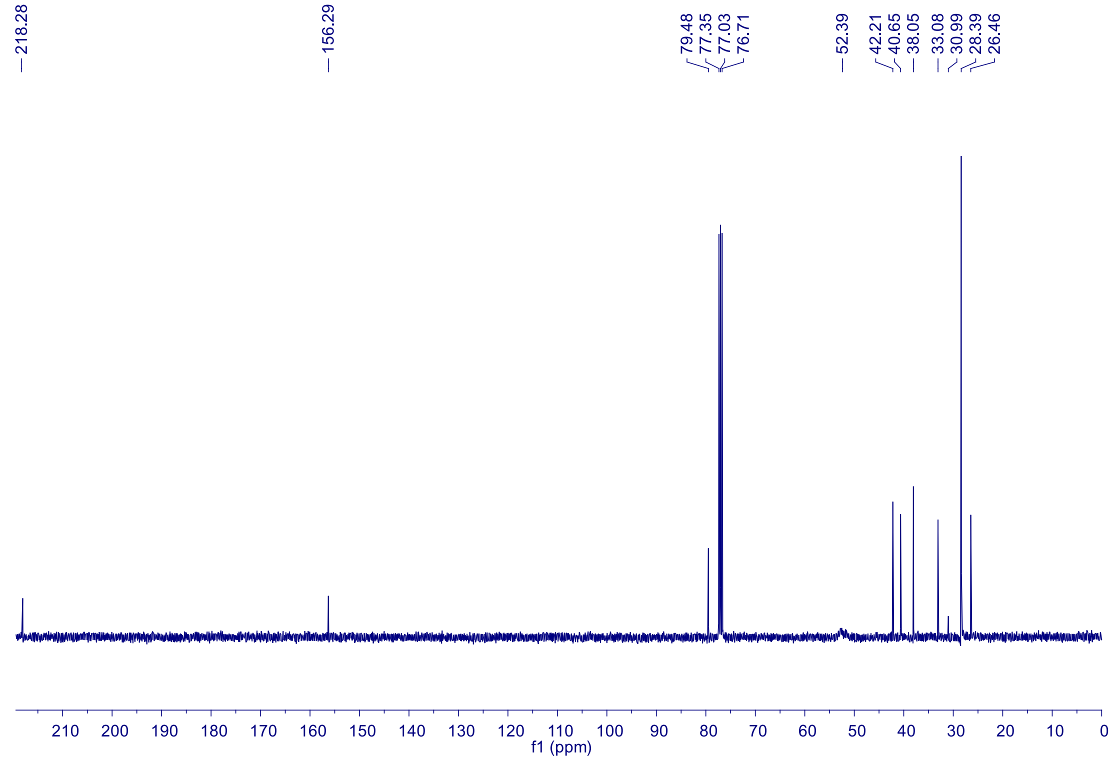

***tert*-butyl 3-(3-oxocyclohexyl)azetidine-1-carboxylate (22)**-^{1}H NMR (400 MHz, $CDCl_3$)

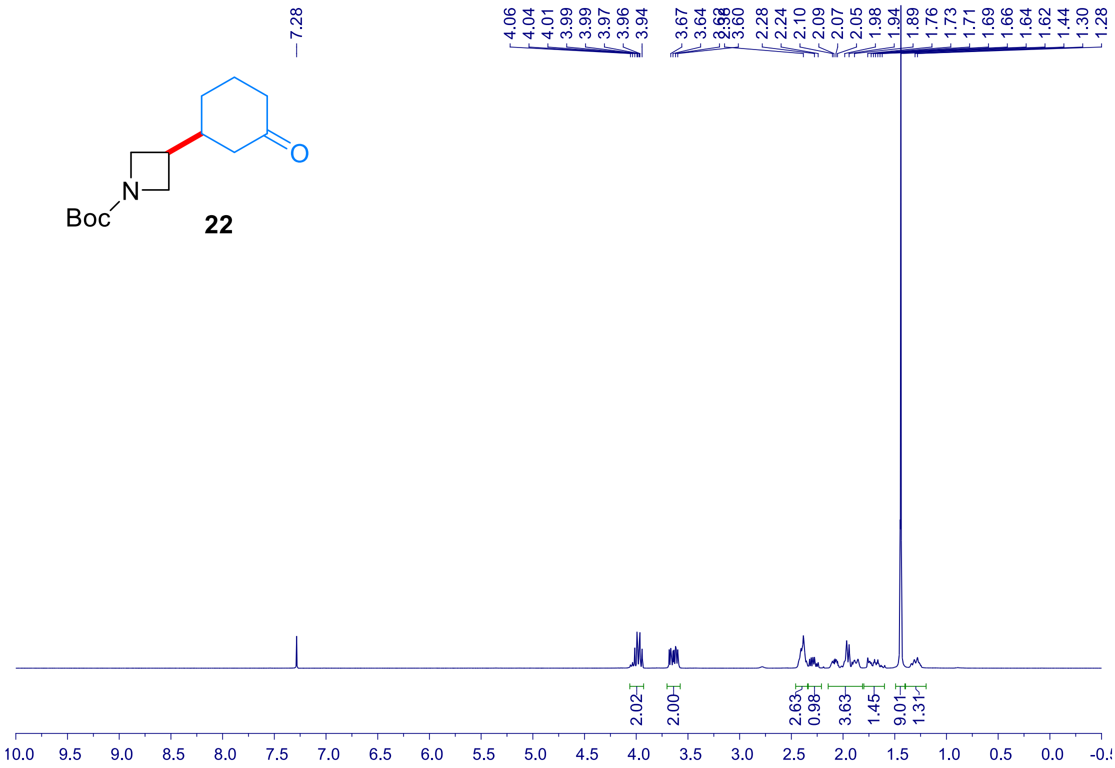

(22)-^{13}C NMR (101 MHz, $CDCl_3$)

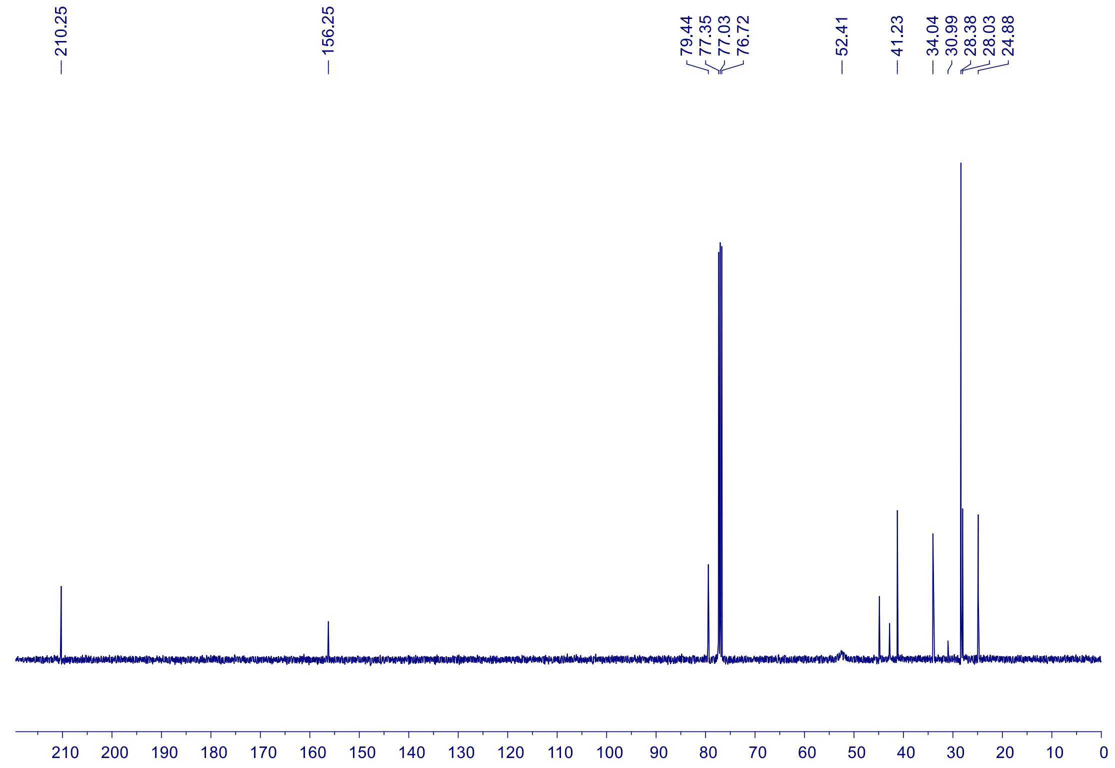

tert-Butyl 3-(2-(Bis(_tert_-butoxycarbonyl)amino)-3-methoxy-3-oxopropyl)azetidine -1-carboxylate (23)-^{1}H NMR (400 MHz, $CDCl_3$)

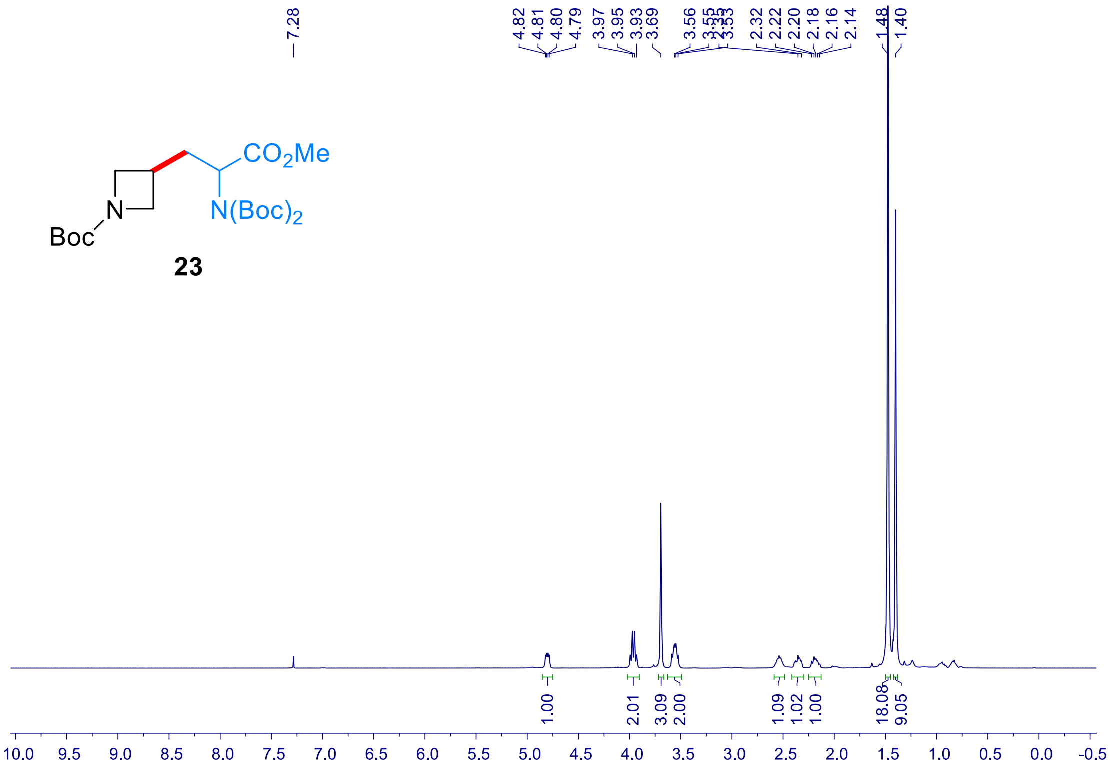

(23)-^{13}C NMR (101 MHz, $CDCl_3$)

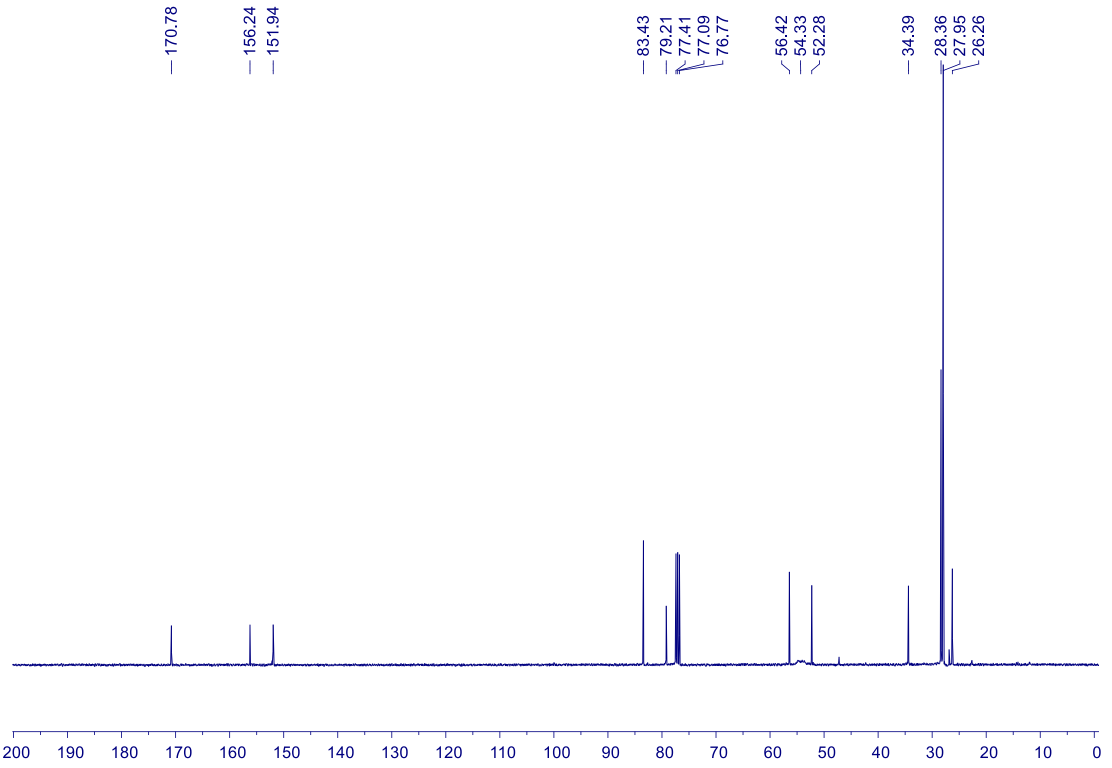

tert-Butyl 3-(2-(Bis(tert-butoxycarbonyl)amino)-3-methoxy-3-oxopropyl)pyrro lidine-1-carboxylate (24)-^{1}H NMR (400 MHz, $CDCl_3$)

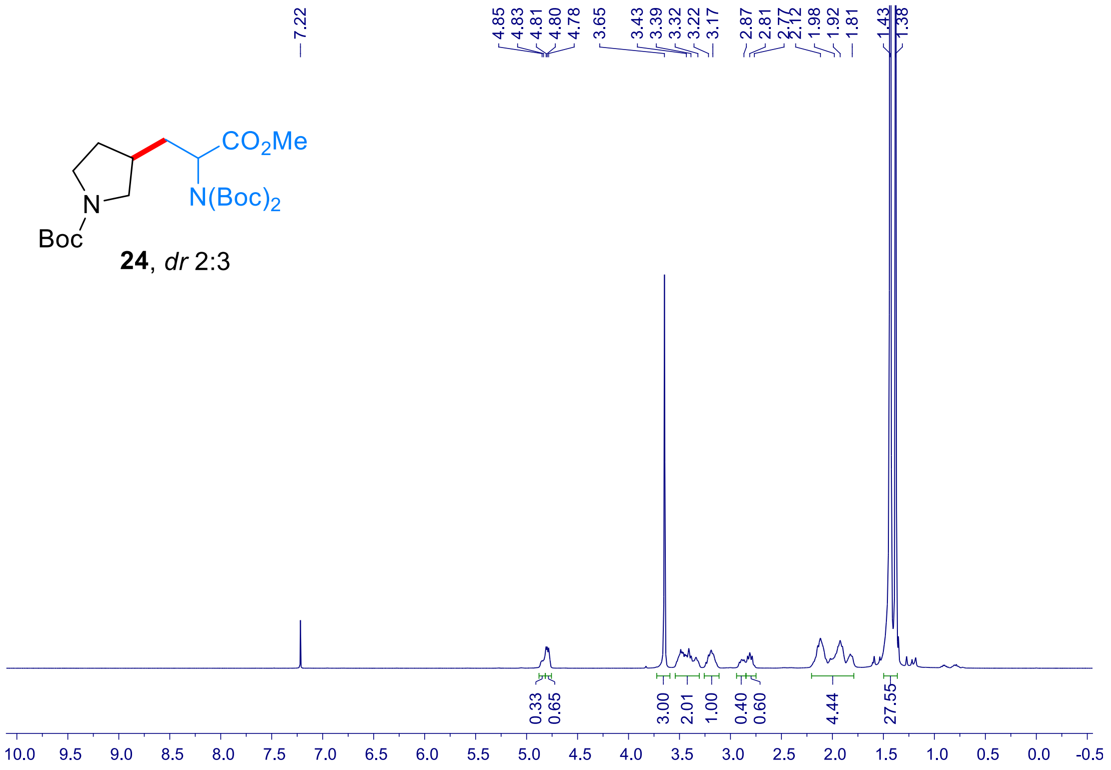

(24)-^{13}C NMR (101 MHz, $CDCl_3$)

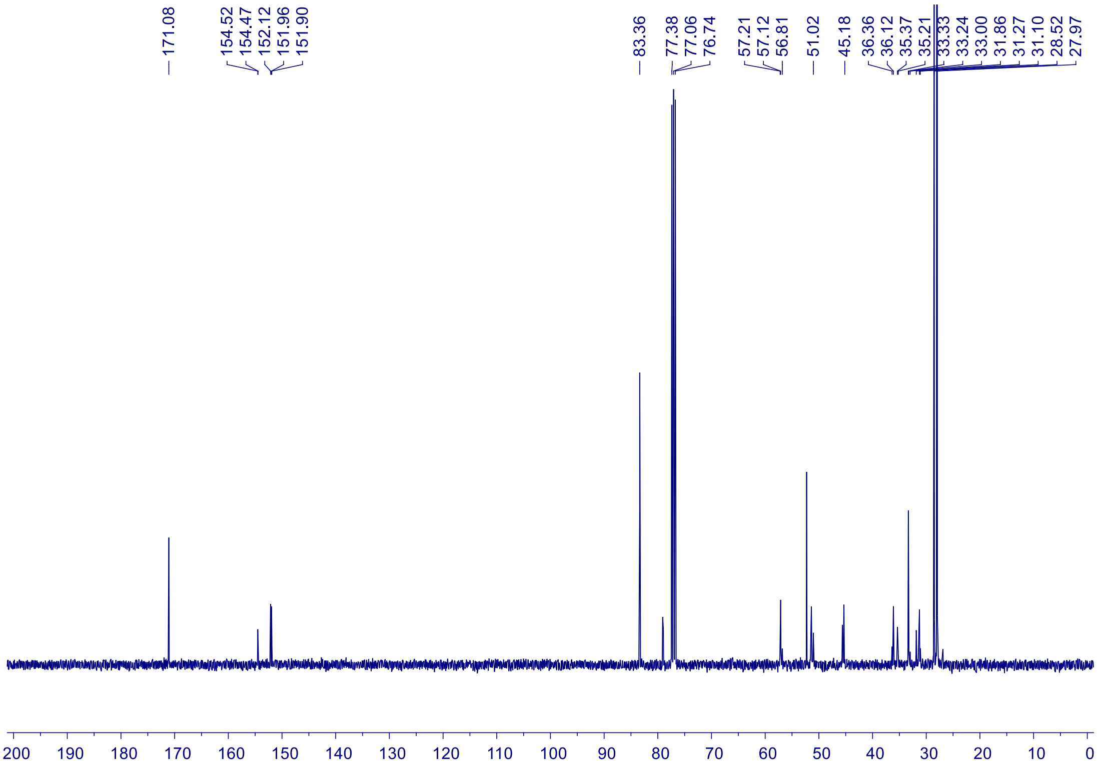

tert-Butyl 4-(2-(bis(_tert_-butoxycarbonyl)amino)-3-methoxy-3-oxopropyl) piperidine-1-carboxylate (25)-^{1}H NMR (400 MHz, $CDCl_3$)

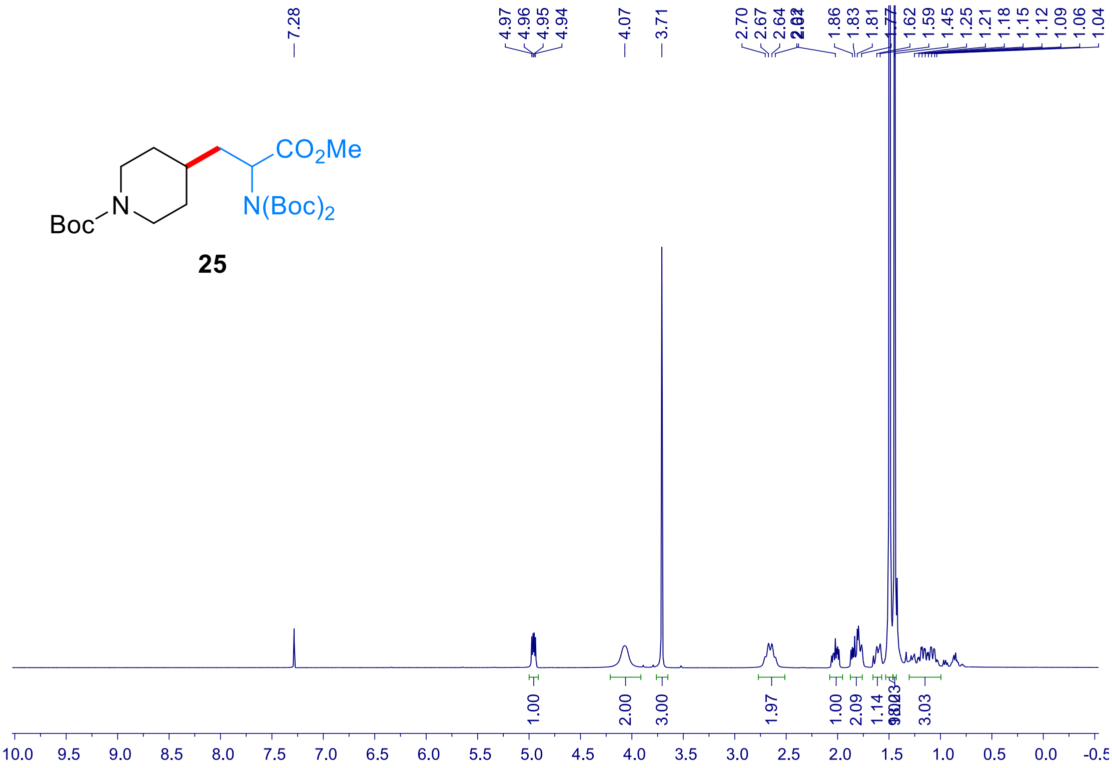

25

(25)-^{13}C NMR (101 MHz, $CDCl_3$)

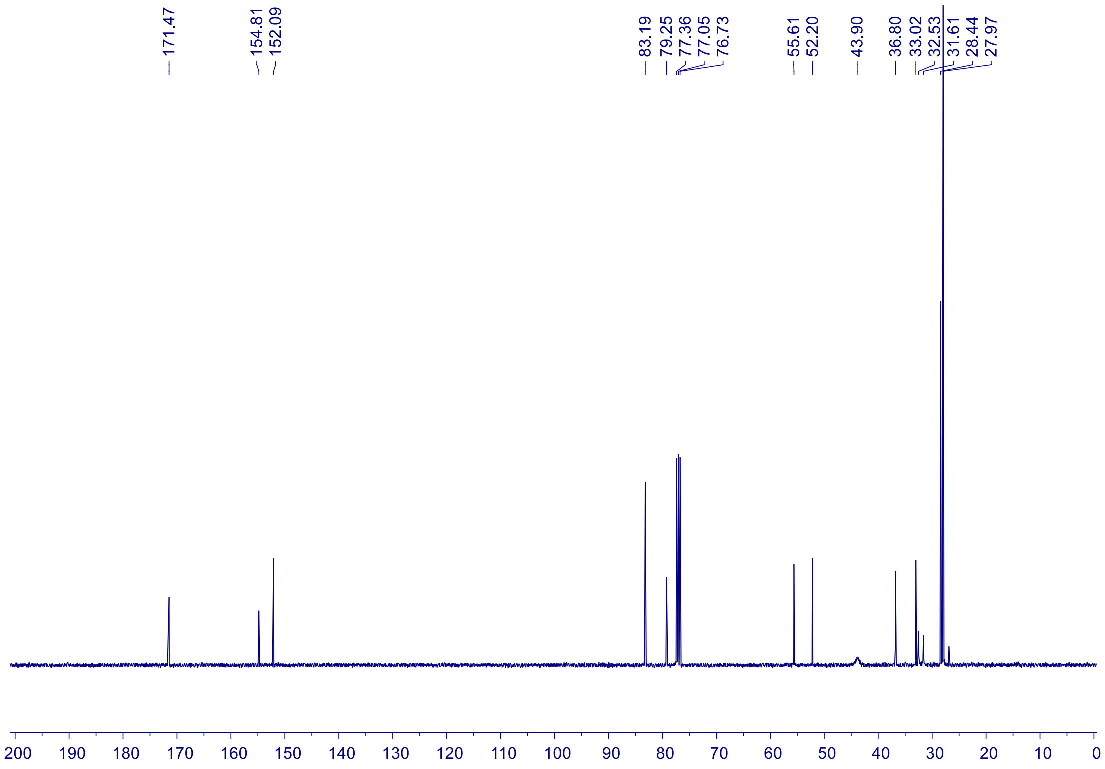

***tert*-Butyl 6-(2-(bis(tert-butoxycarbonyl)amino)-3-methoxy-3-oxopropyl) 2-azaspiro[3.3]heptane-2-carboxylate (26)**-^{1}H NMR (400 MHz, $CDCl_3$)

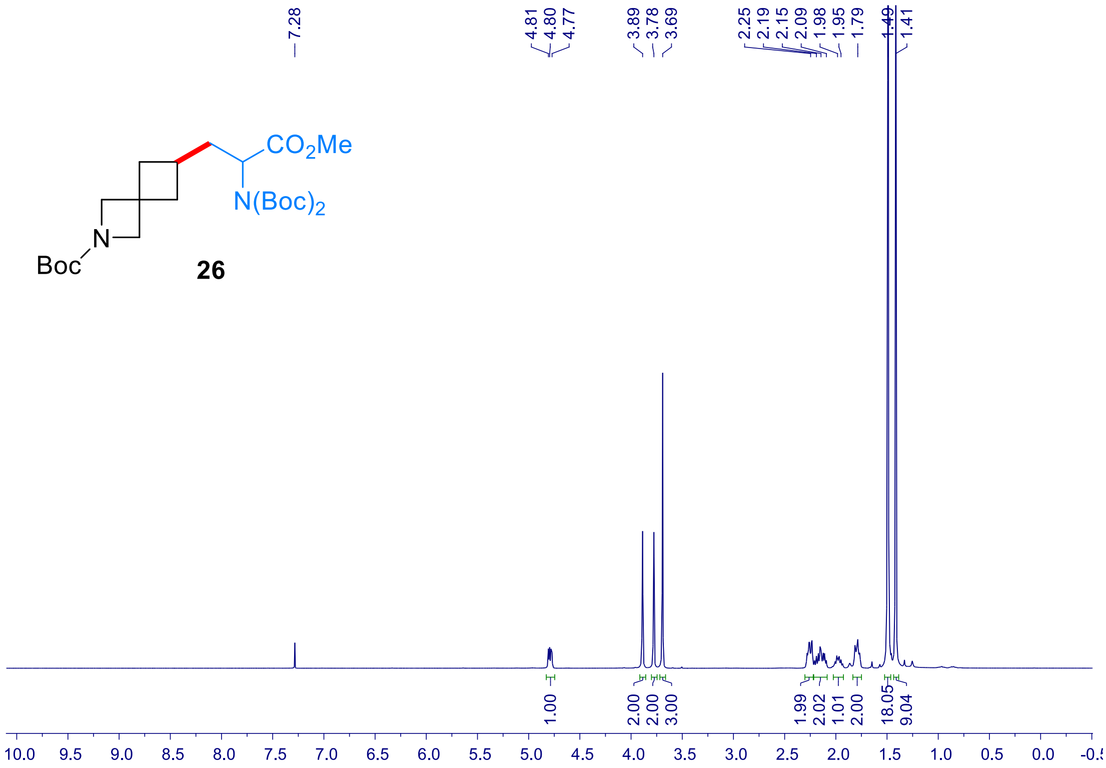

(26)-^{13}C NMR (101 MHz, $CDCl_3$)

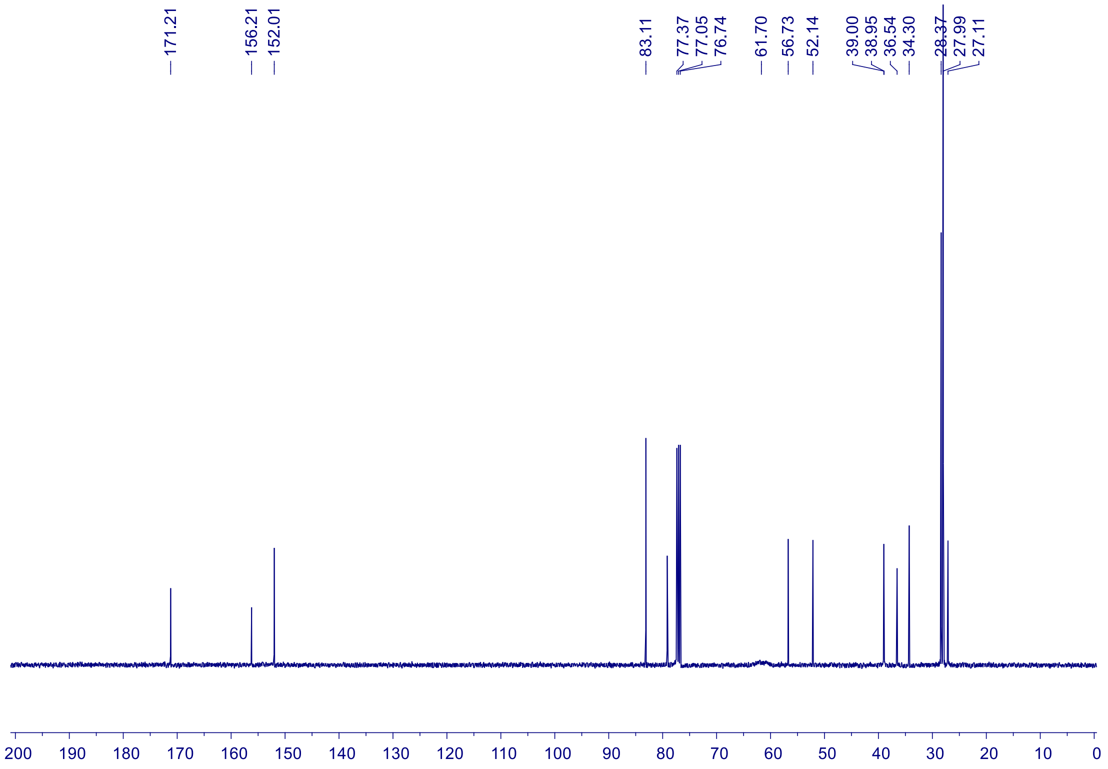

tert-Butyl 2-(2-(bis(tert-butoxycarbonyl)amino)-3-methoxy-3-oxopropyl) 7-azaspiro[3.5]nonane-7-carboxylate (27)-^{1}H NMR (400 MHz, $CDCl_3$)

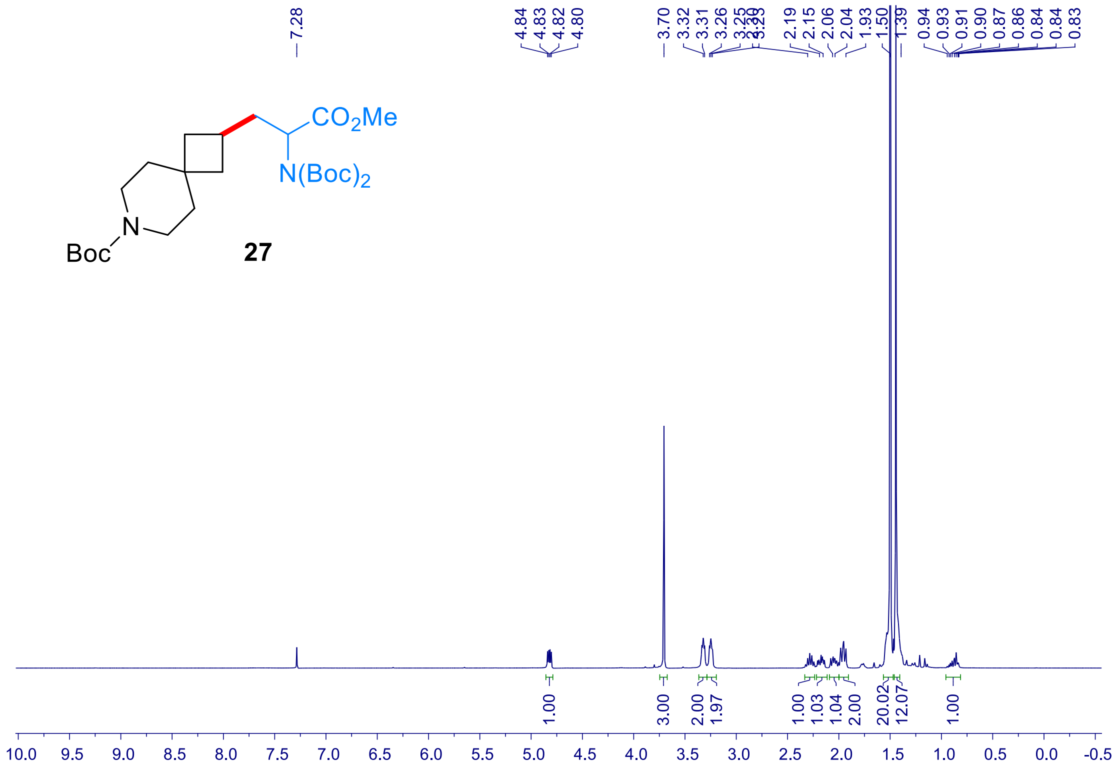

(27)-^{13}C NMR (101 MHz, $CDCl_3$)

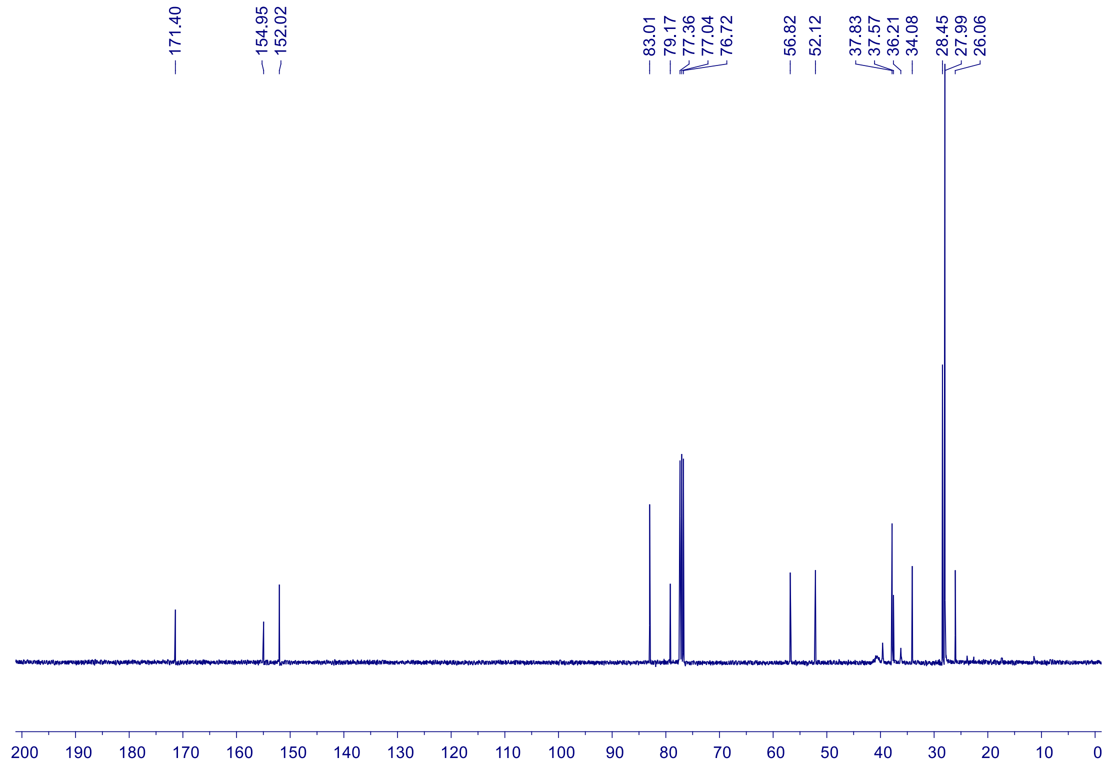

***tert*-Butyl 4-(2-(Bis(tert-butoxycarbonyl)amino)-3-methoxy-3-oxopropyl)azepane -1-carboxylate (28)**-^{1}H NMR (400 MHz, $CDCl_3$)

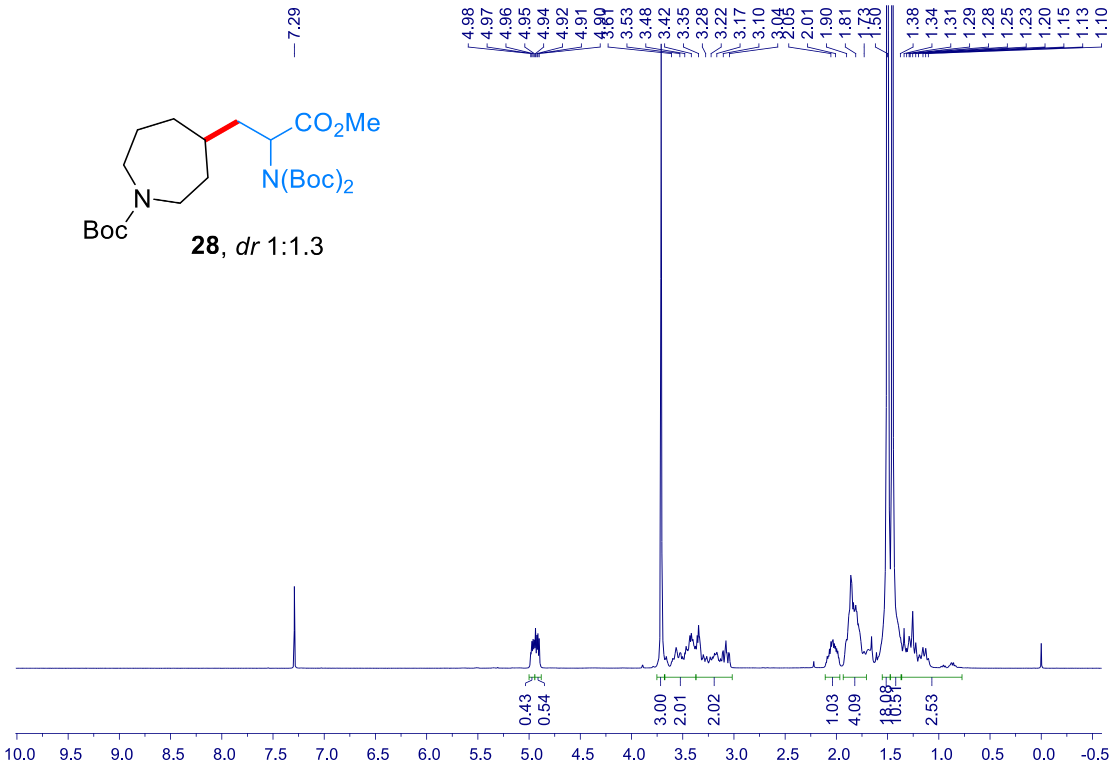

(28)-^{13}C NMR (101 MHz, $CDCl_3$)

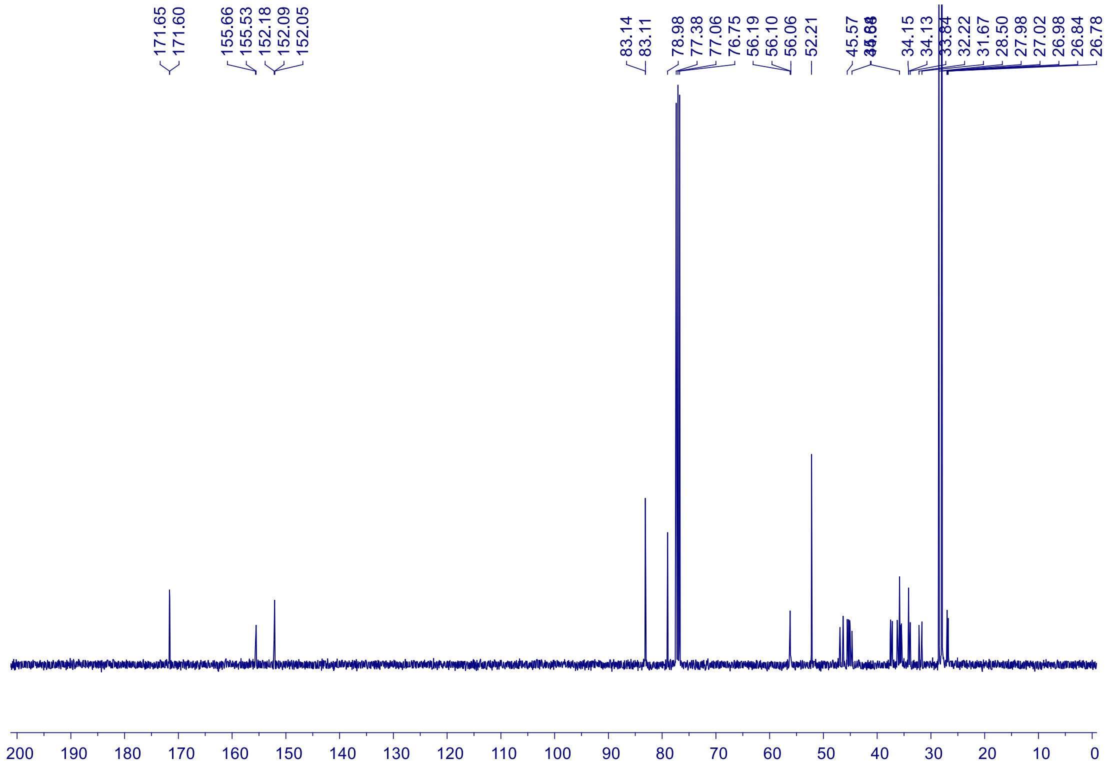

***tert*-butyl 3-(2-(bis(tert-butoxycarbonyl)amino)-3-methoxy-3-oxopropyl)-8-azabi cyclo[3.2.1]octane-8-carboxylate (29)**-^{1}H NMR (400 MHz, $CDCl_3$)

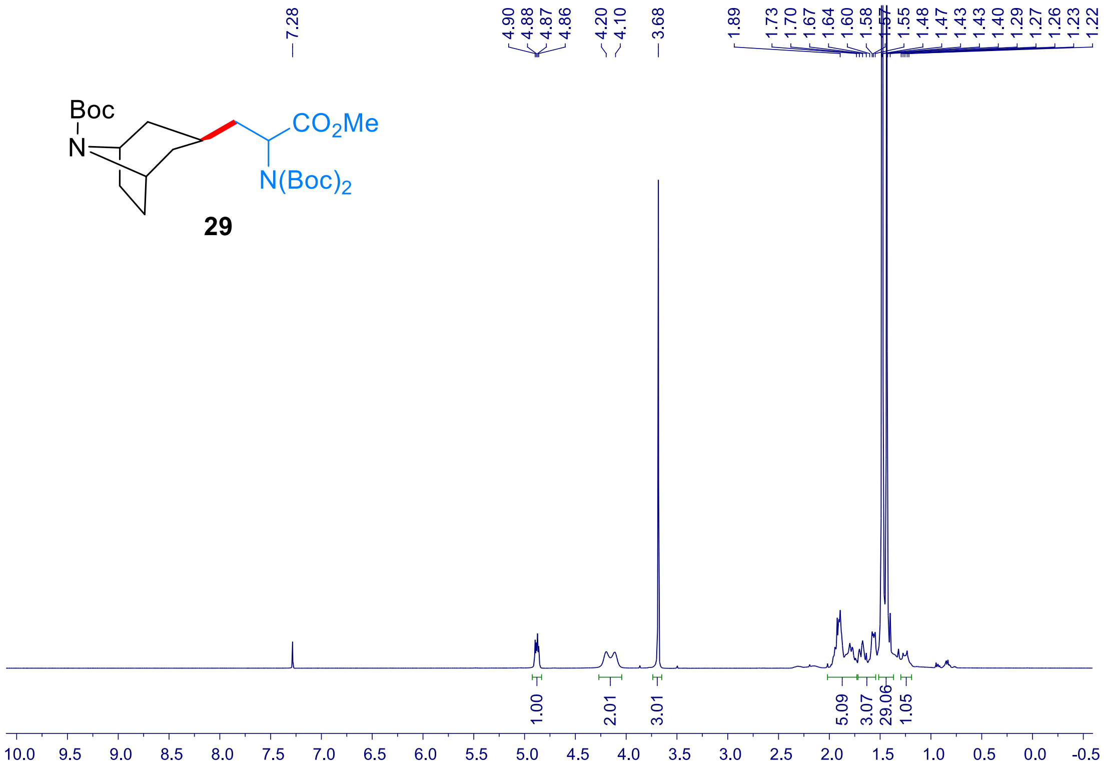

(29)-^{13}C NMR (101 MHz, $CDCl_3$)

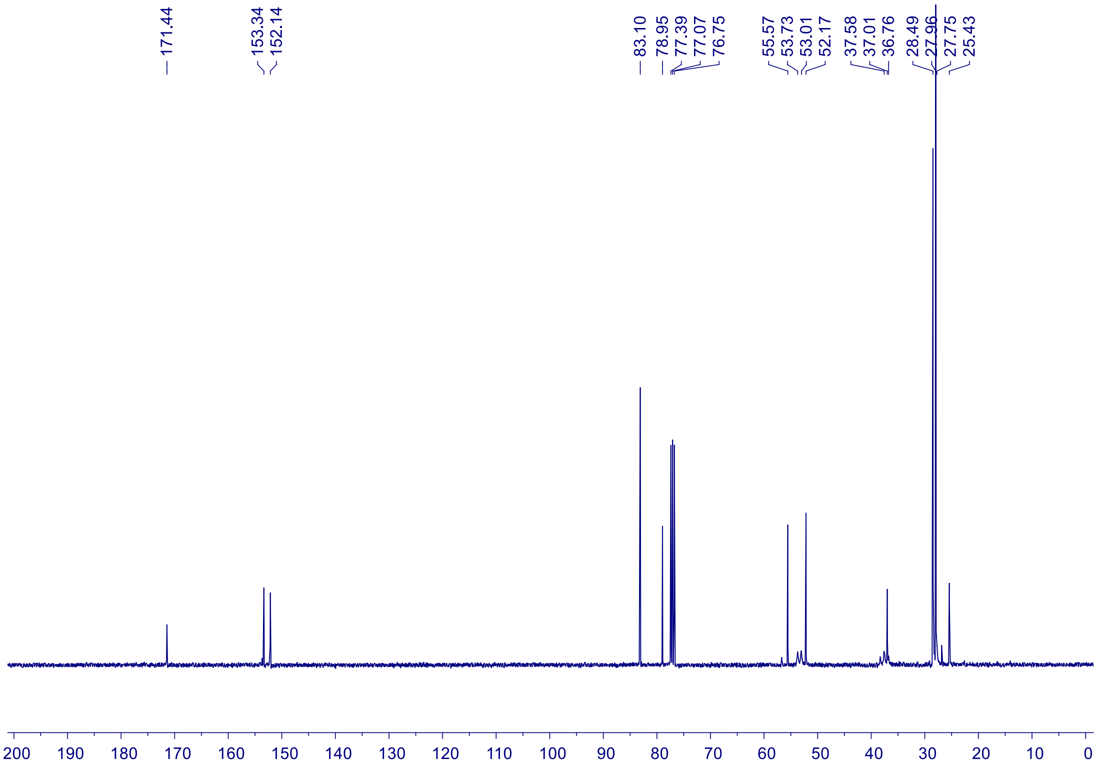

1-(tert-butyl)-2-methyl 4-(2-(Bis(tert-butoxycarbonyl)amino)-3-methoxy -3-oxopropyl)pyrrolidine-1,2-dicarboxylate (30)-^{1}H NMR (400 MHz, $CDCl_3$)

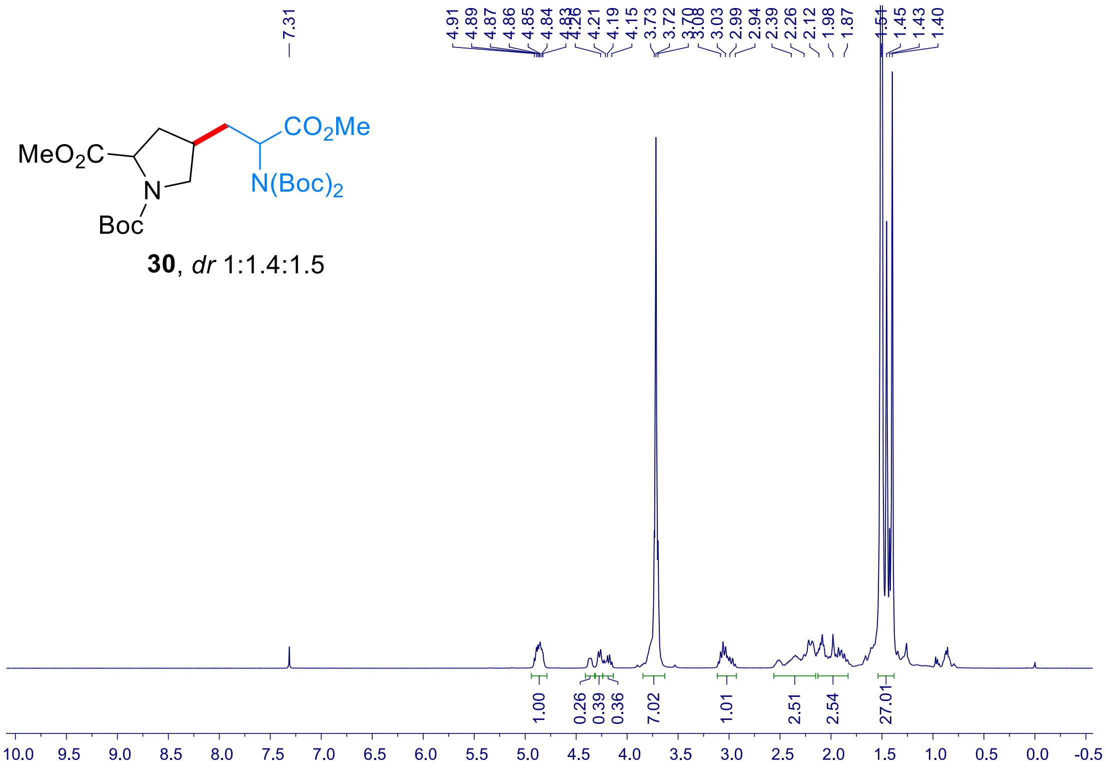

(30)-^{13}C NMR (101 MHz, $CDCl_3$)

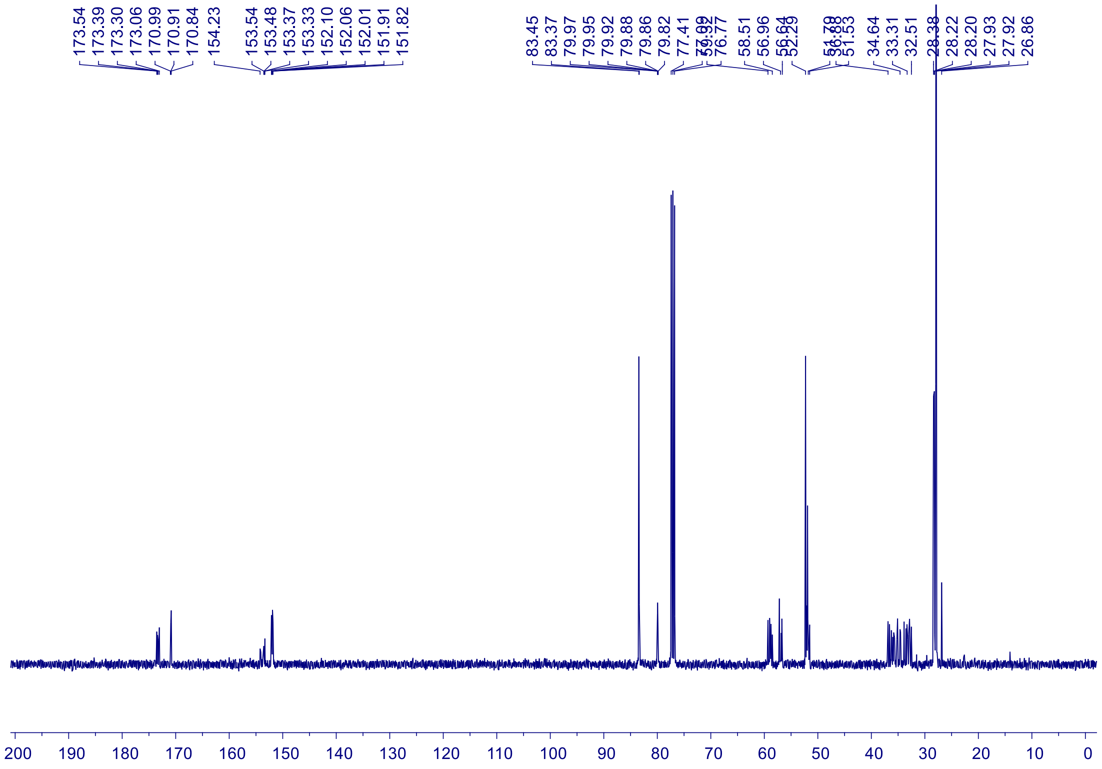

2-(4-(2-(bis(tert-butoxycarbonyl)amino)-3-methoxy-3-oxopropyl)piperidin-1-yl)pyrimidine (31)-^{1}H NMR (400 MHz, $CDCl_3$)

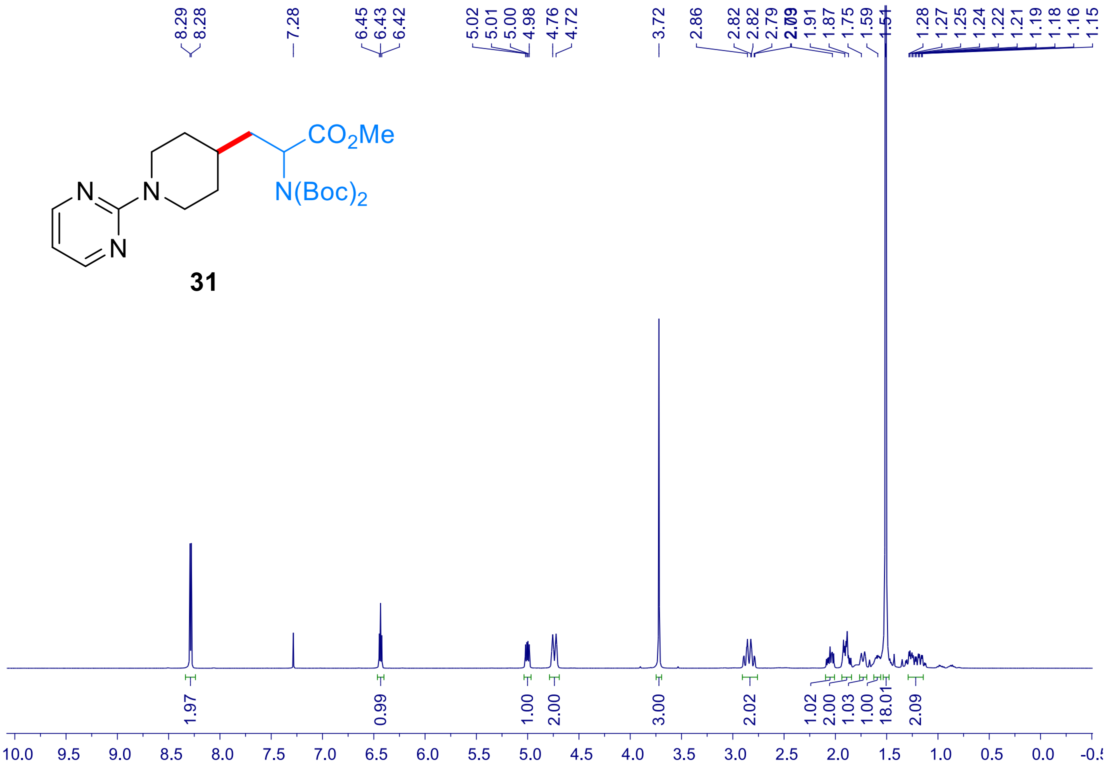

(31)-^{13}C NMR (101 MHz, $CDCl_3$)

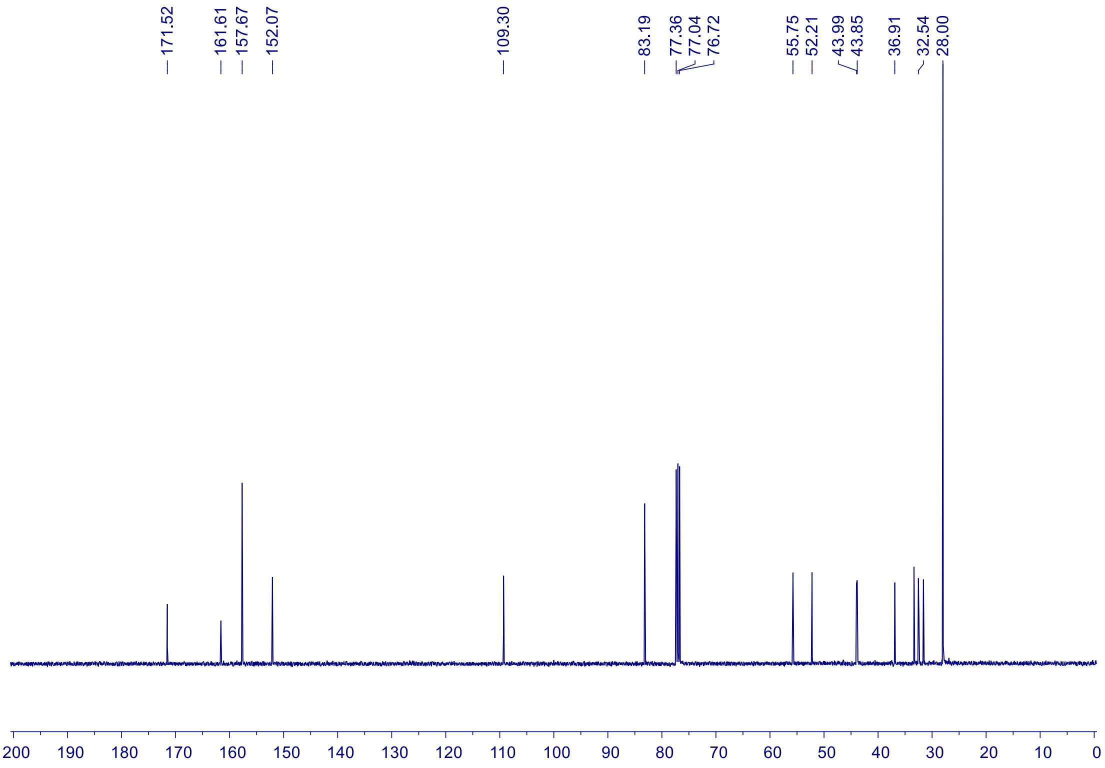

Methyl 2-(Bis(*tert*-butoxycarbonyl)amino)-3-(cyclopentane-4-yl)propanoate (32) -^{1}H NMR (400 MHz, $CDCl_3$)

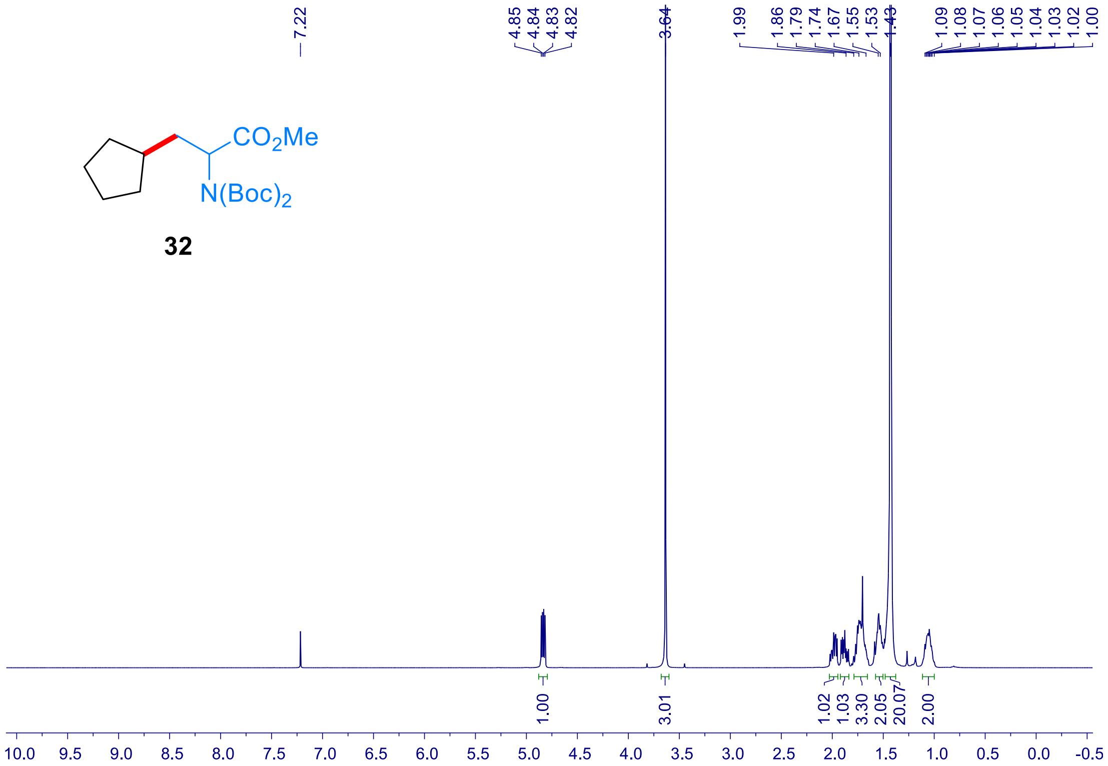

(32)-^{13}C NMR (101 MHz, $CDCl_3$)

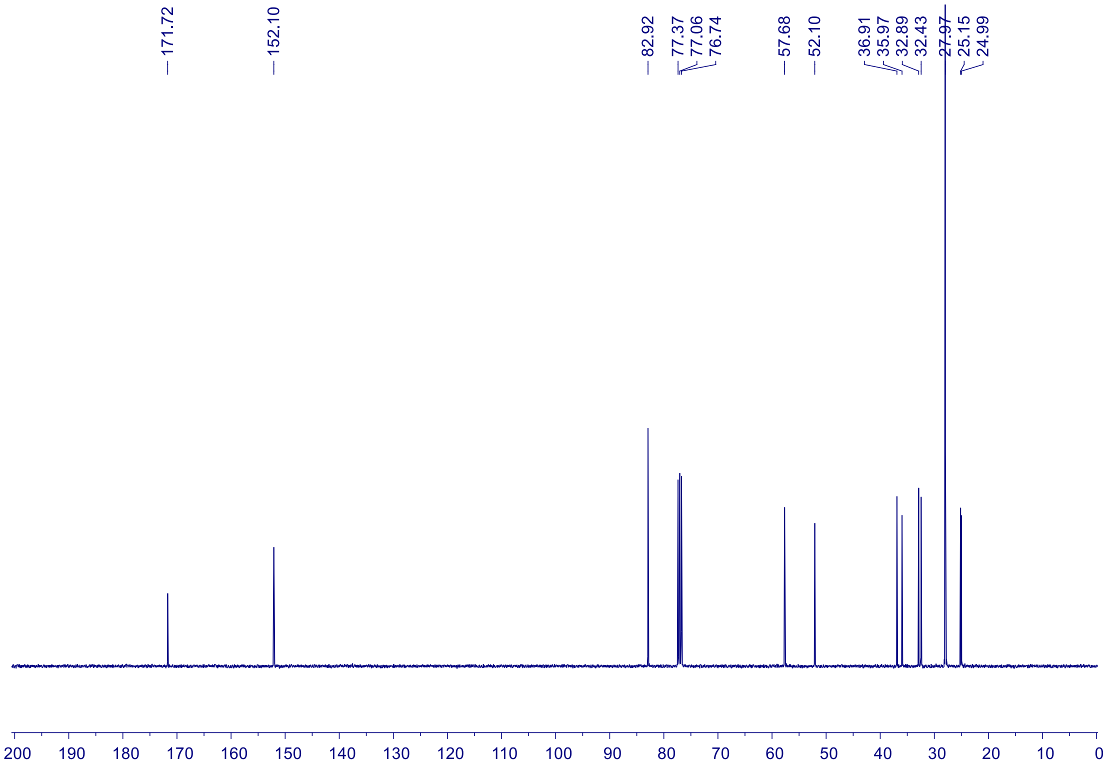

Methyl 2-(Bis(*tert*-butoxycarbonyl)amino)-3-(cyclohexane-4-yl)propanoate (33)-^{1}H NMR (400 MHz, $CDCl_3$)

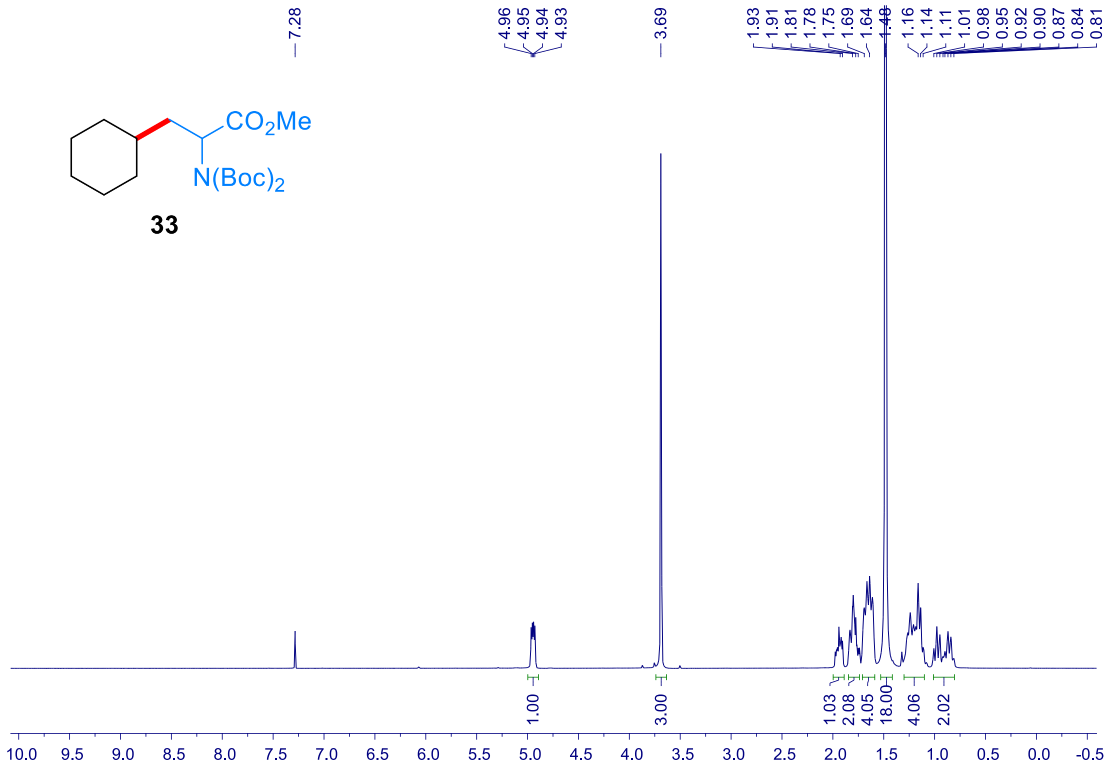

(33)-^{13}C NMR (101 MHz, $CDCl_3$)

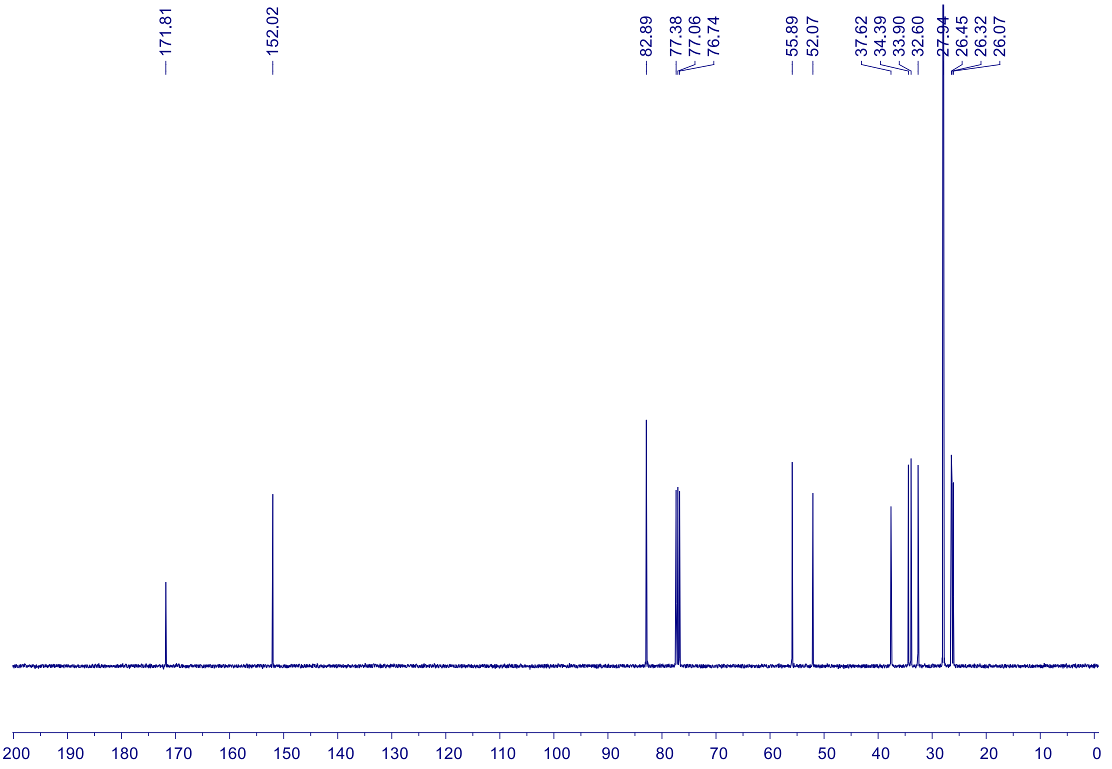

Methyl 2-(bis(tert-butoxycarbonyl)amino)-3-((*2S,5R*)-2-isopropyl-5-methyl cyclohexyl)propanoate (34)-^{1}H NMR (400 MHz, $CDCl_3$)

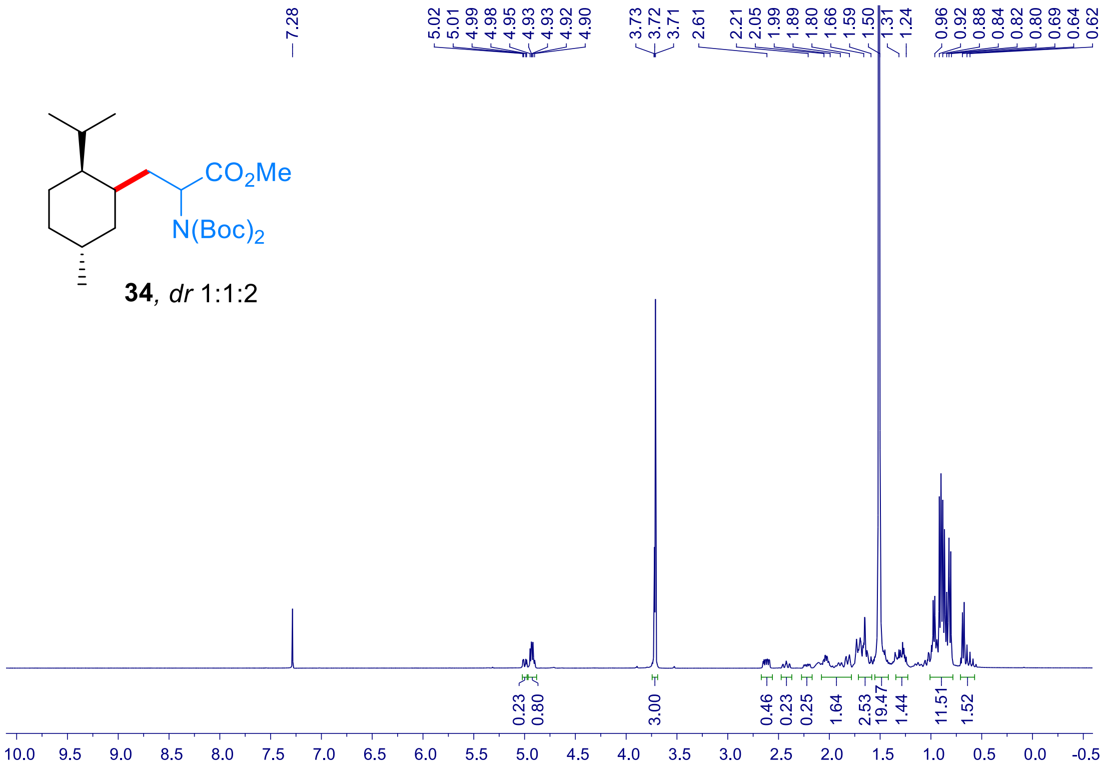

(34)-^{13}C NMR (101 MHz, $CDCl_3$)

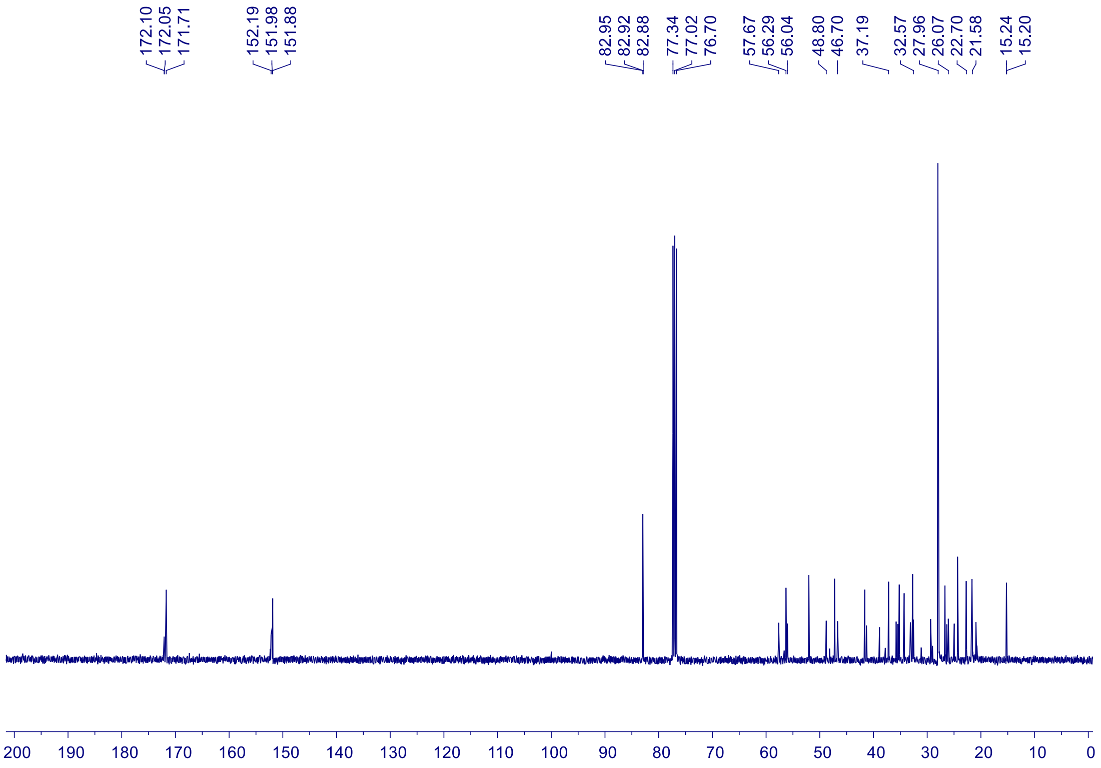

Methyl 2-(Bis(*tert*-butoxycarbonyl)amino)-3-(cycloheptane-4-yl)propanoate (35)-^{1}H NMR (400 MHz, $CDCl_3$)

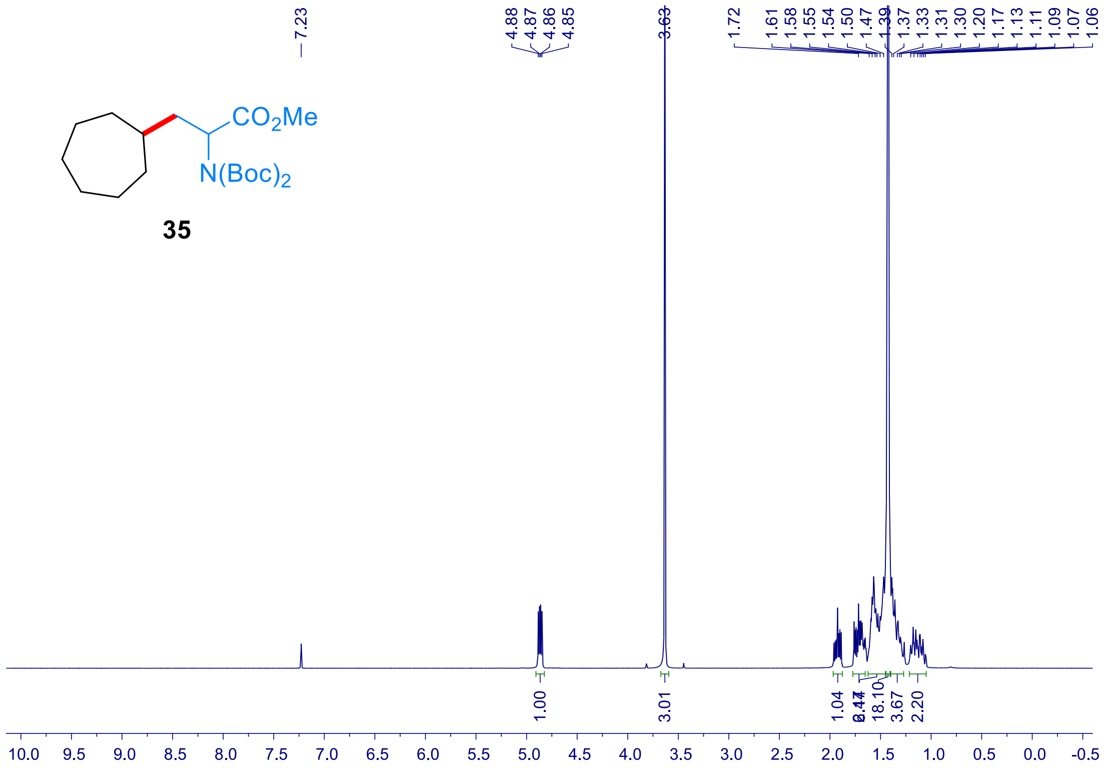

(35)-^{13}C NMR (101 MHz, $CDCl_3$)

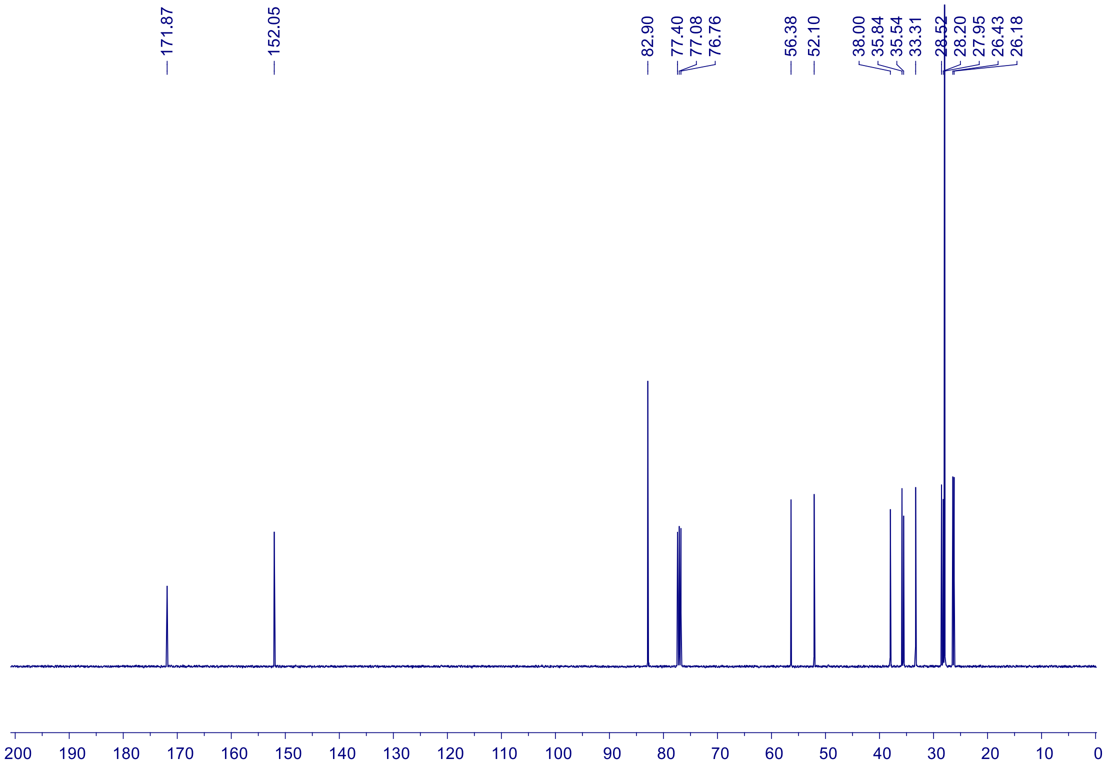

Methyl 2-(Bis(*tert*-butoxycarbonyl)amino)-3-(cyclododecane-4-yl)propanoate (36) -^{1}H NMR (400 MHz, $CDCl_3$)

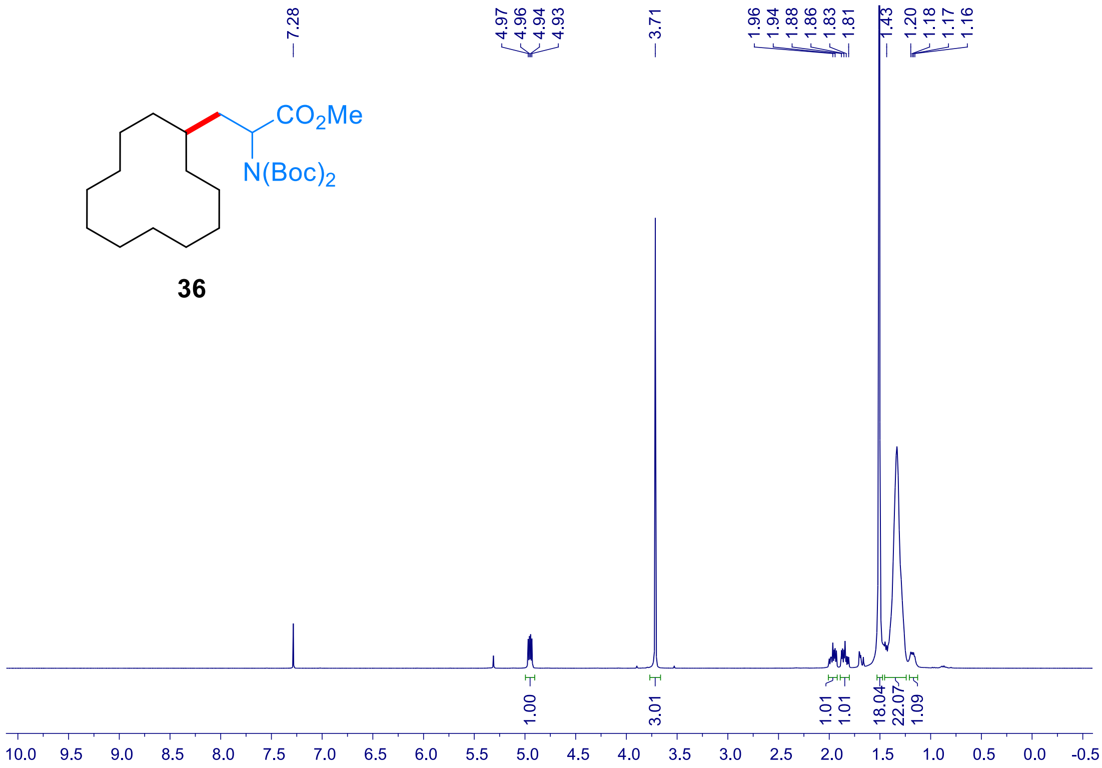

(36)-^{13}C NMR (101 MHz, $CDCl_3$)

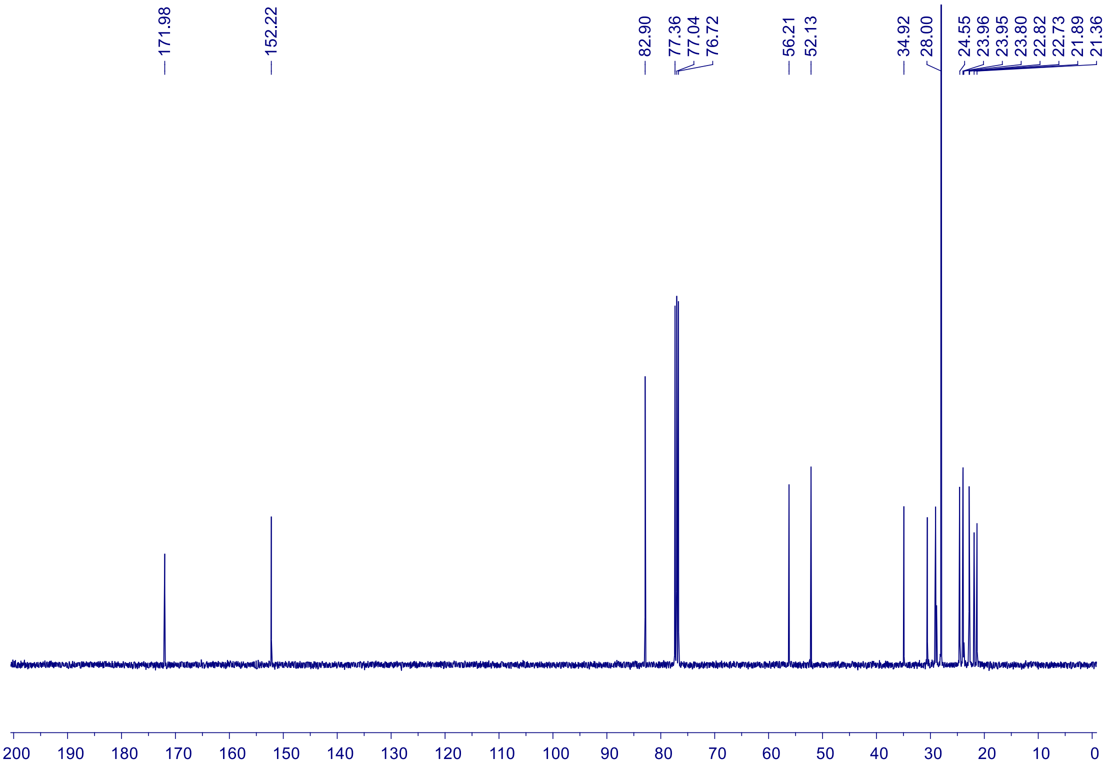

Methyl 2-(Bis(*tert*-butoxycarbonyl)amino)-3-(tetrahydro-2H-pyran-4-yl) propanoate (37)-^{1}H NMR (400 MHz, $CDCl_3$)

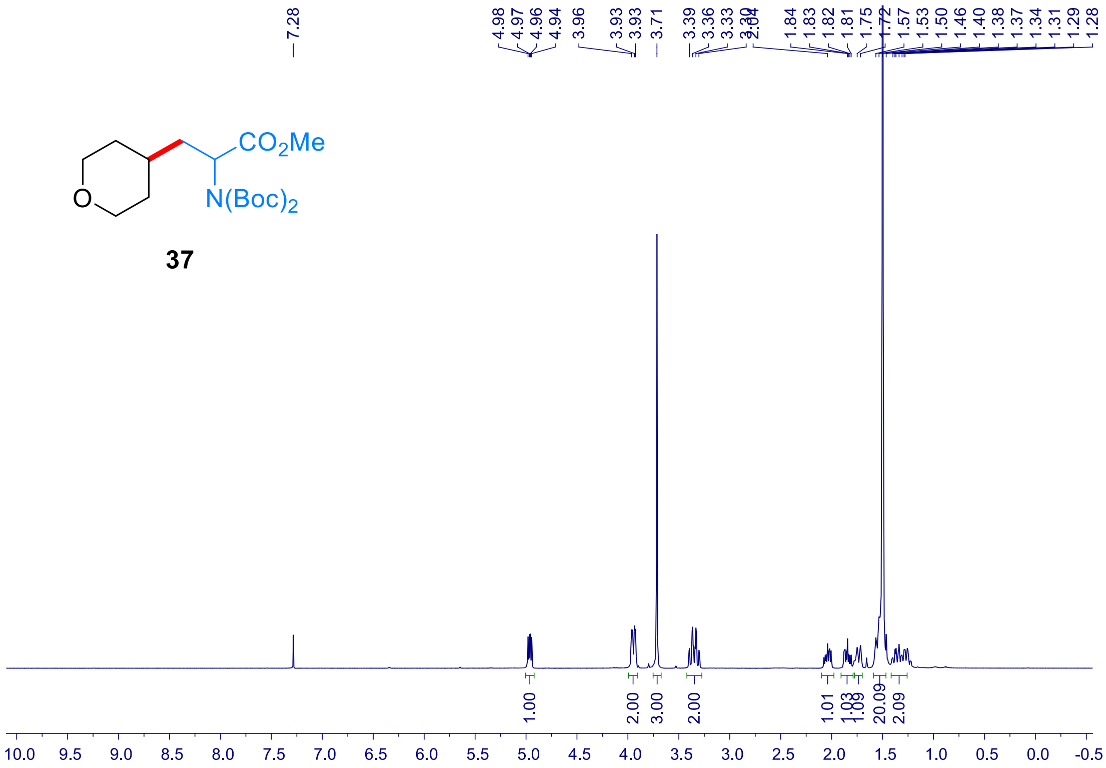

(37)-^{13}C NMR (101 MHz, $CDCl_3$)

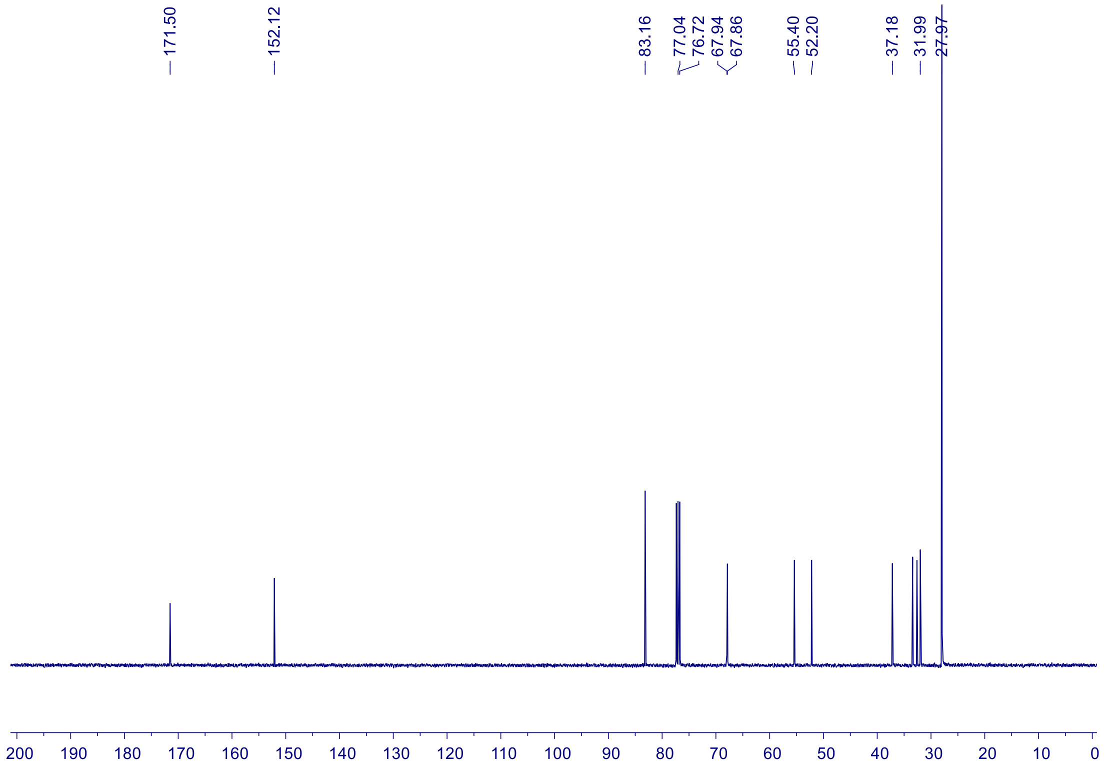

Methyl 2-(Bis(*tert*-butoxycarbonyl)amino)-3-(tetrahydro-2H-thiopyran-4-yl) propanoate (38)-^{1}H NMR (400 MHz, $CDCl_3$)

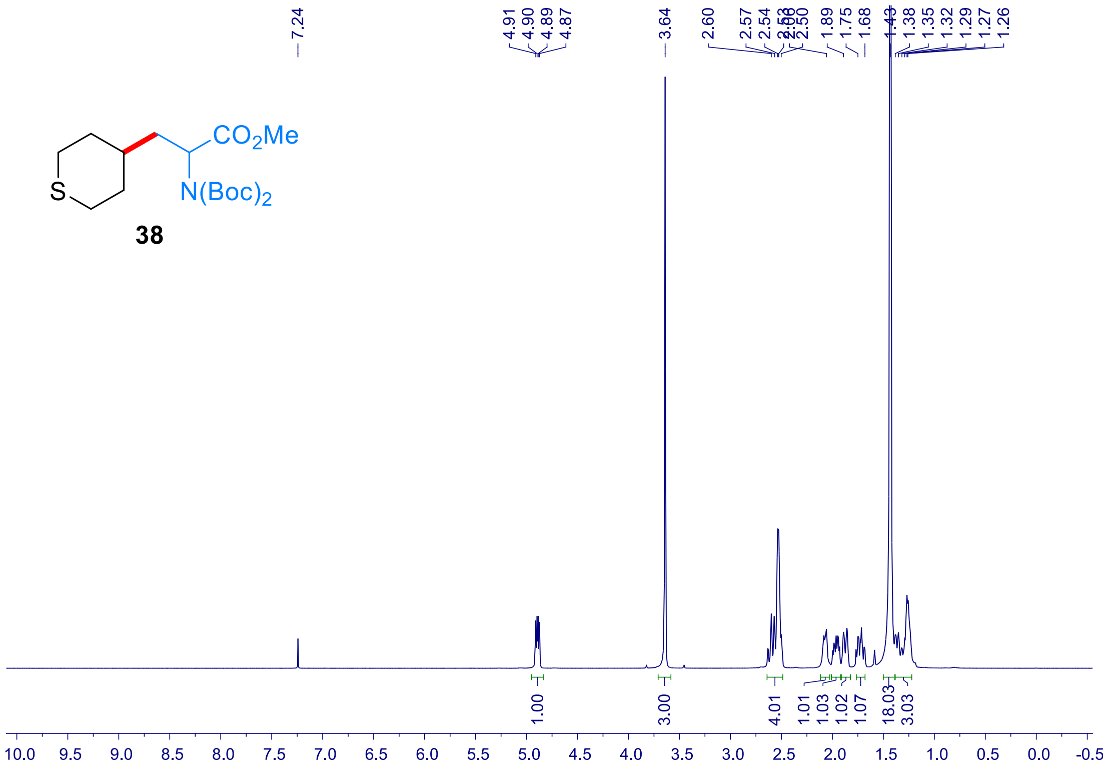

(38)-^{13}C NMR (101 MHz, $CDCl_3$)

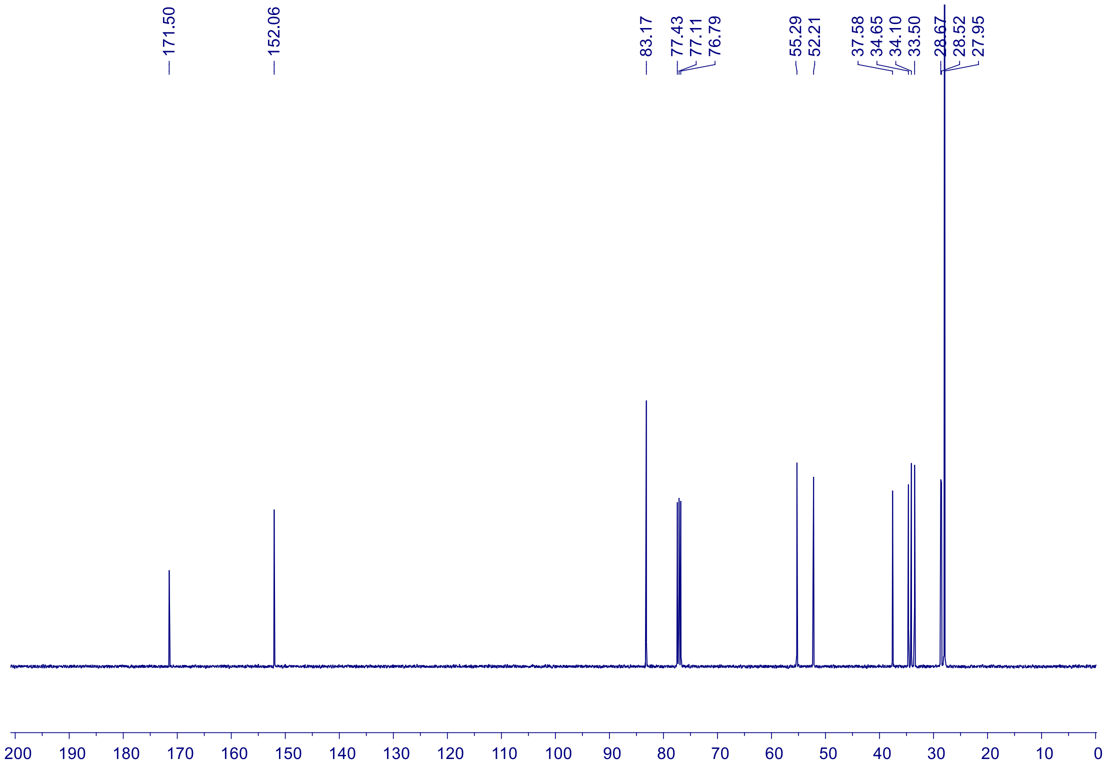

Methyl 2-(Bis(*tert*-butoxycarbonyl)amino)-3-(oxetan-3-yl)propanoate (39)-^{1}H NMR (400 MHz, $CDCl_3$)

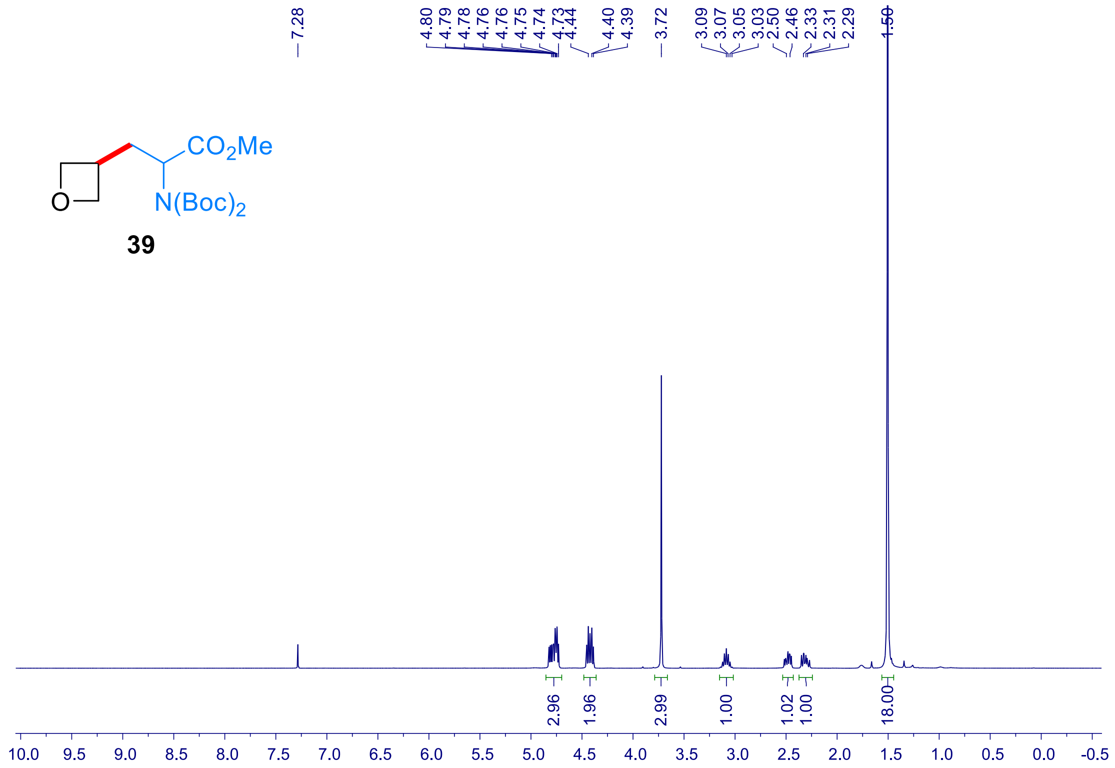

(39)-^{13}C NMR (101 MHz, $CDCl_3$)

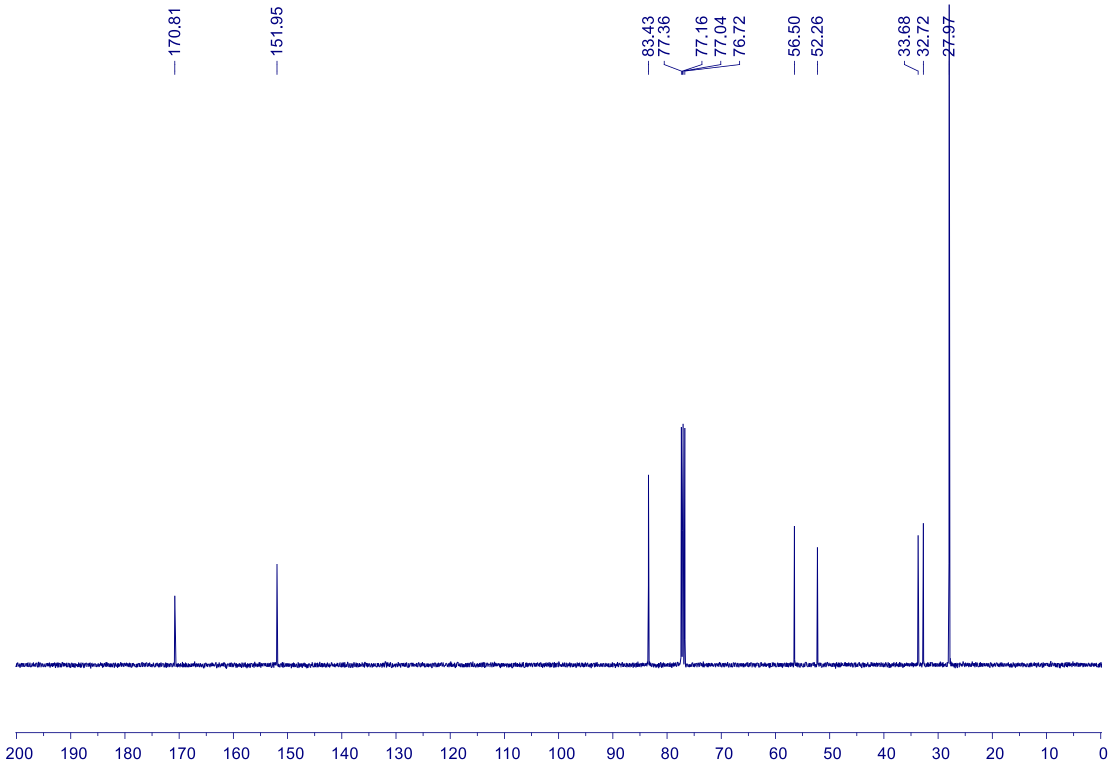

Methyl 2-(Bis(*tert*-butoxycarbonyl)amino)-3-[(diisopropyl cyclobutane-1,1-dicarboxylate)-3-yl]propanoate (40)-^{1}H NMR (400 MHz, $CDCl_3$)

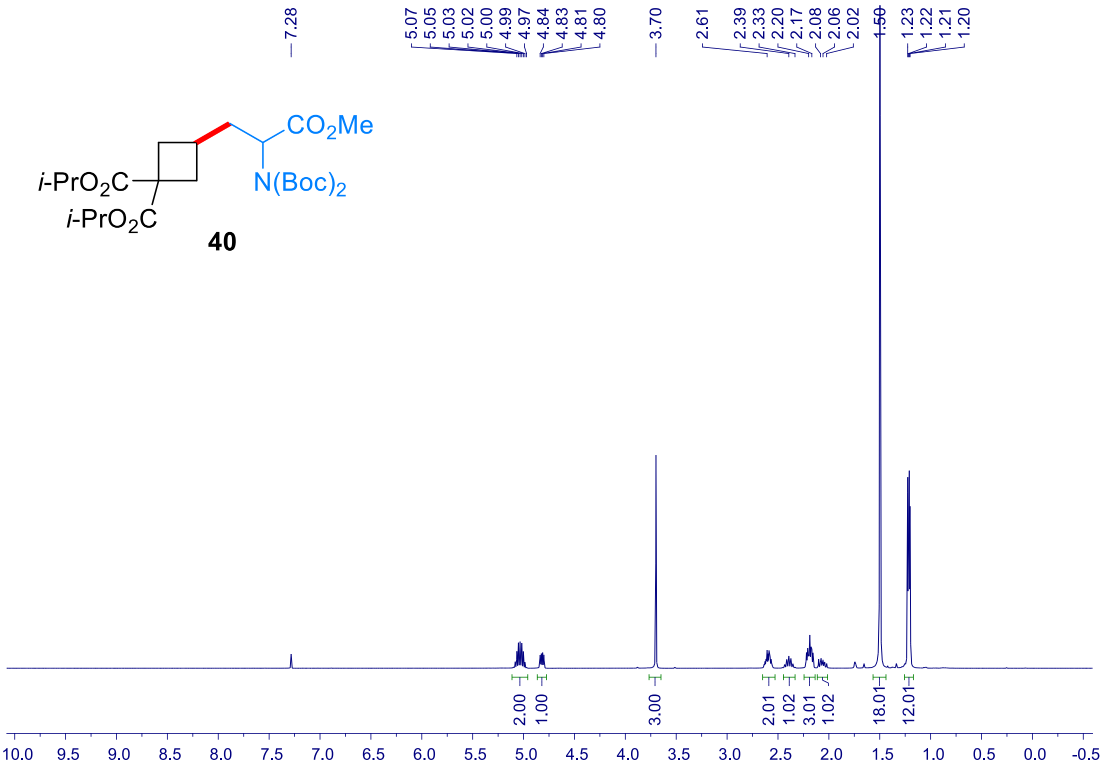

(40)-^{13}C NMR (101 MHz, $CDCl_3$)

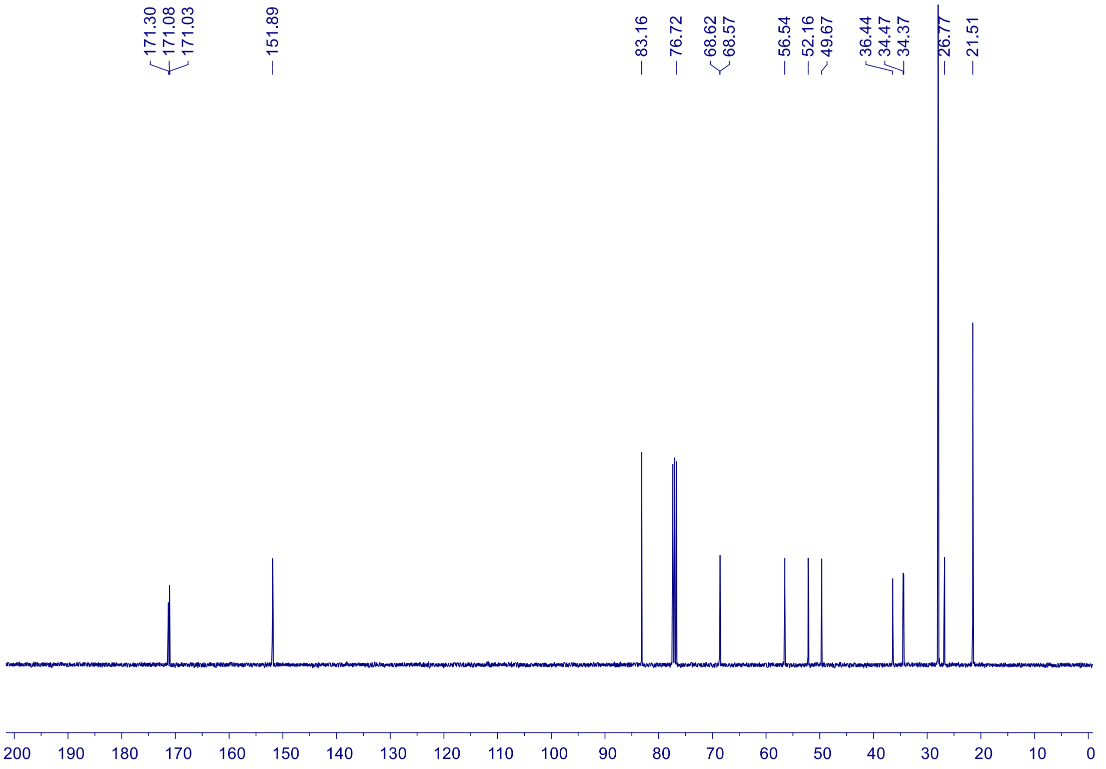

Methyl 2-(Bis(*tert*-butoxycarbonyl)amino)-3-[(1,4-dioxaspiro[4.5]decane)-8-yl] propanoate (41)-^{1}H NMR (400 MHz, $CDCl_3$)

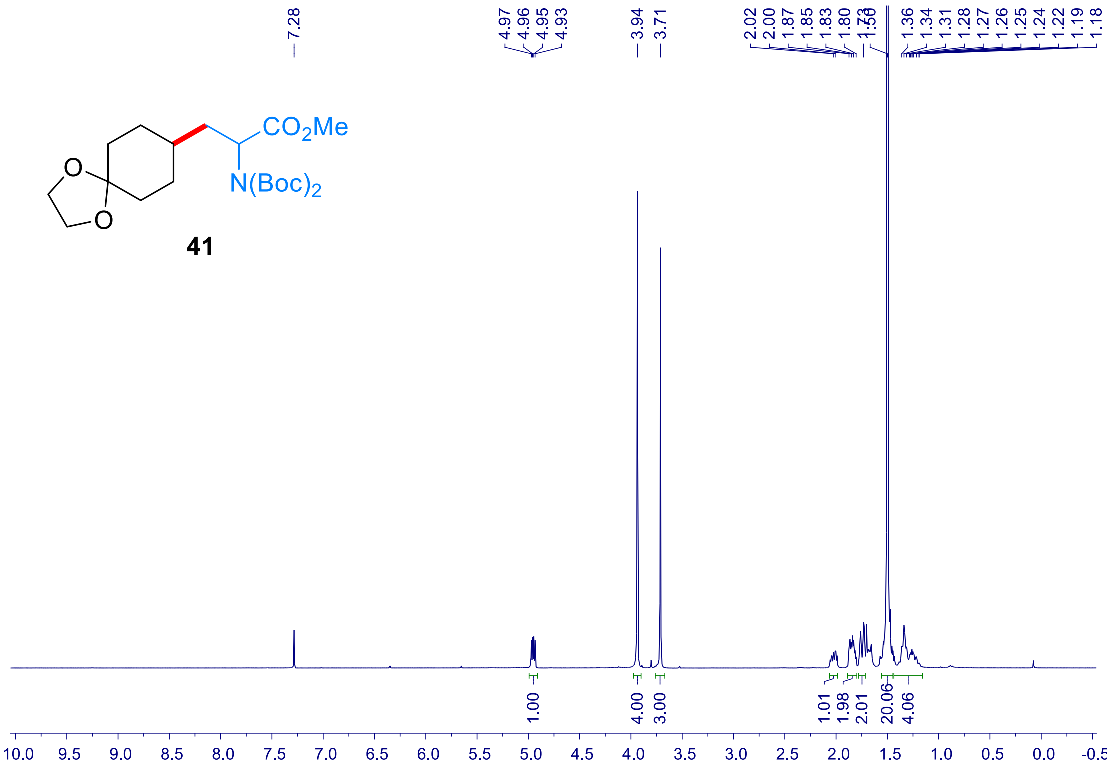

(41)-^{13}C NMR (101 MHz, $CDCl_3$)

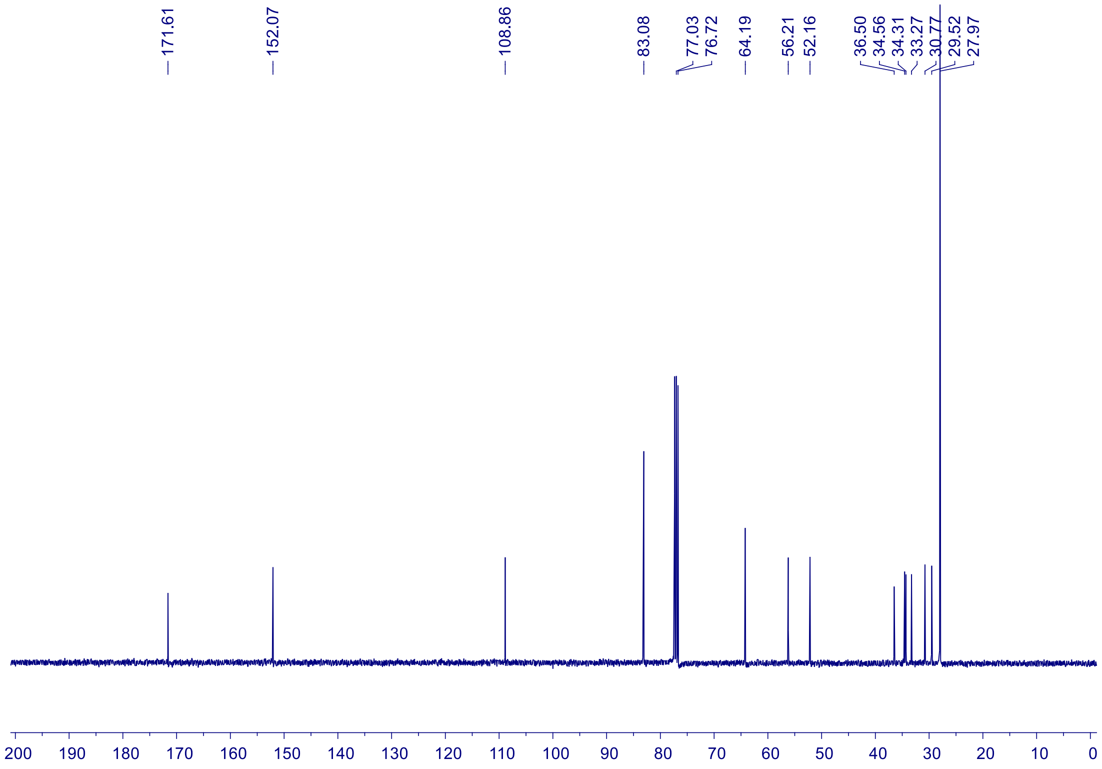

Methyl 2-(Bis(*tert*-butoxycarbonyl)amino)-3-(methoxycyclohexane-2-yl) propanoate (42)-^{1}H NMR (400 MHz, $CDCl_3$)

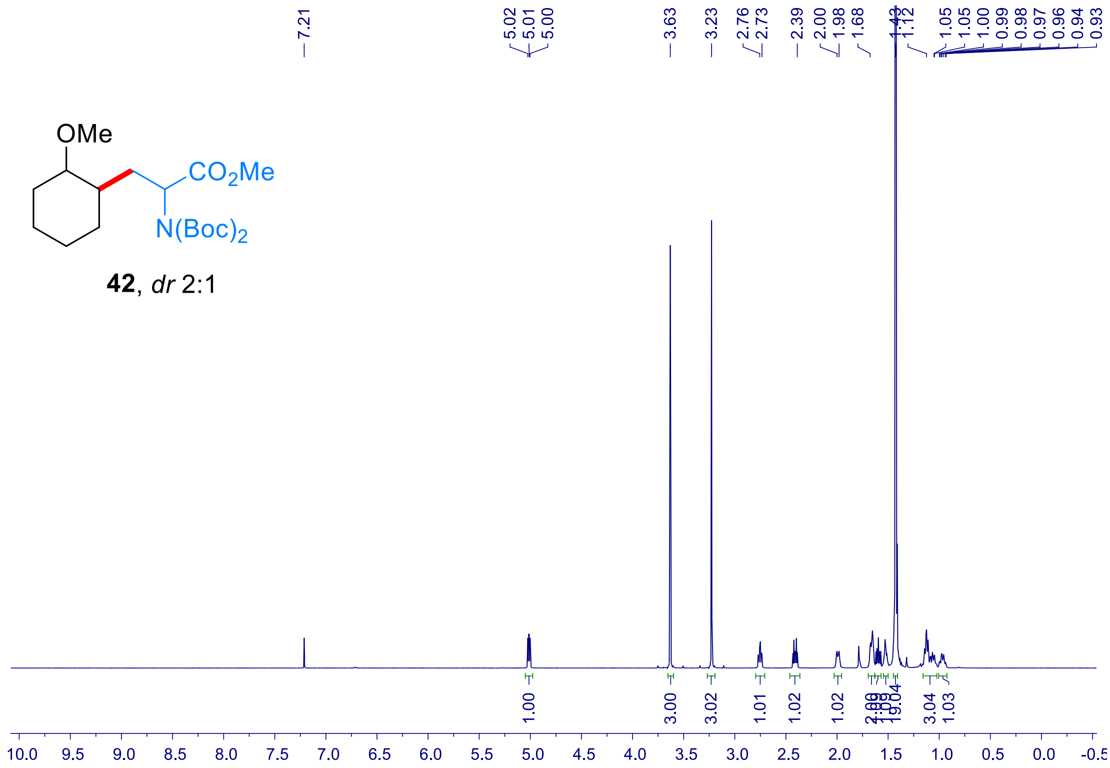

(42)-^{13}C NMR (101 MHz, $CDCl_3$)

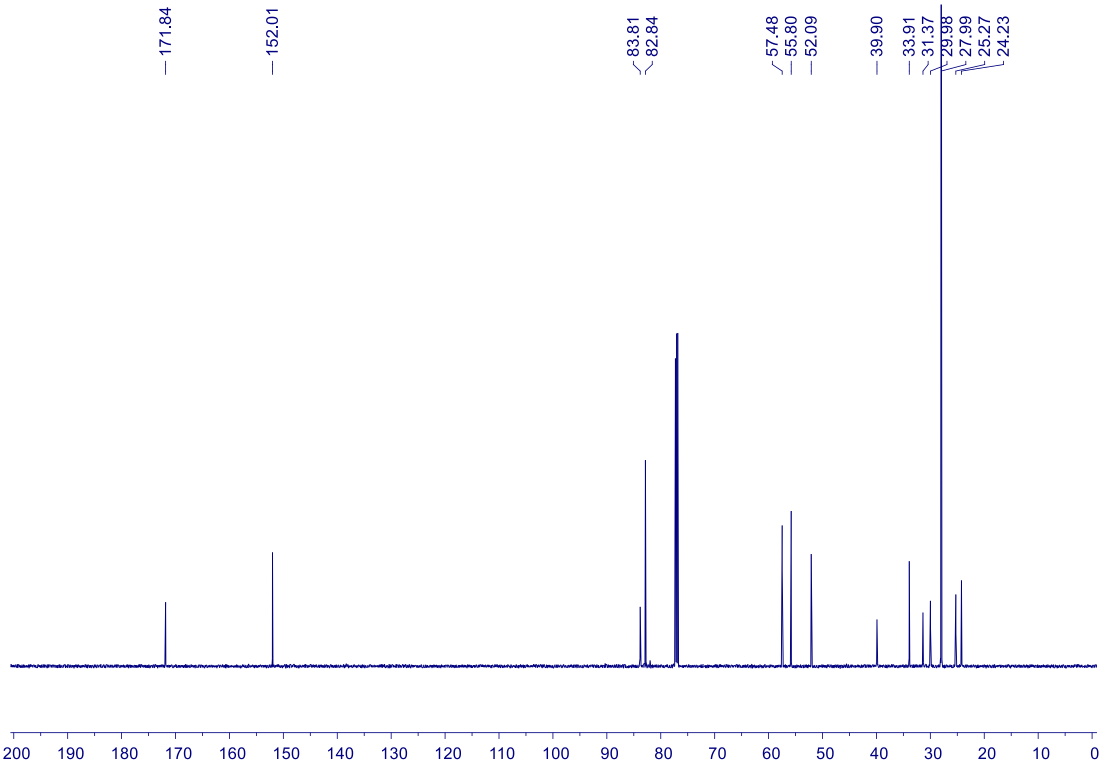

Methyl 2-(Bis(*tert*-butoxycarbonyl)amino)-3-(cyclohexanone-4-yl)propanoate (43)-^{1}H NMR (400 MHz, $CDCl_3$)

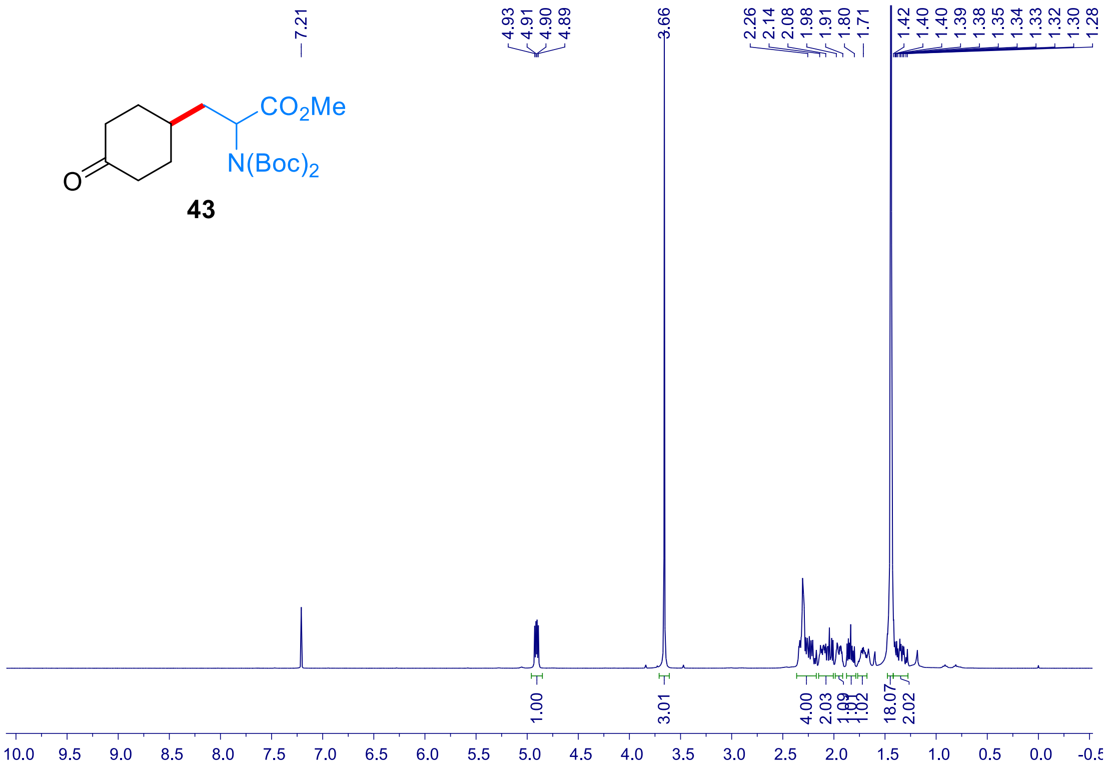

(43)-^{13}C NMR (101 MHz, $CDCl_3$)

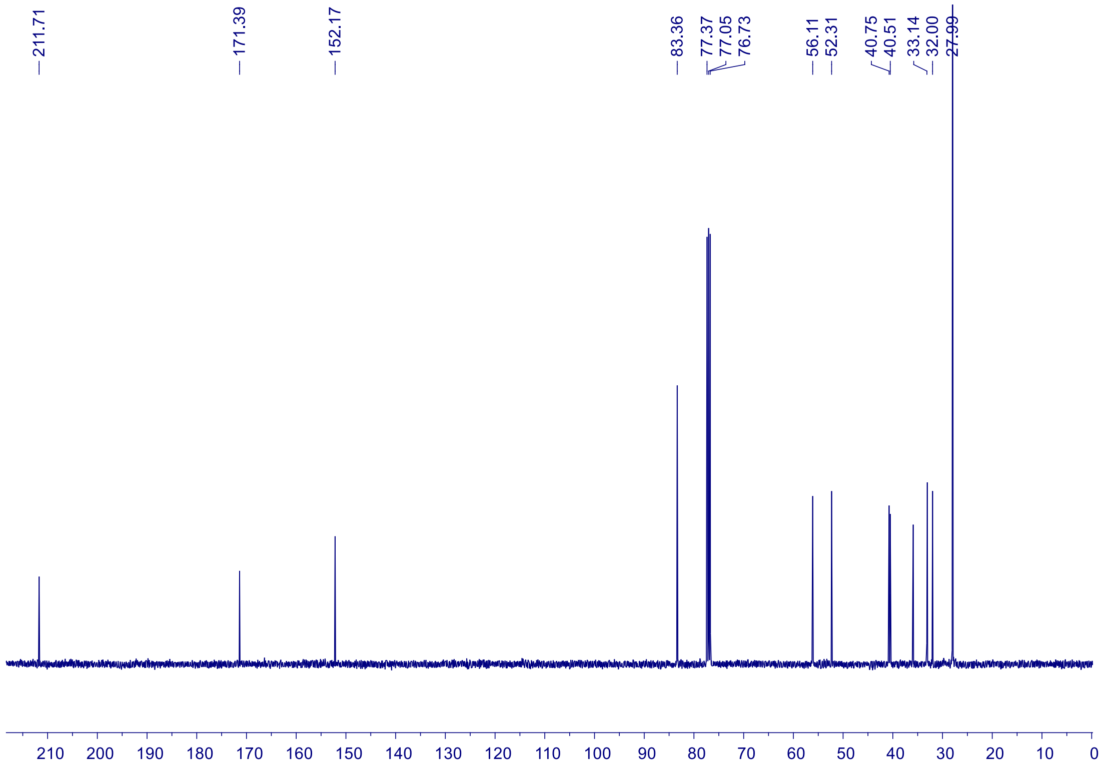

Methyl 2-(Bis(tert-butoxycarbonyl)amino)-3-(1-benzoylpiperidin-4-yl) propanoate (44)-^{1}H NMR (400 MHz, $CDCl_3$)

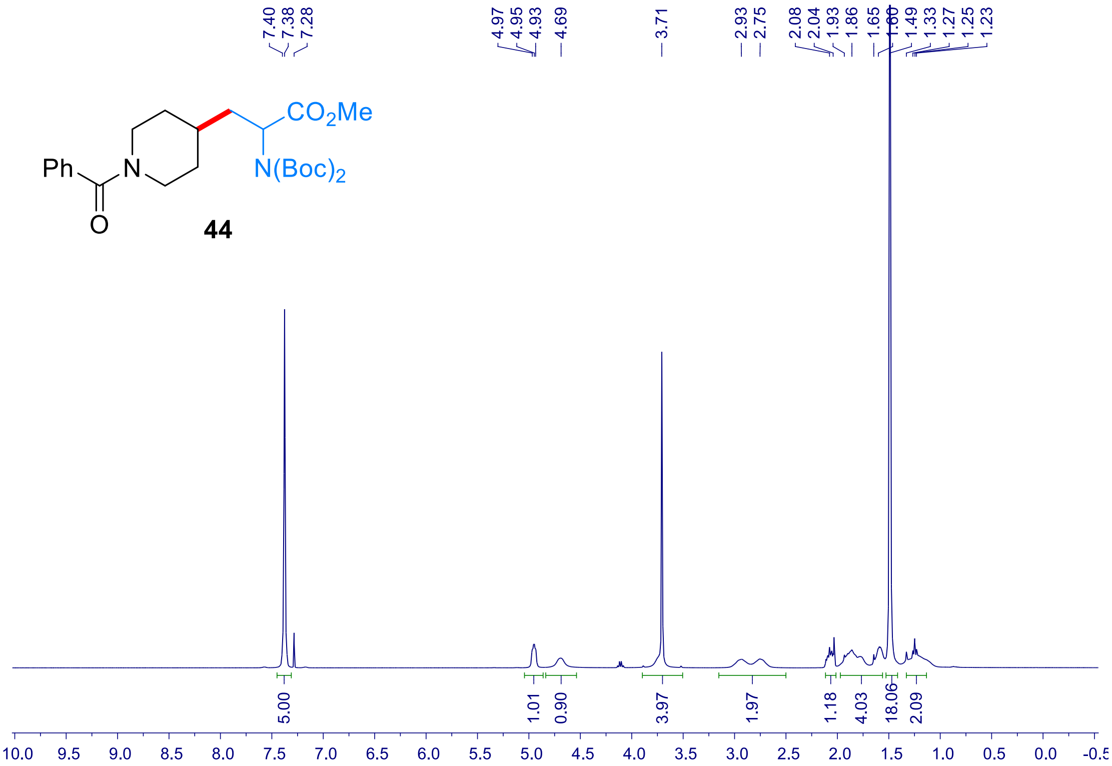

(44)-^{13}C NMR (101 MHz, $CDCl_3$)

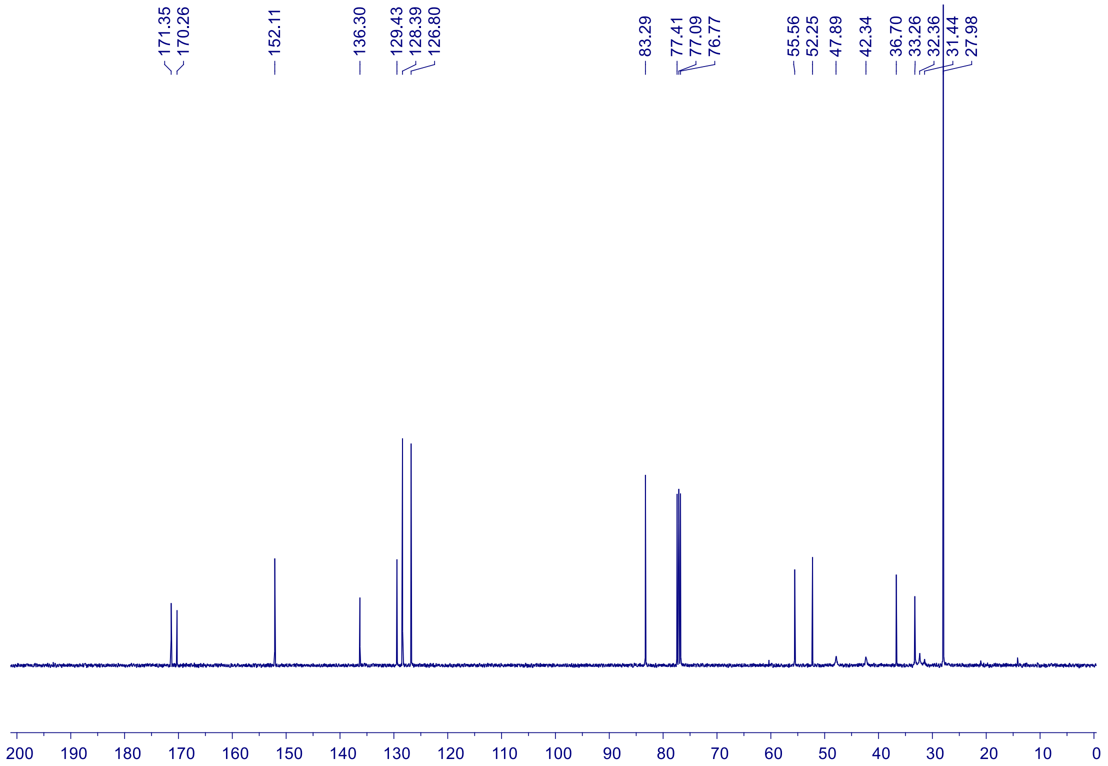

Methyl2-(bis(tert-butoxycarbonyl)amino)-3-(4-((tert-butoxycarbonyl)amino)cyclohexyl)propanoate (45)-^{1}H NMR (400 MHz, $CDCl_3$)

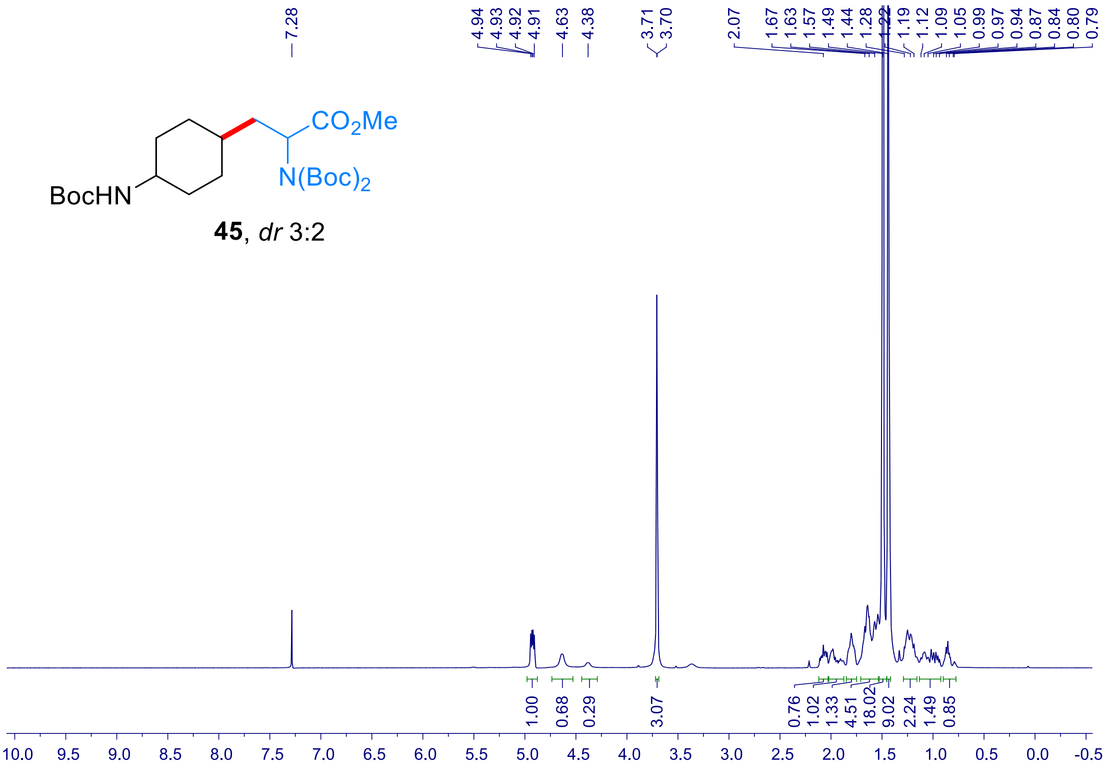

(45)-^{13}C NMR (101 MHz, $CDCl_3$)

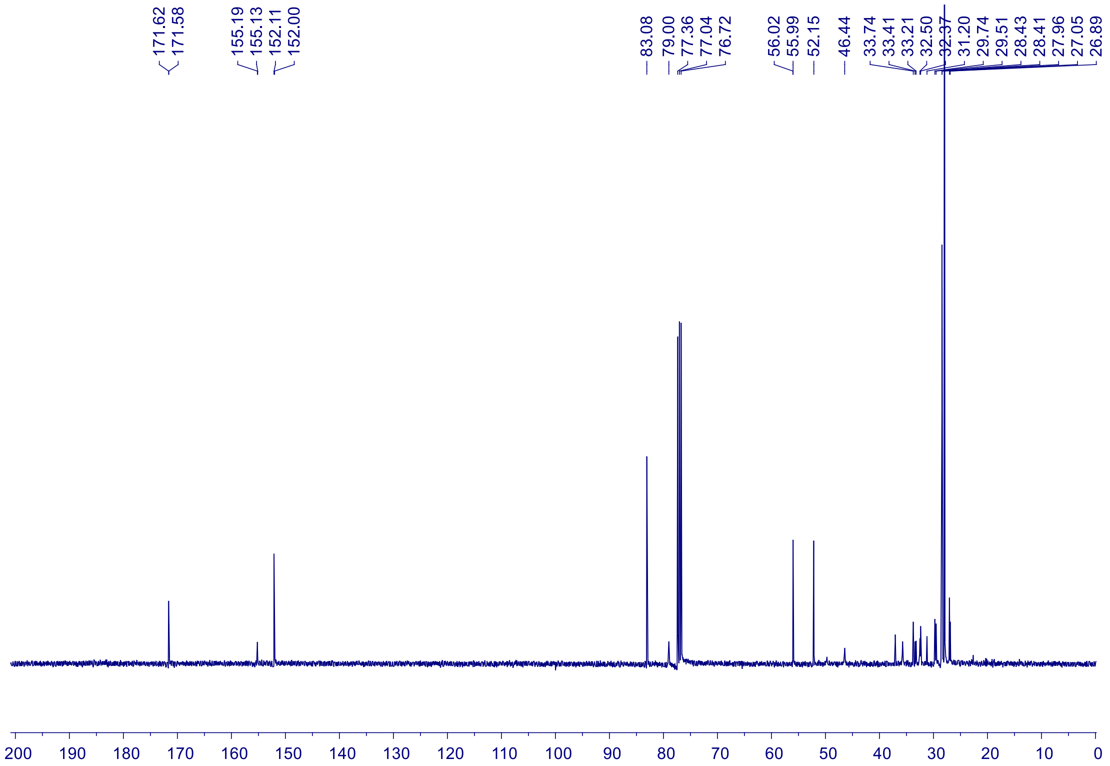

Methyl 2-(Bis(*tert*-butoxycarbonyl)amino)-3-(octahydro-1H-4,7-methanoindene -4-yl)propanoate (46)-^{1}H NMR (400 MHz, $CDCl_3$)

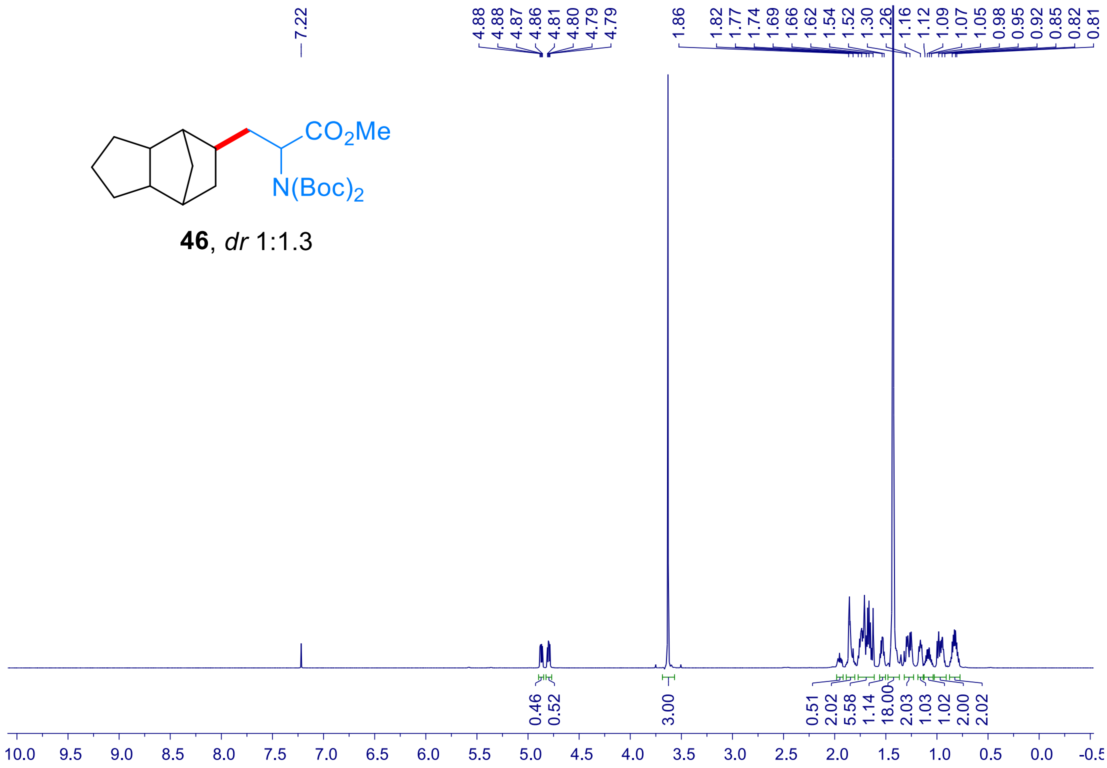

(46)-^{13}C NMR (101 MHz, $CDCl_3$)

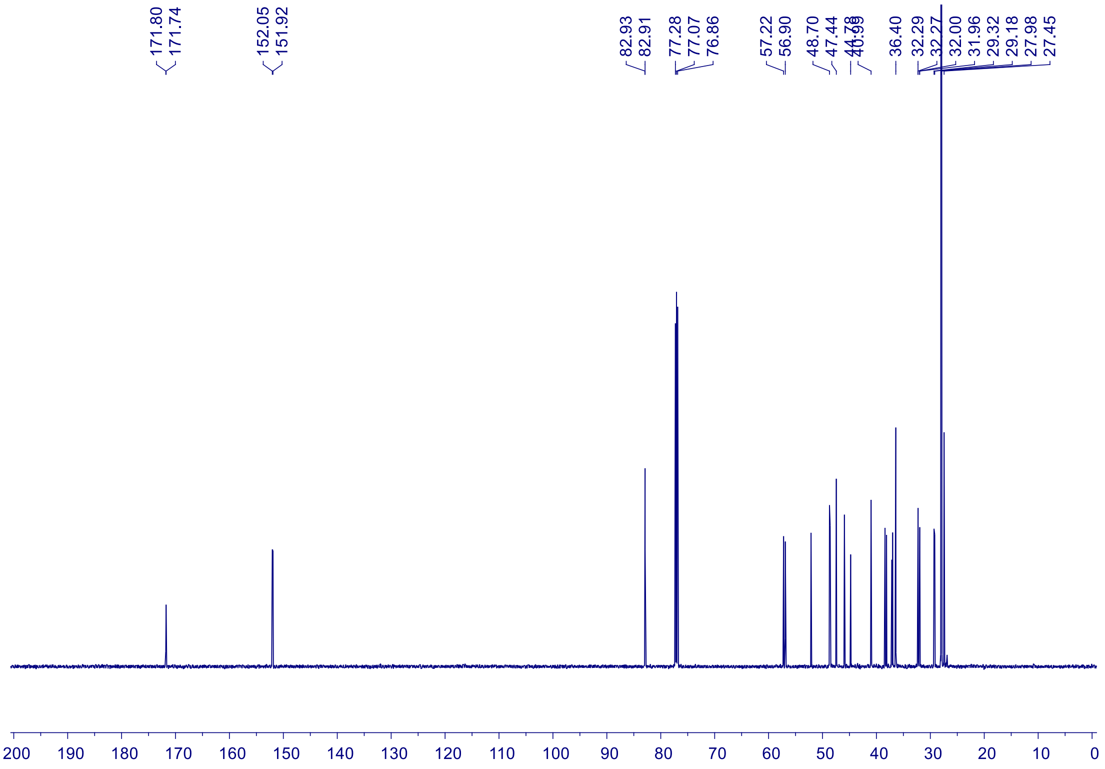

Methyl 2-(Bis(tert-butoxycarbonyl)amino)-3-(4-acetamidooctahydropentalen -1-yl)propanoate (47)-^{1}H NMR (400 MHz, $CDCl_3$)

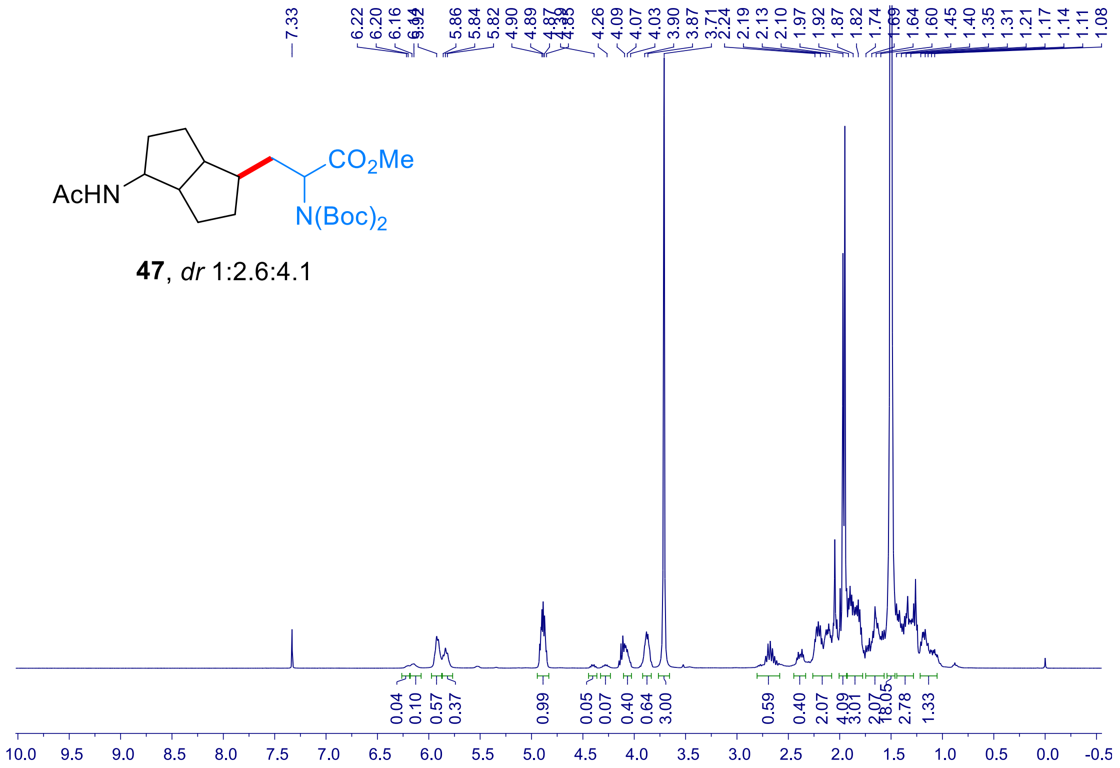

(47)-^{13}C NMR (101 MHz, $CDCl_3$)

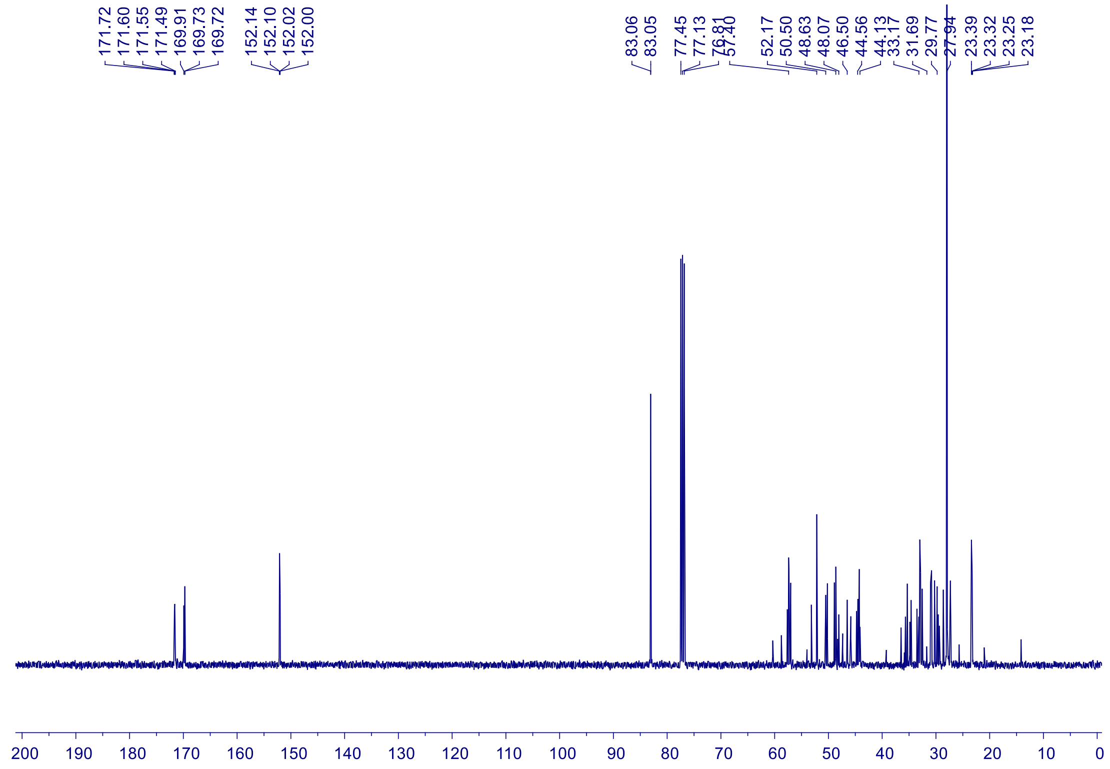

Methyl 2-(Bis(tert-butoxycarbonyl)amino)-4-((*1r,3R,5S*)-adamantan-1-yl) pentanoate (48)-^{1}H NMR (400 MHz, $CDCl_3$)

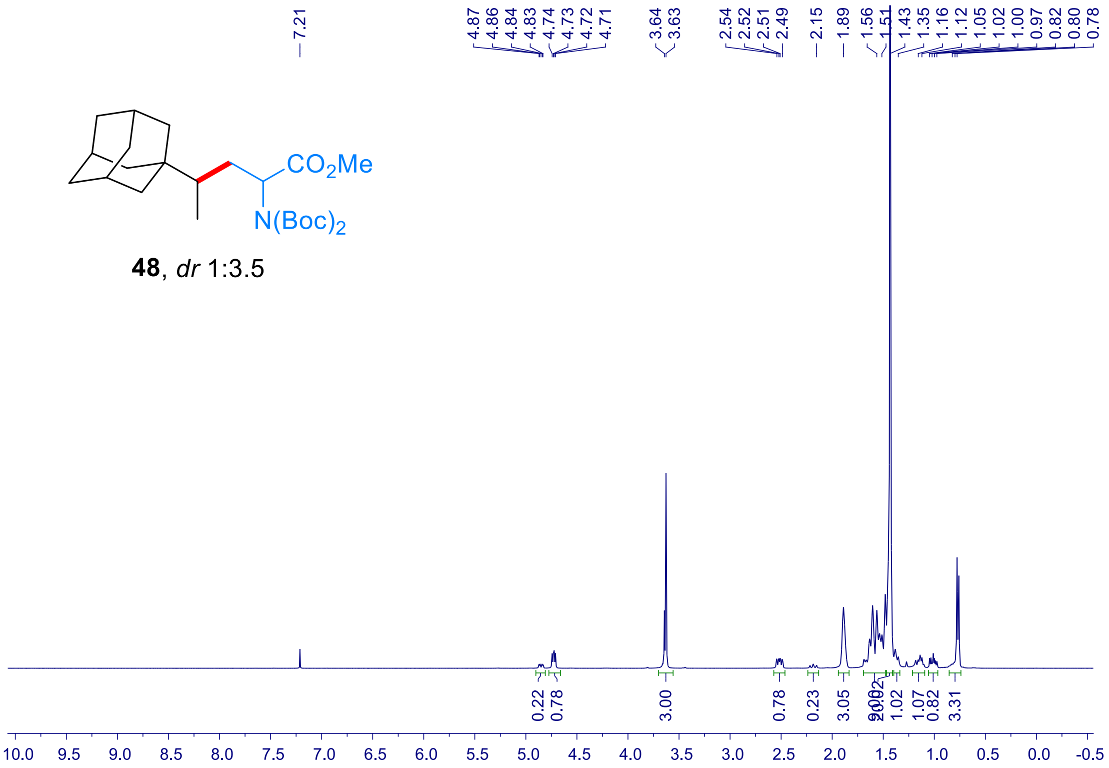

(48)-^{13}C NMR (101 MHz, $CDCl_3$)

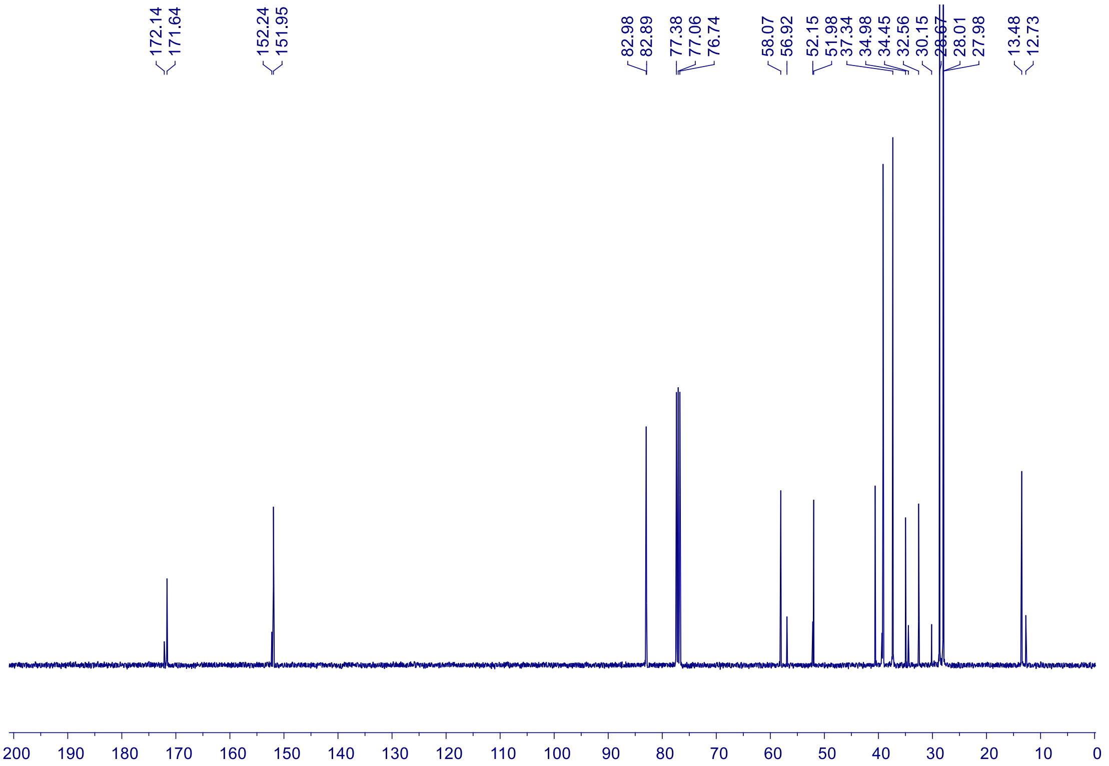

Methyl 2-(Bis(*tert*-butoxycarbonyl)amino)-3-(1-oxaspiro[5.5]undecane-4-yl) propanoate (49)-^{1}H NMR (400 MHz, CDCl$_3$)

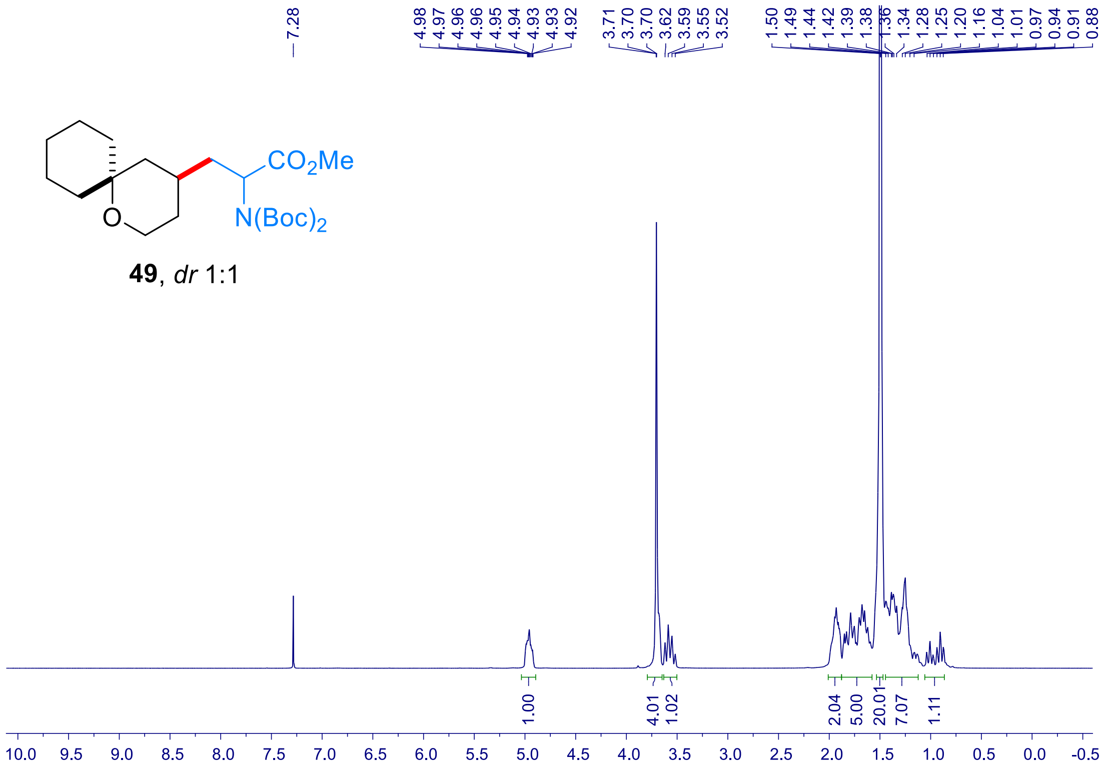

(49)-^{13}C NMR (101 MHz, CDCl$_3$)

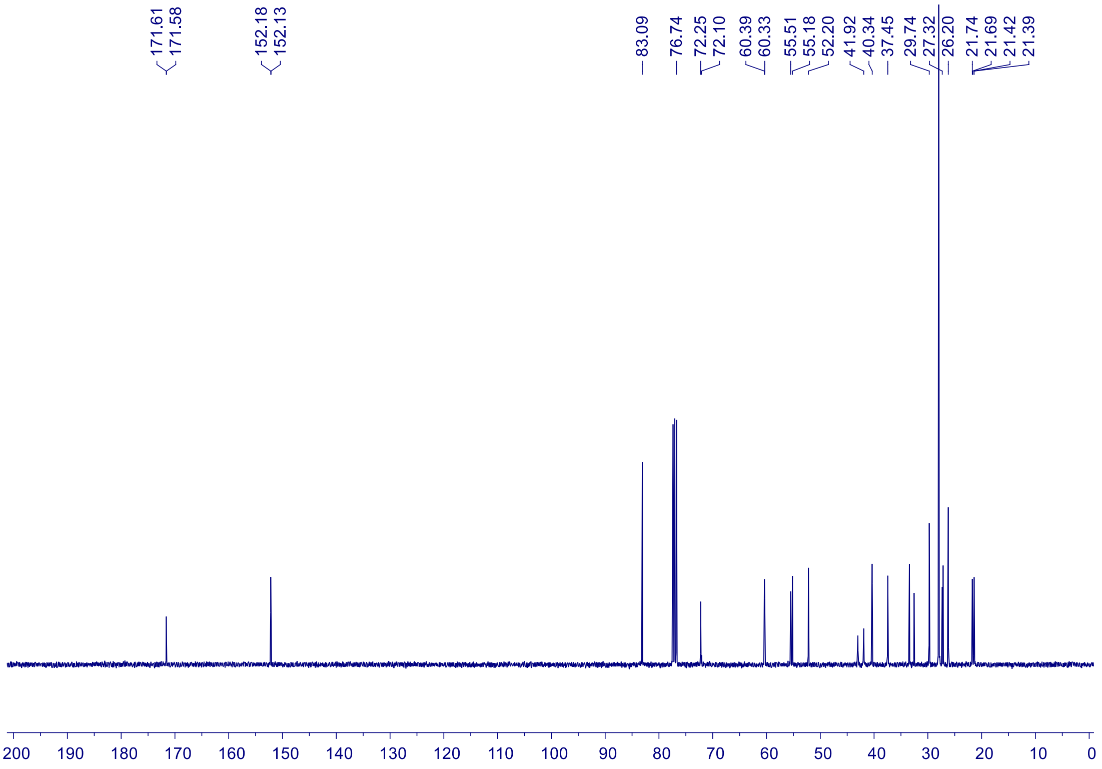

3-(1-tosylpiperidin-4-yl)propanenitrile (50)-^{1}H NMR (400 MHz, $CDCl_3$)

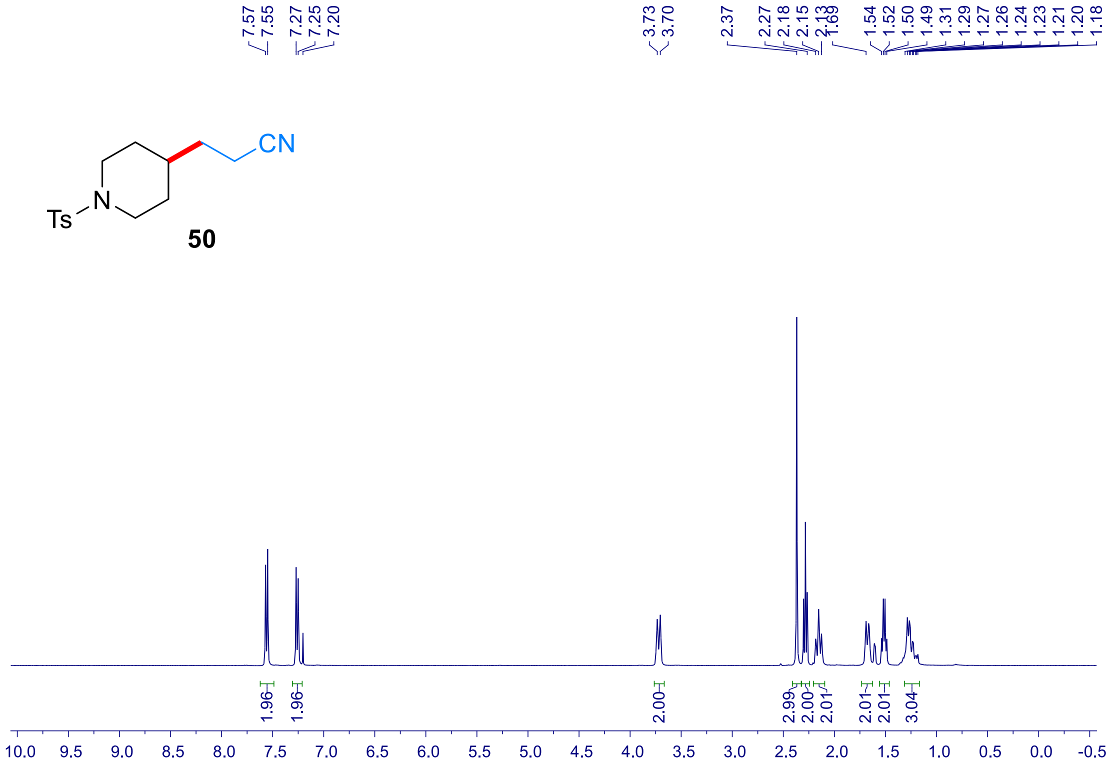

(50)-^{13}C NMR (101 MHz, $CDCl_3$)

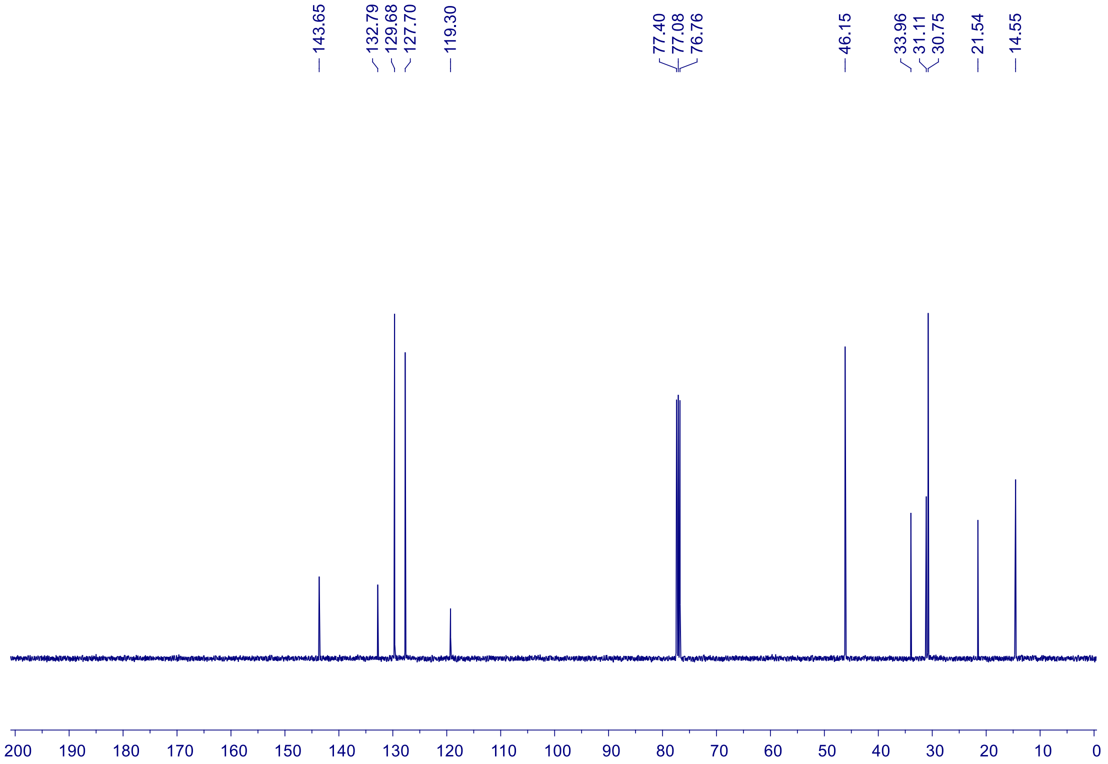

Methyl 2-(bis(tert-butoxycarbonyl)amino)-6-methylheptanoate (51)-^{1}H NMR (400 MHz, CDCl$_3$)

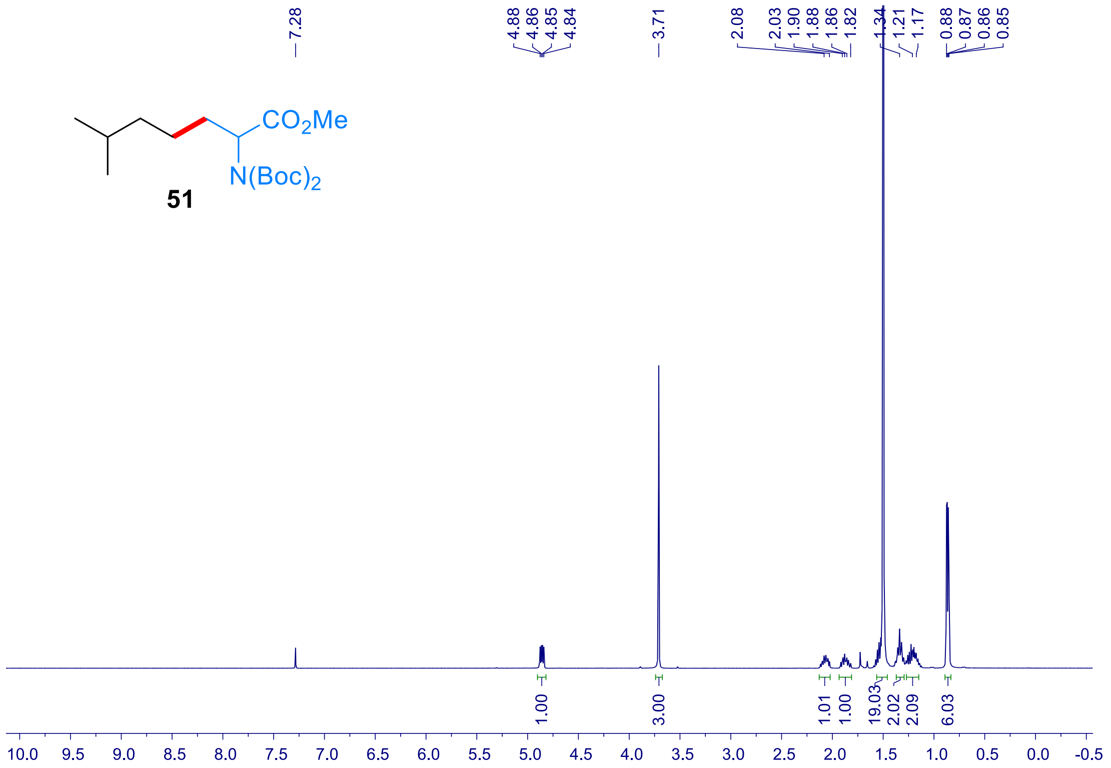

(51)-^{13}C NMR (101 MHz, CDCl$_3$)

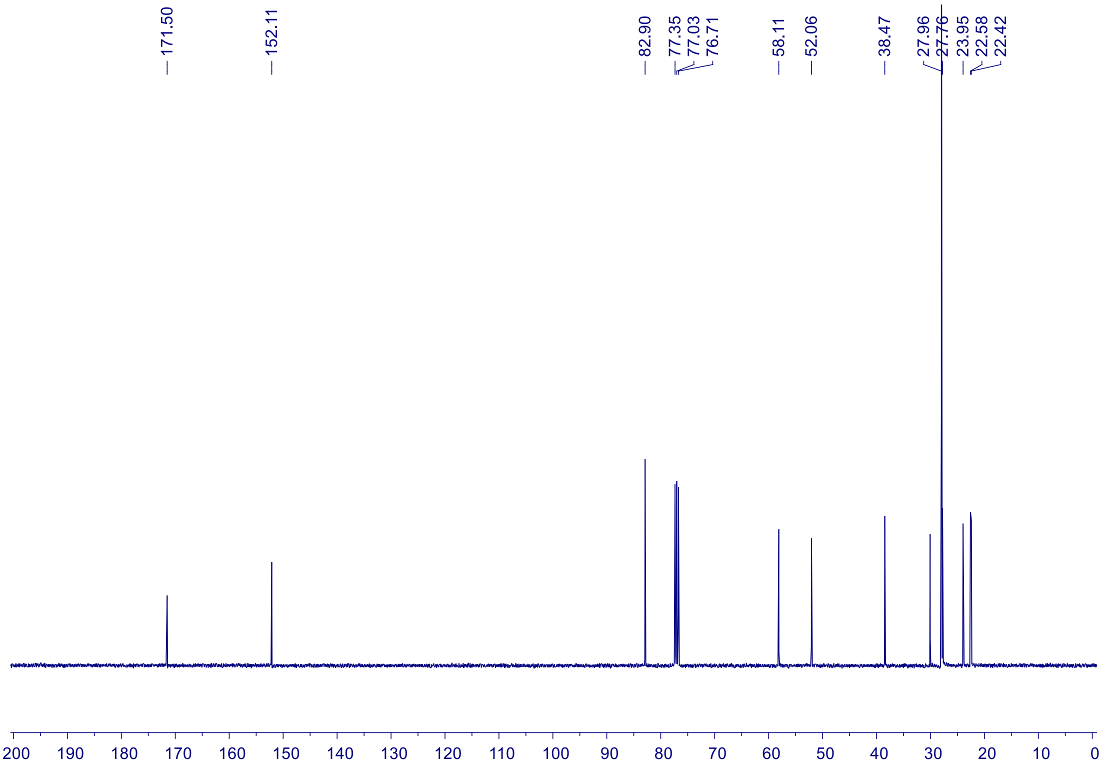

Methyl 2-(bis(tert-butoxycarbonyl)amino)-undecanoate (52)-^{1}H NMR (400 MHz, $CDCl_3$)

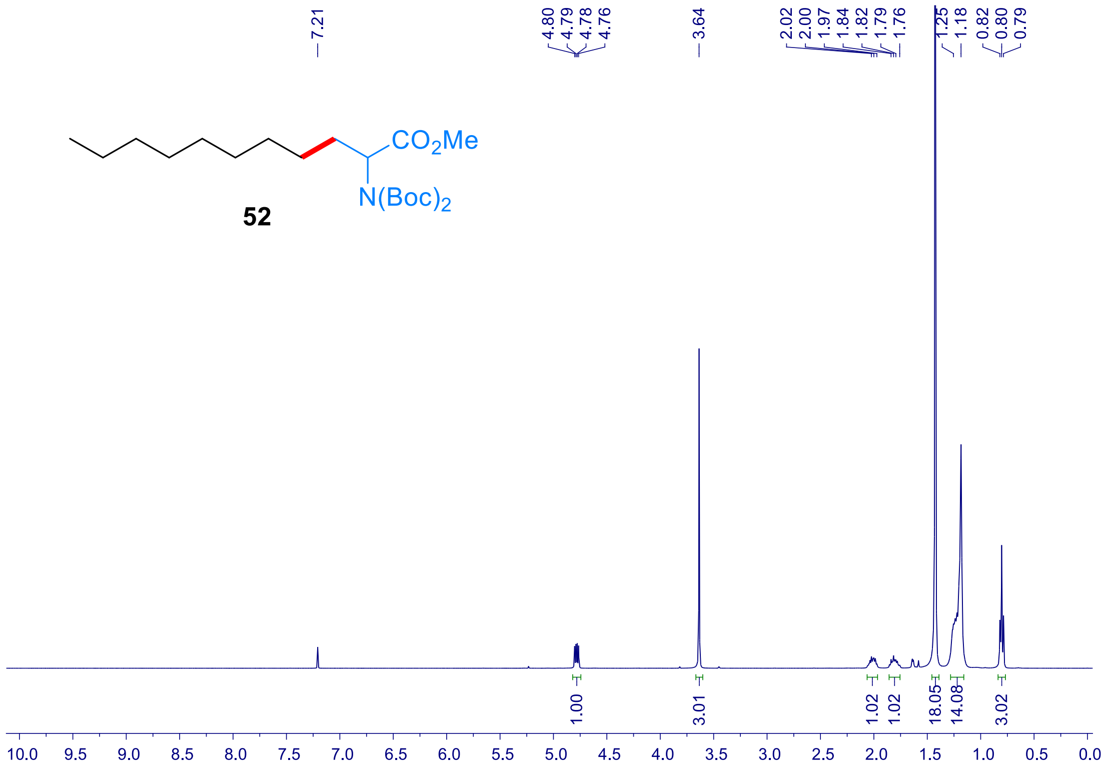

(52)-^{13}C NMR (101 MHz, $CDCl_3$)

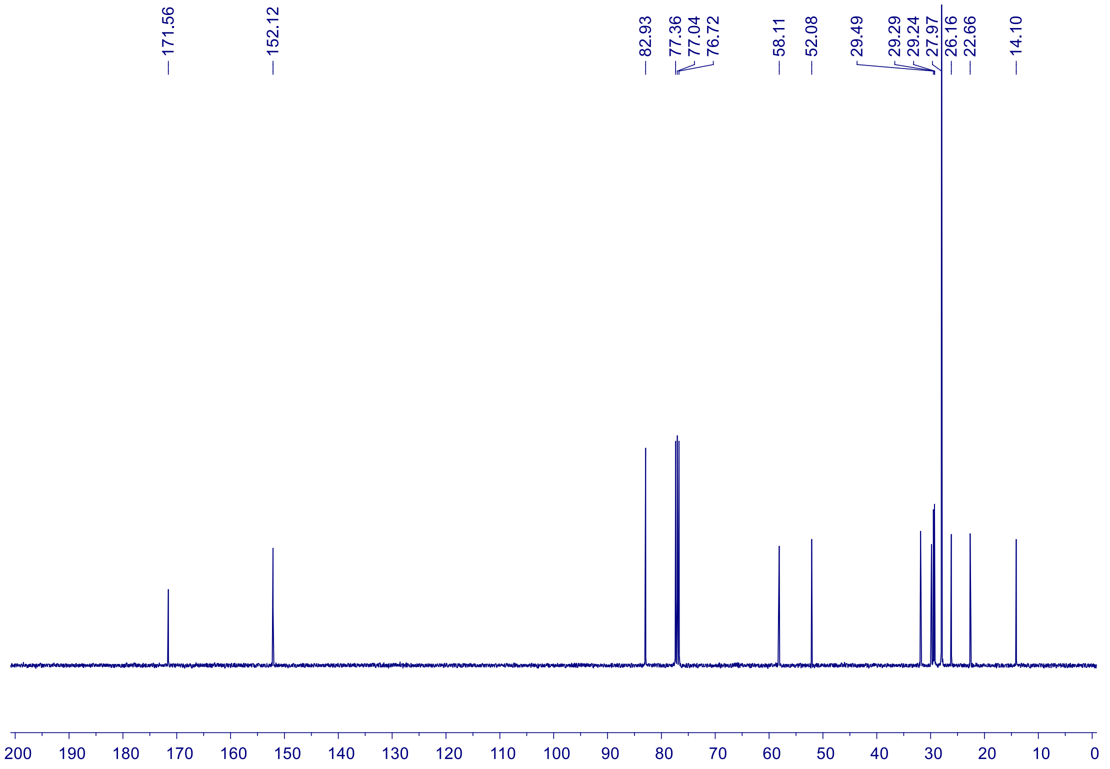

Methyl 2-(bis(*tert*-butoxycarbonyl)amino)-6-chlorohexanoate (53)-^{1}H NMR (400 MHz, $CDCl_3$)

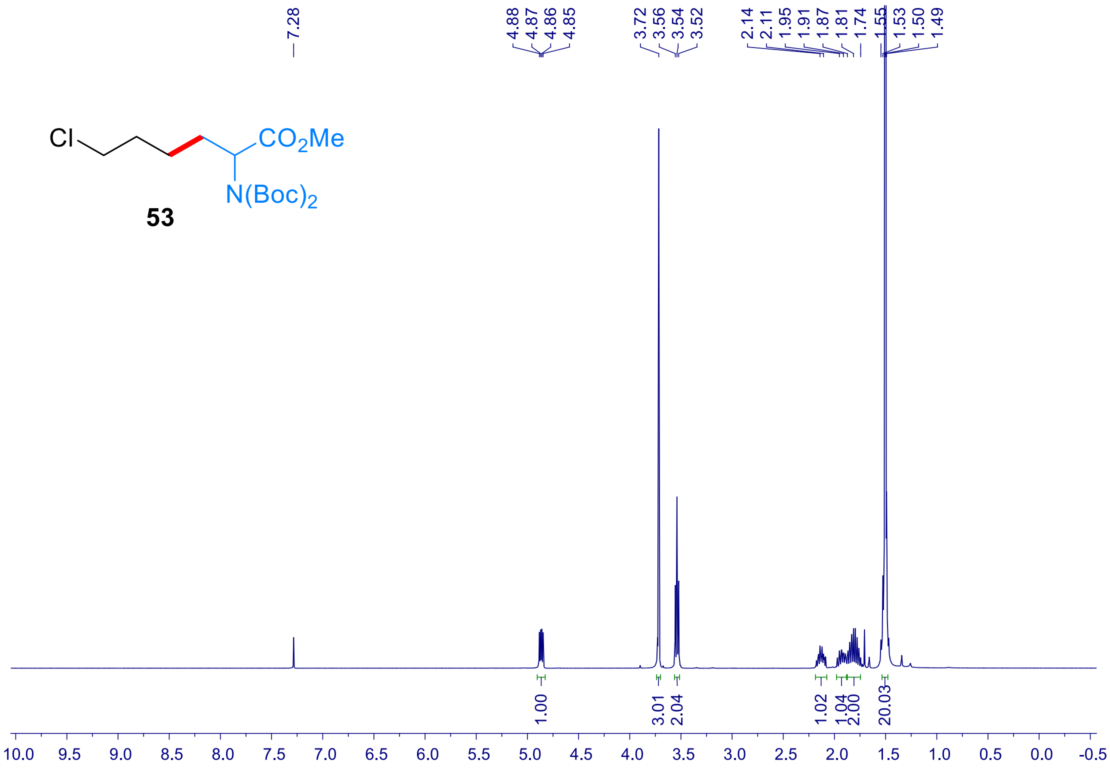

(53)-^{13}C NMR (101 MHz, $CDCl_3$)

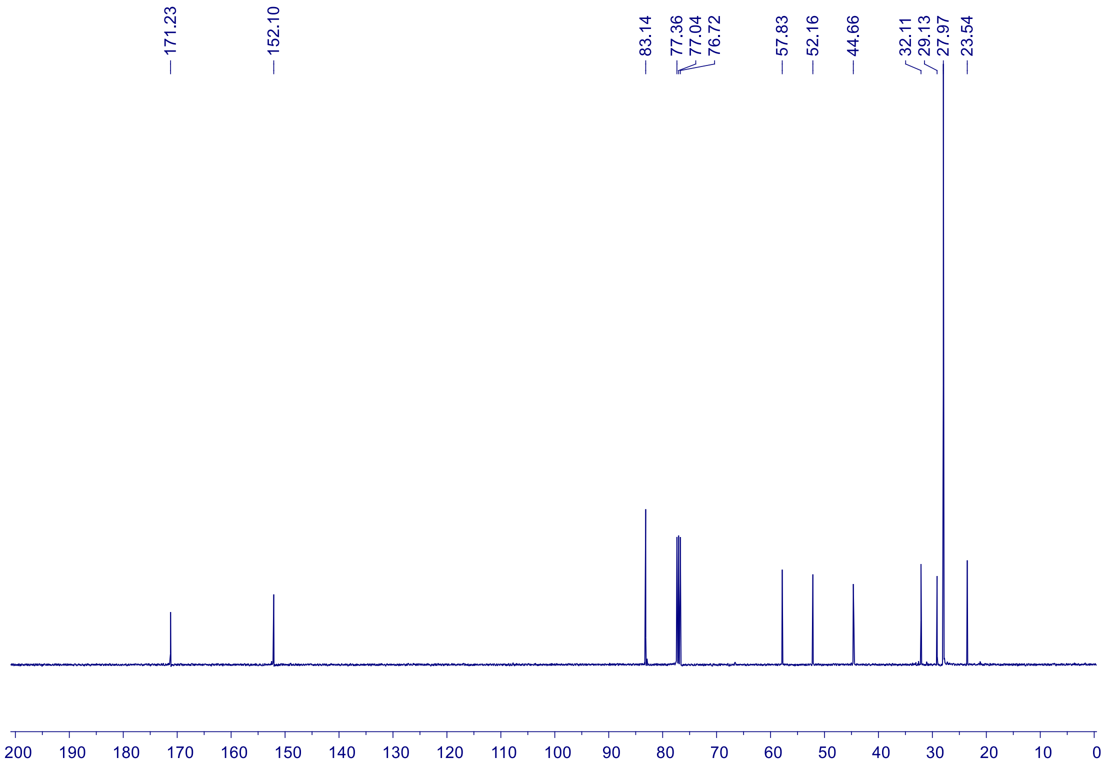

Methyl 2-(bis(*tert*-butoxycarbonyl)amino)oct-7-ynoate (54)-^{1}H NMR (400 MHz, $CDCl_3$)

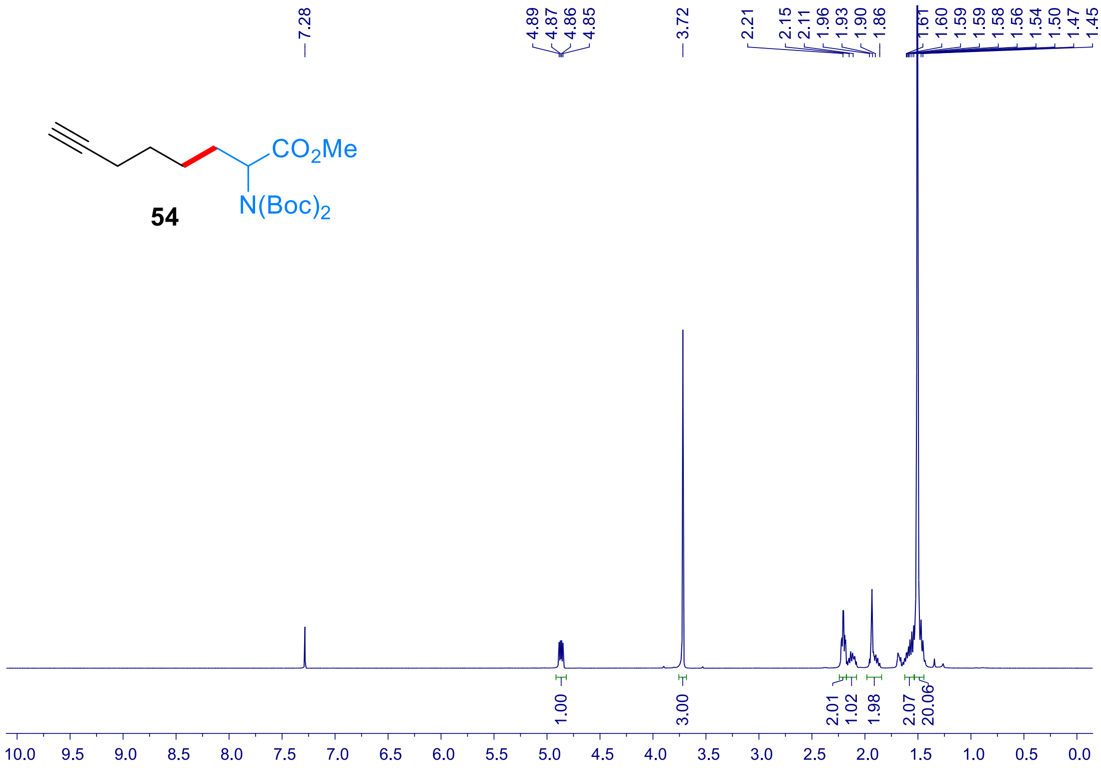

(54)-^{13}C NMR (101 MHz, $CDCl_3$)

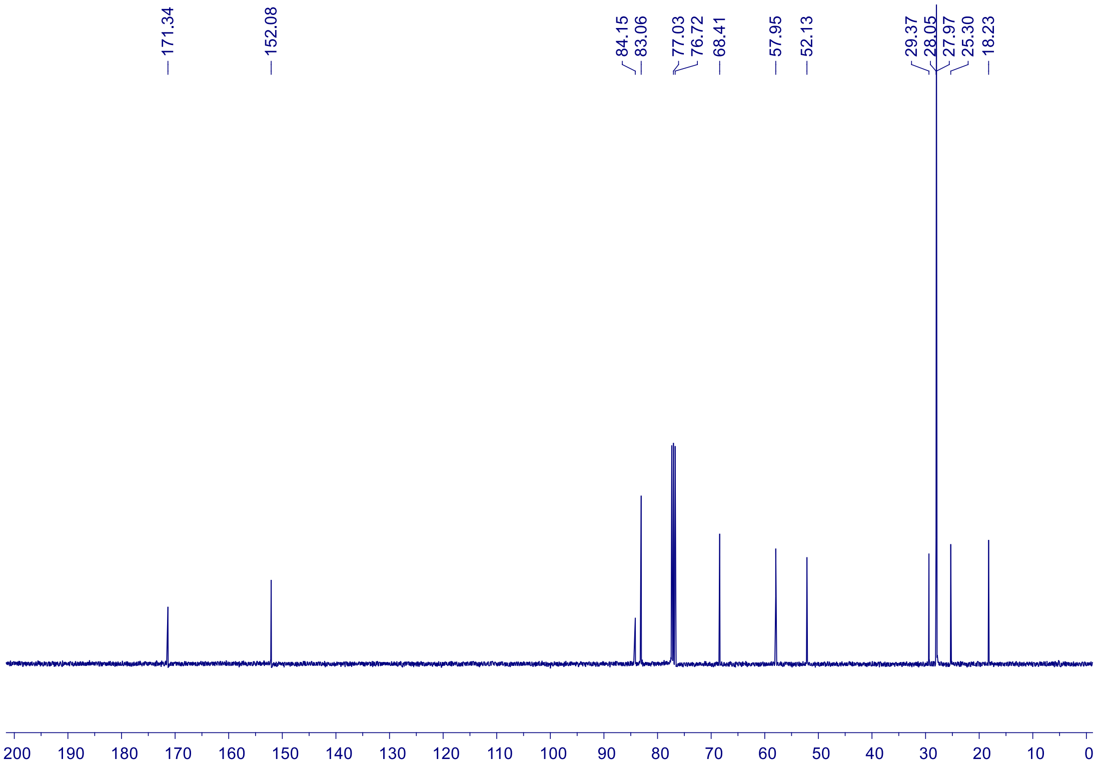

Methyl 2-(bis(tert-butoxycarbonyl)amino)-oct-7-enoate (55)-^{1}H NMR (400 MHz, $CDCl_3$)

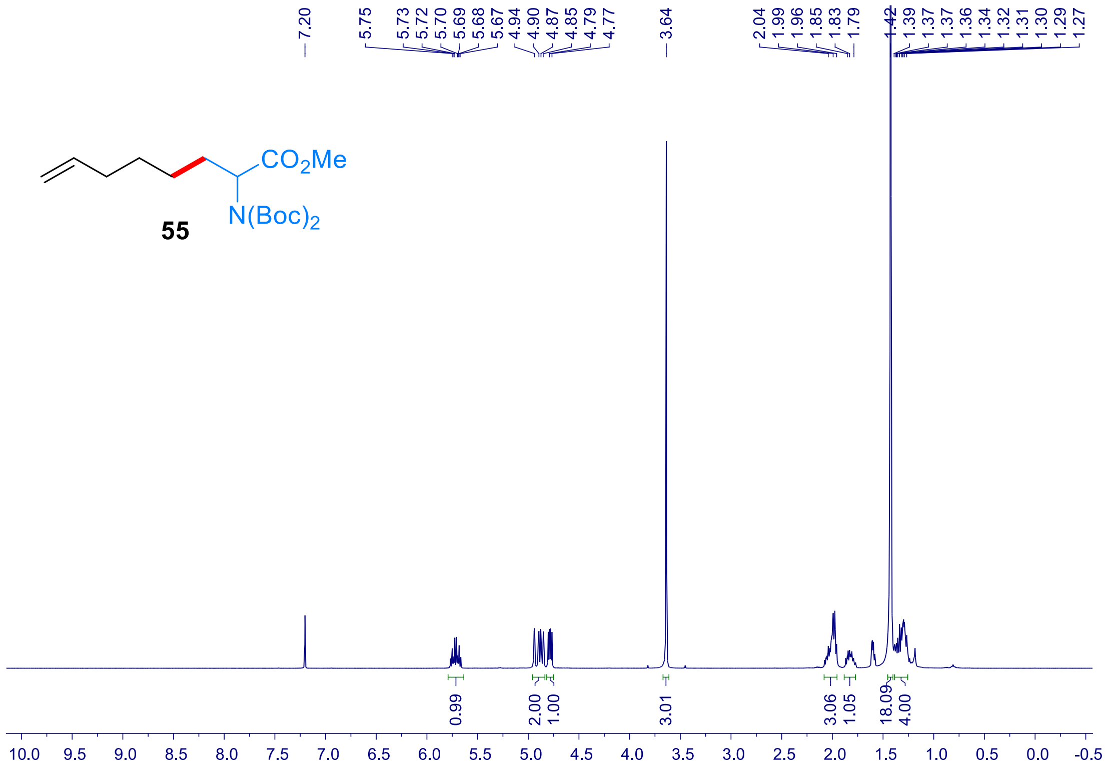

(55)-^{13}C NMR (101 MHz, $CDCl_3$)

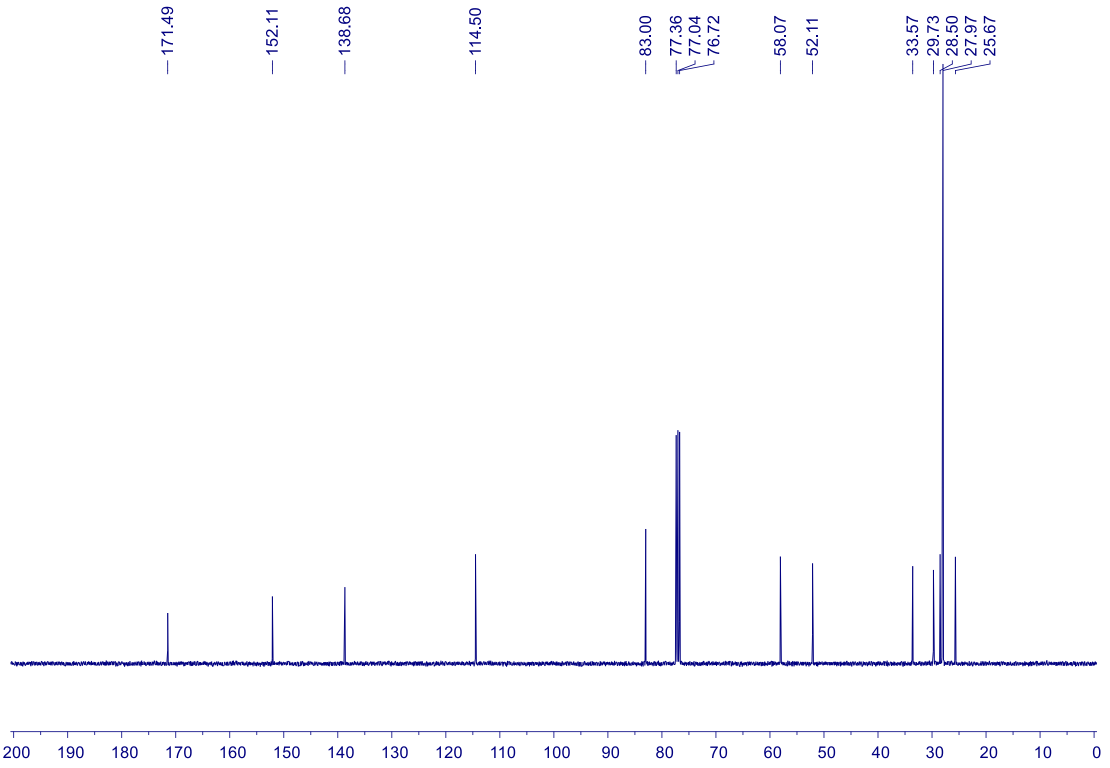

Methyl 2-(bis(tert-butoxycarbonyl)amino)-6-hydroxyhexanoate (56)-^{1}H NMR (400 MHz, $CDCl_3$)

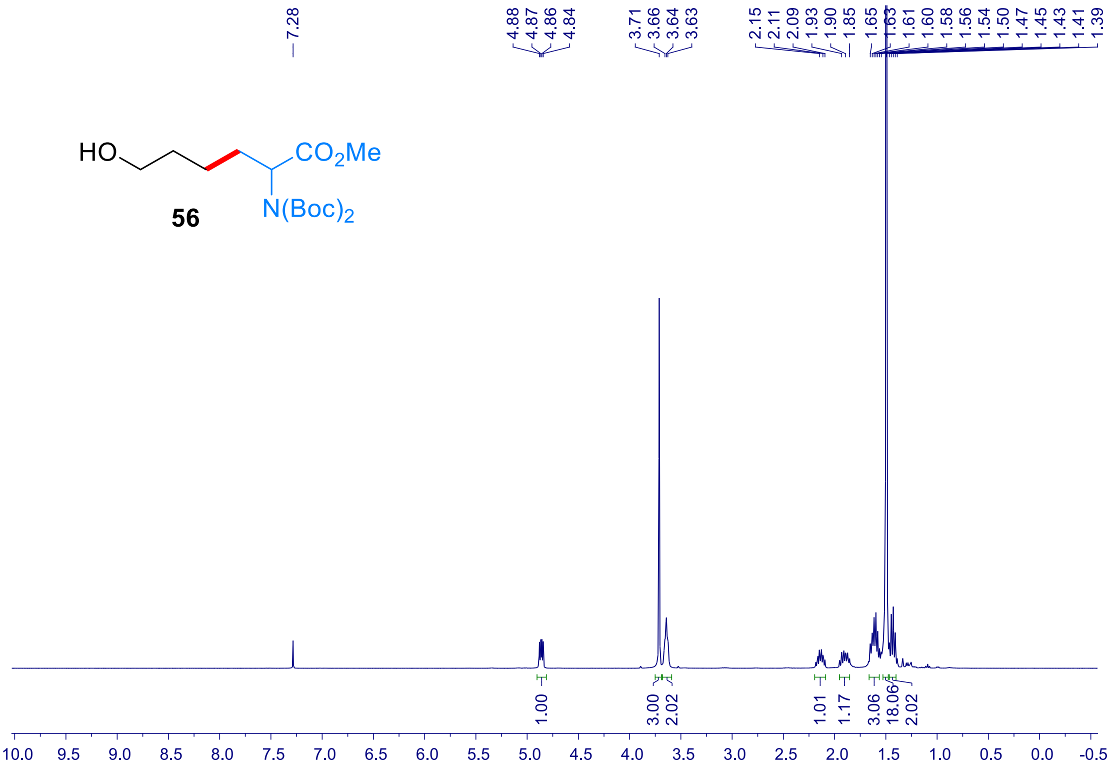

(56)-^{13}C NMR (101 MHz, $CDCl_3$)

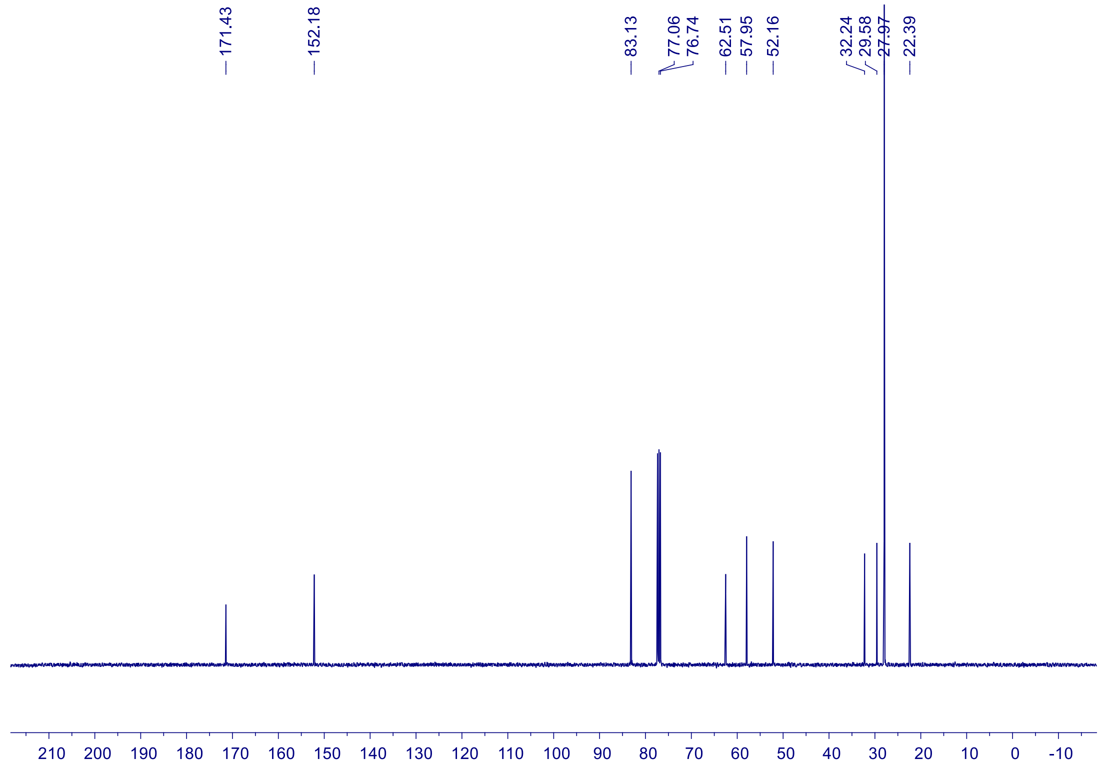

Methyl 2-(bis(tert-butoxycarbonyl)amino)-7-((tert-butyldimethylsilyl)oxy)heptanoate (57)-^{1}H NMR (400 MHz, $CDCl_3$)

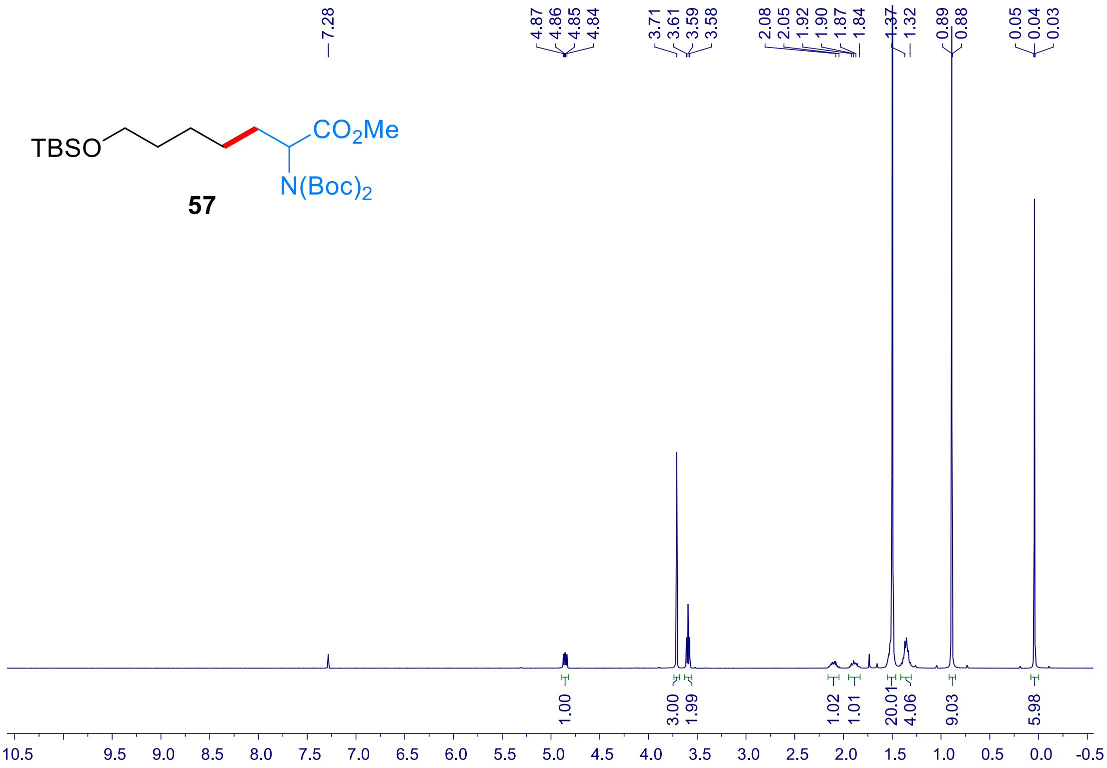

(57)-^{13}C NMR (101 MHz, $CDCl_3$)

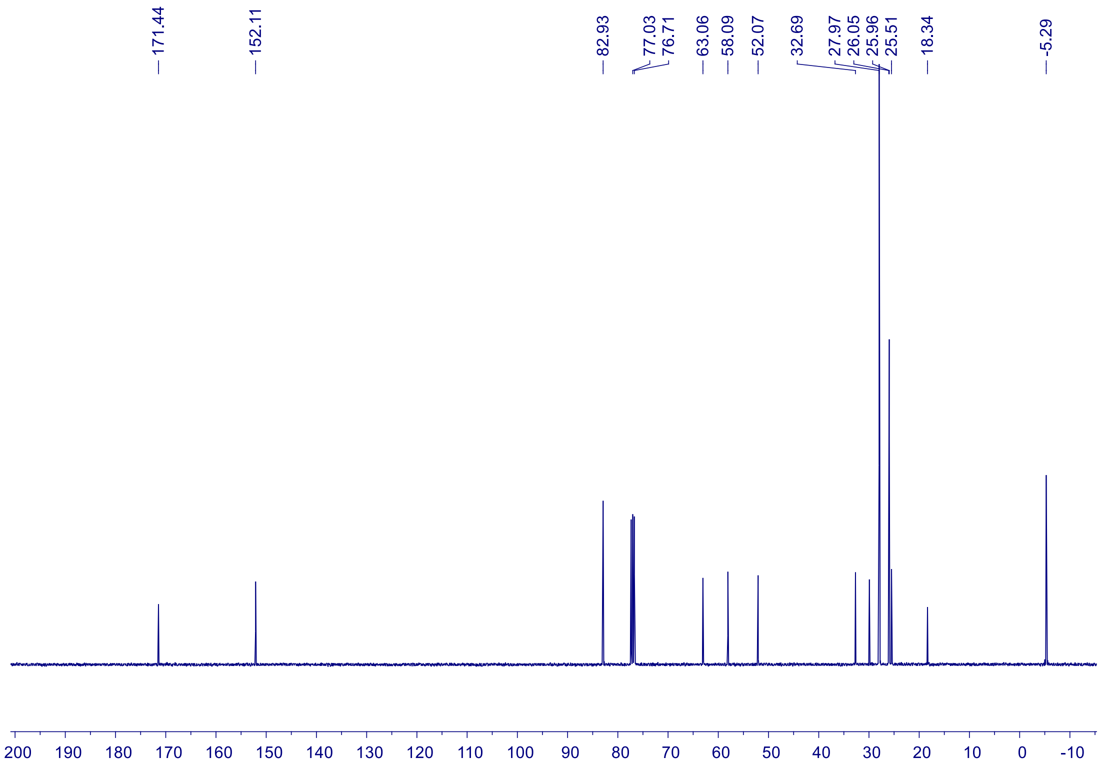

Methyl 2-(bis(tert-butoxycarbonyl)amino)-7-cyanoheptanoate (58)-^{1}H NMR (400 MHz, $CDCl_3$)

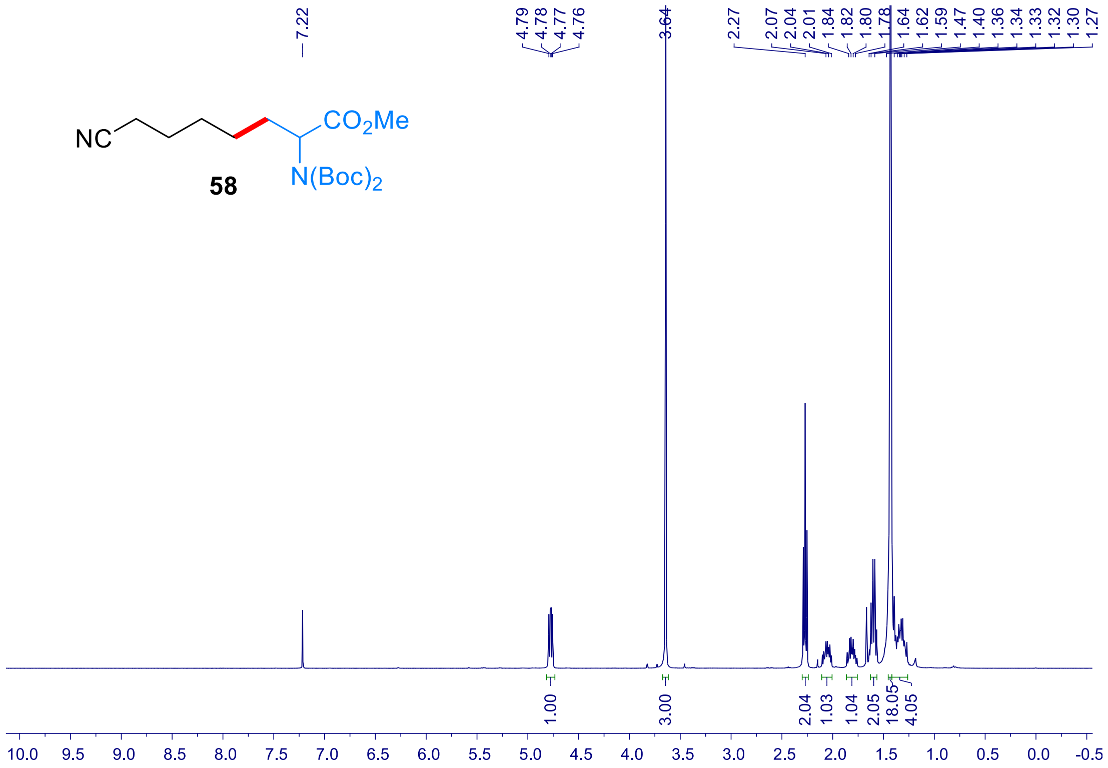

(58)-^{13}C NMR (101 MHz, $CDCl_3$)

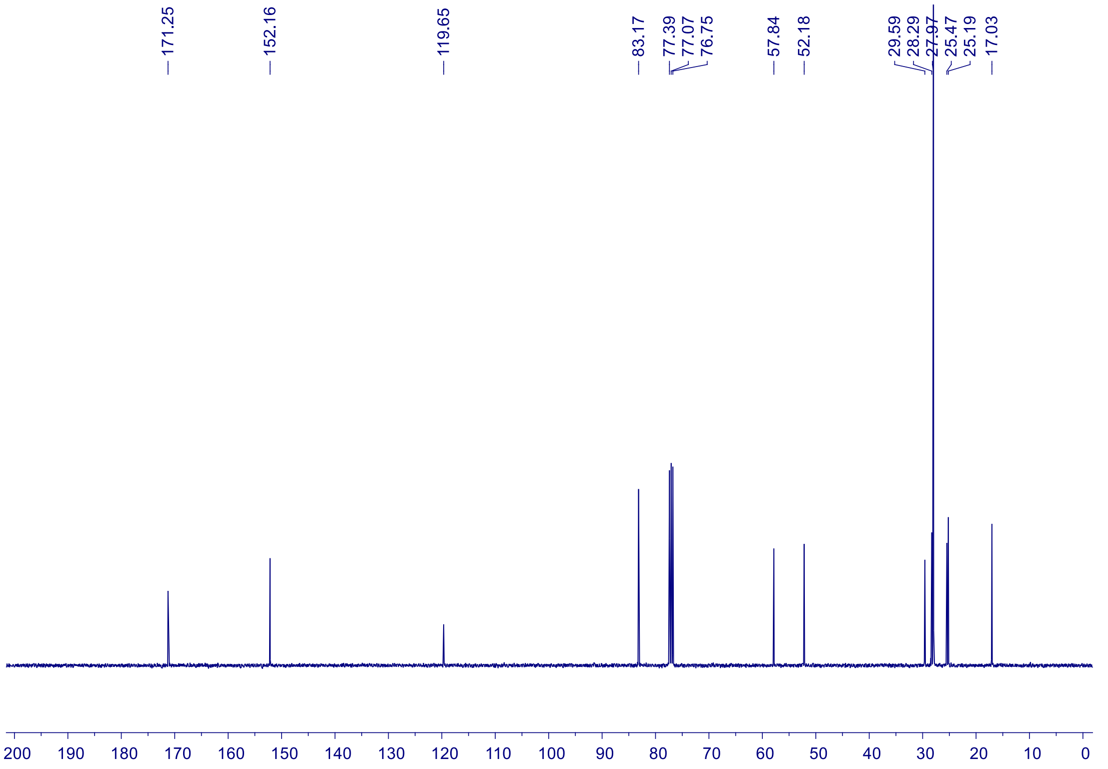

Methyl 2-(Bis(tert-butoxycarbonyl)amino)-4-(trimethylsilyl)butanoate (59)-^{1}H NMR (400 MHz, $CDCl_3$)

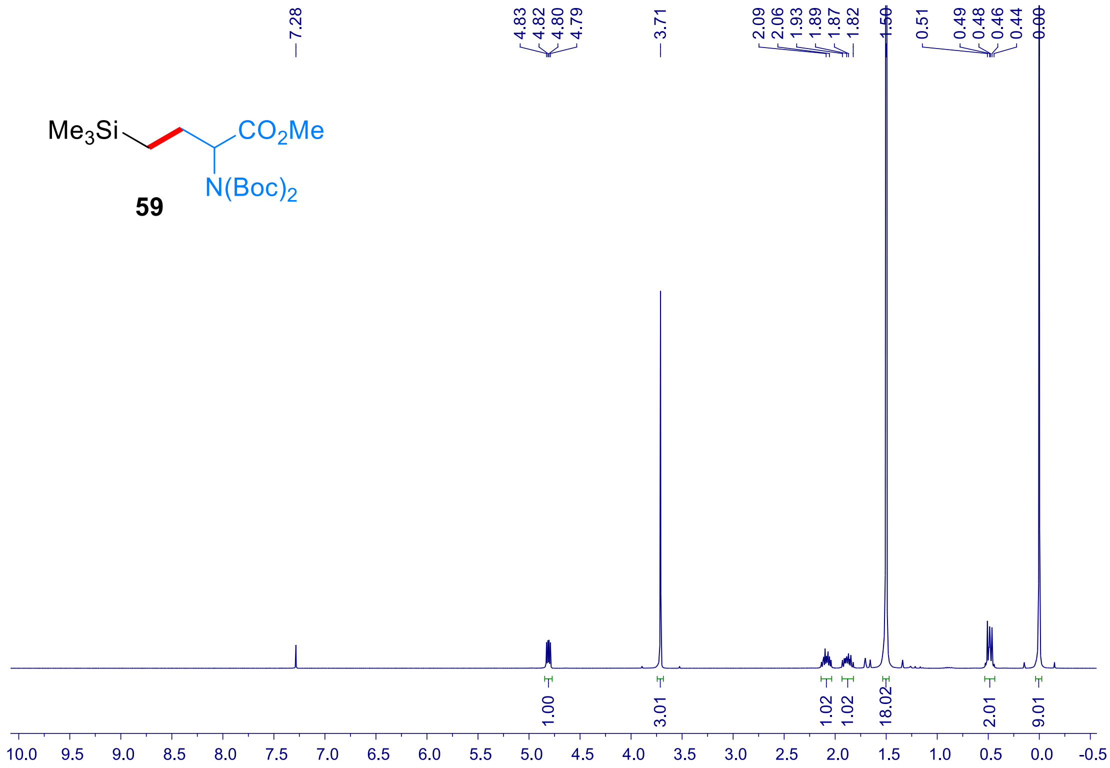

59

(59)-^{13}C NMR (101 MHz, $CDCl_3$)

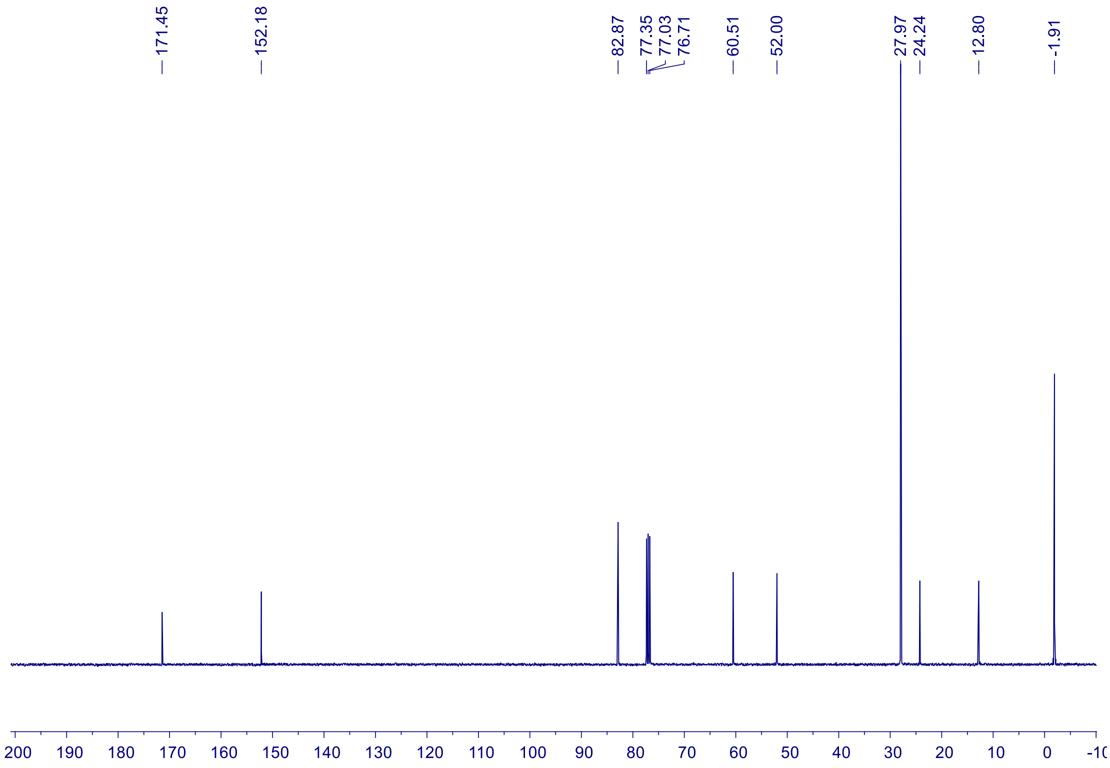

Methyl 2-(bis(tert-butoxycarbonyl)amino)-8-oxononanoate (60)-^{1}H NMR (400 MHz, $CDCl_3$)

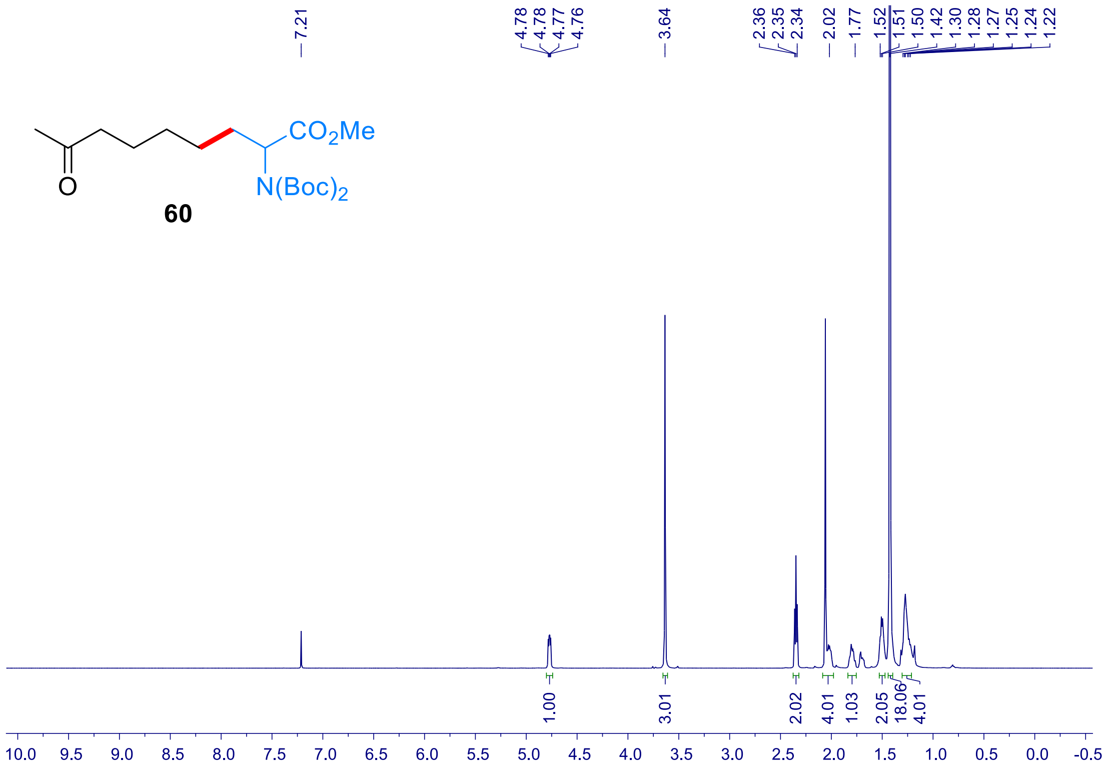

(60)-^{13}C NMR (101 MHz, $CDCl_3$)

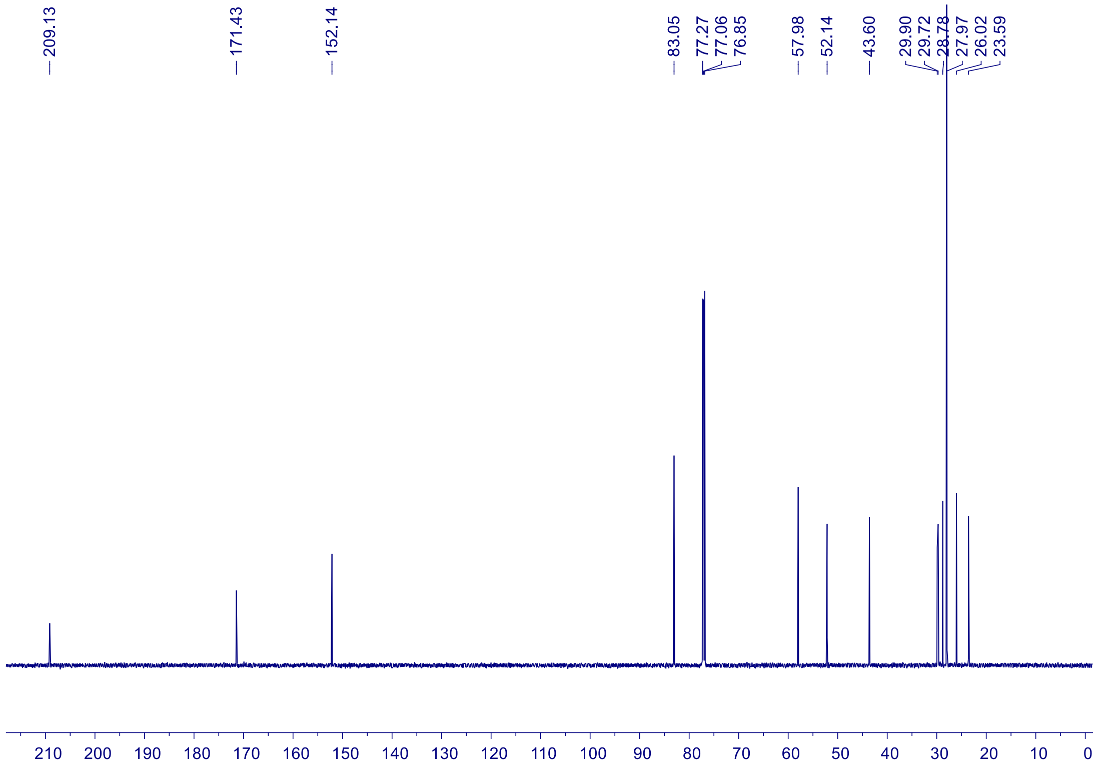

Methyl 2-(bis(tert-butoxycarbonyl)amino)-10-ethyl decanedioate (61)-^{1}H NMR (400 MHz, $CDCl_3$)

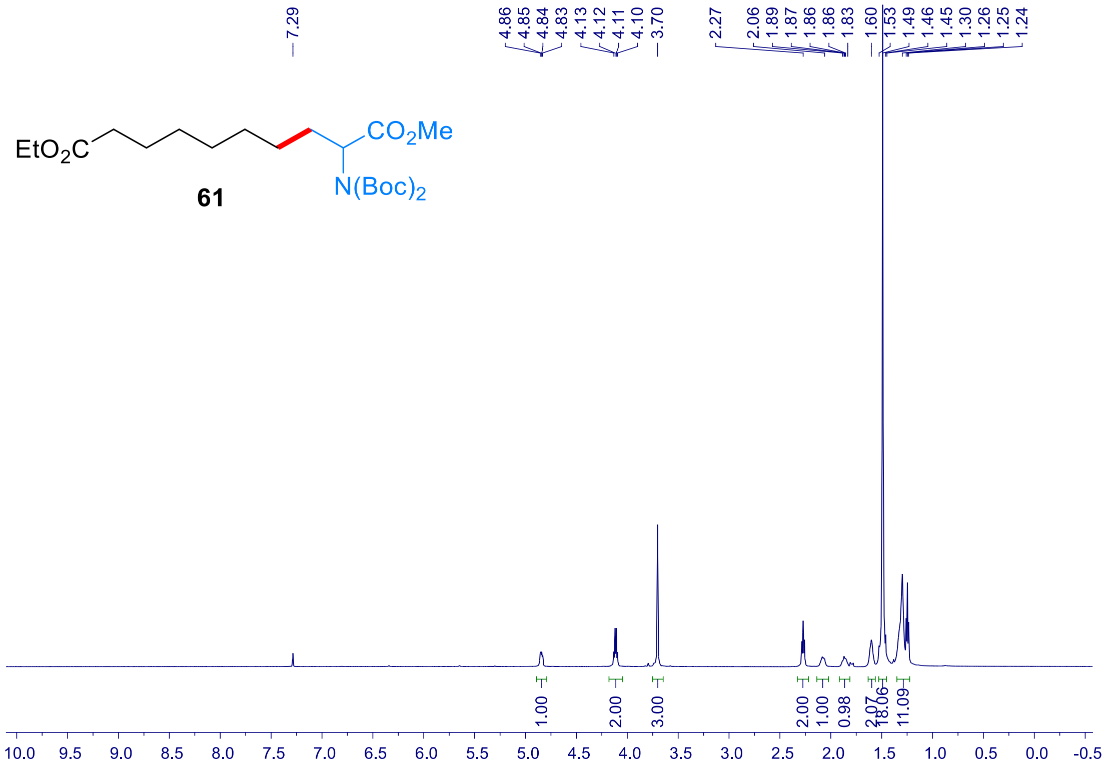

(61)-^{13}C NMR (101 MHz, $CDCl_3$)

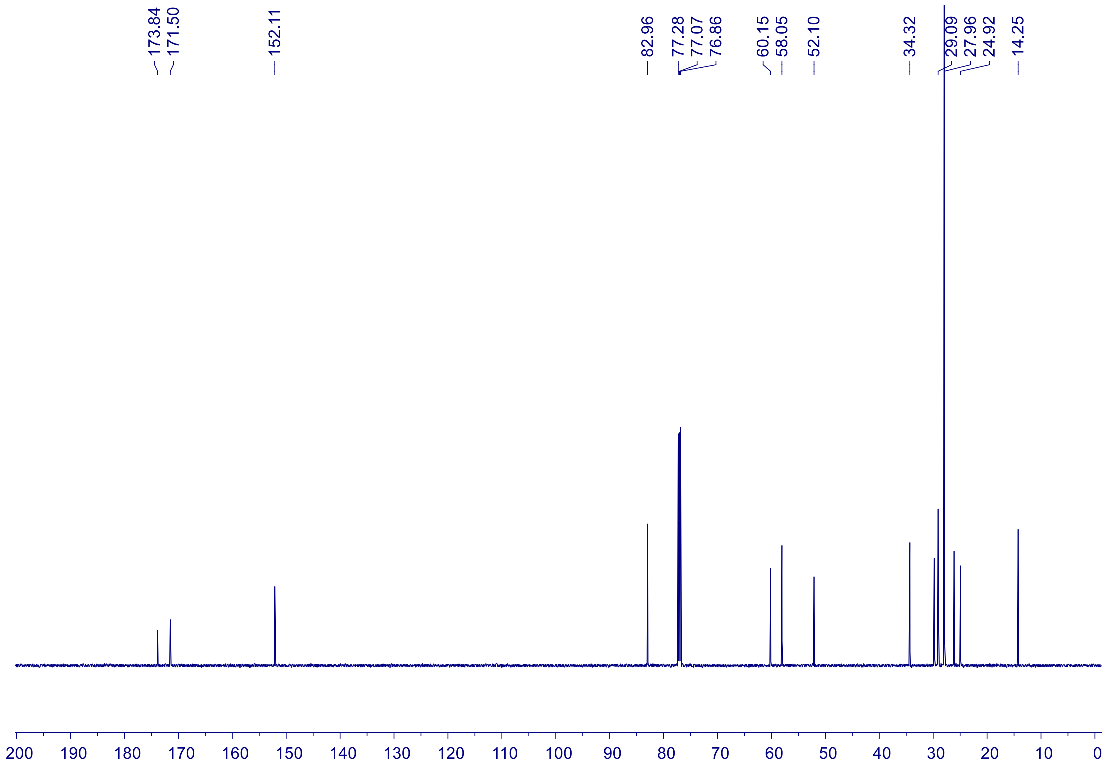

Methyl 2-(bis(tert-butoxycarbonyl)amino)-6,6,7,7,8,8,9,9,10,10,11,11,12,12,13,13,13-heptadecafluorotridecanoate (62)-^{1}H NMR (400 MHz, $CDCl_3$)

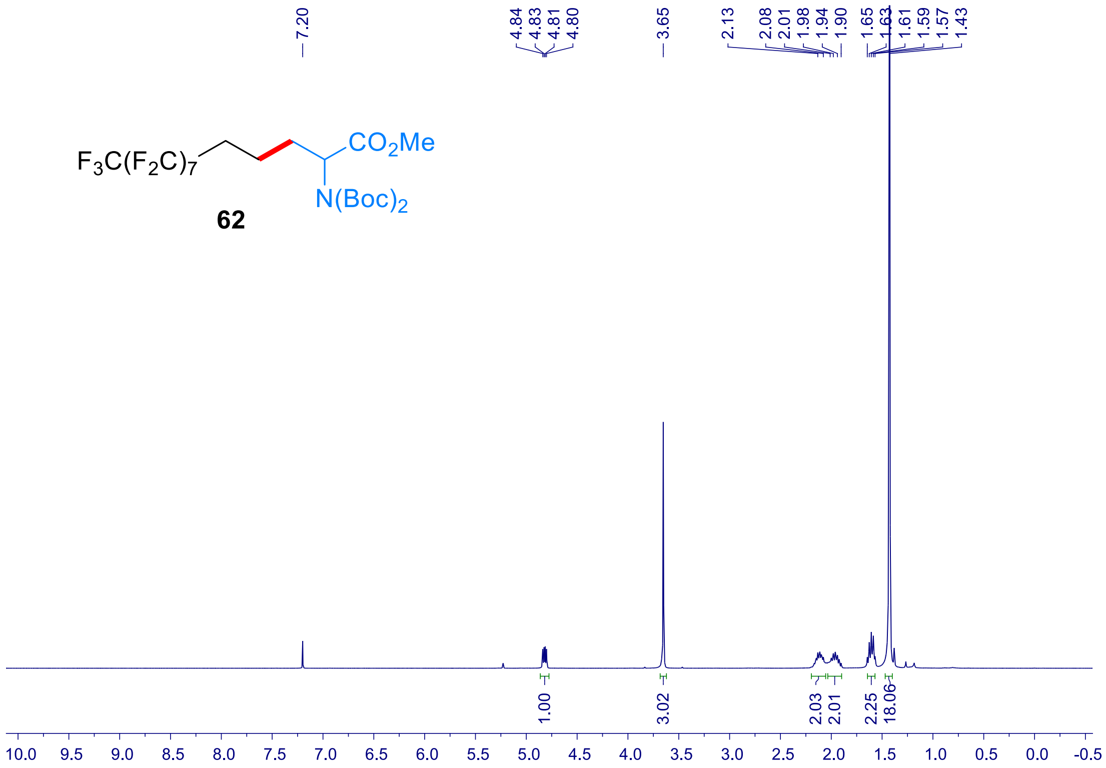

(62)-^{13}C NMR (101 MHz, $CDCl_3$)

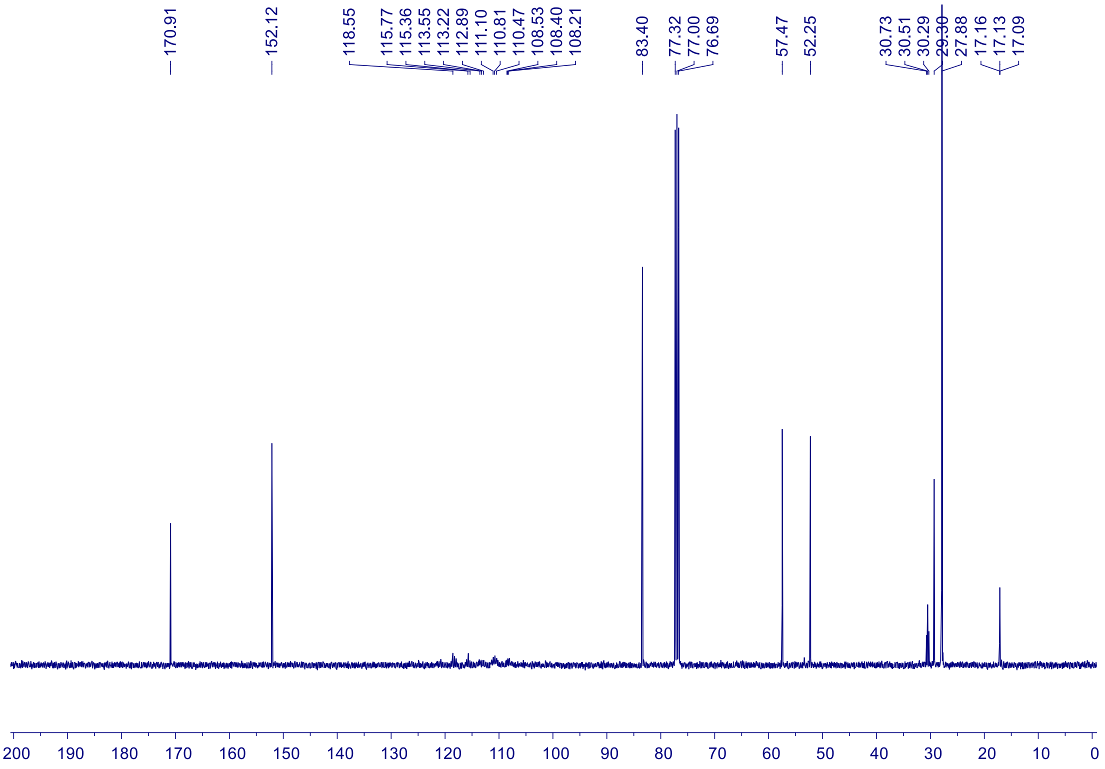

(62)-^{19}F NMR: (376 MHz, $CDCl_3$)

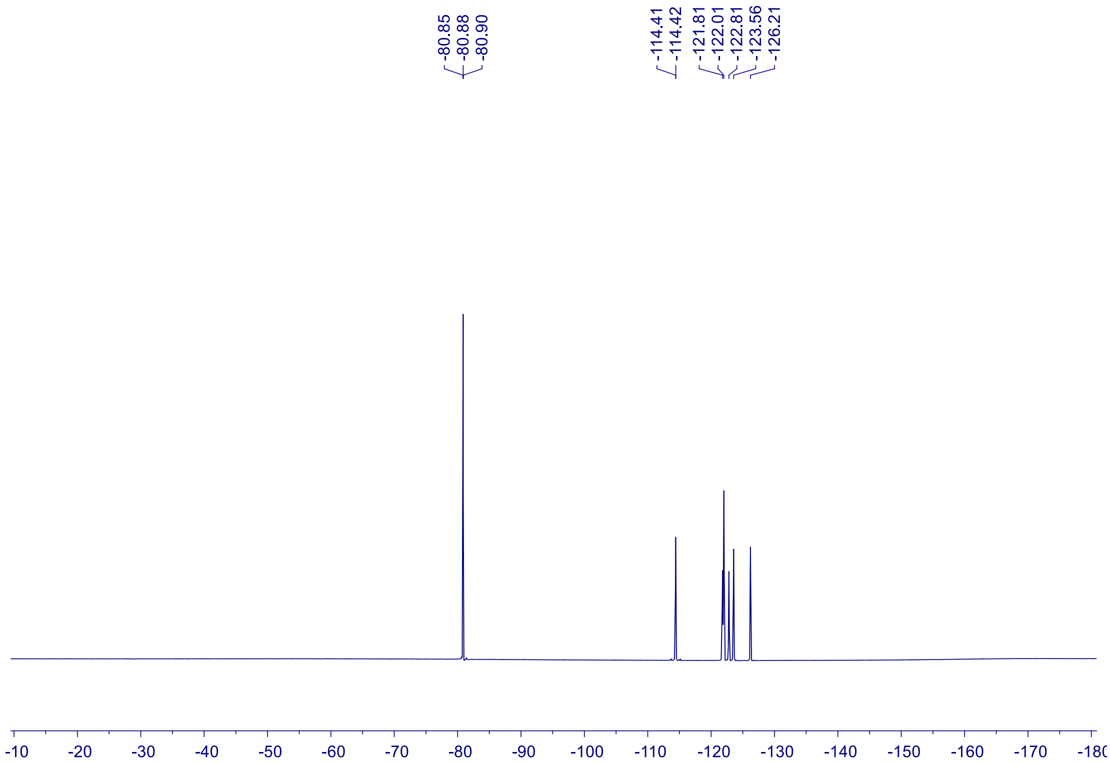

Dimethyl 2,8-bis(bis(tert-butoxycarbonyl)amino)nonanedioate (63)-^{1}H NMR (400 MHz, $CDCl_3$)

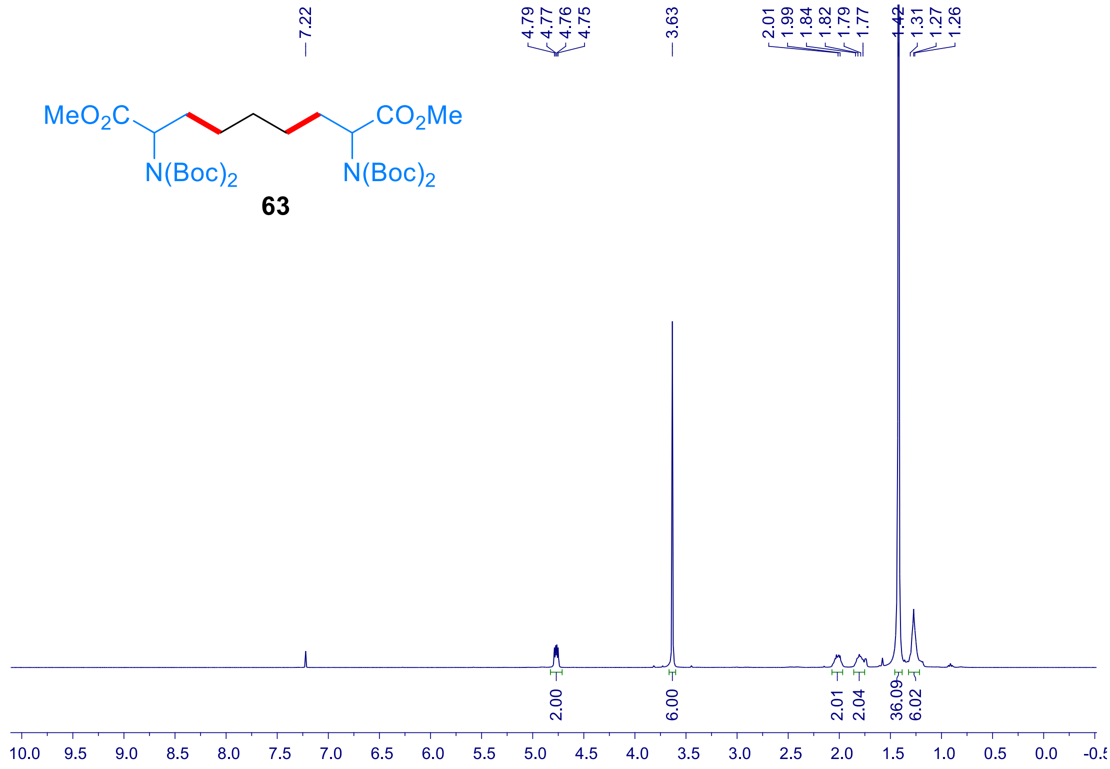

(63)-^{13}C NMR (101 MHz, $CDCl_3$)

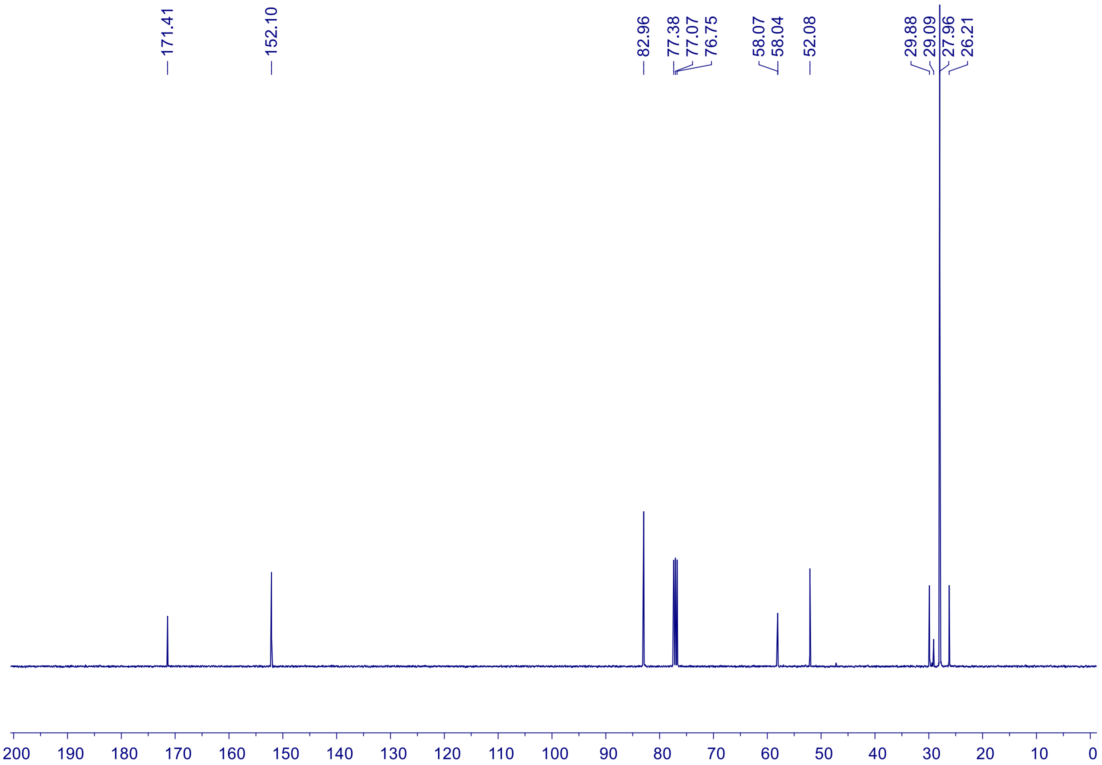

***tert*-butyl 4-(4-(bis(tert-butoxycarbonyl)amino)-5-methoxy-5-oxopentyl) piperidine-1-carboxylate (64)-**^{1}H NMR (400 MHz, $CDCl_3$)

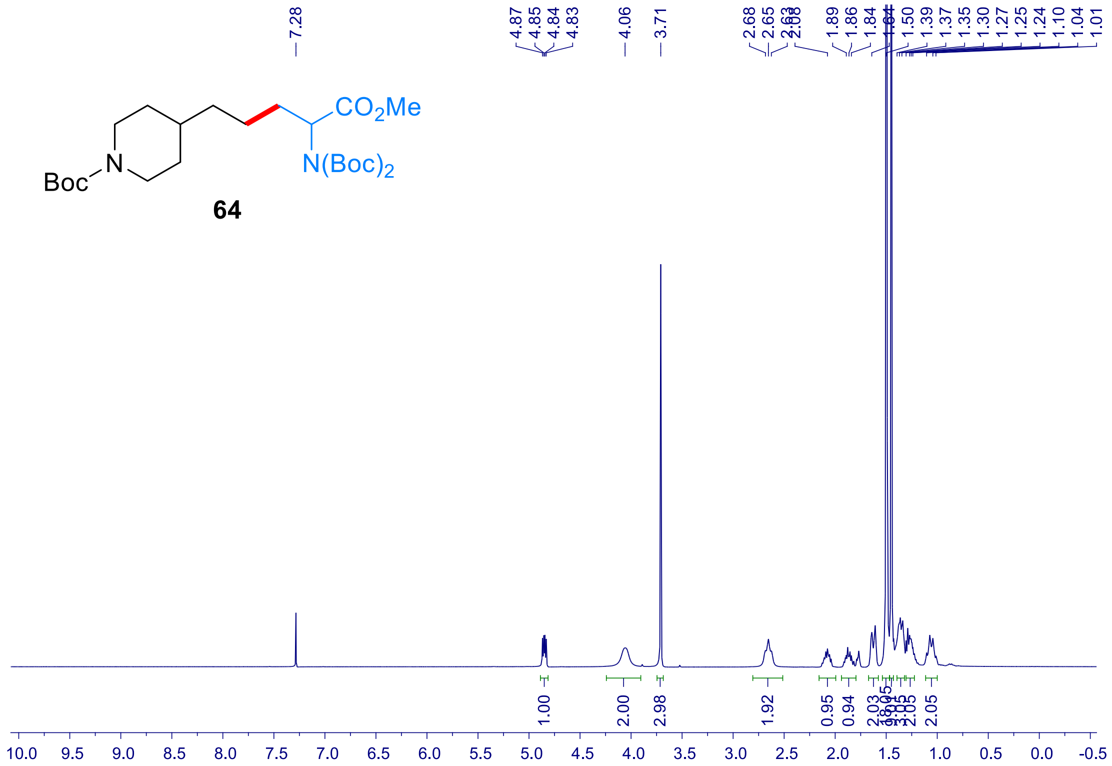

(64)-^{13}C NMR (101 MHz, $CDCl_3$)

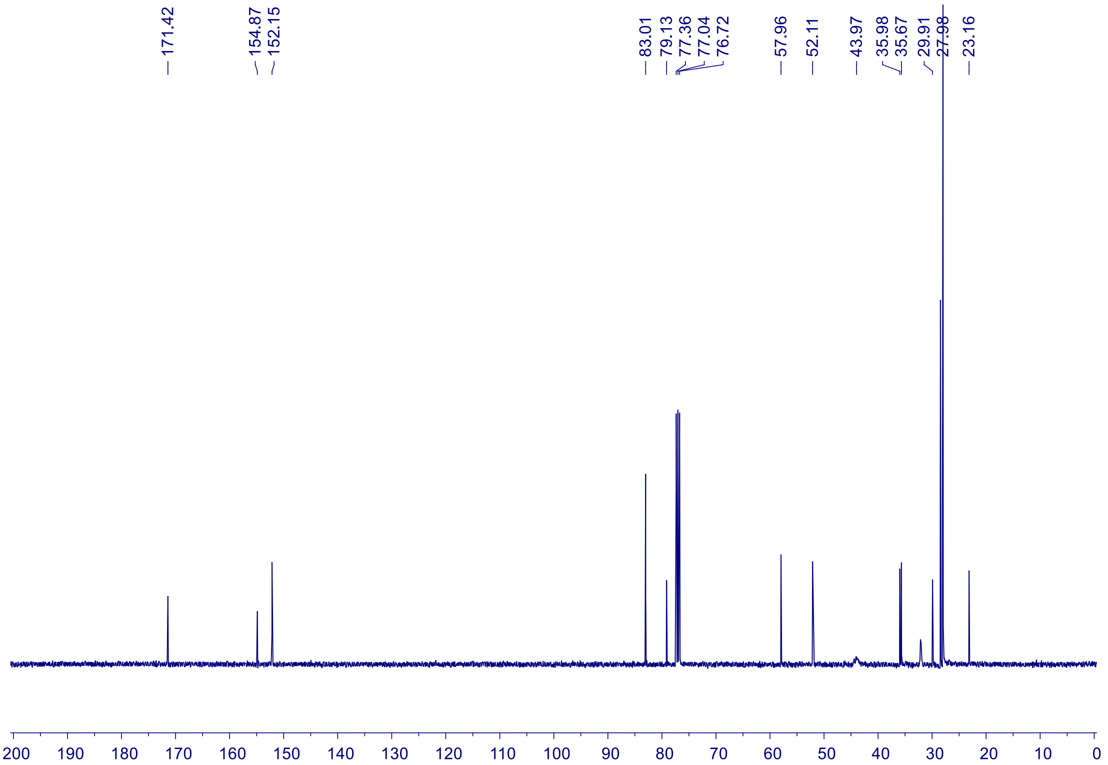

Methyl 2-(Bis(*tert*-butoxycarbonyl)amino)-3-(4-methyltetrahydro-2H-pyran-4-yl) propanoate (65)-^{1}H NMR (400 MHz, $CDCl_3$)

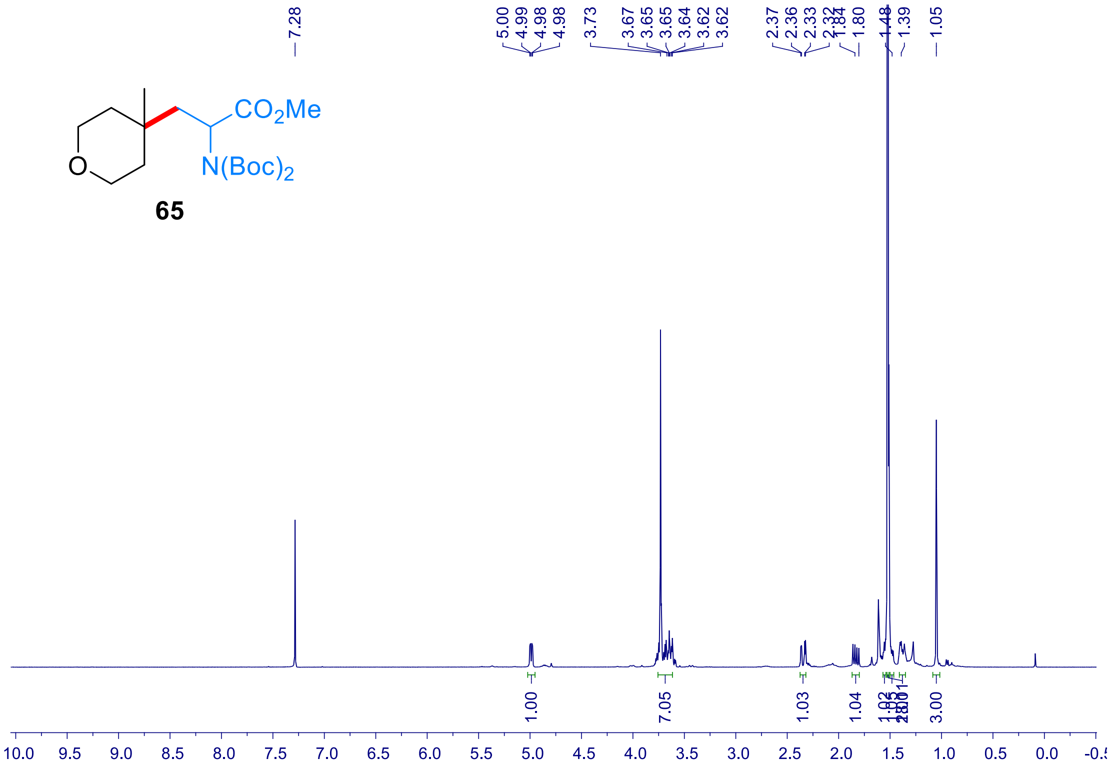

(65)-^{13}C NMR (101 MHz, $CDCl_3$)

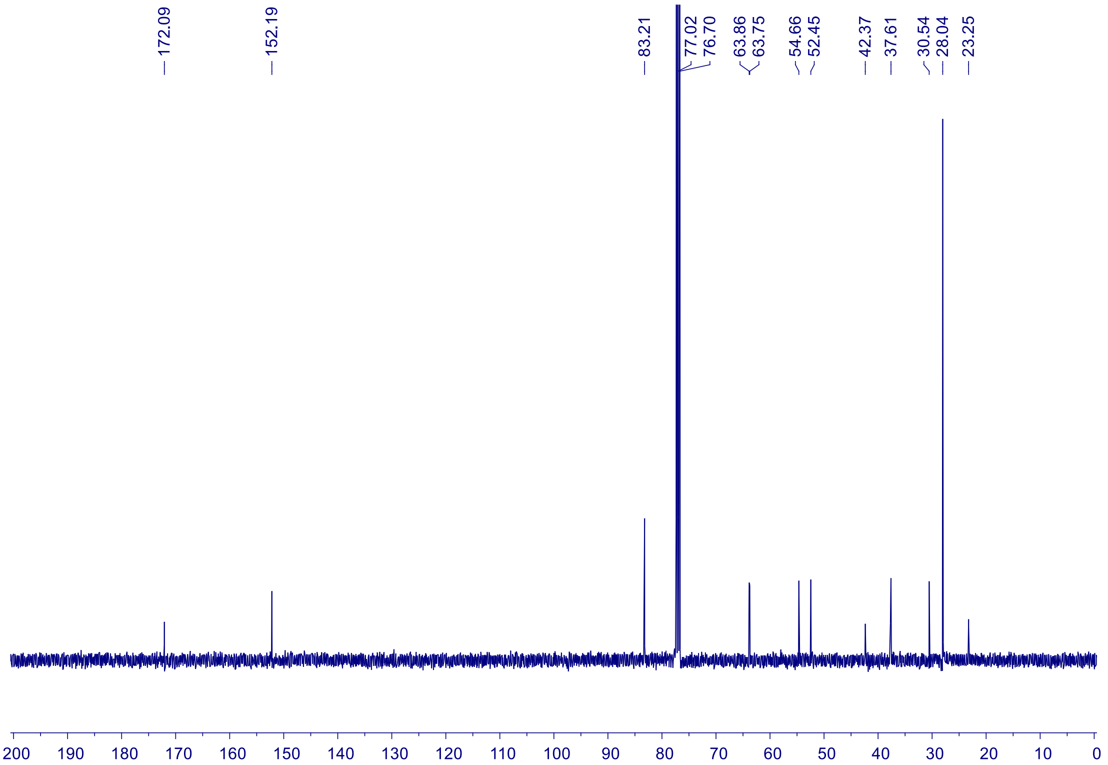

Methyl 2-(Bis(*tert*-butoxycarbonyl)amino)-3-(4-methyltetrahydro-2H-thiopyran-4-yl)propanoate (66)-^{1}H NMR (400 MHz, $CDCl_3$)

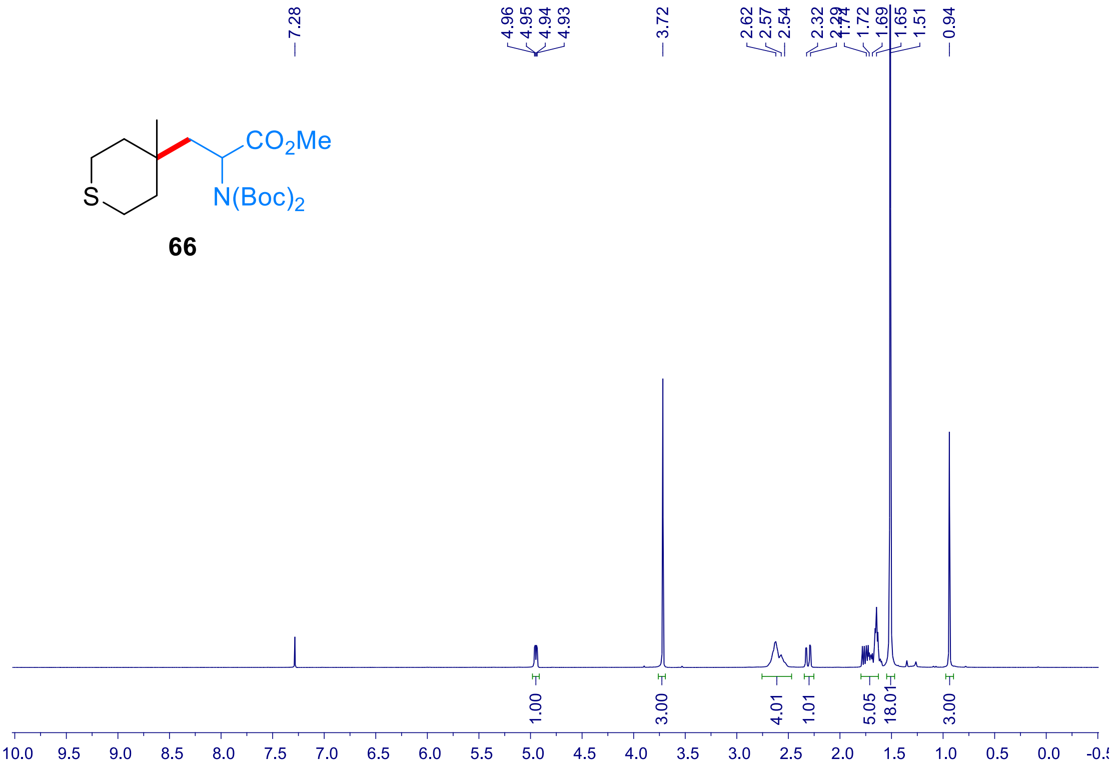

(66)-^{13}C NMR (101 MHz, $CDCl_3$)

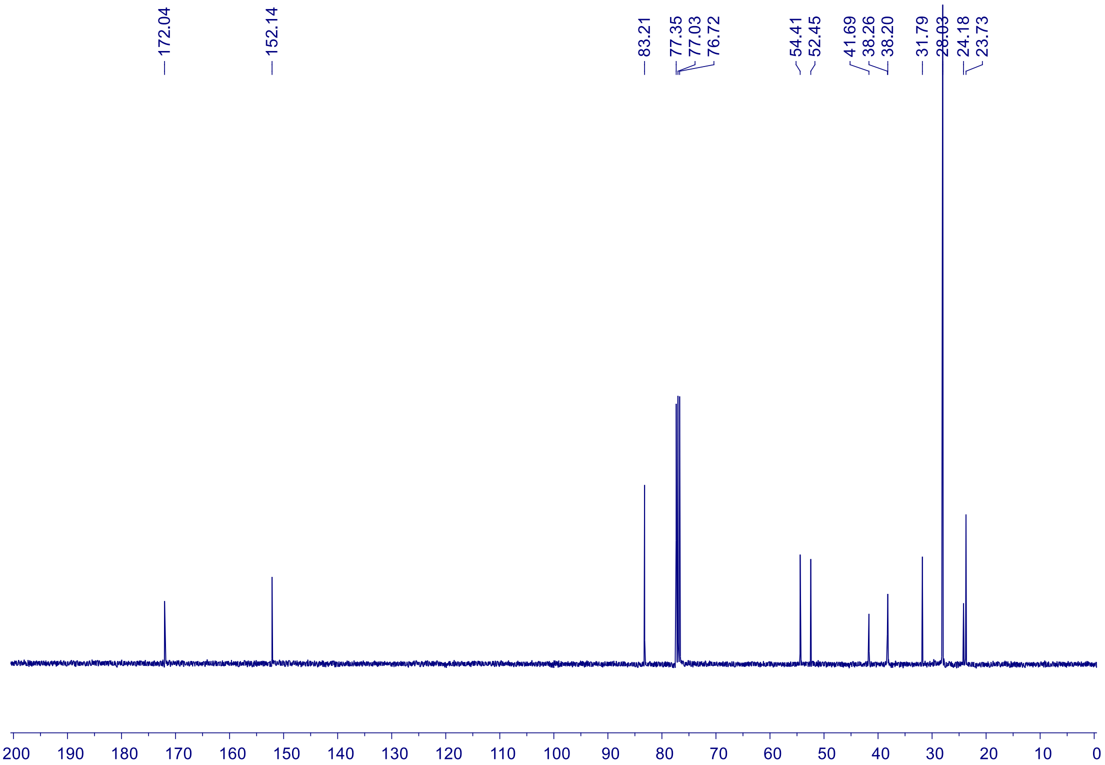

Methyl 2-(Bis(tert-butoxycarbonyl)amino)-3-(1-tosyloctahydro-3aH-indol-3a-yl) propanoate (67)-^{1}H NMR (400 MHz, $CDCl_3$)

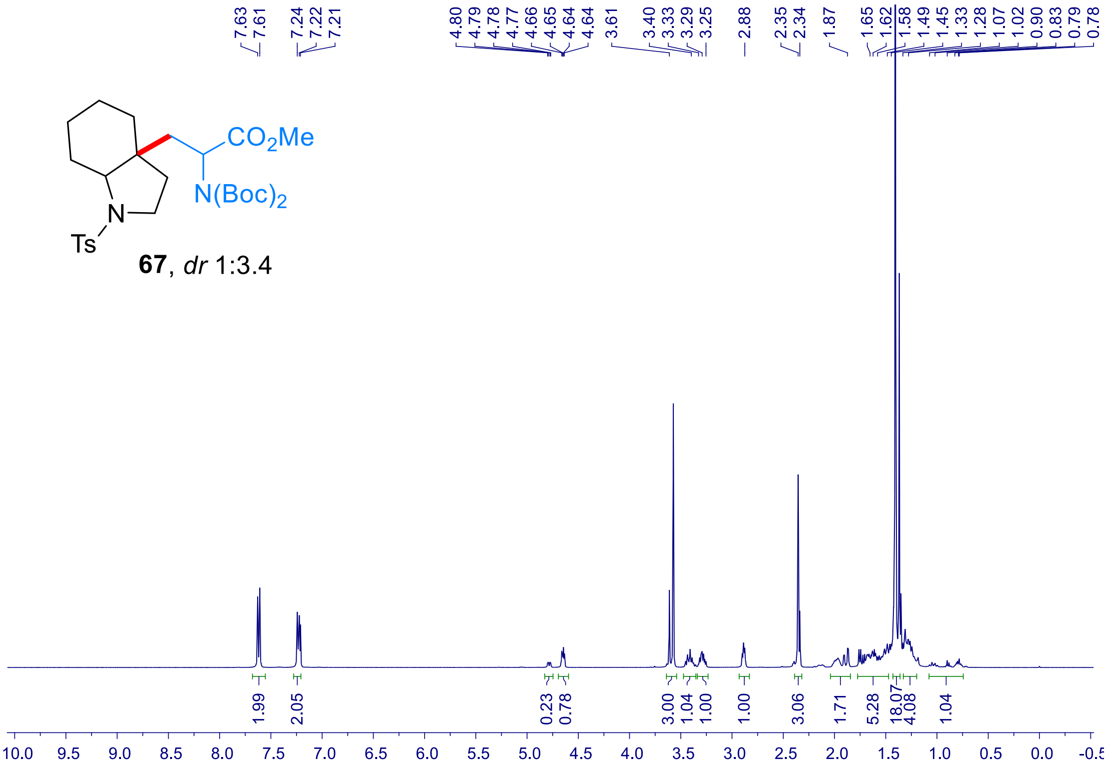

(67)-^{13}C NMR (101 MHz, $CDCl_3$)

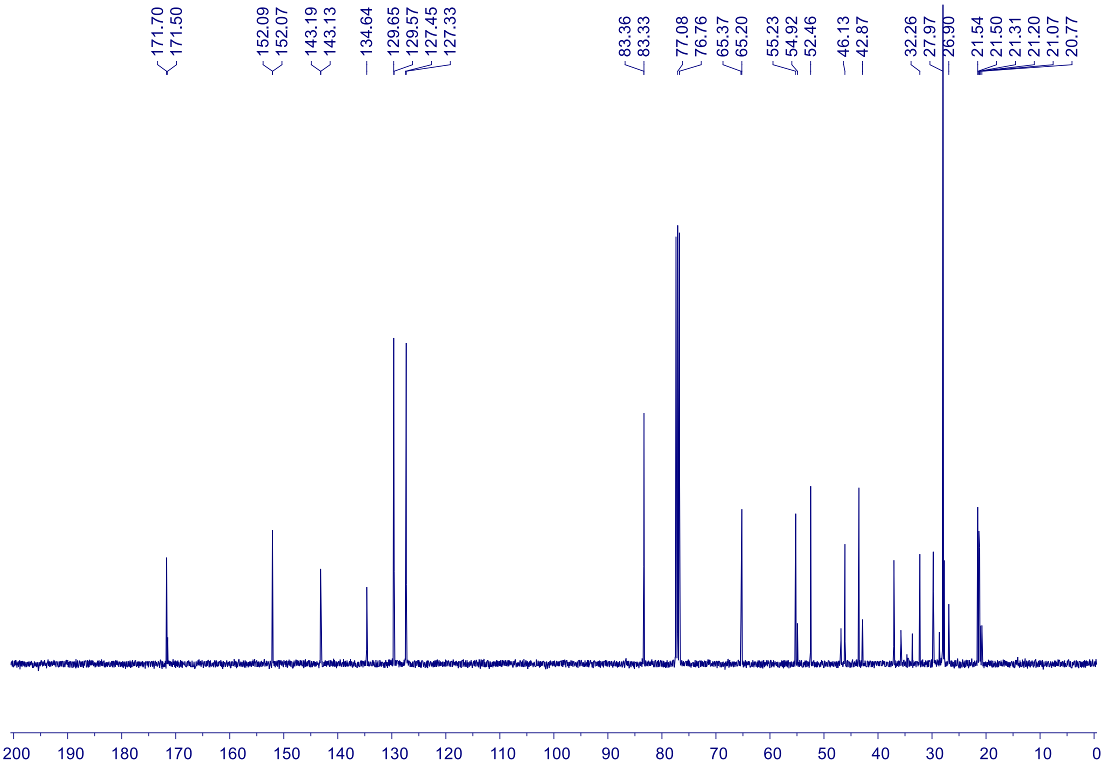

Methyl 3-(Adamantan-1-yl)-2-(bis(*tert*-butoxycarbonyl)amino)propanoate (68)-^{1}H NMR (400 MHz, $CDCl_3$)

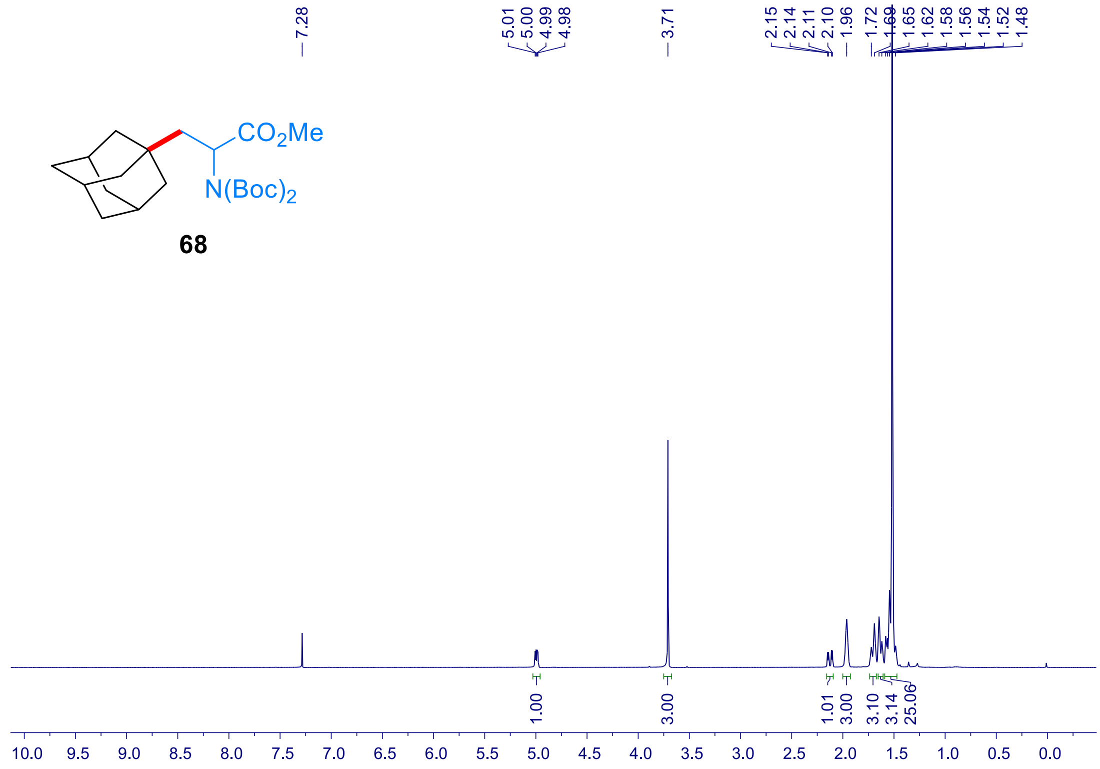

(68)-^{13}C NMR (101 MHz, $CDCl_3$)

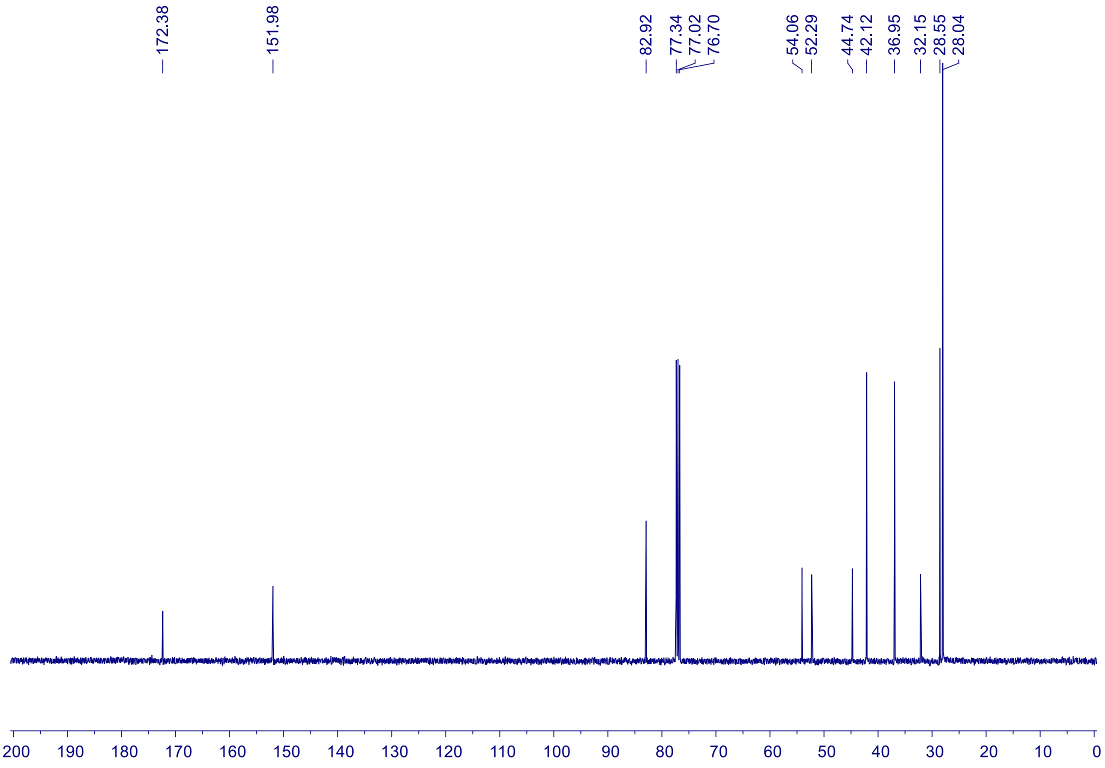

3-(1-tosyloctahydro-3aH-indol-3a-yl)propanenitrile (69)-^{1}H NMR (400 MHz, $CDCl_3$)

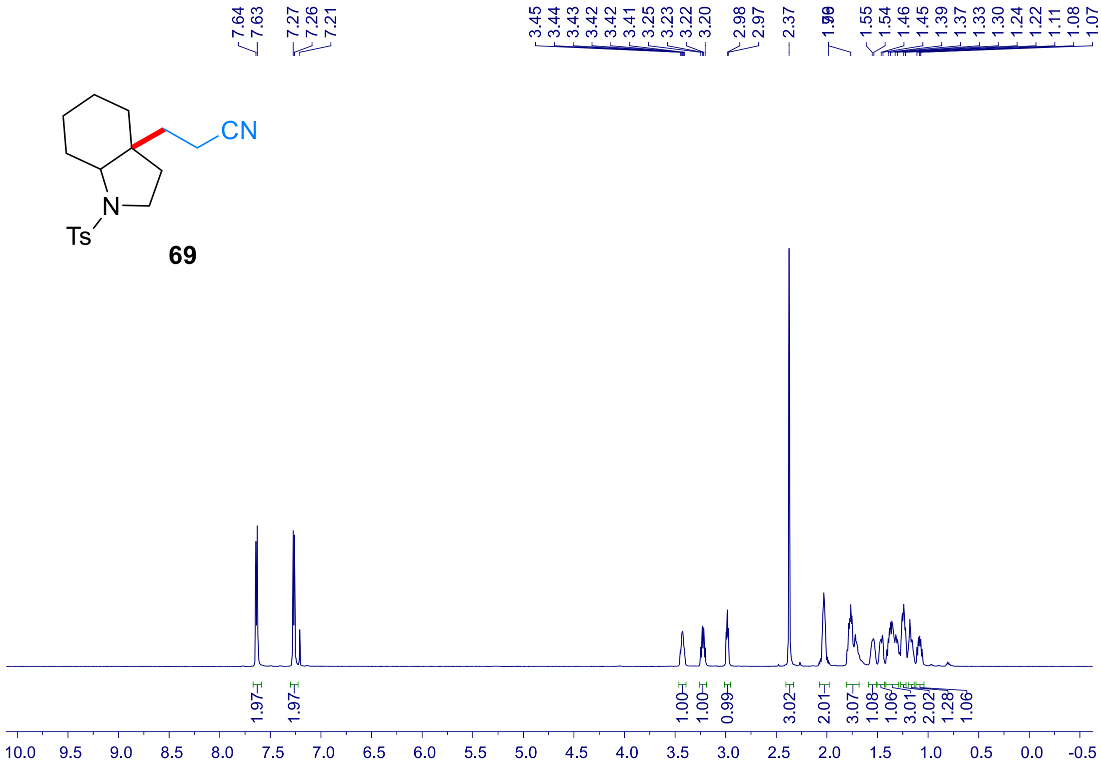

(69)-^{13}C NMR (101 MHz, $CDCl_3$)

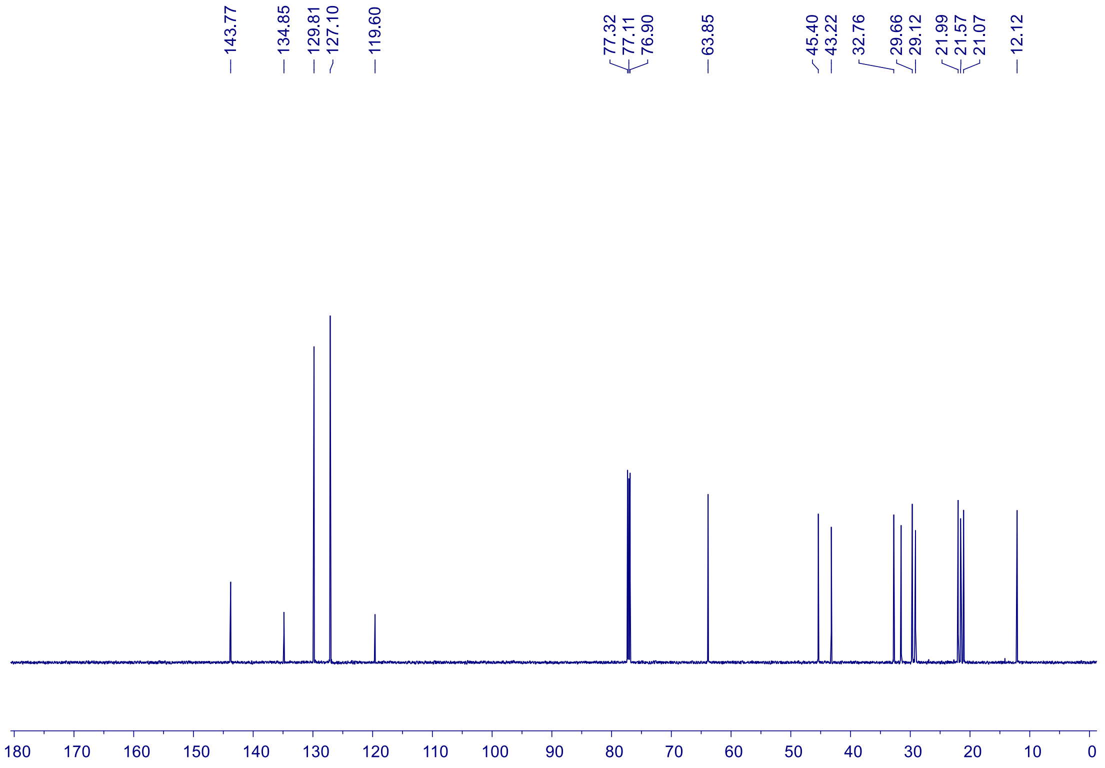

***tert*-butyl 3-(1-tosyloctahydro-3aH-indol-3a-yl)propanoate (70)**-^{1}H NMR (400 MHz, $CDCl_3$)

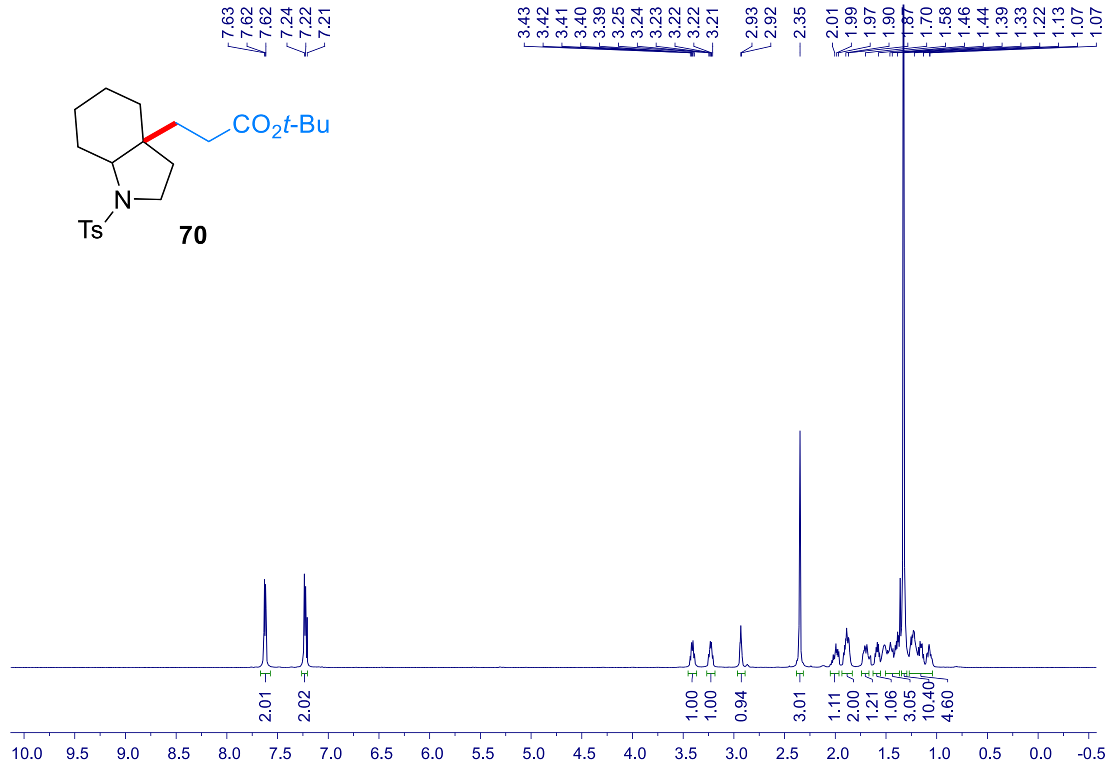

(70)-^{13}C NMR (101 MHz, $CDCl_3$)

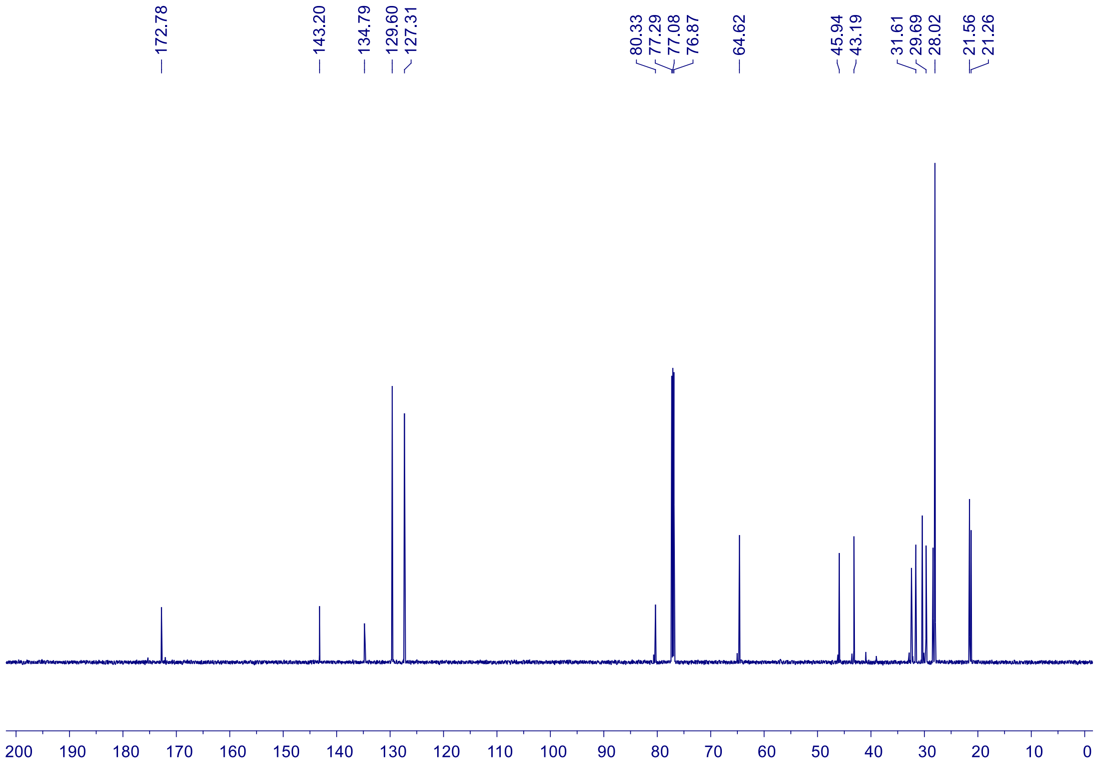

***tert*-butyl 3-(3-(2-((*1S,5R*)-6,6-dimethylbicyclo[3.1.1]hept-2-en-3-yl)ethoxy)-3-oxopropyl)azetidine-1-carboxylate (71)**-^{1}H NMR (400 MHz, $CDCl_3$)

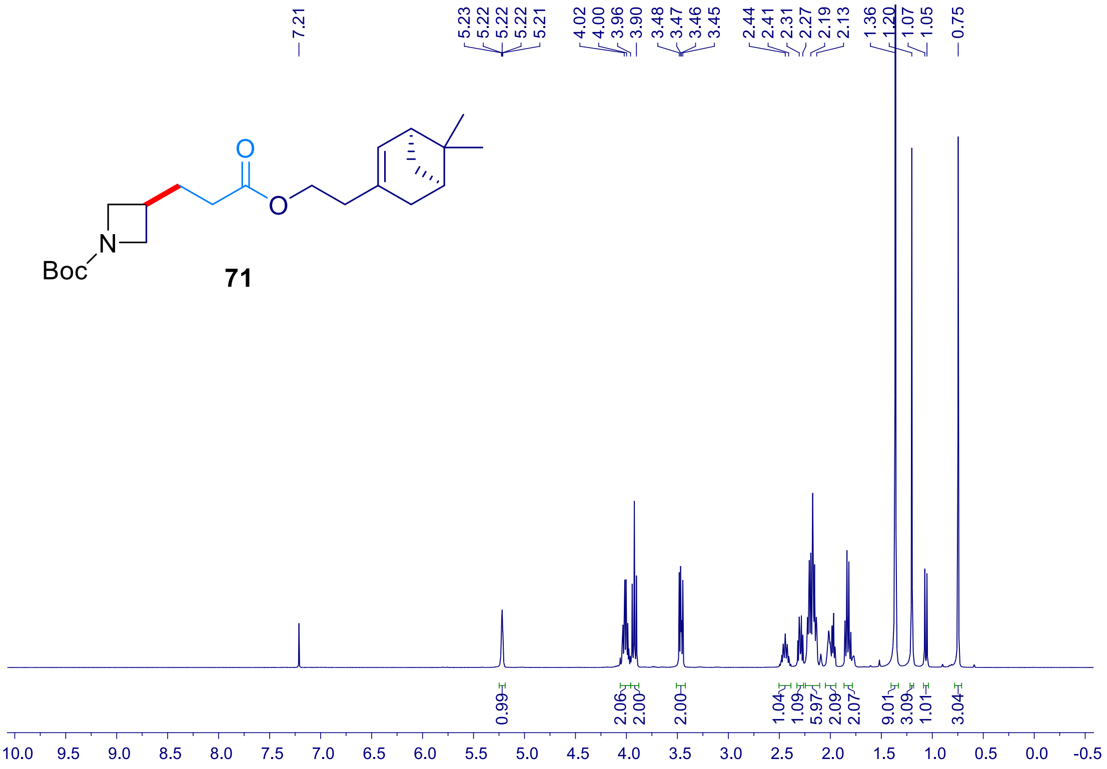

(71)-^{13}C NMR (101 MHz, $CDCl_3$)

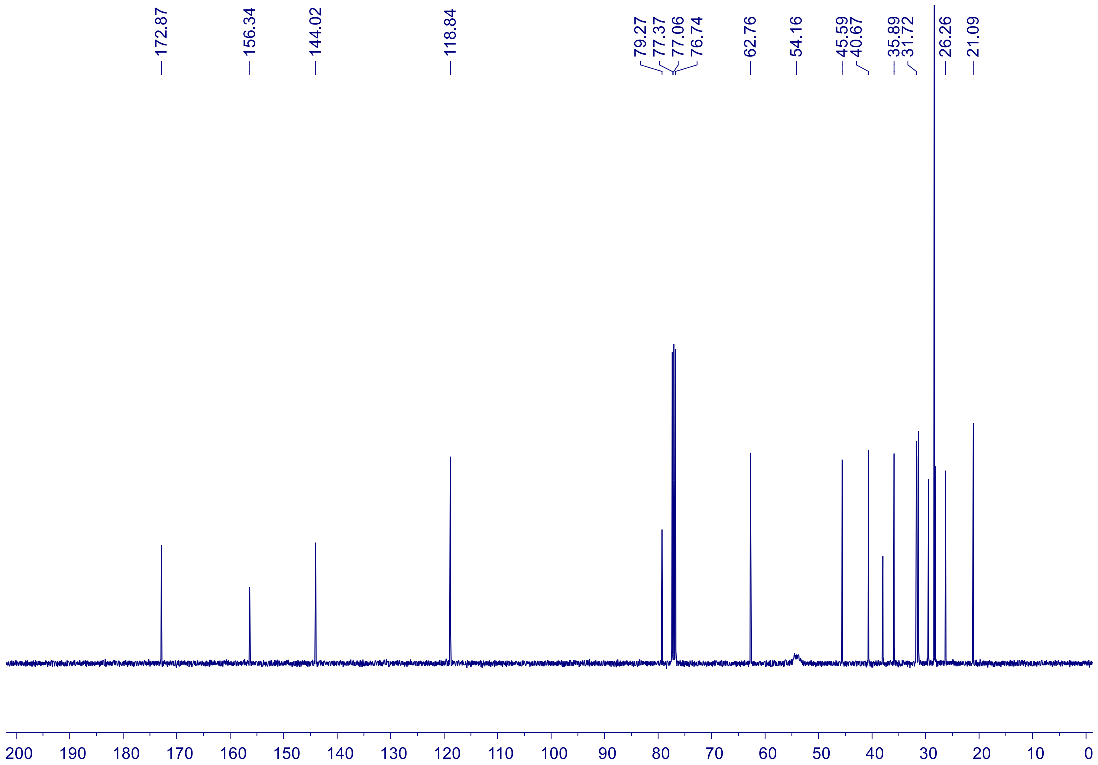

***tert*-butyl (*R*)-3-(3-((3,7-dimethyloct-6-en-1-yl)oxy)-3-oxopropyl)azetidine-1-carboxylate (72)-**^{1}H NMR (400 MHz, $CDCl_3$)

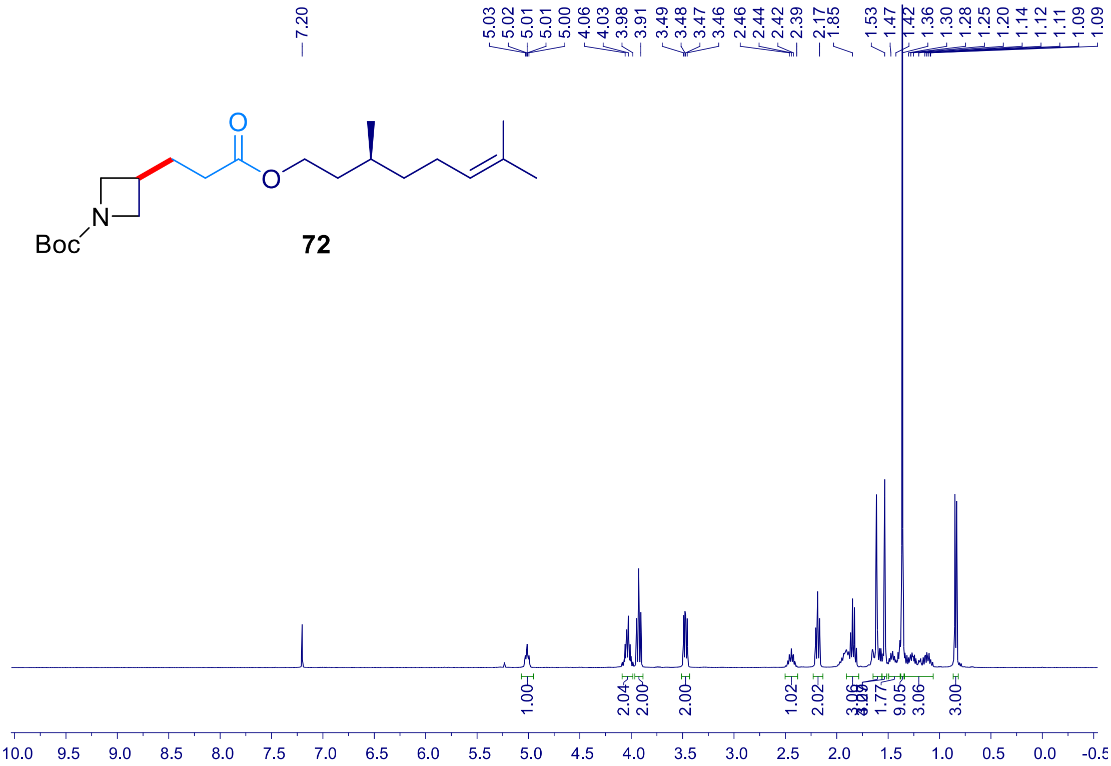

(72)-^{13}C NMR (101 MHz, $CDCl_3$)

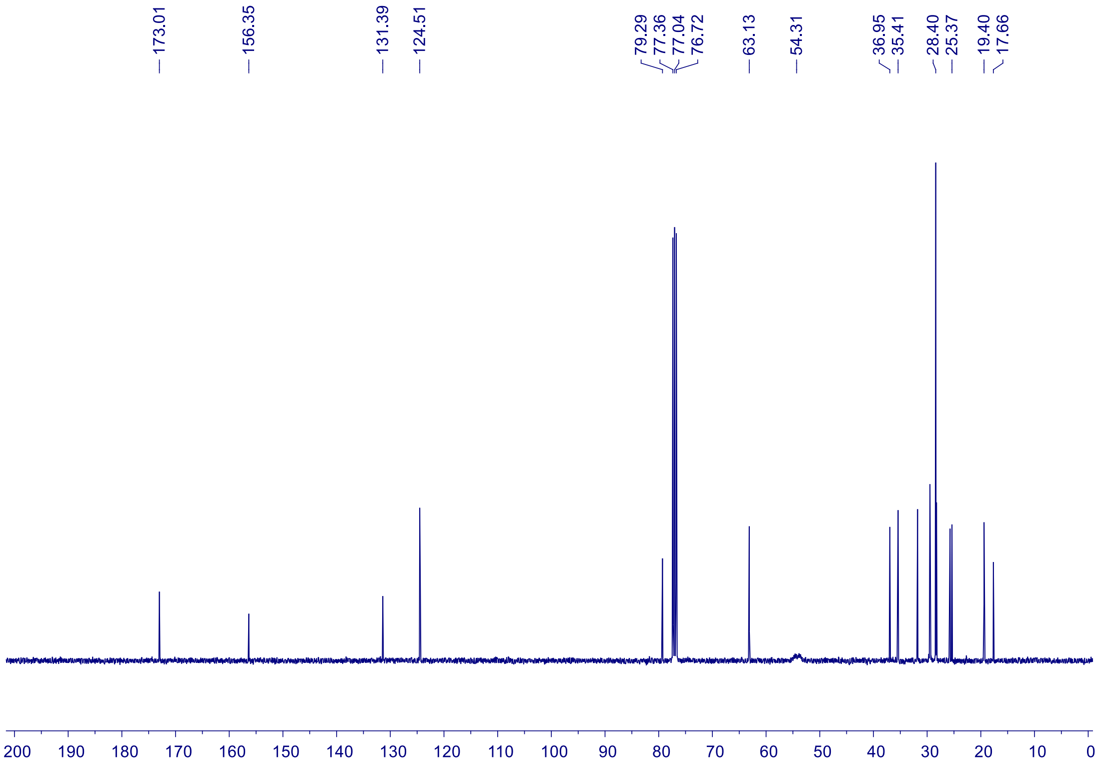

***tert*-butyl 3-(3-((2-(4-methylcyclohex-3-en-1-yl)propan-2-yl)oxy)-3-oxopropyl) azetidine-1-carboxylate (73)**-^{1}H NMR (400 MHz, $CDCl_3$)

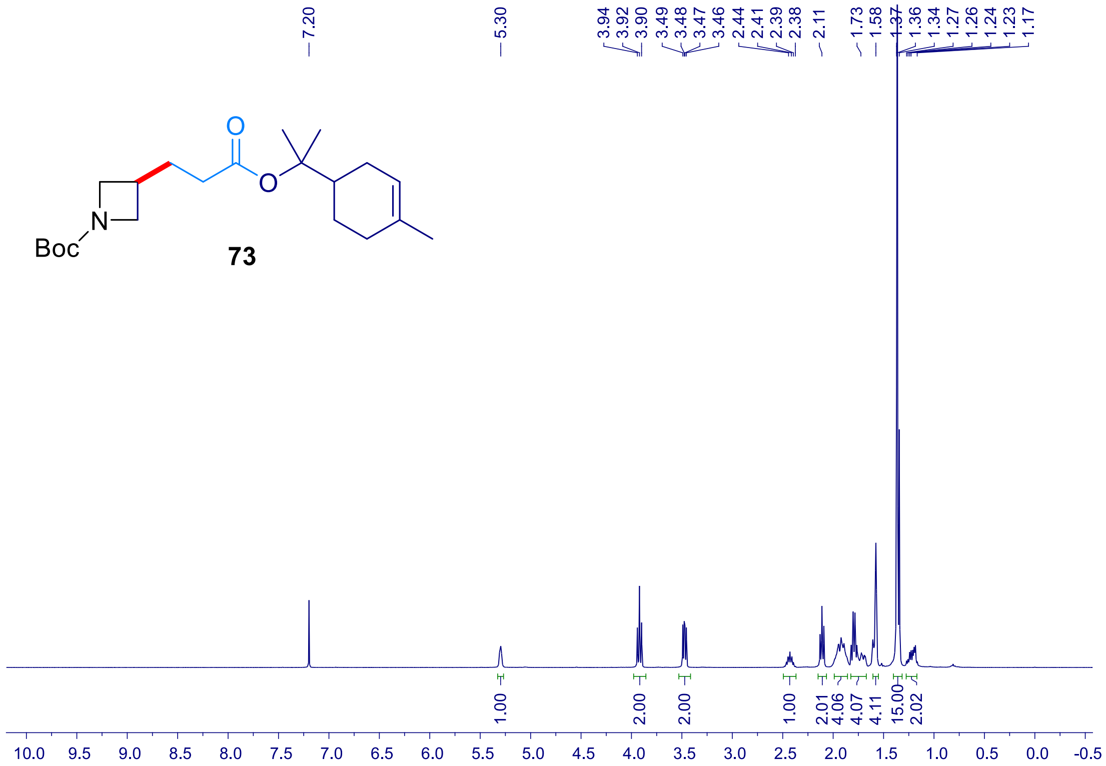

(73)-^{13}C NMR (101 MHz, $CDCl_3$)

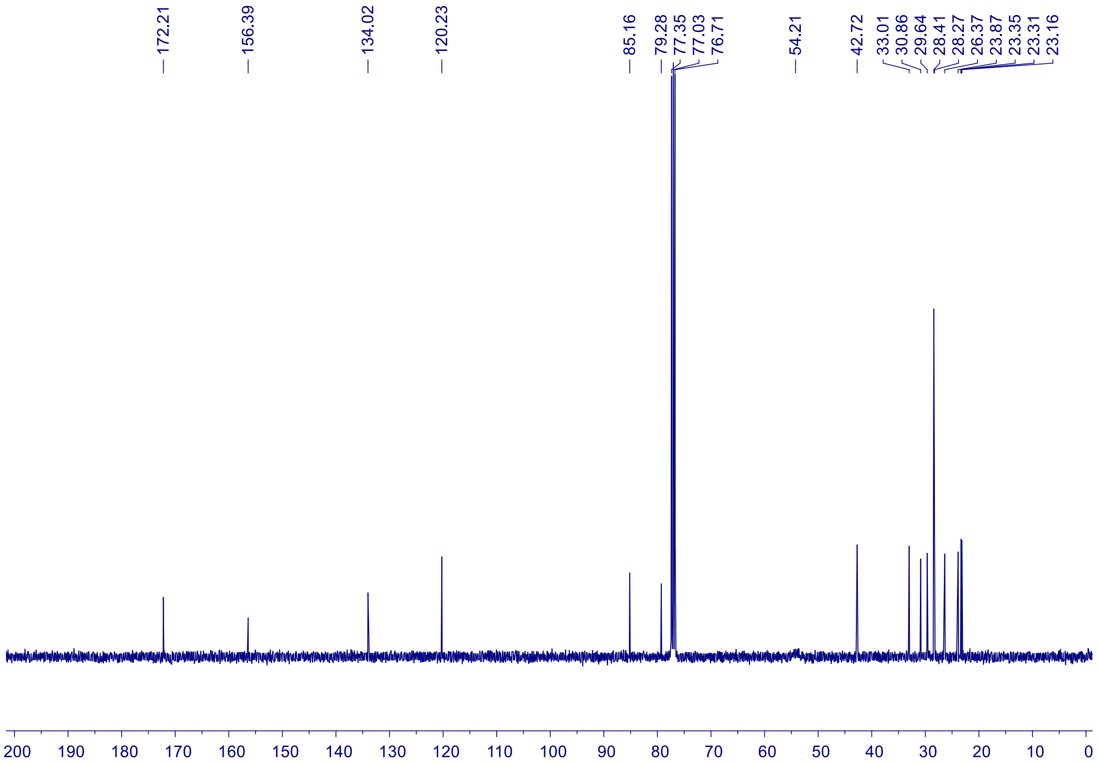

***tert*-butyl (*E*)-3-(3-((3,7-dimethylocta-2,6-dien-1-yl)oxy)-3-oxopropyl)azetidine-1-carboxylate (74)**-^{1}H NMR (400 MHz, $CDCl_3$)

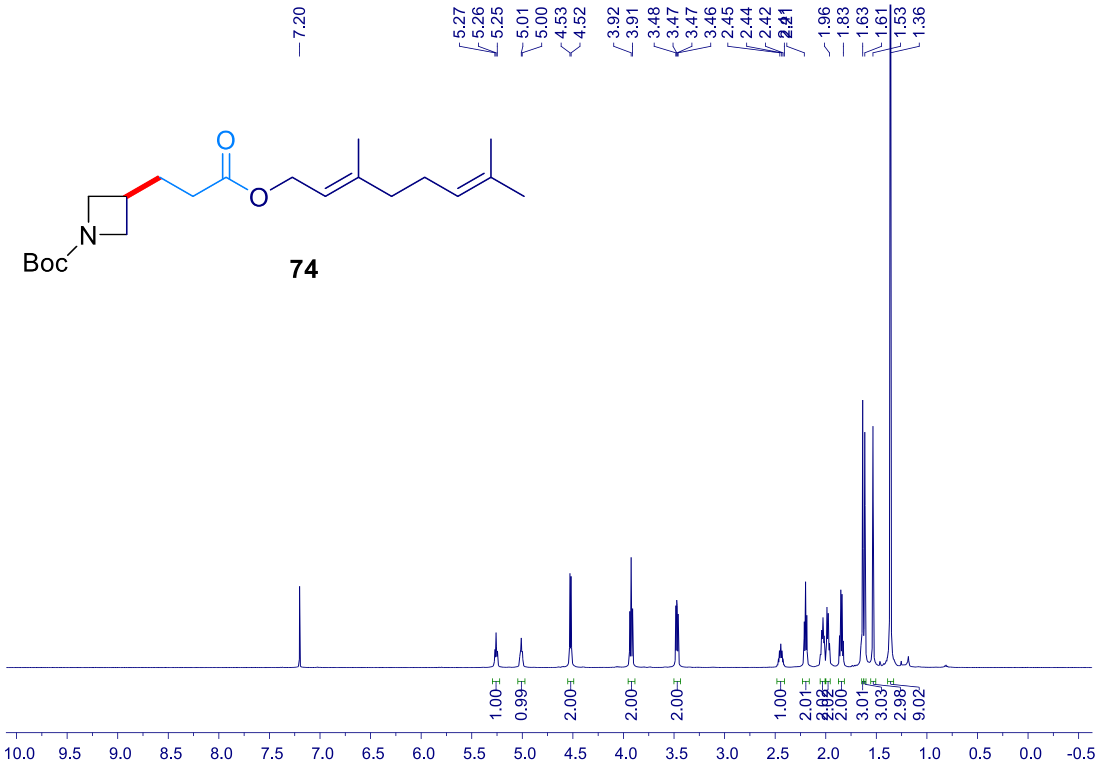

(74)-^{13}C NMR (101 MHz, $CDCl_3$)

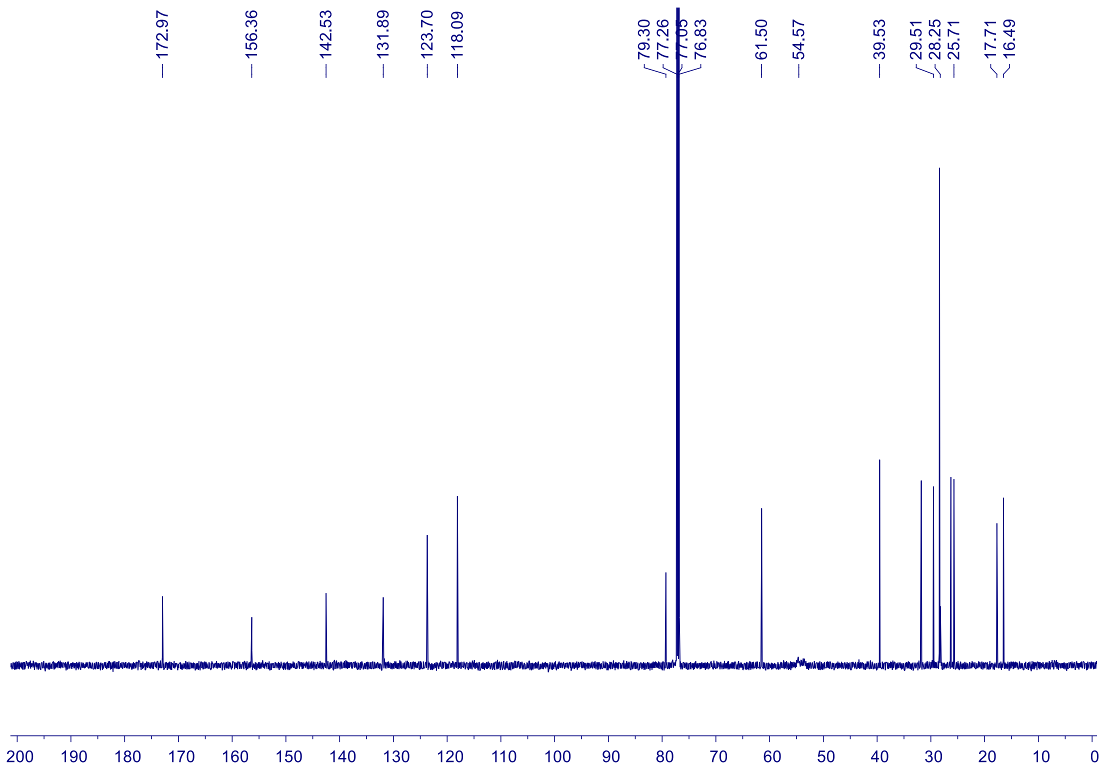

***tert*-butyl 3-(3-oxo-3-(((*3R,3aS,6R,7R,8aS*)-3,6,8,8-tetramethyloctahydro-1H-3a,7-methanoazulen-6-yl)oxy)propyl)azetidine-1-carboxylate (75)**-^{1}H NMR (400 MHz, $CDCl_3$)

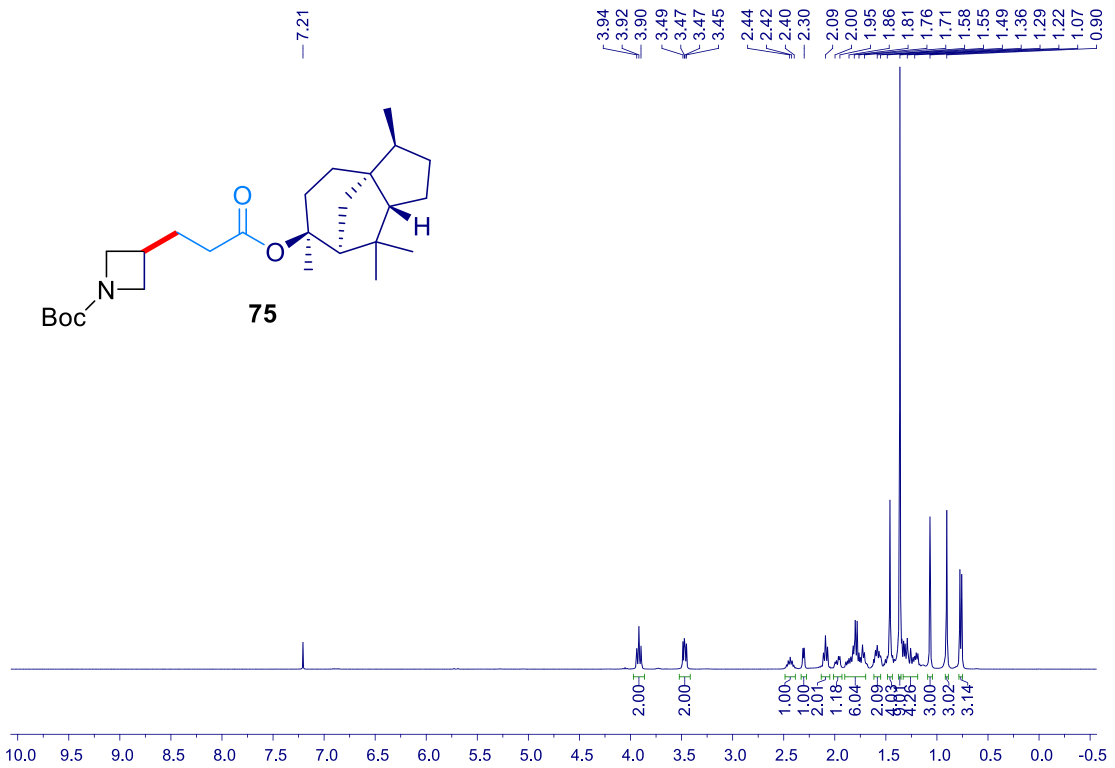

75

(75)-^{13}C NMR (101 MHz, $CDCl_3$)

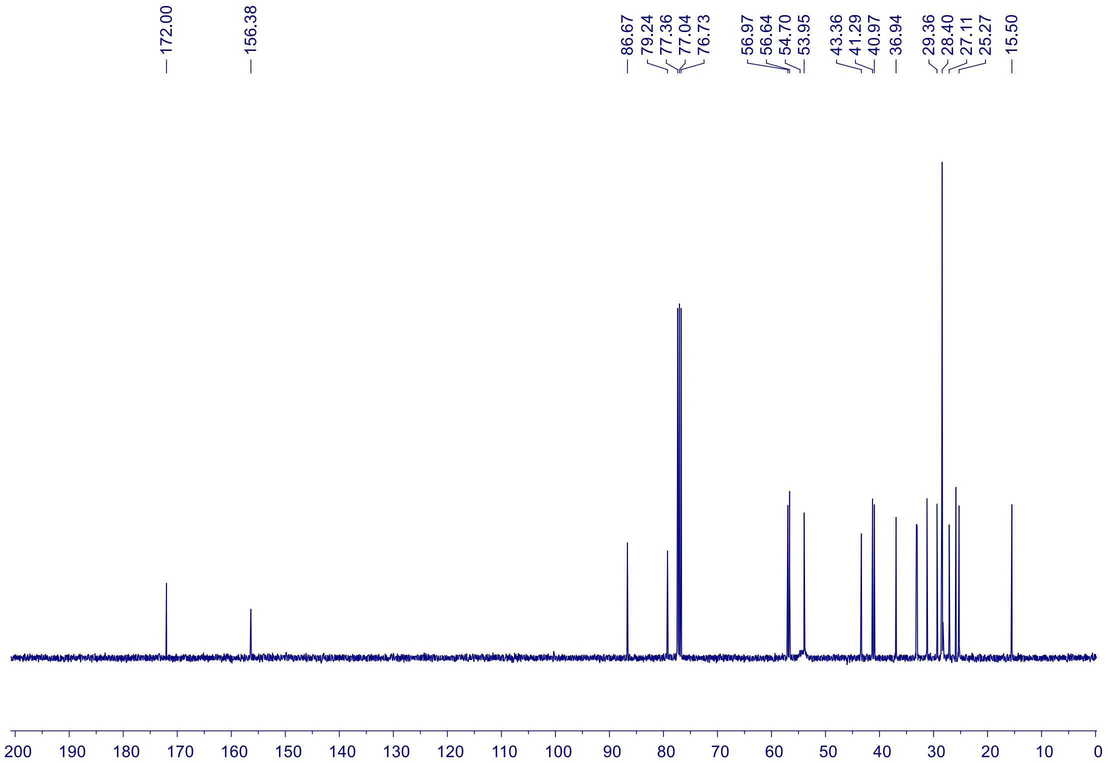

***tert*-butyl 3-(3-oxo-3-(((*1R,2R,4S*)-1,3,3-trimethylbicyclo[2.2.1]heptan-2-yl)oxy) propyl)azetidine-1-carboxylate (76)**-^{1}H NMR (400 MHz, $CDCl_3$)

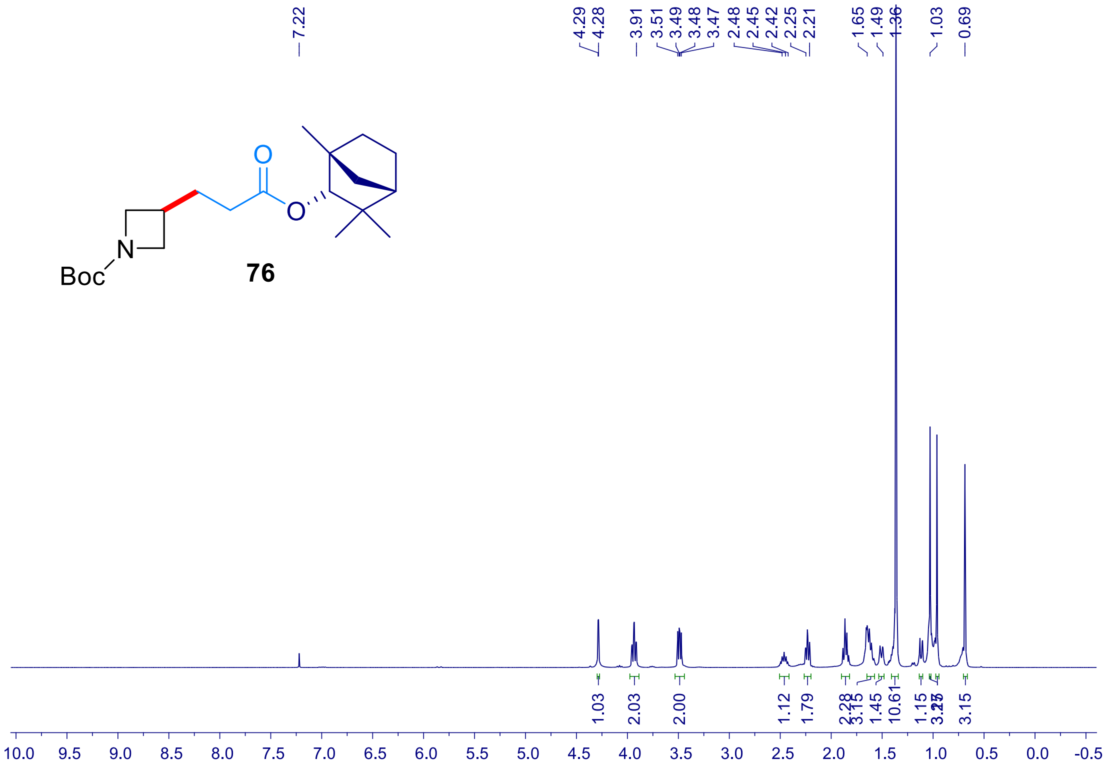

(76)-^{13}C NMR (101 MHz, $CDCl_3$)

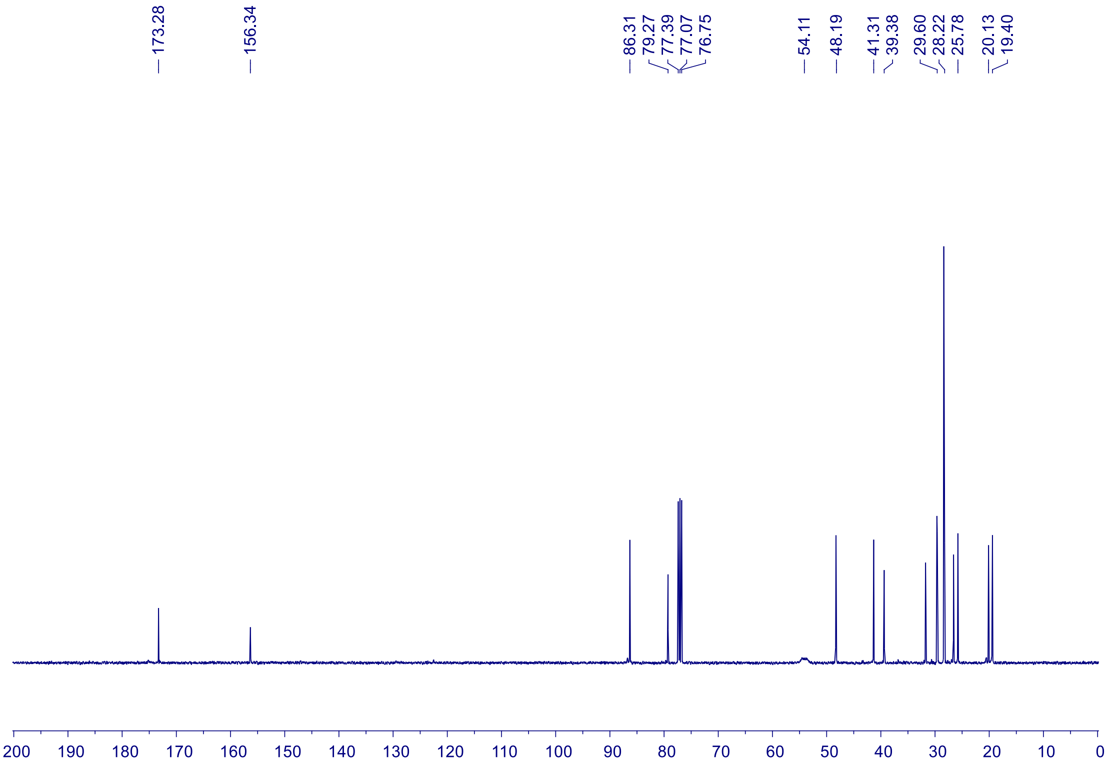

Methyl 2-(Bis(*tert*-butoxycarbonyl)amino)-5-((1S,5R)-6,6-dimethylbicyclo[3.1.1] hept-3-ene-3-yl)pentanoate (77)-^{1}H NMR (400 MHz, $CDCl_3$)

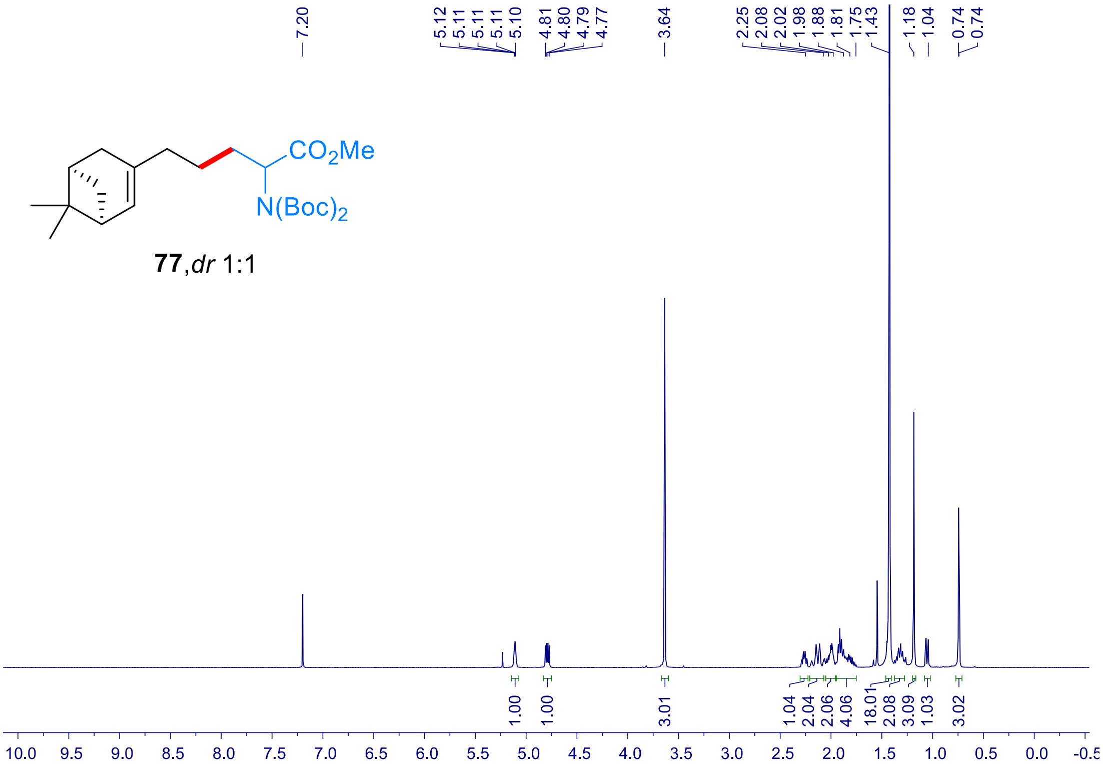

(77)-^{13}C NMR (101 MHz, $CDCl_3$)

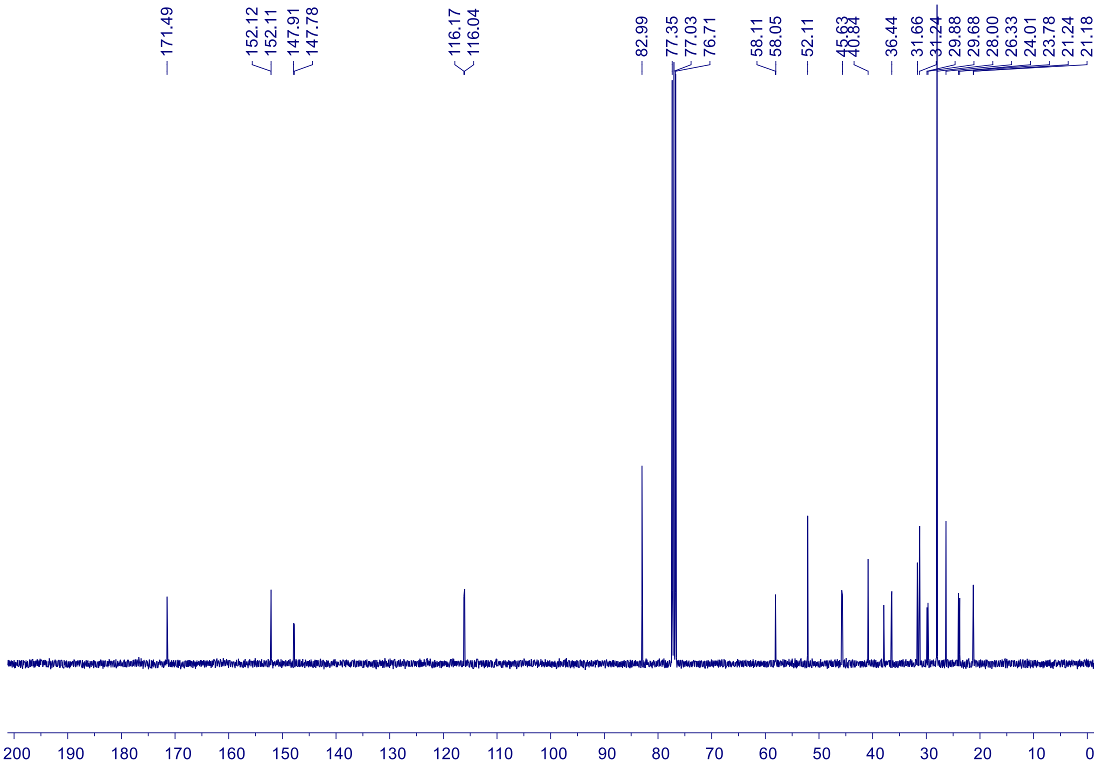

Methyl 2-(bis(tert-butoxycarbonyl)amino) (*S*)-6,10-dimethylundec-9-enoate (78)-^{1}H NMR (400 MHz, $CDCl_3$)

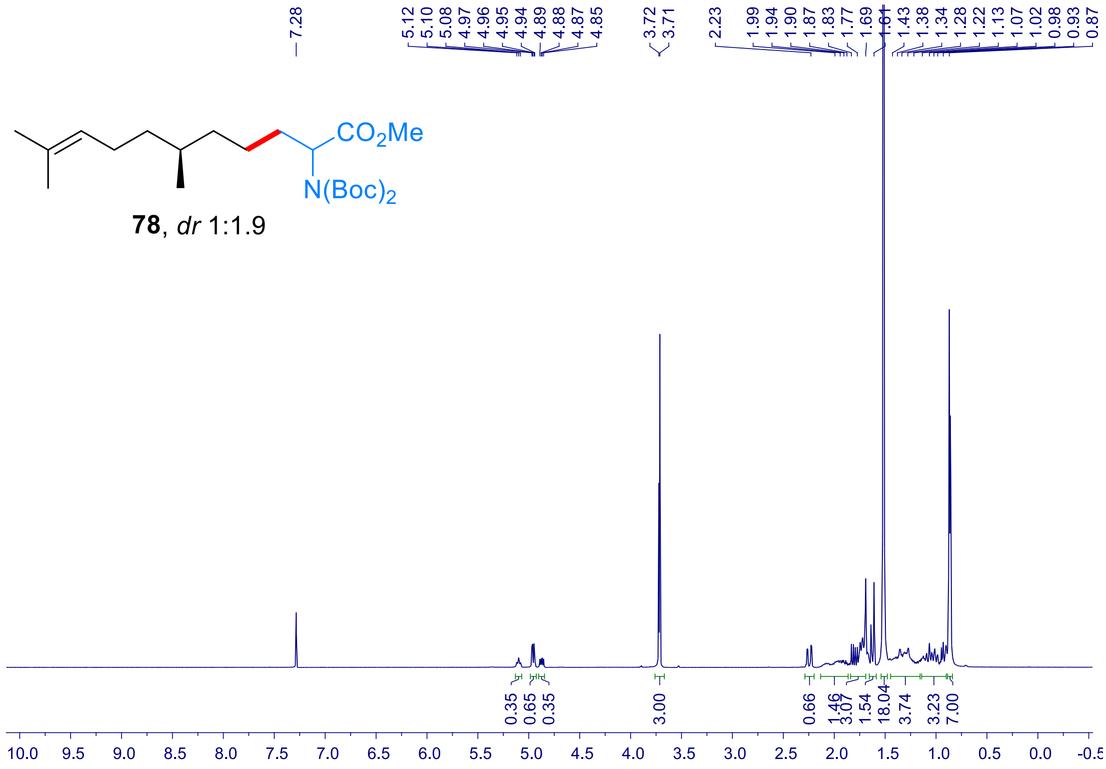

78, *dr* 1:1.9

(78)-^{13}C NMR (101 MHz, $CDCl_3$)

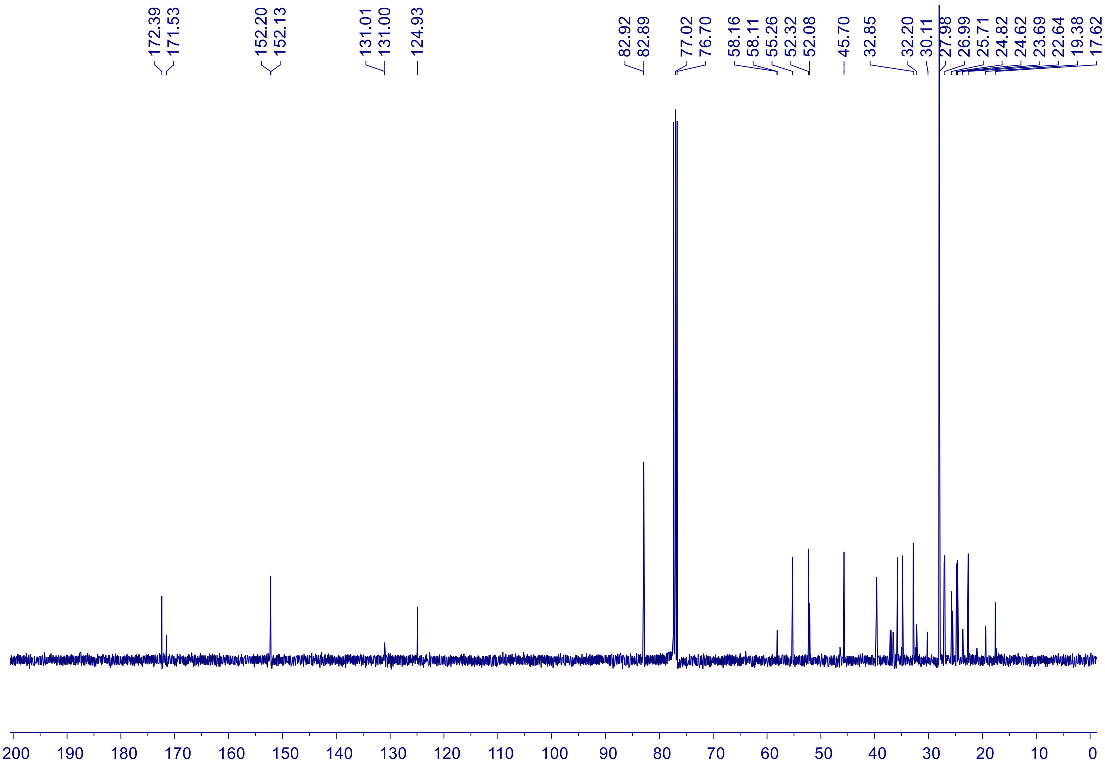

Methyl 2-(bis(tert-butoxycarbonyl)amino)-4-((3aR,4R,6R,6aR)-6-methoxy-2,2-dimethyltetrahydrofuro[3,4-d][1,3]dioxol-4-yl)butanoate (79)-^{1}H NMR (400 MHz, $CDCl_3$)

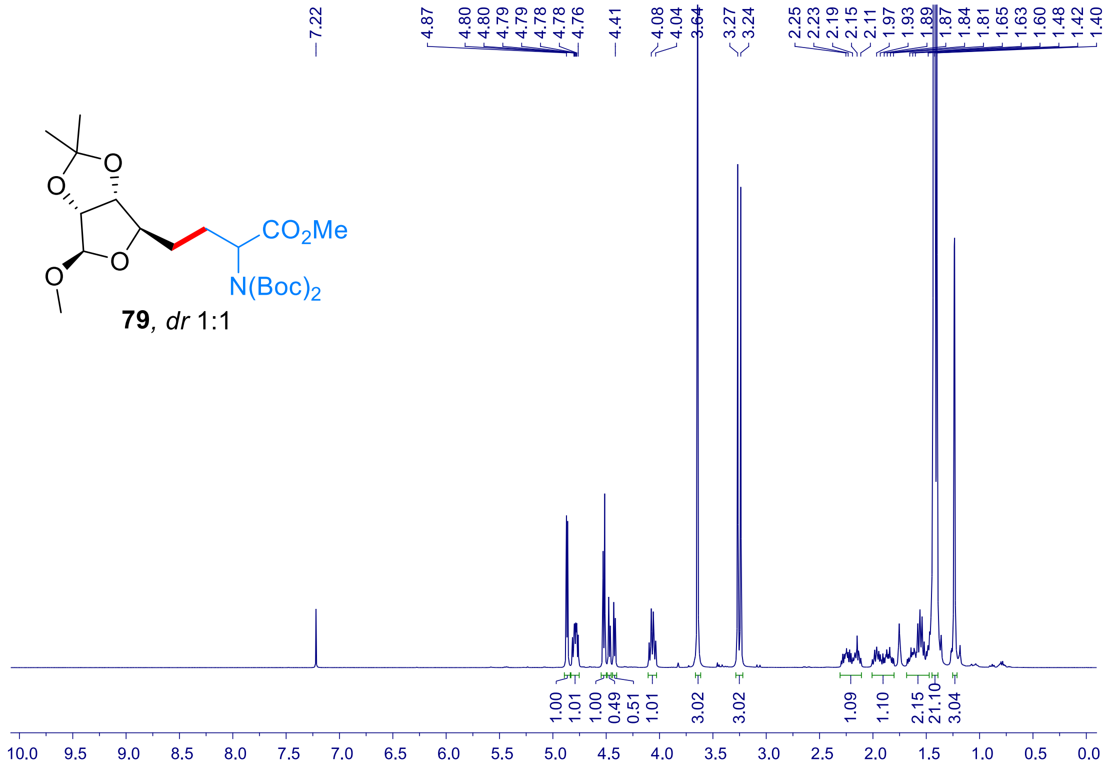

(79)-^{13}C NMR (101 MHz, $CDCl_3$)

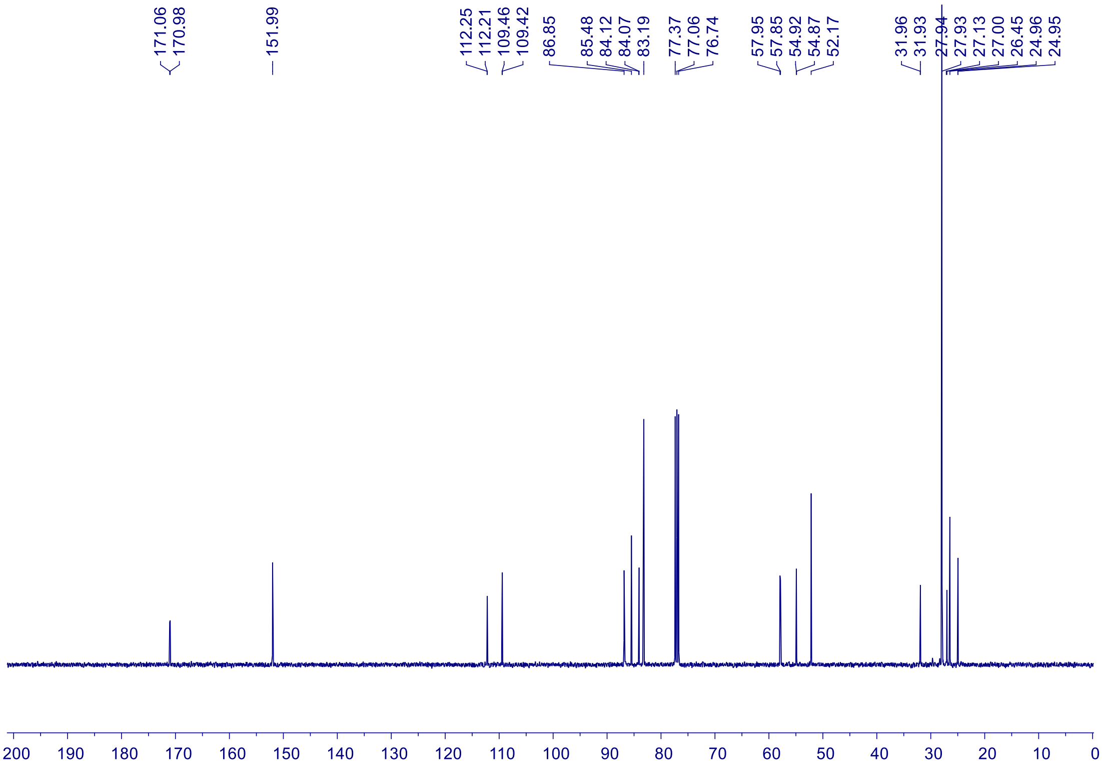

Methyl 2-(Bis(*tert*-butoxycarbonyl)amino)-4-((*3aR,6S,6aR*)-6-hydroxy-2,2dimethyltetrahydrofuro[2,3-d][1,3]dioxol-5-yl)butanoate (80)-^{1}H NMR (400 MHz, $CDCl_3$)

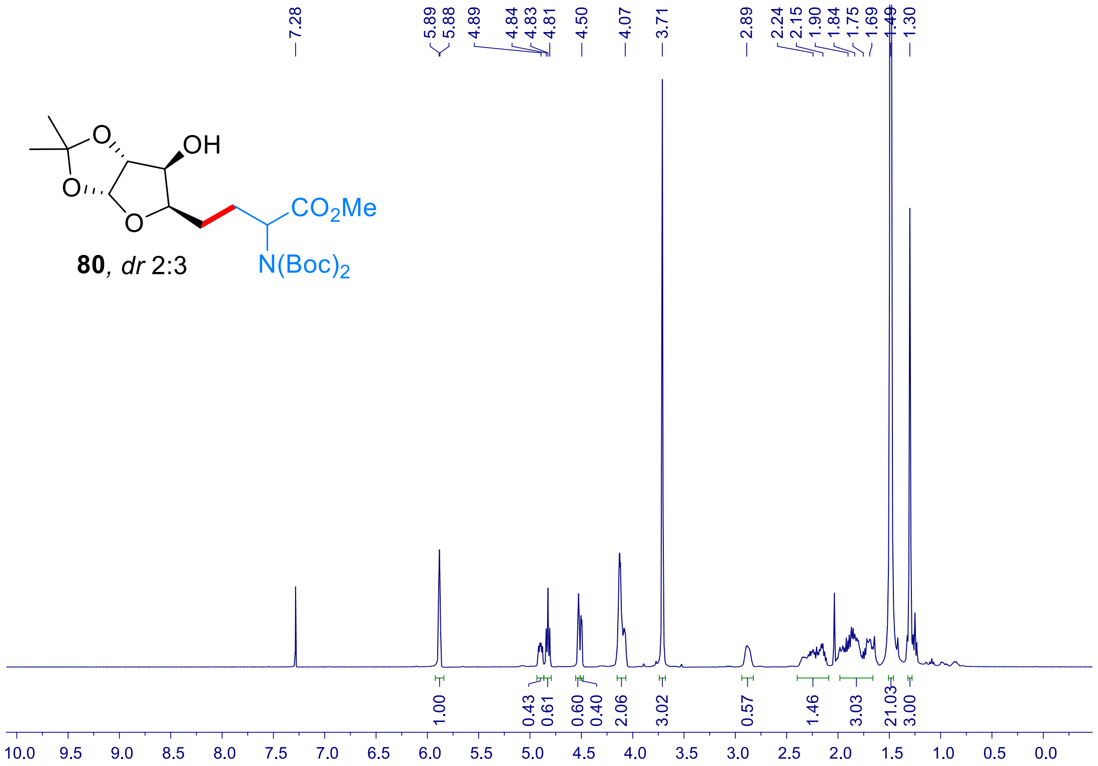

(80)-^{13}C NMR (101 MHz, $CDCl_3$)

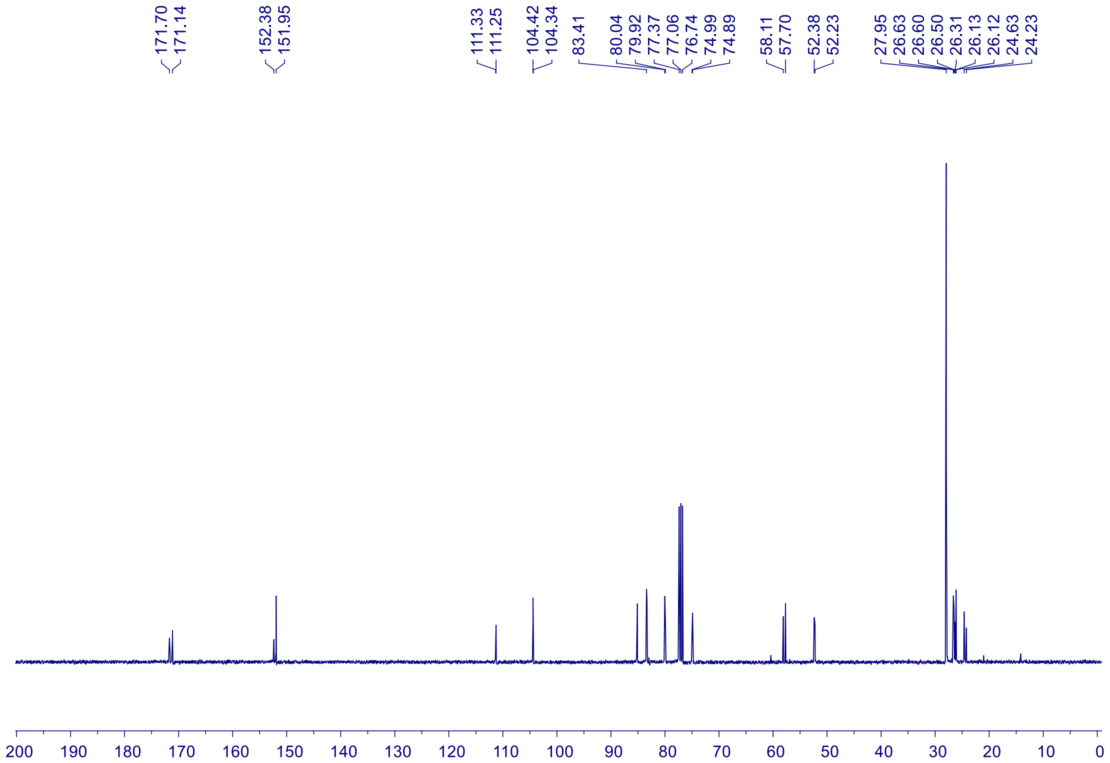

Methyl 2-(bis(tert-butoxycarbonyl)amino)-3-((3aR,5S,6S,6aR)-5-((R)-2,2-dimethyl-1,3-dioxolan-4-yl)-2,2-dimethyltetrahydrofuro[2,3-d][1,3]dioxol-6-yl)propanoate (81)-^{1}H NMR (400 MHz, $CDCl_3$)

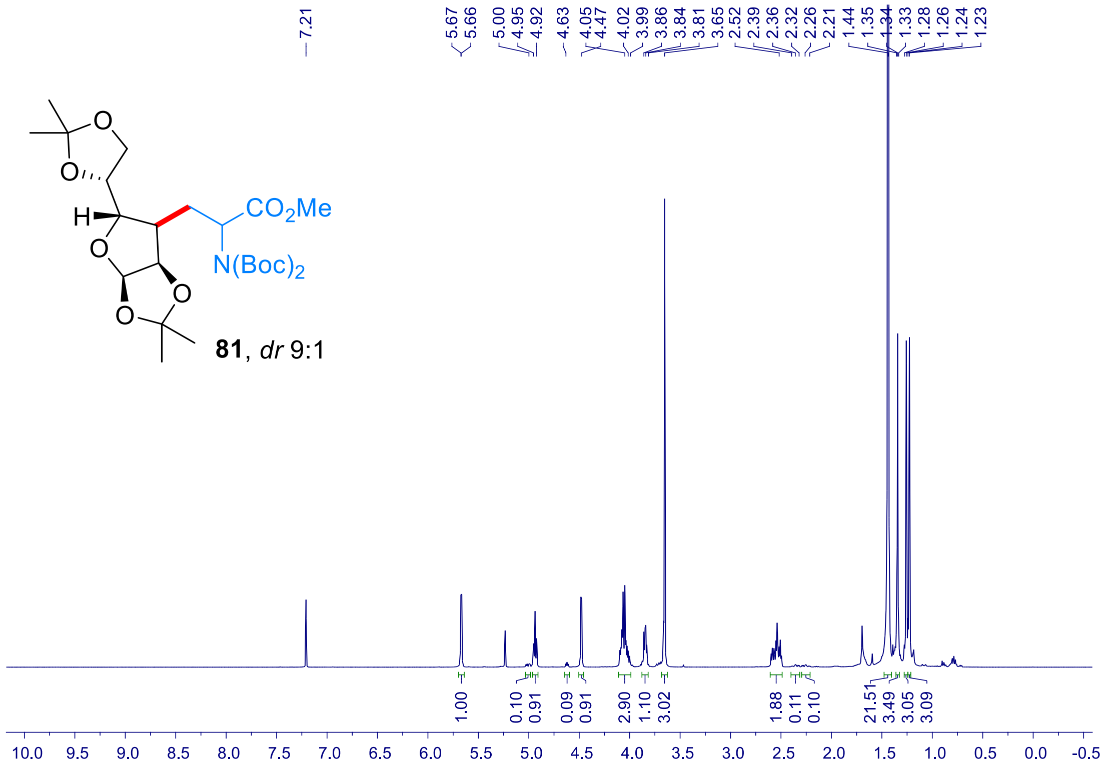

(81)-^{13}C NMR (101 MHz, $CDCl_3$)

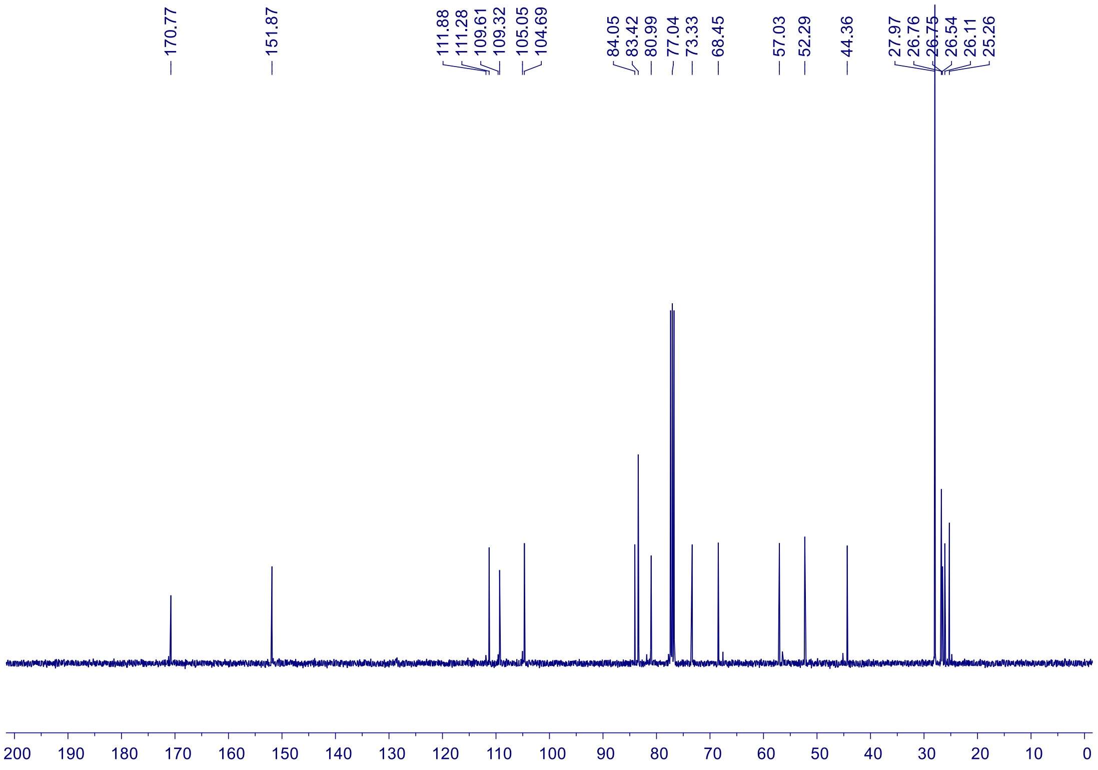

Methyl 2-(bis(tert-butoxycarbonyl)amino)-4-((3aR,5R,5aS,8aS,8bR)-2,2,7,7-tetra methyltetrahydro-5H-bis([1,3]dioxolo)[4,5-b:4',5'-d]pyran-5-yl)butanoate (82)-^{1}H NMR (400 MHz, $CDCl_3$)

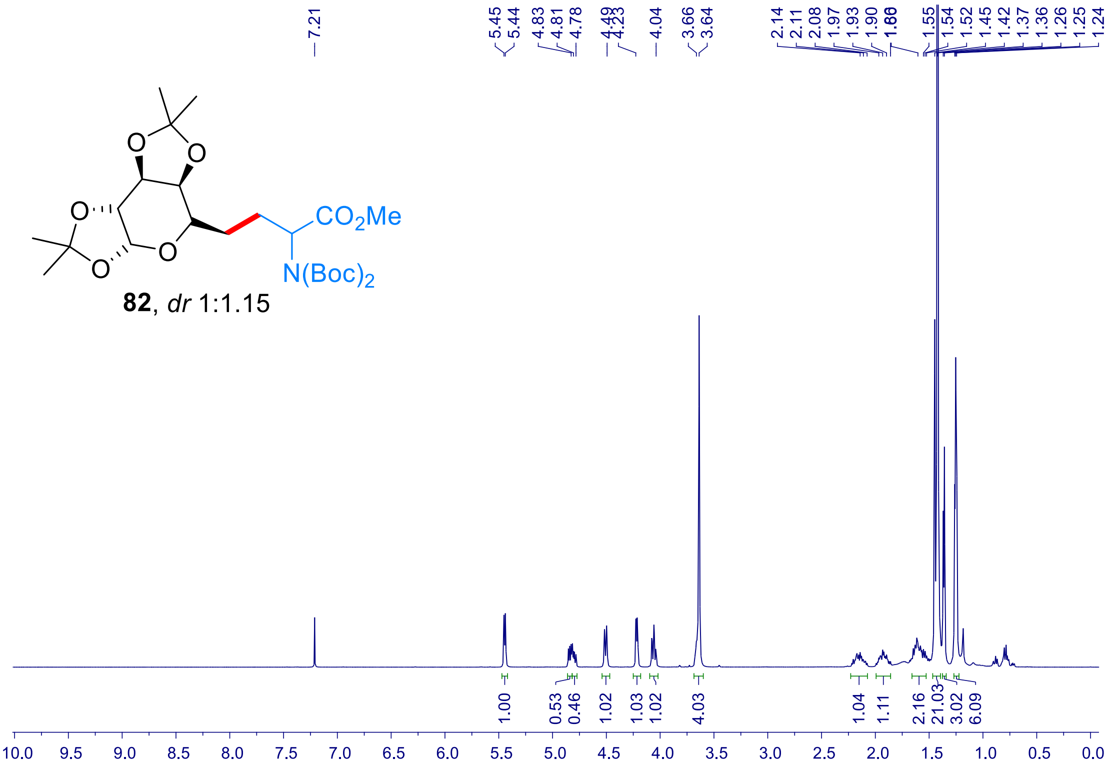

(82)-^{13}C NMR (101 MHz, $CDCl_3$)

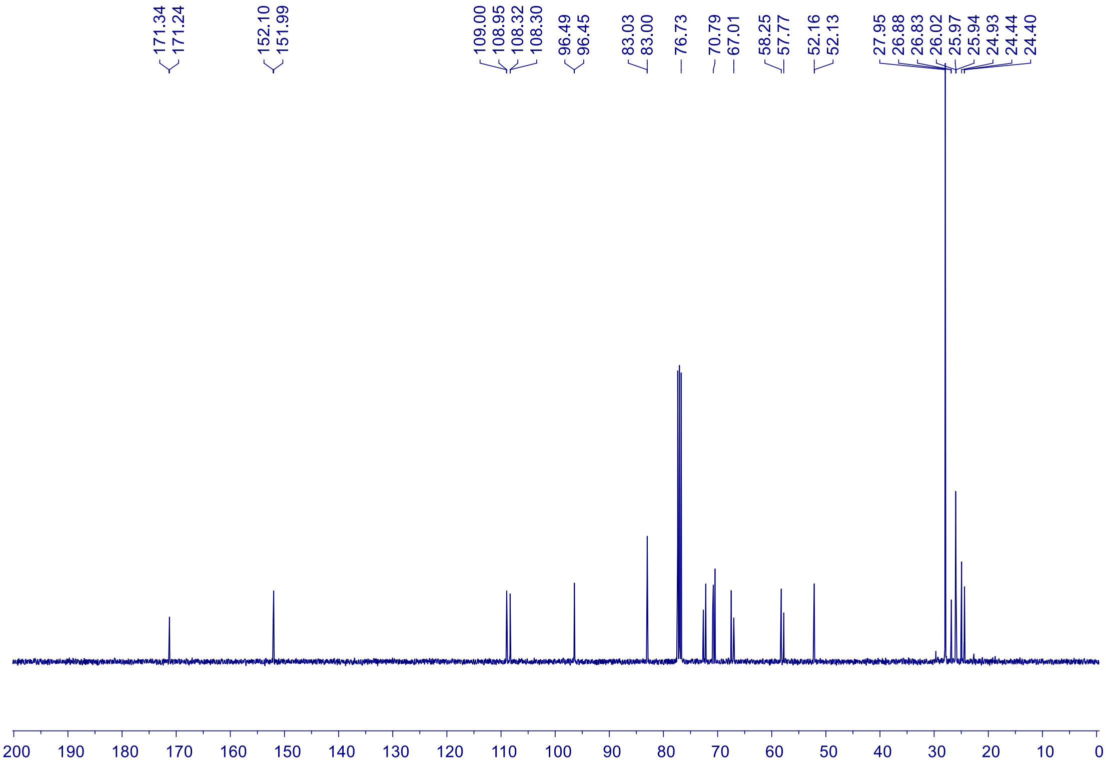

Methyl 2-(bis(tert-butoxycarbonyl)amino)-4-((3aS,5aR,8aR,8bS)-2,2,7,7-tetra methyltetrahydro-3aH-bis([1,3]dioxolo)[4,5-b:4',5'-d]pyran-3a-yl)butanoate(83)-
^{1}H NMR (400 MHz, $CDCl_3$)

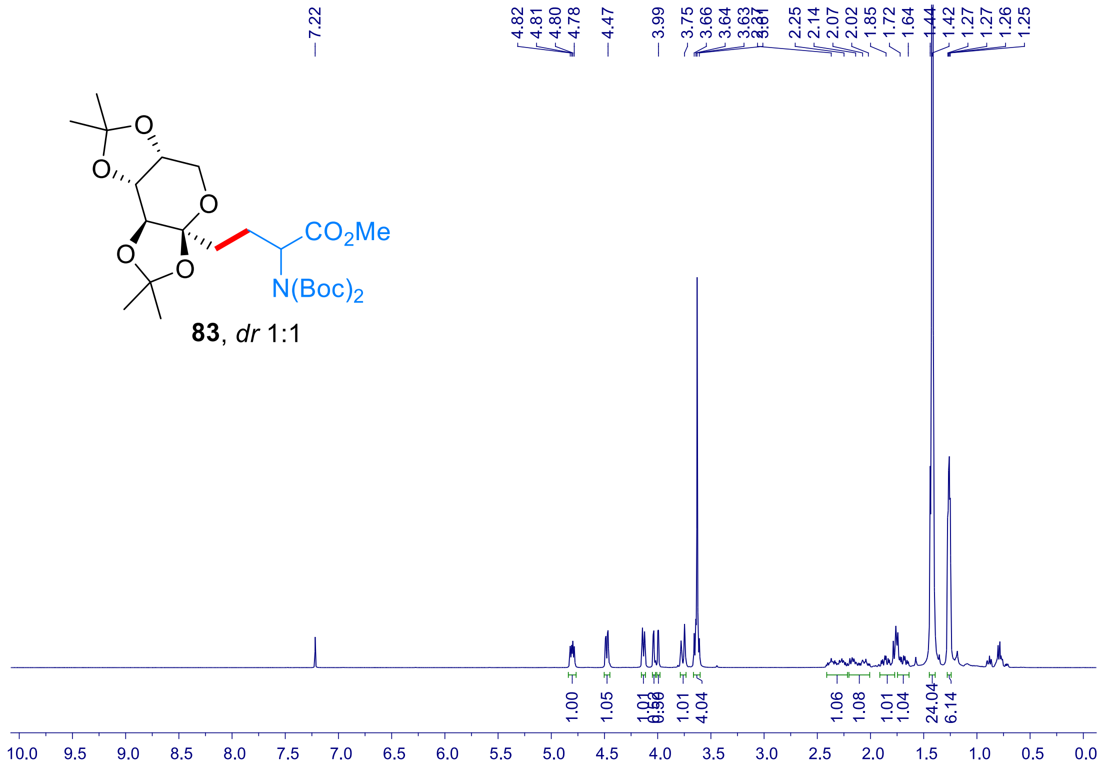

83, *dr* 1:1

(83)-^{13}C NMR (101 MHz, $CDCl_3$)

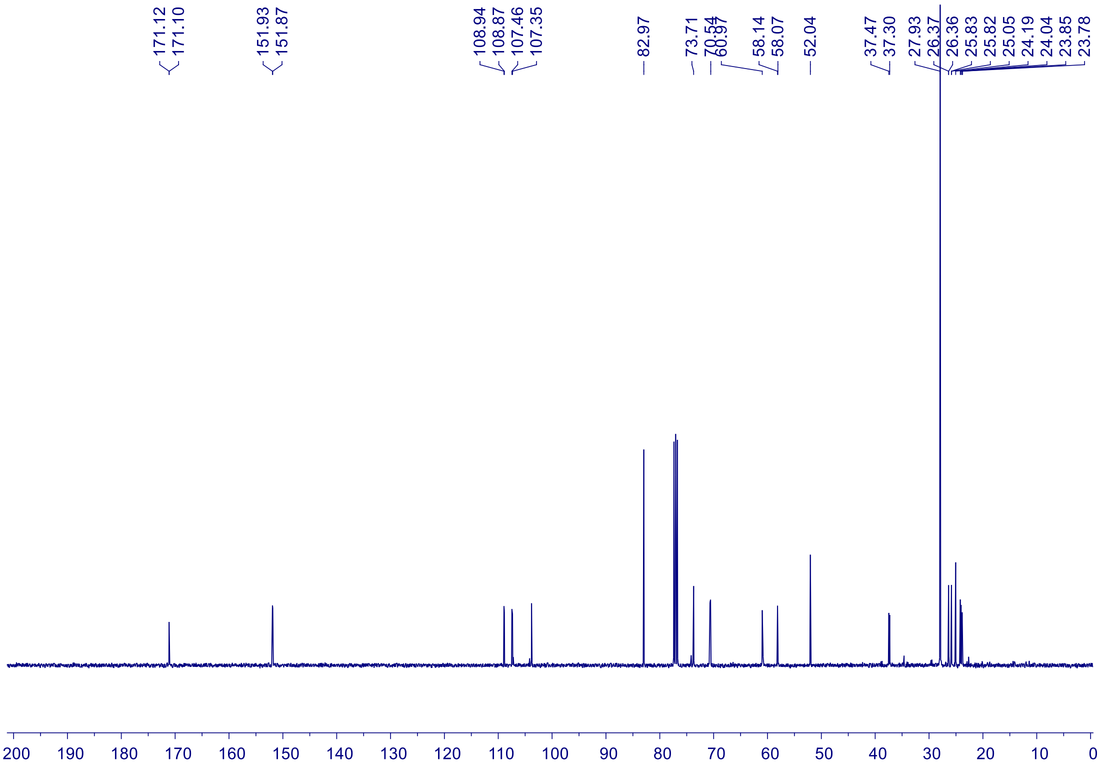

4-(4-(2-cyanoethyl)piperidine-1-carbonyl)-N,N-dipropylbenzenesulfonamide (84)
-^{1}H NMR (400 MHz, $CDCl_3$)

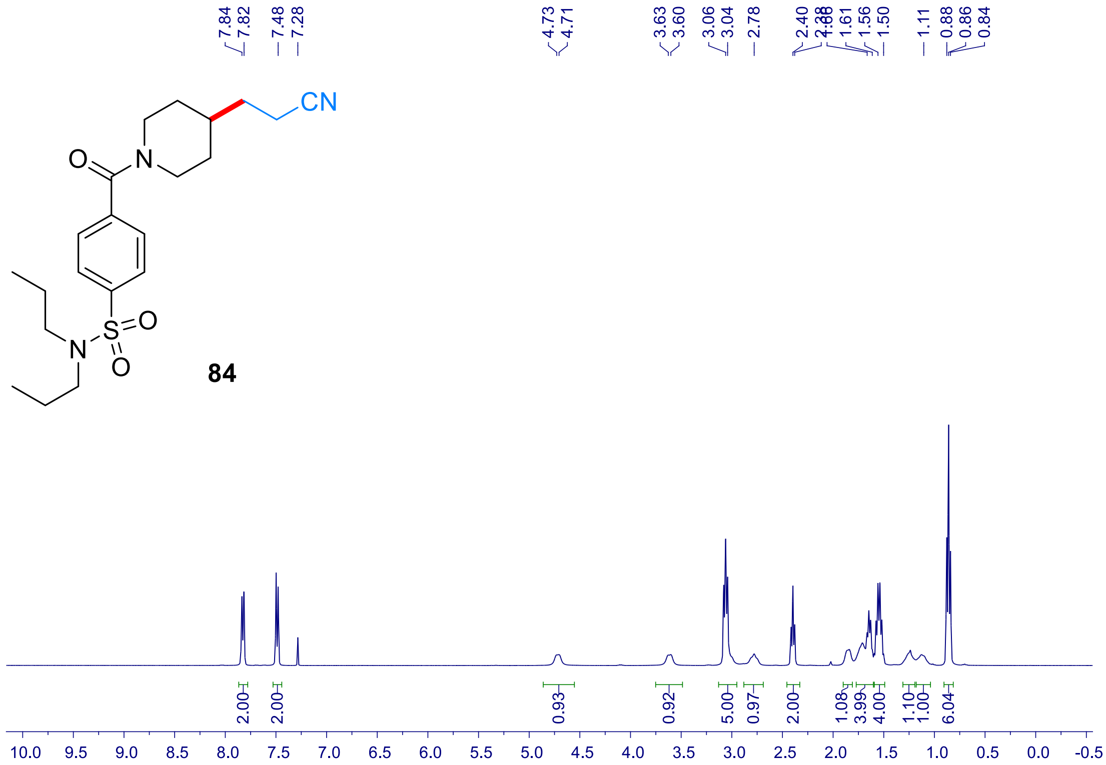

(84)-^{13}C NMR (101 MHz, $CDCl_3$)

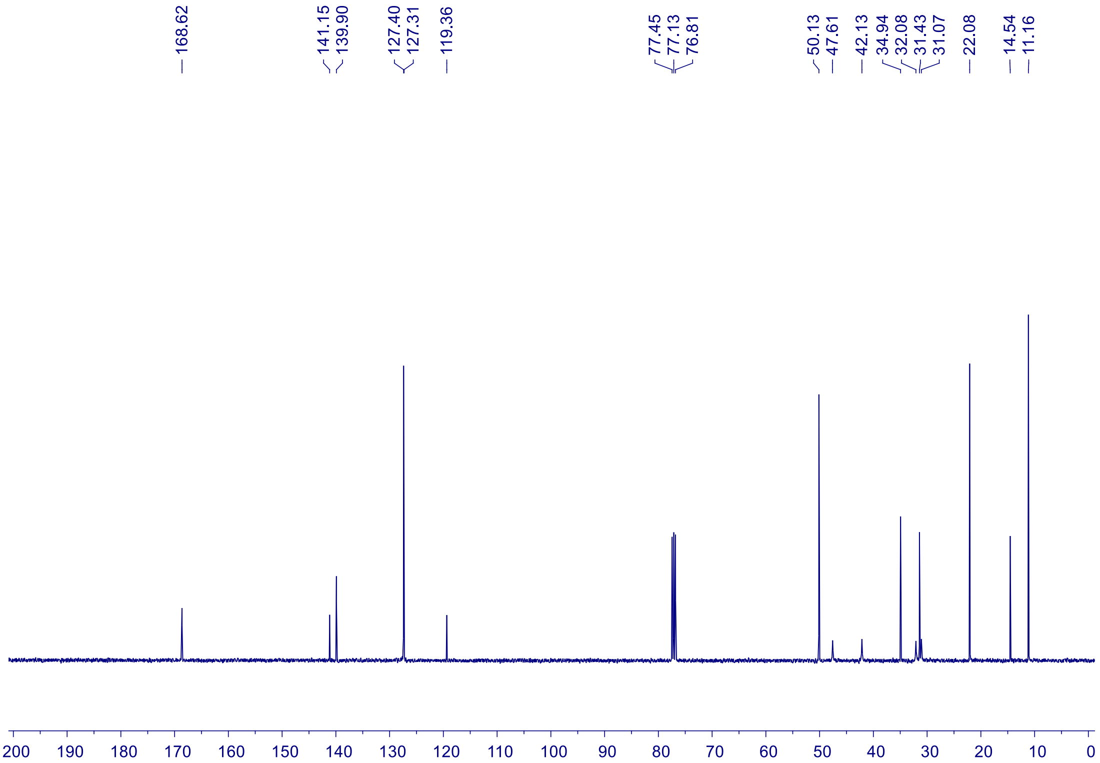

(*R*)-3-(iodomethyl)-1-tosylpyrrolidine (86)-^{1}H NMR (400 MHz, $CDCl_3$)

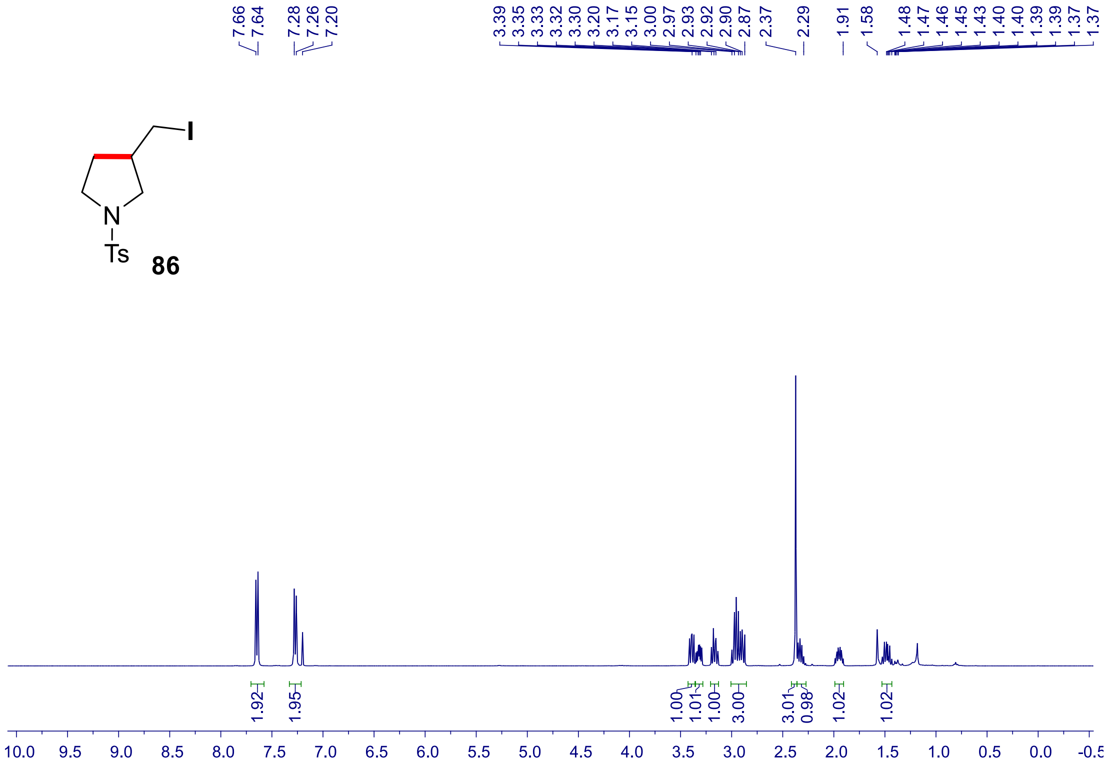

(86)-^{13}C NMR (101 MHz, $CDCl_3$)

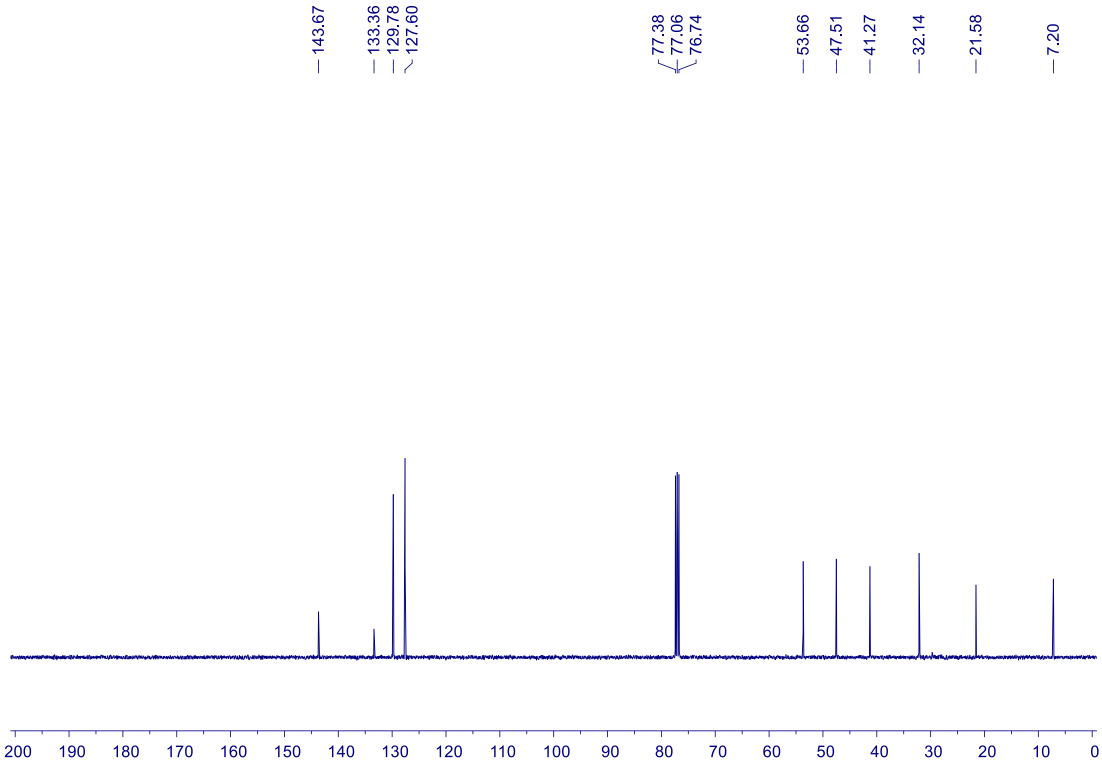

4-(1-tosylpyrrolidin-3-yl)butanenitrile (87a)-^{1}H NMR (400 MHz, $CDCl_3$)

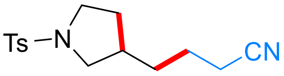

87a

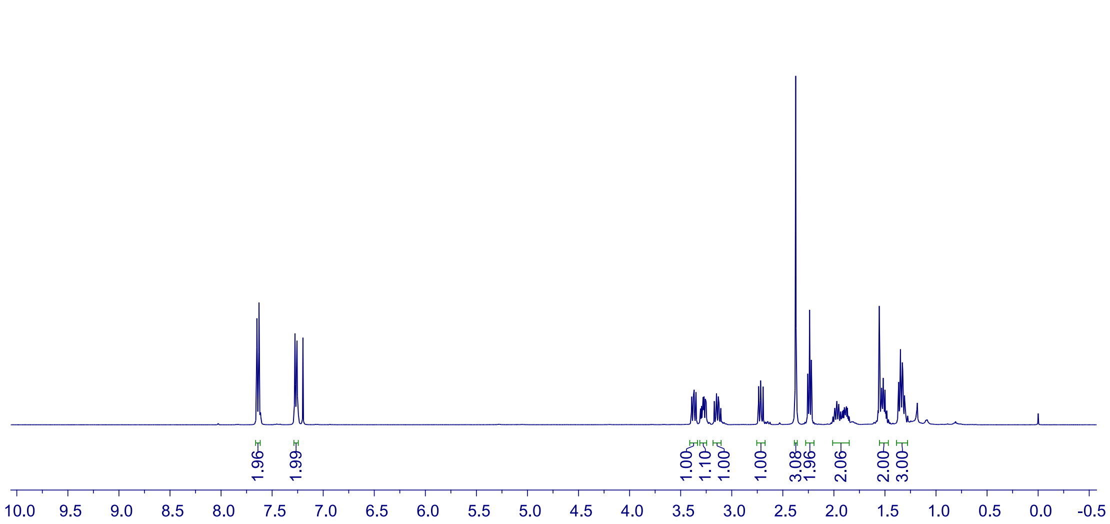

(87a)-^{13}C NMR (101 MHz, $CDCl_3$)

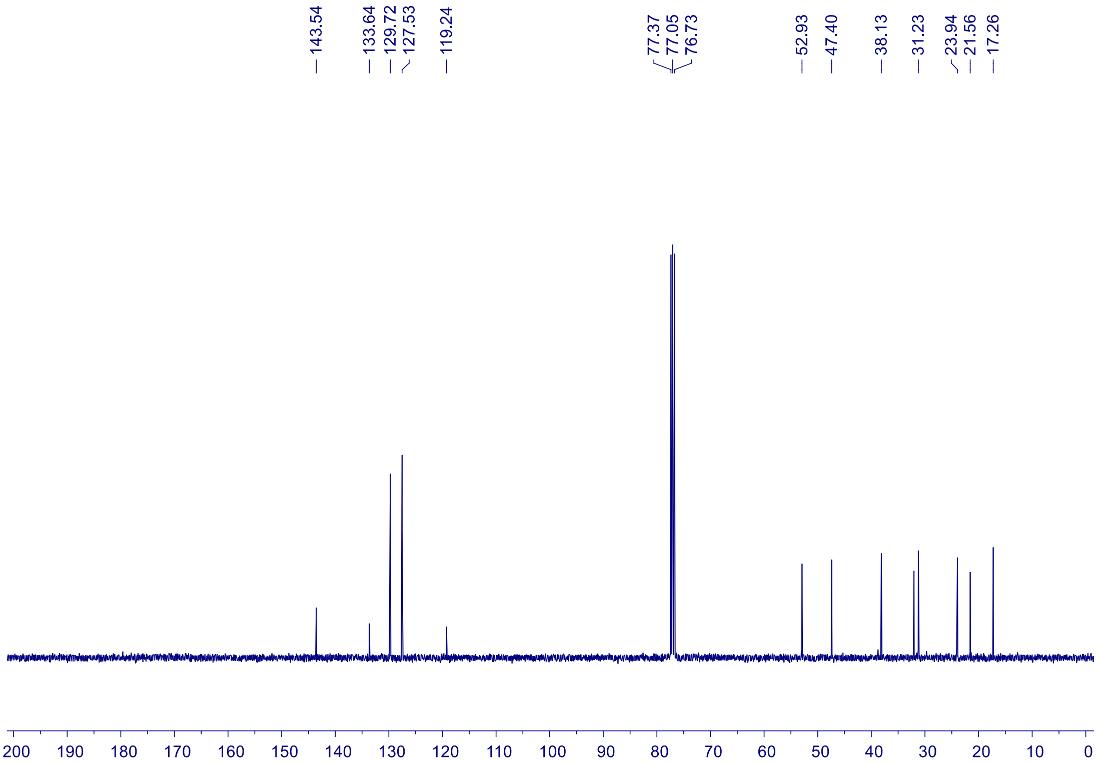

***tert*-butyl 4-(1-tosylpyrrolidin-3-yl)butanoate (87b)**-^{1}H NMR (400 MHz, $CDCl_3$)

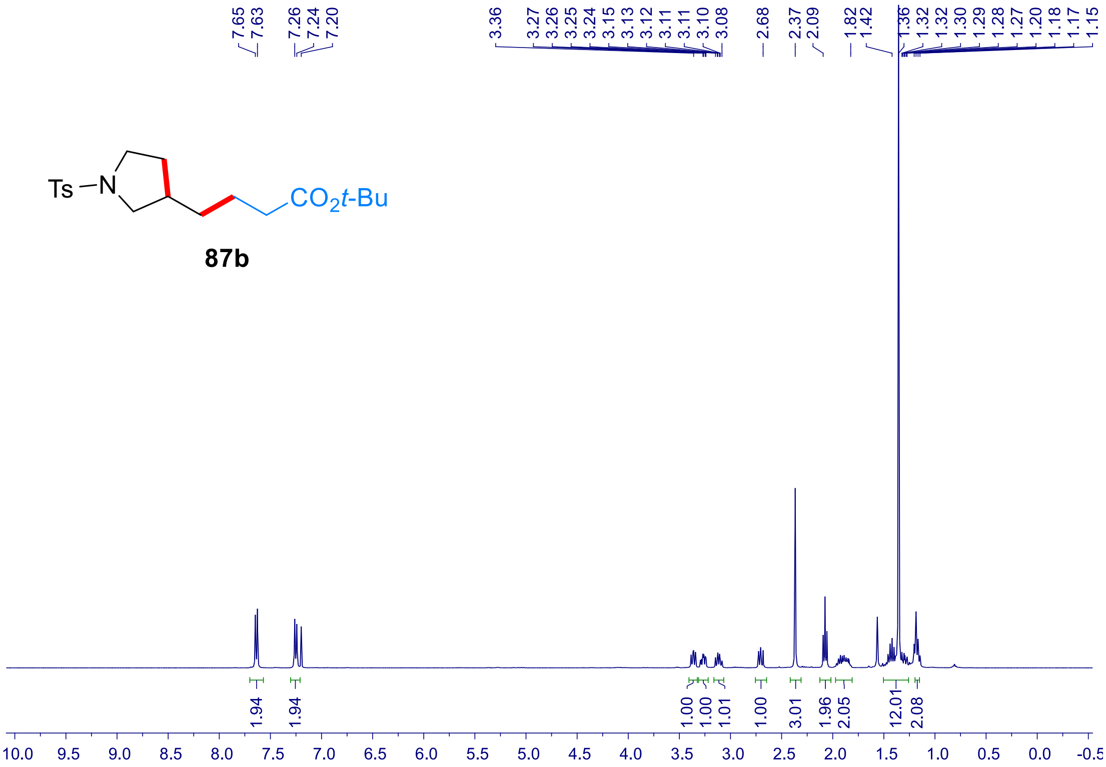

(87b)-^{13}C NMR (101 MHz, $CDCl_3$)

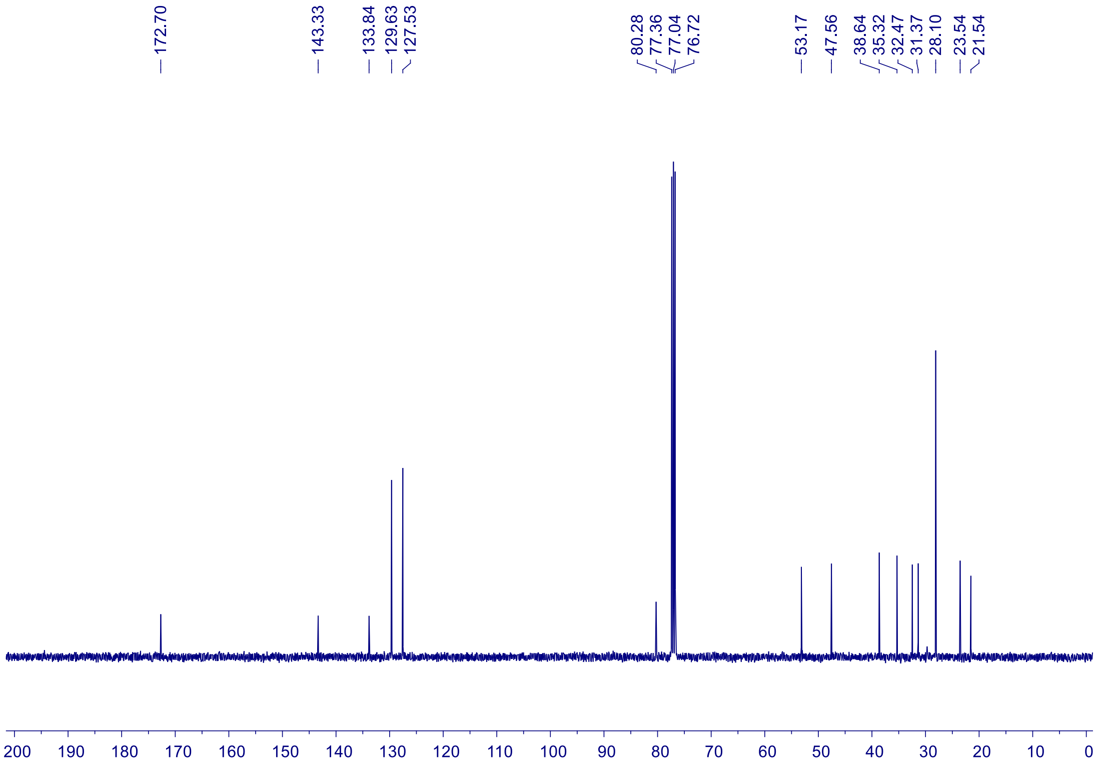

Methyl 2-(Bis(tert-butoxycarbonyl)amino)-4-(tosylpyrrolidine-4-yl)butanoate (87c)-^{1}H NMR (400 MHz, $CDCl_3$)

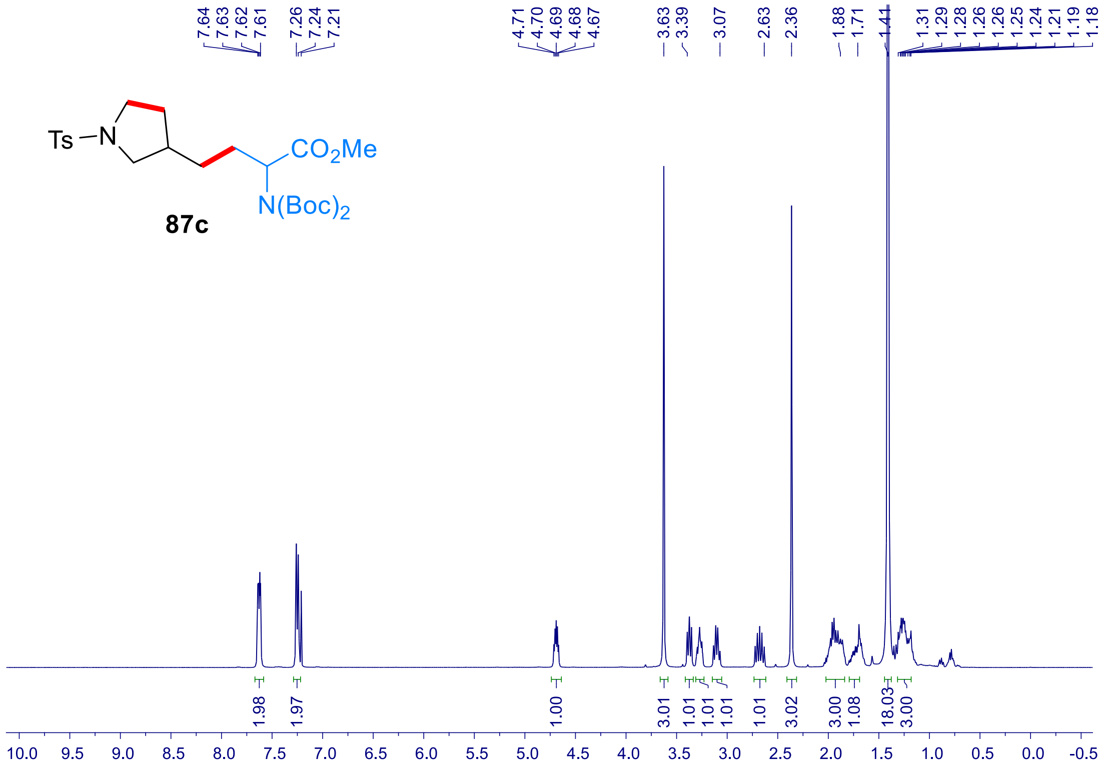

(87c)-^{13}C NMR (101 MHz, $CDCl_3$)

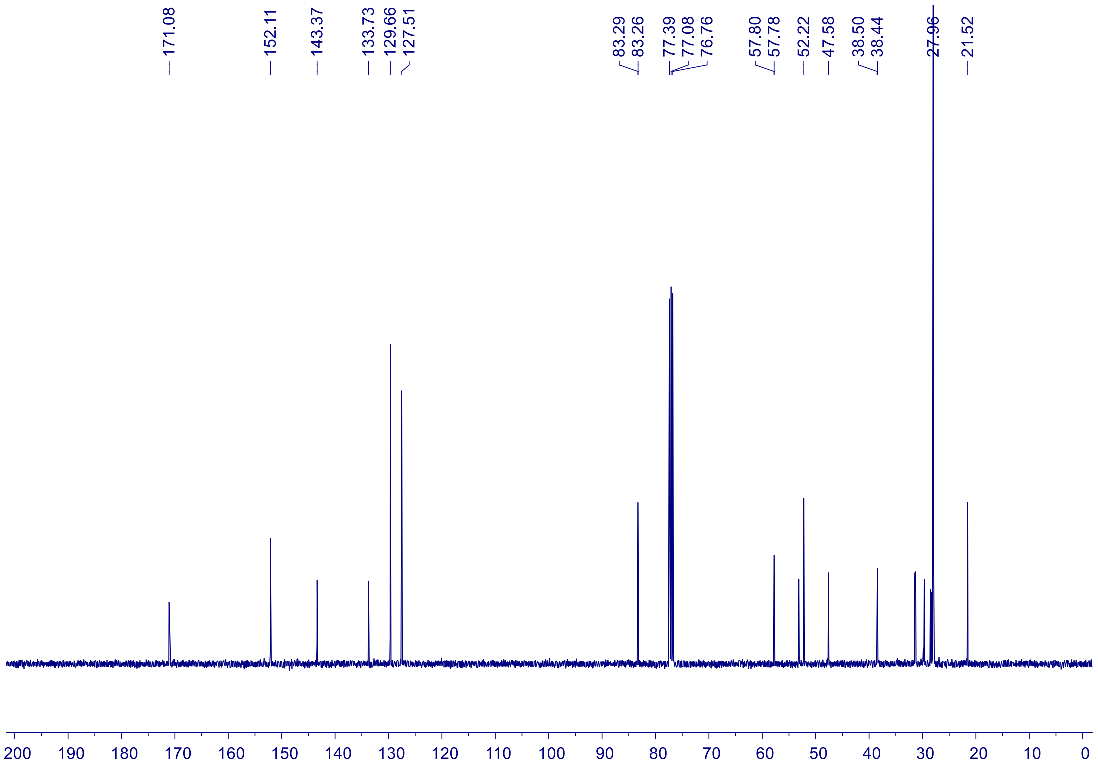

Methyl 2-(bis(tert-butoxycarbonyl)amino)-4-(cyclopropaneyl)butanoate (89) + Methyl 2-(bis(tert-butoxycarbonyl)amino)-hept-6-enoate (90)-^{1}H NMR (400 MHz, $CDCl_3$)

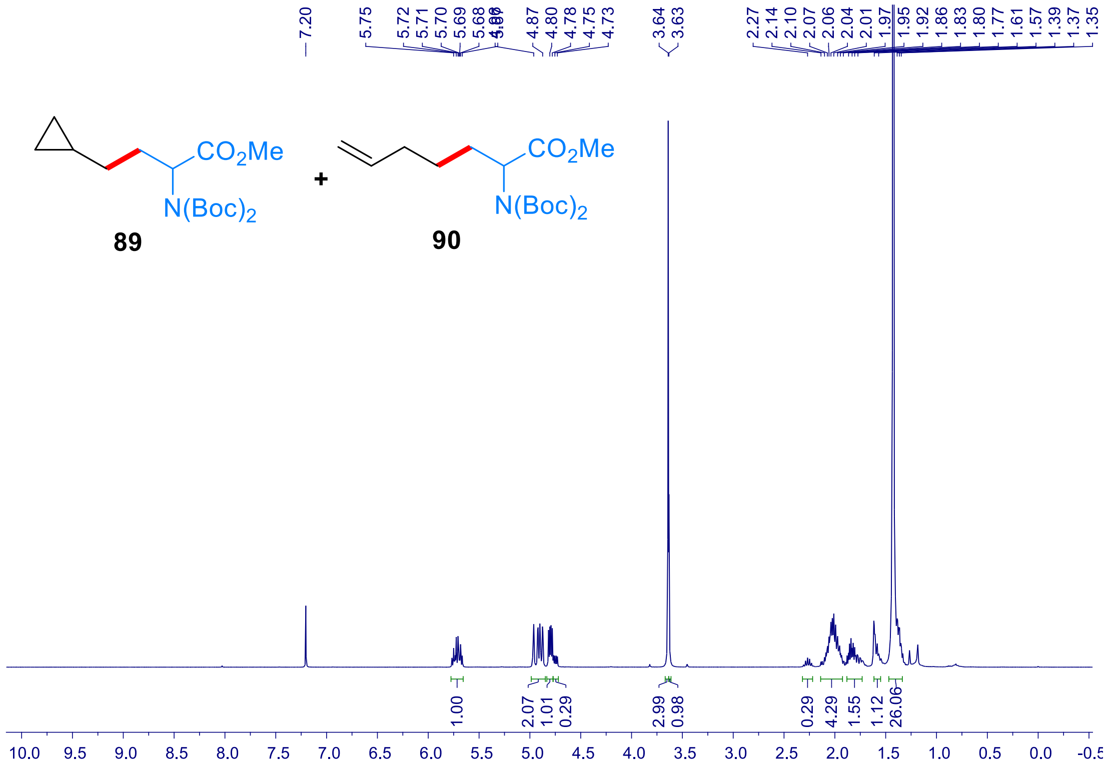

(89)+(90)-^{13}C NMR (400 MHz, $CDCl_3$)

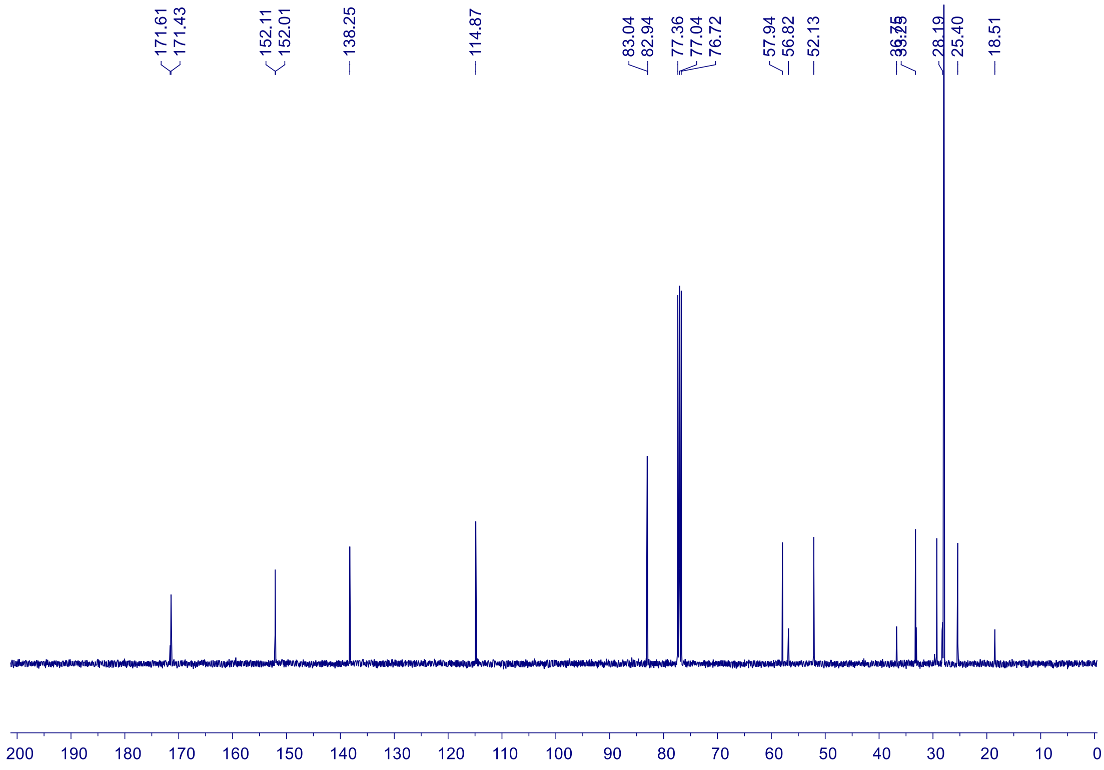

***tert*-butyl 3-(2-cyanoethyl-2-d)azetidine-1-carboxylate (91)-**^{1}H NMR (400 MHz, $CDCl_3$)

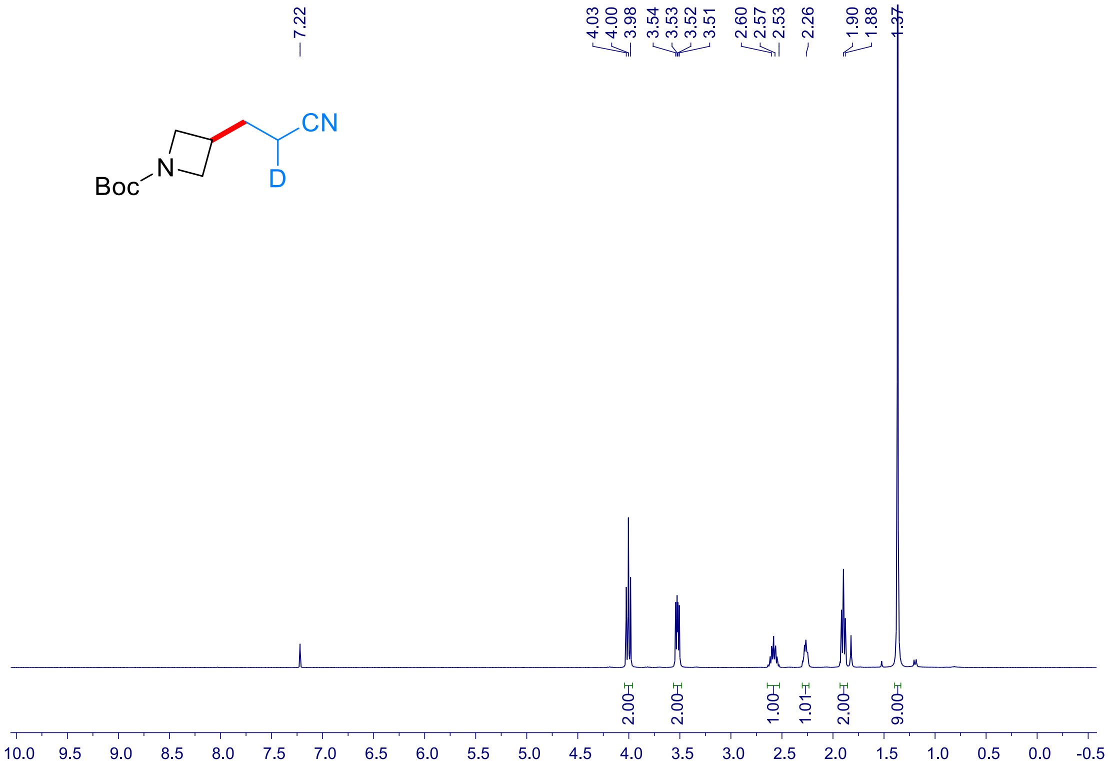

(91)-^{13}C NMR (400 MHz, $CDCl_3$)

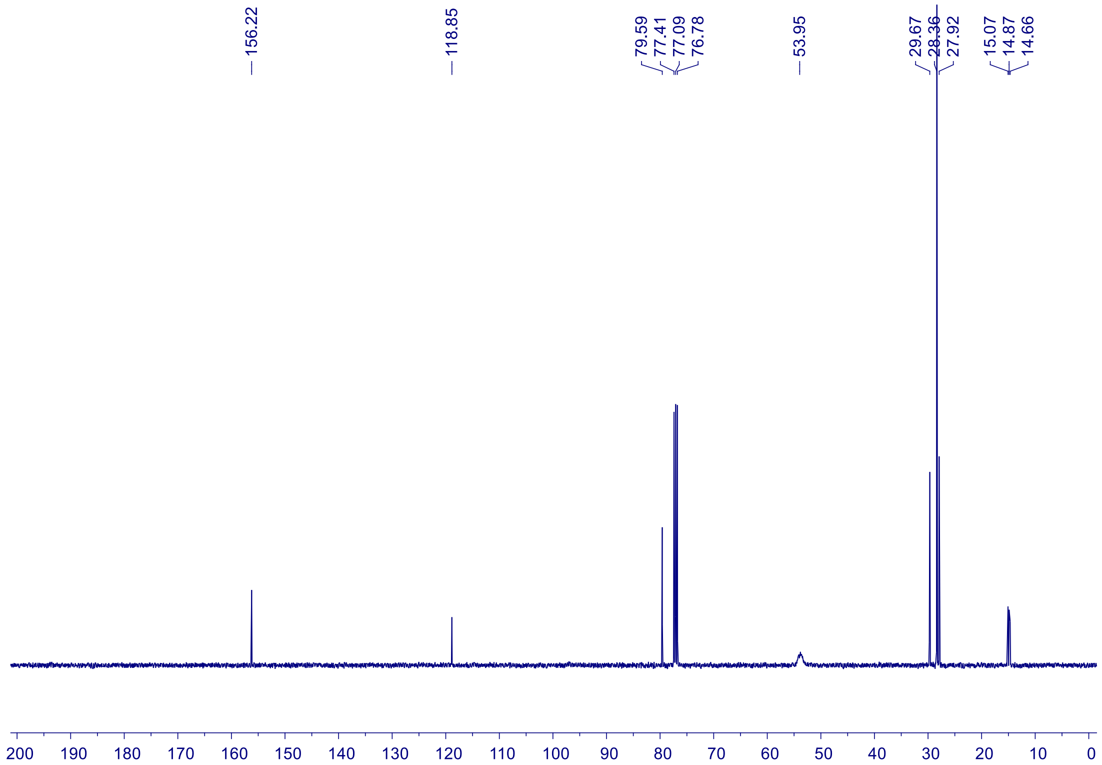

(*R*)-4-(1-(diethylamino)ethyl)benzonitrile (92)-^{1}H NMR (400 MHz, $CDCl_3$)

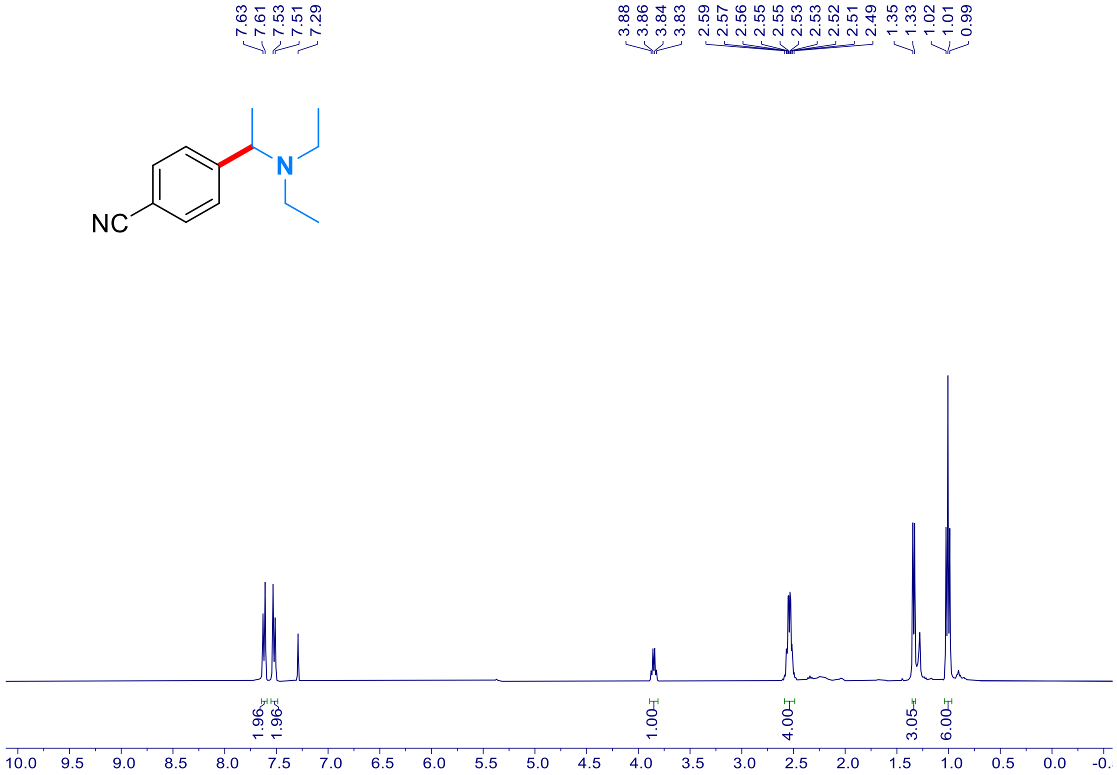

(92)-^{13}C NMR (101 MHz, $CDCl_3$)

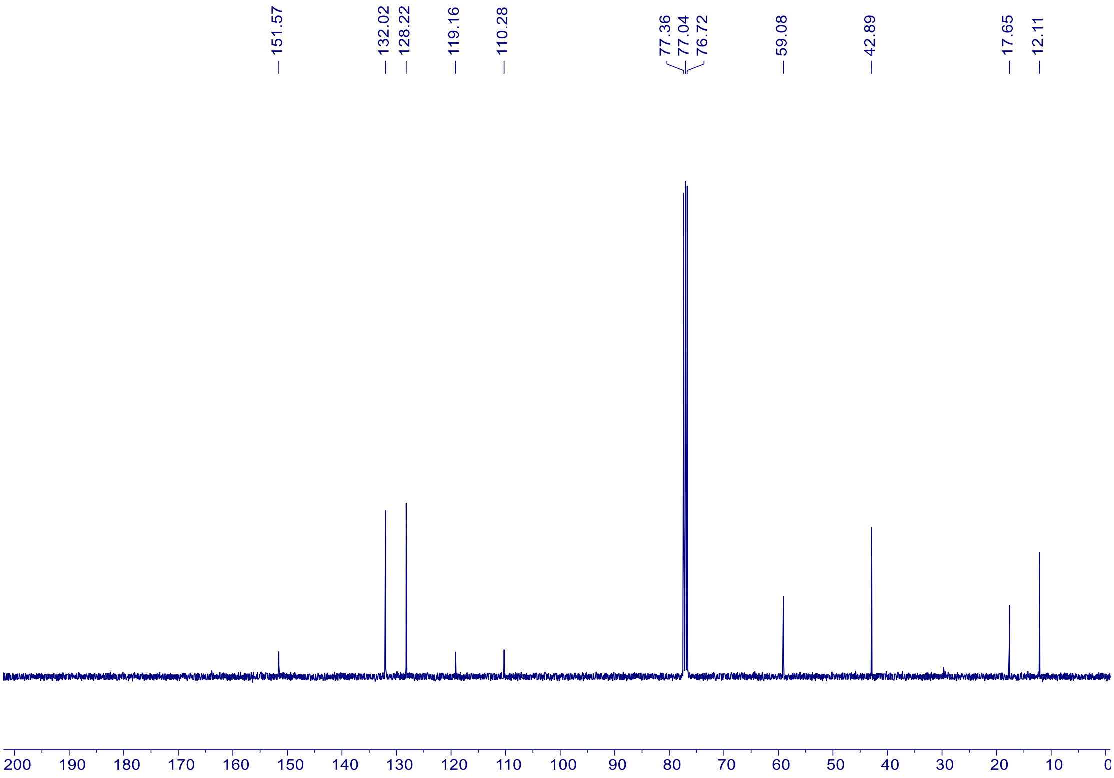

(*R*)-4-(1-(diisopropylamino)ethyl)benzonitrile (93) + 4-(2-(ethyl(isopropyl)amino)propan-2-yl)benzonitrile (94)-^{1}H NMR (400 MHz, $CDCl_3$)

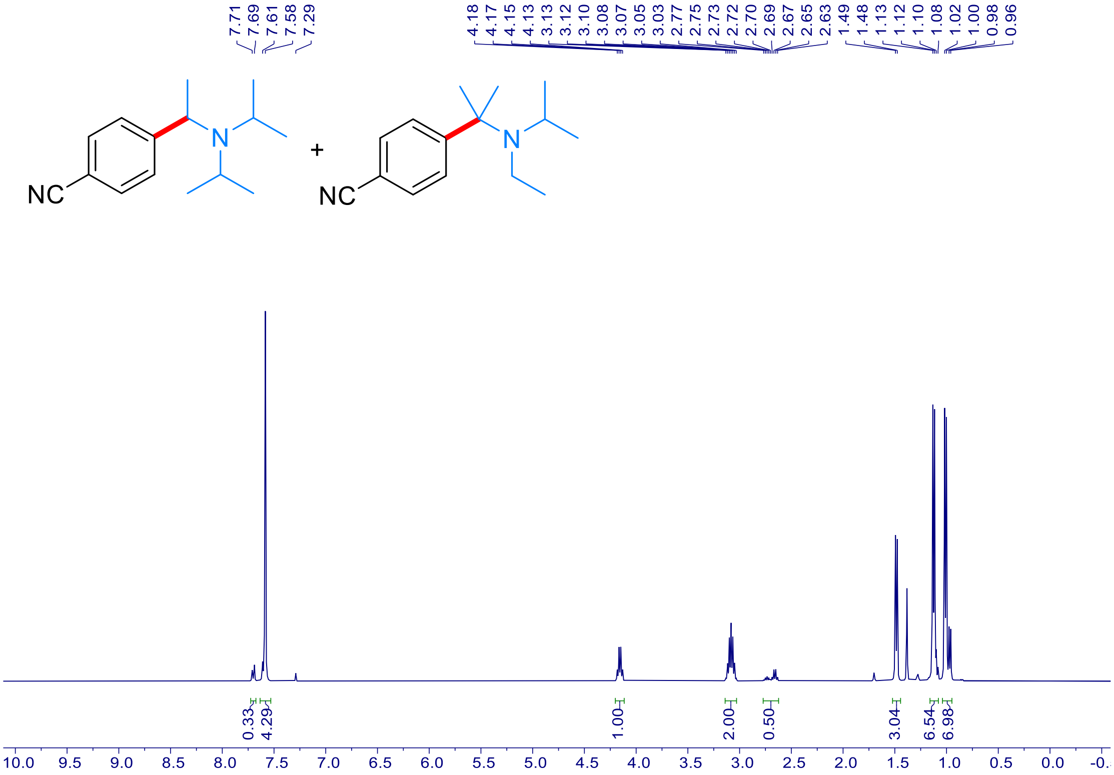

(93)+(94)-^{13}C NMR (101 MHz, CDCl$_3$)

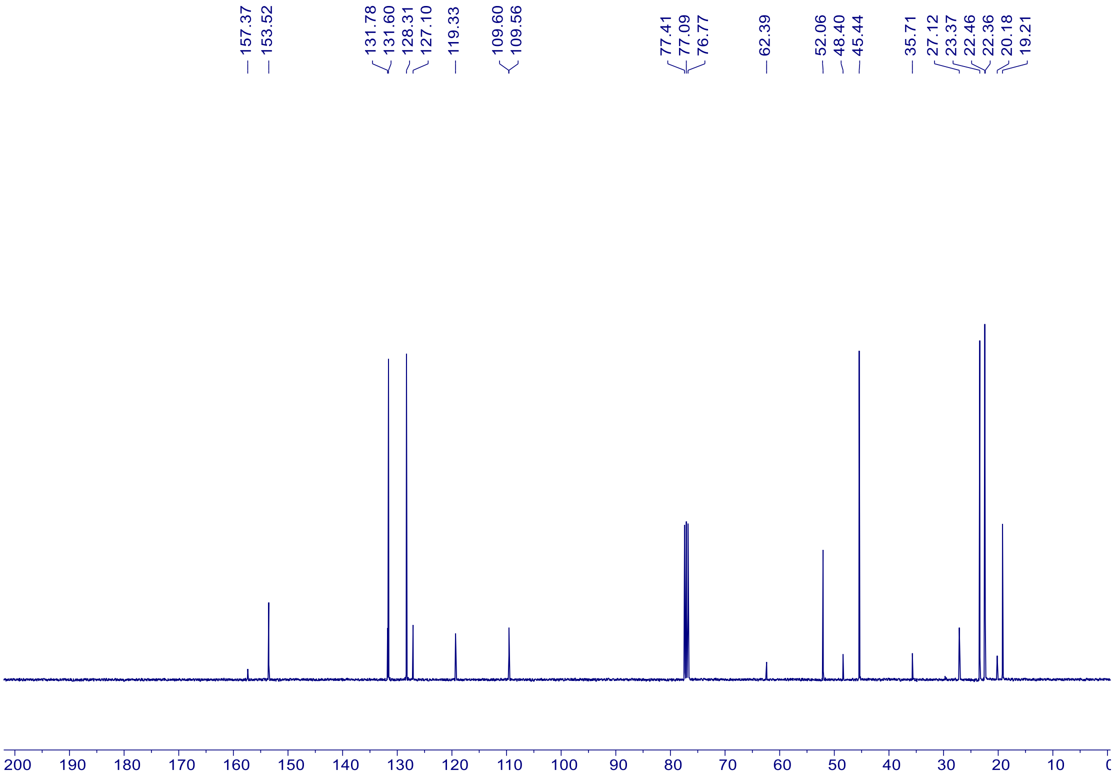

***tert*-butyl 3-(4-cyanophenyl)azetidine-1-carboxylate (95)**-^{1}H NMR (400 MHz, CDCl$_3$)

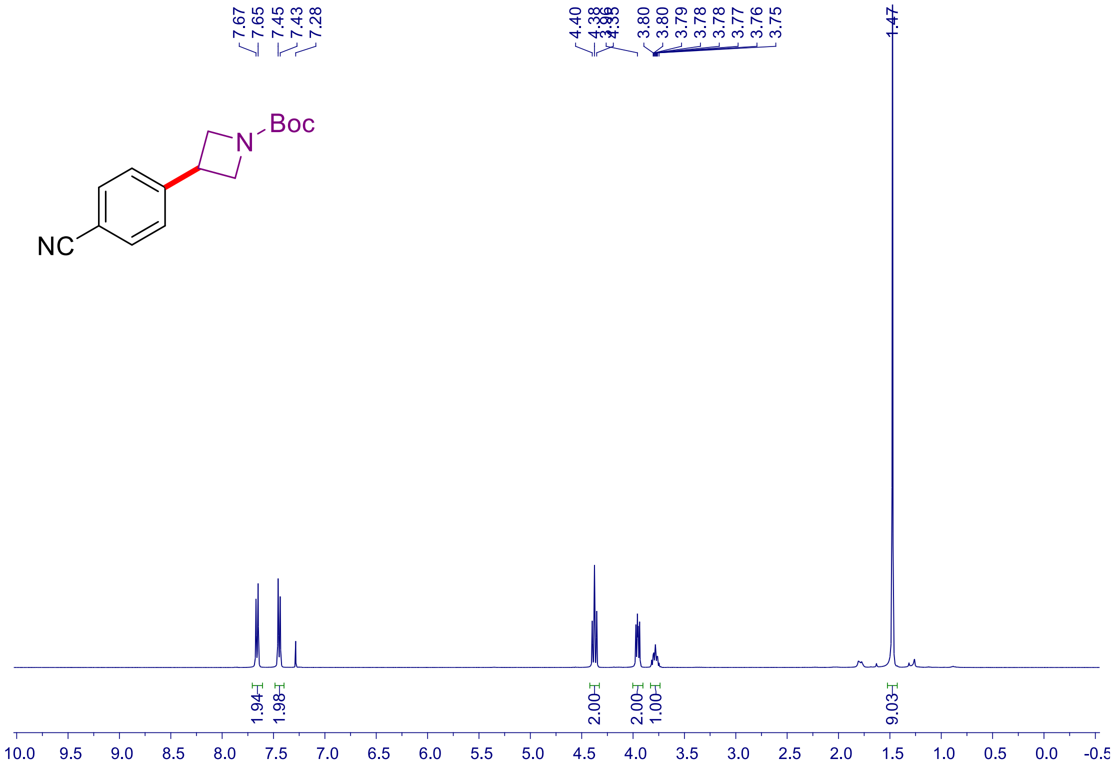

(95)-^{13}C NMR (101 MHz, $CDCl_3$)

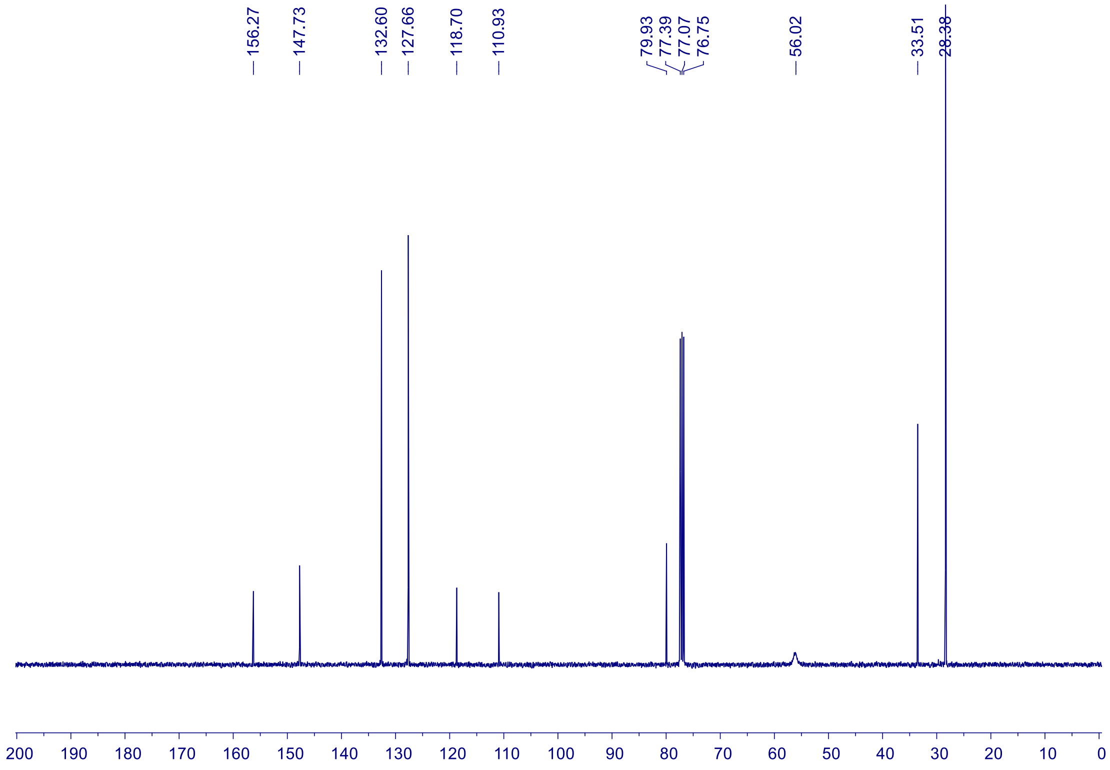

7. References

[1] Constantin, T.; Zanini, M.; Regni, A.; Sheikh, N. S.; Julia, F.; Leonori, D. Aminoalkyl radicals as halogen-atom transfer agents for activation of alkyl and aryl halides. *Science* **2020**, *367*, 1021−1026.

[2] Zhang, Z.; Górski, B.; Leonori, D., Merging Halogen-Atom Transfer (XAT) and Copper Catalysis for the Modular Suzuki−Miyaura-Type Cross-Coupling of Alkyl Iodides and Organoborons. *J. Am. Chem. Soc.* **2022**, *144*, 1986−1992.

[3] Caiger, L.; Sinton, C.; Constantin, T.; Douglas, J. J.; Sheikh, N. S.; Juliá, F.; Leonori, D., Radical hydroxymethylation of alkyl iodides using formaldehyde as a C1 synthon. *Chem. Sci.* **2021**, *12*, 10448.

[4] Niu, B.; Sachidanandan, K.; Blackburn, B. G.; Cooke, M. V.; Laulhé, S., Photoredox Polyfluoroarylation of Alkyl Halides via Halogen Atom Transfer. *Org. Lett.* **2022**, *24*, 916−920.

[5] Chen, Y.; Su, L.; Gong, H., Copper-Catalyzed and Indium-Mediated Methoxycarbonyl ation of Unactivated Alkyl Iodides with Balloon CO. *Org. Lett.* **2019**, *21*, 4689−4693.

[6] Guisán-Ceinos, M.; Soler-Yanes, R.; Collado-Sanz, D.; Phapale, V. B.; Buñuel, E.; Cárdenas, D. J., Ni-Catalyzed Cascade Cyclization–Kumada Alkyl–Alkyl Cross–Coupling, *Chem. Eur. J.* **2013**, *19*, 8405.

[7] Qian, D.; Hu, X., Ligand-Controlled Regiodivergent Hydroalkylation of Pyrrolines. *Angew. Chem. In. Ed.* **2019**, *58*, 18519.

[8] Cai, A.; Yan, W.; W, C.; Liu, W., Copper-Catalyzed Difluoromethylation of Alkyl Iodides Enabledby Aryl Radical Activation of Carbon–Iodine Bonds. *Angew.Chem. Int. Ed.* **2021**, *60*, 27070–27077.

[9] Zhang, Q.; Huang, Y.; Zhan, L-W.; Tang, W-Y.; Hou, J.; Li, B-D., Photoredox-Catalyzed α-C(sp^3)-H Activation of Unprotected Secondary Amines: Facile Access to 1,4-Dicarbonyl Compounds. *Org. Lett.* **2020**, *22*, 7460-7464.

[10] Delgado, J. A. C.; Correia, J. T. M.; Pissinati, E. F.; Paixao, M. W., Biocompatible Photoinduced Alkylation of Dehydroalanine for the Synthesis of Unnatural α-Amino Acids. *Org. Lett.* **2021**, *23*, 5251-5255.

[11] Aycock, R. A.; Pratt, C. J.; Jui, N. T. Aminoalkyl Radicals as Powerful Intermediates for the Synthesis of Unnatural Amino Acids and Peptides. *ACS Catal.* **2018**, *8*, 9115–9119.

[12] Ma, Y.; Yao, X.; Zhang, L.; Ni, P.; Cheng, R.; Ye, J. *Angew.Chem. Int. Ed.* **2019**, *58*, 16548 –16552.

[13] Wang, J.; Qin, T.; Chen, T.-G.; Wimmer, L.; Edwards, J. T.; Cornella, J.; Vokits, B.; Shaw, S. A.; Baran, P. S. *Angew. Chem. Int. Ed.* **2016**, *55*, 9676−9679.
